# A first-in-human, phase I/IIa trial of the T cell immunotherapy personalised tumour trained lymphocytes in patients with advanced colorectal cancer: Study protocol

**DOI:** 10.1371/journal.pone.0351697

**Published:** 2026-09-18

**Authors:** Ahmed Tarfy, Andrea Salmén, Guro Gafvelin, Hans Grönlund, Abbas Chabok, Maziar Nikberg, Per J. Nilsson, Mattias Carlsten, Maximilian Kordes, Sofia Berglund

**Affiliations:** 1 Department of surgical sciences, UppsalaUniversity, Sweden; 2 Centre for clinical research Västmanland, Uppsala University, Västerås, Sweden; 3 Neogap Therapeutics AB, Stockholm, Sweden; 4 Department of Clinical Neuroscience (CNS), Karolinska Institutet, Stockholm, Sweden; 5 Department of Clinical Sciences, Division of Surgery, Danderyd Hospital, Karolinska Institutet, Stockholm, Sweden; 6 Medical Unit Pelvic Cancer - Colorectal Cancer, Karolinska Comprehensive Cancer Center, Karolinska University Hospital, Stockholm, Sweden; 7 Medical Unit Cell Therapy and Allogeneic Stem cell Transplantation (ME CAST), Karolinska Comprehensive Cancer Center, Karolinska University Hospital, Stockholm, Sweden; 8 Phase I-unit, Centre for Clinical Cancer Studies, Karolinska Comprehensive Cancer Center, Karolinska University Hospital, Stockholm, Sweden; Goethe University Hospital Frankfurt, GERMANY

## Abstract

**Background:**

Adoptive T cell therapy is a promising alternative to conventional therapies for certain solid tumours. Personalized tumour-trained lymphocytes (pTTL) is an autologous T cell therapy derived from tumour-draining regional lymph nodes (RLNs), trained to target patient-specific neoantigens arising from tumour-specific mutations. The protocol for an ongoing phase I/IIa first-in-human trial of pTTL in Stage IV Colorectal Cancer (CRC), NEOGAP-CRC-01, is presented here.

**Methods:**

pTTL is produced by *in vitro* expansion of T cells from RLNs using EpiTCer® technology. Tumour-specific neoantigen-forming mutations are identified through next-generation sequencing of tumour and blood samples and the most optimal neoantigens are selected using the bioinformatics software PIOR®. Selected neoantigen epitopes are included in recombinantly produced proteins and attached to paramagnetic EpiTCer® micro-particles, forming the tumour-selective T cell stimulus TC0301 used in pTTL manufacturing. The trial comprises three parts: **Part I** includes sequencing of tumour and blood samples, RLNs collection, TC0301 production and pTTL manufacturing. **Part II** includes preconditioning, pTTL administration and 26 weeks follow-up, and **Part III** consists of up to five years follow-up after pTTL administration. Up to 16 patients can be included. pTTL is administered as a single-dose infusion following preconditioning with cyclophosphamide and fludarabine. Between the limits of 20 x 10^6^–1 x 10^9^ cells, the entire pTTL yield will be administered.

**Discussion:**

pTTL represents a novel approach within adoptive T-cell therapy, using autologous T cells trained to target selected neoantigens without genetic modification. The integration of PIOR® and EpiTCer® technology enables individualized neoantigen identification and delivery. Interpretation of treatment efficacy may be challenging due to the highly personalized nature of pTTL and the heterogeneity of CRC. The primary trial endpoint is safety. Secondary endpoints include objective treatment response, overall survival, and progression-free survival. Biomarkers of pTTL persistence, product characteristics, and treatment response will be assessed.

**Trial registration:**

Authorized under the Clinical Trial Regulation (Regulation EU No 536/2014), EU CT #2024-512296-13-00. **ClinicalTrials.gov ID** NCT05908643

## Introduction

Colorectal cancer (CRC) is one of the most common cancer forms in the industrialized countries, with over 1.9 million new cases and nearly 1 million CRC-related deaths annually [1,2]. In Sweden, approximately 7000 cases are diagnosed annually [3]. Surgery is the primary treatment modality, often combined with chemotherapy, and, for rectal cancer, radiotherapy, particularly in more advanced cases. Over 90% of patients with stage I-II tumours achieve long-term survival. In contrast, patients with metastatic disease (around 20–25% of patients at diagnosis) [2] have poor prognosis, with a five-year survival rate below 20%, despite standard-of-care (SOC) treatment. The SOC in cases where a curative approach is possible typically includes resection or ablation of metastatic lesions, systemic chemotherapy, immunotherapy where indicated, radiotherapy for rectal cancer, and resection of the primary tumour when a curative approach is intended. For patients with unresectable disease who are not candidates for curative treatment, palliative systemic therapy, including chemotherapy or immunotherapy where appropriate, remains the main treatment option [3,4]. The prognosis for stage IV CRC and the efficacy of available standard therapies, including immunotherapies, are variable and depend on several factors, including the molecular characteristics of the cancer [5–9].

Adoptive Cell Therapy (ACT) is a type of advanced immunotherapy where infusion of *in vitro*-manipulated immune cells is used to enhance anti-tumour immunity. This therapeutic strategy has demonstrated efficacy against various malignancies and has the potential to induce deep and lasting responses. Examples of ACTs include chimeric antigen receptor (CAR) T cells [10–12], transgenic T cell receptor (TCR)-modified T cells [13–15], and tumour-infiltrating lymphocyte (TIL) therapy [16–21]. Of these, TIL therapy is most similar to personalized tumour-trained lymphocytes (pTTL), the ACT investigated in this trial.

The rationales for TIL therapy and pTTL are both centred on sourcing T cells from tissue with a natural enrichment of tumour-specific T cells. For TIL therapy, T cells are derived from tumour tissue, where accumulation of anti-tumoral T cells is known to occur. The anti-tumoral potency of this enriched tumour-specific T cell population when administered after *in vitro* expansion to patients with advanced melanoma has been demonstrated in multiple trials over several decades [16–21]. For pTTL, the source of an enriched tumour-specific population of T cells is regional lymph nodes (RLNs). Pre-clinical studies have indicated that T cells sourced from RLNs have the same advantage as TILs, e.g., enrichment of tumour-specific T cells, while maintaining a more functional phenotype than T cells found in tumour tissue [22]. RLNs have been used as a starting material for ACT previously in treatment of CRC and urinary bladder cancer [23–25].

In TIL therapy, T cells are expanded using non-specific *in vitro* stimulation which induces activation, breaks immune tolerance, and promotes proliferation, enabling re-infusion of a large number of T cells made more functional by the production process. The TIL product Lifileucel, under the name AMTAGVI^TM^, is approved in the US for treatment of patients with metastatic melanoma [26]. Although the application of TILs in melanoma has been successful, with durable response rates of more than 20% [16,17,27], it has proven difficult to obtain similar effects in other solid cancers. A phase 2 trial of TIL therapy in gastrointestinal tumours, including 68 patients with stage IV CRC, detected no objective response to unselected TIL. However, responses could be seen when the TIL starting material was selected based on neoantigen-responsiveness, and the response rate was further enhanced when combining with Pembrolizumab [28].

In contrast to TILs, pTTL is produced by *in vitro* activation of T cells against patient-specific neoantigens identified through next-generation sequencing (NGS) [29,30]. The pTTL approach has thus the same advantages as is gained by standard TIL manufacturing (sourcing of an enriched tumour-specific T cell population and *in vitro* expansion), with the additional benefit of training the T cells against neoantigens to achieve the tumour-specific neoantigen reactivity.

The primary objective of this study is to evaluate the safety and tolerability of pTTL in patients with stage IV colorectal cancer. Secondary objectives are to explore preliminary signals of anti-tumour activity and to characterise feasibility of the manufacturing process and treatment delivery. Patients will be closely monitored throughout the study and adverse events systematically recorded and graded.

## Methods

This is a non-randomised, open-label phase I/IIa first-in-human trial investigating the safety and tolerability of the novel advanced therapy medicinal product (ATMP), pTTL, in up to 16 patients with metastatic CRC with limited remaining treatment options.

The recommendations for clinical trials set up by the SPIRIT-CONSORT group to ensure transparent and consistent trial reporting have been taken into regard, see S1 Checklist: Standard Protocol Items: Recommendations for Interventional Trials (SPIRIT) checklist.

### Personalized tumour-trained lymphocytes, pTTL

pTTL is composed of autologous tumour-draining RLN-derived T cells stimulated and expanded *in vitro* with neoantigens. The neoantigens are selected through a process starting with NGS of tumour material and normal tissue (blood). This is followed by analysis and ranking of detected neoantigenic mutations using the bioinformatic software PIOR® (Personalised Immuno-Oncology Ranking), developed by NEOGAP Therapeutics AB. The PIOR® software is optimised for finding the best neoantigen targets according to currently available knowledge. Up to 36 selected neoantigenic epitopes, depending on each patients mutational landscape, are recombinantly expressed as proteins including six neoantigen epitopes each and coupled to paramagnetic beads using the EpiTCer® technology for efficient antigen presentation and T cell stimulation. Each neoantigenic epitope sequence in the recombinant proteins is 21 amino acids long, allowing presentation by both MHC-I and MHC-II, and the micrometre size of the EpiTCer® beads promote efficient phagocytosis by antigen presenting cells (APCs) present in the RLNs material [31]. Recombinant neoantigen epitope-containing proteins linked to the EpiTCer® beads are then processed by the APCs’ natural antigen processing machinery and presented as multiple different shorter peptides containing the neoantigenic mutations on MHC molecules in a manner independent of human leukocyte antigen (HLA) type. The presentation is mediated by both MHC-I and MHC-II, as the delivery of antigens in a particulate form promotes cross-presentation, enabling antigen-induced expansion of both CD4 and CD8 T cells [32–34]. The pTTL production process is initiated by introduction of EpiTCer® beads to a single cell suspension of RLNs cells *in vitro*, inducing neoantigen presentation by the APCs naturally occurring in RLNs to the T cells, resulting in antigen-specific T cell activation and proliferation. T cell expansion is further promoted by addition of interleukin 2 (IL-2). *In vitro* expansion is maintained for approximately two weeks (adjusted to growth kinetics) before harvest of the pTTL product.

### Trial Design

The study is a sponsor-driven trial designed in collaboration with the clinical investigators. Trial design was set up with no direct involvement from patients or members of the public, but the sponsor actively interacts with the Swedish patient’s association for CRC.

**Fig 1:**
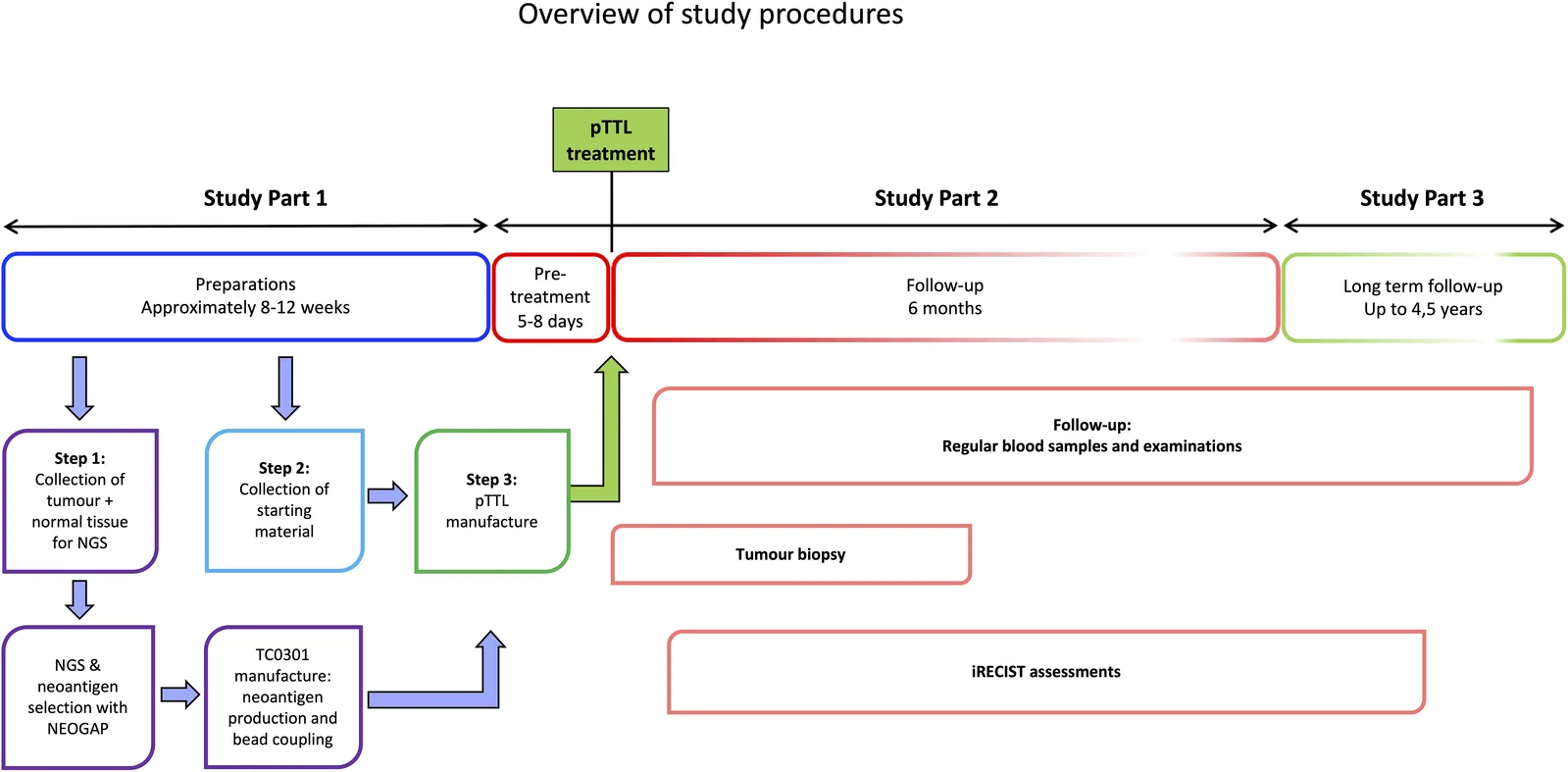
Overview of study procedures. The figure displays the timeline of trial procedures in Part I, II and III including pTTL production steps. The collection of tissue for step 1 and step 2 in Part I can be performed during the same surgery or at separate occasions according to what is most suitable for each included patient. NGS, next generation sequencing; pTTL, personalised tumour-trained lymphocytes; TC0301, neoantigen-coupled EpiTCer® beads.

The study is performed on three sites in Sweden, two Recruitment and Surgery Sites (the Department of Surgery, Västmanlands hospital, Västerås, and the Department of Surgery and Urology, Danderyd’s Hospital AB) and a Recruitment, Surgery & Treatment Site (including three medical units: the Phase 1 unit, Medical Unit Cell therapy and Allogeneic stem cell transplantation (ME CAST), and Medical Unit Pelvic cancer – Colorectal cancer, at the Karolinska University Hospital, Stockholm). It is composed of three parts (Fig 1) in which patients participate sequentially. Initially, patients will provide written consent to be pre-screened for eligibility before participation in the study. Study subjects will then provide separate written informed consent for participation in Part I (tissue material collection and pTTL production) and for participation in Part II-III (pre-conditioning, pTTL treatment, and follow up) of the study. The current version of the clinical trial protocol is found as a Supporting information file.

**Pre-screening** encompasses identification of potential participants at local multidisciplinary team (MDT) conferences. All potentially eligible patients subsequently undergo a secondary central imaging review performed in collaboration with an experienced gastrointestinal radiologist and a colorectal surgeon to assess tumour extent, lymph node involvement, and eligibility according to the inclusion and exclusion criteria.

**Part I** of the trial entails the steps to generate pTTL (which will be administered during Part II). No upper limit is set for the number of patients that can be enrolled in Part I, as not all patients included in Part I are expected to be able to proceed to Part II (because of, e.g., rapid disease progression). However, recruitment to Part I is continuously assessed and adjusted to ensure that all patients included in Part I can continue to Part II and receive pTTL administration when they become eligible. The entire pTTL process requires 8–12 weeks and has three main steps:

1. Collection of tumour samples and normal samples for NGS and neoantigen selection, and production of stimulatory EpiTCer® beads carrying neoantigens.
2. Collection of starting material for pTTL manufacturing from the RLNs. When possible, RLNs are obtained during planned surgery of the primary tumour as part of SOC for patients with stage IV disease (palliative local tumour resection or stoma formation). Otherwise RLNs will be obtained during a study-specific surgery performed with the sole aim to harvest starting material for pTTL production [23,24]. (Study-specific surgery is discussed in further detail below.) The starting material will be cryopreserved to facilitate study logistics and increase flexibility of pTTL administration.
3. Manufacturing of pTTL requires approximately two weeks and can be started at any suitable time following the completion of bead production. The pTTL product is cryopreserved to be thawed just before administration to the patient.

**Study-specific surgery** is planned and executed according to the principles of minimally invasive surgery, to achieve the least extensive intervention compatible with safe and effective access to the predefined lymph nodes. Preoperative evaluation includes contrast-enhanced computed tomography (CT) and, when indicated, magnetic resonance imaging (MRI), performed to identify relevant anatomical structures and to define the spatial relationship between the tumour, regional lymph nodes, and adjacent vasculature.

Surgery will be as limited as possible depending on each patient’s individual circumstances, and laparoscopic or robot-assisted lymphadenectomy will be performed whenever feasible. In cases where minimally invasive surgery proves technically unfeasible, for instance, due to dense adhesions or other anatomic constraints precluding safe exposure of the CT-identified lymph node stations, conversion to an open procedure may be considered intra-operatively. A decision to convert to open surgery intraoperatively rests with the operating colorectal surgeon in consultation with the Principal Investigator (PI), with explicit consideration of the balance between procedural risk and potential patient benefit.

All decisions and deviations from the planned minimally invasive approach are documented in the operative record and in the study case report form.

**Fig 2:**
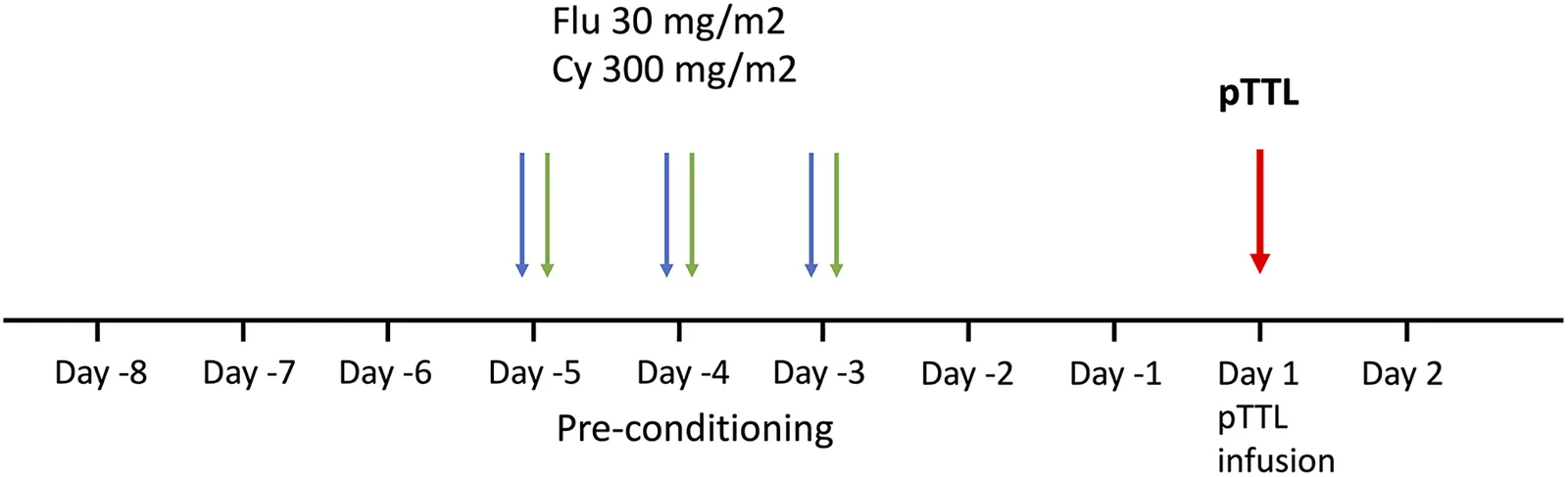
pTTL pre-treatment chemotherapy and administration schedule. Schedule for pre-conditioning and pTTL administration. Preconditioning chemotherapy is administered on three consecutive days, starting on Day-5 and completed on Day-3, in relation to pTTL administration. Exceptions to this schedule are allowed, but the 3-day preconditioning must be finalised earliest 120 hours and latest 48 hours prior to pTTL administration. The patients will receive the pTTL therapy on Day 1 (red arrow). Flu: fludarabine (blue arrows), Cy: cyclophosphamide (blue and green arrows).

**Part II** of the trial entails pre-conditioning, treatment with pTTL and 26 weeks of post-infusion safety follow-up. Up to 16 patients with Stage IV CRC will be included in Part II, with the goal of having 12 evaluable patients. All patients for which an approved pTTL product have been manufactured during part I are assessed for inclusion in Part II. The preconditioning therapy consists of cyclophosphamide, 300 mg/m^2^ patient body surface area (BSA), and fludarabine, 30 mg/m^2^ BSA, on three consecutive days [35]. Standard supportive care medications, including intravenous hydration therapy, antiemetic therapy and anti-microbial prophylaxis will be administered. pTTL is administered a minimum of 48 hours and a maximum of 120 hours after the pre-conditioning has been completed (Fig 2).

**pTTL administration** will be performed as a single intravenous dose within the dose range 20x10⁶ to 10⁹ viable cells. The dose within this range is determined by the recovery of cells in each individual pTTL product. Free dosing within the dose range has been approved by the Swedish medical products agency based on an expected acceptable safety profile, by the unique characteristics of the product and also by regulatory guidance: The characteristics of each batch, including the autologous and thus variable nature of the cells and the patient-specific neoantigens towards which the product is targeted, result in uncertainty regarding the comparability of individual pTTL products. These unique properties also makes the production yield of pTTL vary significantly. The primary objective of the trial is to characterize the safety profile of the feasible dose range rather than identifying a maximum tolerated dose (MTD). This aligns with regulatory guidance that acknowledge that practical limits on dose production or delivery may preclude traditional dose escalation designs for Advance Therapy Medicinal Products (EMA Guidance on quality, non-clinical and clinical requirements for investigational advanced therapy medicinal products in clinical trials (EMA/CAT/22473/2025), FDA guidance “Considerations for the Design of Early-Phase Clinical Trials of Cellular and Gene Therapy (CGT) Products”).

The total duration of each patient’s involvement in Part II is estimated to be approximately 29 weeks including a 14-day screening period, 7 days of pre-administration supportive care therapy (of which 3 days include pre-conditioning), a 28-day dose-limiting toxicity (DLT) observation period, and 26 weeks of safety follow-up after pTTL administration. Patients with disease progression receiving further standard of care therapy after pTTL will remain in the study but transfer from Part II to Part III at the time of confirmed progression, unless withdrawn from the study for other reasons, such as treatment within another clinical trial.

Part II will end when the last treated patient has been followed for 26 weeks or earlier in case of death, withdrawal of this patient or their participation in another clinical trial.

**Part III** of the trial is a long-term follow-up initiated after completion of Part II. The long-term follow-up in Part III will continue for 4.5 years, i.e., in total 5 years follow-up after pTTL administration, or until death of the subject or their withdrawal from the trial.

Part III, and the trial, will end when the last evaluable treated patient has been followed for 5 years after pTTL administration or earlier in case of death, withdrawal from the study or participation in another clinical trial.

### Endpoints

**The primary endpoint** of the trial is safety, based on the frequency and severity of adverse events (AEs) assessed according to CTCAE v5.0. Adverse events of special interest (AESI) correspond to immune-related AEs and AEs known to be associated with T cell therapies, including autoimmune reactions potentially resulting from off-target toxicity such as colitis, and immune-mediated reactions associated with immune cell activation, such as cytokine release syndrome.

Safety in the study is evaluated at regular meetings of an internal safety review committee.

**Secondary endpoints:**

- Objective response (according to iRECIST).
- Characterisation of treatment response:
  - Time to response
  - Duration of response
- Time to tumour progression assessed using the iRECIST criteria (time from inclusion in Part II of the study until disease progression).
- Kinetics of tumour progression/growth (compared to pre-treatment)
- Overall survival (time from inclusion in Part II of the trial to death of any cause).
- Progression-free survival (time from inclusion in Part II of the trial to tumour progression or death of any cause).
- Disease-specific survival (time from inclusion in Part II of the trial to death of disease).

**Exploratory endpoints:**

Evaluation of biomarkers for:

- pTTL persistence. T cell receptor sequencing (TCRseq) will be used to characterise the pTTL product and to trace the identified pTTL clones in peripheral blood.
- pTTL tumour infiltration. If feasible, pre- and post-treatment biopsies will be taken for histological assessment of T cell infiltration and characterisation of tumour-infiltrating T cells.
- pTTL neoantigen specificity and efficacy:
  - Functional T cell assays will be used to evaluate neoantigen-specific responses in the pTTL product and in peripheral blood T cells.
  - Markers of tumour burden will be evaluated.
- pTTL characteristics. Analysis of the pTTL product and peripheral blood samples will be performed to evaluate the functional status, differentiation and lineage-identity of T cells and other immune cells.

Storage of data relevant for the correlation of neoantigen selection to clinical outcome. The type of neoantigens targeted will be assessed in the context of clinical outcomes, signals of efficacy and biomarker evaluation.

### Eligibility

#### Study population and sample size.

- The target group of the study is adults (≥18 years of age) with stage IV CRC who are able to provide written informed consent. Patients are eligible for inclusion if they have received treatments according to SOC or further SOC treatment options are judged not to be in the patients’ best interest at the time (*e.g.,* due to toxicity issues). Inclusion and treatment with pTTL can also occur in a scheduled pause in SOC therapy, if judged appropriate by the responsible investigator.
- Eligible patients must have a primary or metastatic tumour that is accessible to biopsy or surgery to obtain tumour material for NGS analysis.
- In addition, it is a requirement that the patient can undergo surgical excision of regional lymph nodes, draining the primary tumour or a metastasis, for pTTL production.
- No formal limit is set for the number of recruited patients in Part I, to enable replacement for patients that are unable to continue to Part II due to, e.g., disease progression or occurences precluding manufacturing of an approved pTTL product.
- The maximum number of patients included in Part II is 16, aiming to achieve at least 12 evaluable patients.
- Patients with remaining available therapeutic options with a chance of cure are not eligible for inclusion in Part II and pTTL treatment. However, a patient may be eligible for inclusion if further SOC treatment options are judged not to be in the patients’ best interest (e.g., due to toxicity issues) or during a scheduled pause in ongoing palliative therapy, at the discretion of the treating Investigator. Materials for Part II of the study can be collected in Part I while curative therapy options are still available.

#### Eligiblity criteria Part I.

##### Inclusion criteria Part I:

- Signed informed consent.
- Age of 18 years or greater.
- Histological or cytological confirmation of colorectal cancer.
- Metastatic colorectal cancer.
- Measurable disease according to RECIST 1.1.
- ECOG PFS 0–1.
- Minimum life expectancy of 6 months from inclusion in Part I (as per Investigator’s clinical assessment).
- Adequate bone marrow, hepatic, and renal function.
- Patients of childbearing potential or their partners of childbearing potential must be willing to take the appropriate precaution to avoid pregnancy or fathering a child for the duration of Part I and Part II of the study.
- Able to undergo surgery to obtain tumour tissue and lymph nodes for neoantigen evaluation and expansion of T cell populations.
- The area from which the RLNs will be obtained shall not have been exposed to radiotherapy.

##### Exclusion criteria Part I:

- Less than 4 months since a clinically significant cardiovascular event such as myocardial infarction, unstable angina, angioplasty, bypass surgery, stroke, or TIA.
- Congestive heart failure, NYHA class III or IV.
- For patients scheduled for trial specific surgery: In case of suspected impaired lung function, a spirometry is obligatory. Results of less than 65% of the expected value regarding forced expiratory volume in 1 second (FEV1) and/or diffusion capacity is regarded as a criterium for exclusion.
- Any severe acute or chronic medical condition that places the patient at increased risk or interferes with the interpretation of study results.
- Immunodeficiency disorders if present will be discussed with the chief medical officer (CMO) and primary investigator (PI) to judge if they place the patient at increased risk.
- Autoimmunity disorders if present will be discussed with the CMO and PI to judge if they place the patient at increased risk.
- Leptomeningeal metastases (patient with previously treated brain metastases are eligible if there is no evidence of disease progression for a minimum of 8 weeks prior to inclusion).
- Brain metastases necessitating steroid medications for symptoms alleviation.
- Immunosuppressive concomitant medications (steroid medications are allowed if they are used as substitution or are administered topically or as inhalations).
- Previous Grade 3 or greater immune-related toxicity from checkpoint modulation.
- Known acute or chronic infection with hepatitis B or C or syphilis.
- Known HIV infection.
- Pregnant or breastfeeding women.
- Patients with reproductive potential not implementing accepted and effective means of contraception.
- Unable to comply with study procedures.
- For patients required to undergo trial specific surgery to obtain starting material:
  a) No identifiable lymph nodes on pre-surgery radiology accessible, or lymph nodes assessed as unsuitable for surgical excision.
  b) Previous surgical removal of the primary CRC tumour (would entail a high-risk surgery)
  c) Unable to withstand the planned surgery (including ineligibility for general anaesthesia).

**Eligbility criteria Part II: Inclusion Criteria specific to Part II (additional to Inclusion Criteria for Part I stated above and except for criteria specific for surgery and RLNs retrieval)**

- Minimum life expectancy of 3 months (not 6 months which is the requirement for Part I) from the time that the pTTL product is available (as per Investigator’s clinical assessment).
- Have received all possible standard of care therapies, OR further standard of care therapies are currently not considered to be in the patient’s best interest, OR toxicity from previous therapy limits the choice of suitable standard of care therapy OR scheduled pause in palliative standard of care therapy as judged by the Investigator.

**Exclusion Criteria specific to Part II (additional to Exclusion Criteria for Part I stated above and excepting Part I criteria specific for surgery)**

- Less than 6 months (not 4 months which is the requirement for Part I) since a clinically significant cardiovascular event such as myocardial infarction, unstable angina, angioplasty, bypass surgery, stroke, or TIA.
- Less than 4 weeks since last previous systemic cancer treatment.
- Less than 2 weeks since last palliative radiotherapy.
- Less than 4 weeks to start of pre-conditioning after major surgery and less than 3 weeks after minor surgery.
- Participation in any other clinical cancer therapy trial within the previous 4 weeks.
- Less than 4 weeks prior to start of pre-conditioning since administration of live attenuated vaccines.

### Follow-up

A 26-week follow-up period is scheduled after pTTL administration. The follow-up time (26 weeks/6 months) is based on that the vast majority of treatment-related adverse events are expected to occur within the first six months. Long-term follow-up for up to 5 years is in accordance with requirements from the Swedish Medical Products Agency to enable assessment of long-term safety and survival outcomes.

#### Assessment details.

- Safety blood tests will be done prior to pTTL administration, daily during the first week after administration, and on follow-up visits.
- Follow-up visits will be scheduled weekly for the first 6 weeks and then every 4^th^ week.
- Medical Imaging CT and/or MRI will be performed at baseline, weeks 6, 12, 18 and 24.
- Blood for ctDNA will be drawn at pTTL administration and at approximately 6-week intervals for the first 4 months. Thereafter, continued collection of samples is performed if judged of interest.
- Tumour markers, such as carcinoembryonic antigen (CEA), cancer antigen 19−9, and cancer antigen 125, if applicable will be drawn within 24 hours of the start of treatment and every 6 weeks thereafter.
- Tumour biopsies will be obtained when considered feasible based on an assessment of procedural risk and patient burden. Biopsies will be performed within 3 weeks prior to treatment initiation and repeated approximately 4 weeks after pTTL infusion.
- Study blood samples for additional biomarker analyses will be drawn at 2 weeks, 6 weeks, and then every 6 weeks for TCR sequencing, collection of blood cells for functional T cell assays and T cell phenotyping and collections of serum for protein analysis.
- Study samples will also be collected and preserved for future explorative analyses.

### Data management

All available relevant data are in the manuscript and/or supporting information files. Clinical data will be captured using a 21 CFR Part 11–compliant web-based electronic case report form (eCRF; Viedoc™, Viedoc Technologies, Uppsala, Sweden) with password protection and built-in validation checks. Authorised site personnel designated by the Investigator will enter data from source documents. Data quality will be ensured through programmed edit checks, regular monitoring, and query resolution. All data entries and modifications will be recorded in a protected audit trail. Adverse events and medical history will be coded using the Medical Dictionary of Regulatory Activities (MedDRA), and prior and concomitant medications using the WHO Anatomical Therapeutic Chemical classification. The database will be locked after all discrepancies have been resolved and before analysis.

### Statistical analysis

#### General.

Continuous data will be presented in terms of evaluable and missing observations, arithmetic mean, standard deviation, median, minimum and maximum value. Categorical data will be presented as counts and percentages. When applicable, summary data will be presented by treatment, and by assessment time. Individual patient data will be listed by patient number, treatment, and, where applicable, by assessment time. All descriptive summaries and statistical analyses will be performed using SAS Version 9.4 or later (SAS Institute, Inc., Cary, NC). Baseline will be defined as the visit with last data collection point prior to the administration of pTTL. No imputation of missing data will be performed.

#### Determination of sample size.

No formal sample size calculation has been performed for this trial. The proposed sample size is considered sufficient for evaluation of the trial endpoints.

#### Analysis data sets.

The Safety analysis set will include all participants who receive a pTTL infusion, while secondary and exploratory endpoints will be analysed for participants with evaluable data for the respective endpoint.

#### Description of trial population.

- Descriptive statistics for demographics, weight and height will be presented.
- Medical/surgical history will be presented by system organ class (SOC) and preferred term (PT). Prior/concomitant medications will be presented by Anatomical Therapeutic Chemical (ATC) level 3 and 5.
- The patients treated and their individual dose will be listed.

#### Analysis of primary endpoints.

- An overview of AEs, including serious adverse events (SAEs), relationship to pTTL, and deaths will be presented. AESI and related AEs will be summarized separately if considered relevant.
- Clinically significant and non-clinically significant abnormal findings will be specified and summarised. Changes over time will be presented using shift tables, if considered appropriate.
- Vital signs, 12-leads ECG, and safety laboratory analyses clinically significant values will be summarised separately, if considered appropriate.
- All data will be listed by patient.

#### Analysis of secondary endpoints.

Response rate, time to progression, overall survival, progression-free survival and disease specific survival, will be presented with descriptive statistics. All “time-to-event” variables will be descriptively summarised and displayed in, e.g., Kaplan- Meier plots.

#### Exploratory analyses.

pTTL persistence, anatomical location post infusion and capacity for neoantigen recognition will be assessed and presented but may be reported separately from the clinical trial report.

#### Data presentation.

All collected data will be presented by patient, and, when applicable, by assessment timepoint. Categorical data will be presented as counts and percentages. Continuous data will be presented in terms of evaluable observations, arithmetic mean, SD, median, minimum and maximum value.

### Ethical considerations

The study is authorized under the Clinical Trial Regulation (Regulation EU No 536/2014), EU CT #2024-512296-13-00. It is conducted in accordance with the Declaration of Helsinki and the guidelines of the European Commission on Good Clinical Practice (GCP) specific to Advanced Therapy Medicinal Products [36–38]. Subjects ≥18 years of age are eligible for participation in the study and their informed concent is obtained prior to trial inclusion. The paricipants do not receive any financial compensation, and any harm incurred will be covered by regular patient insurance. The study results will be published in peer-reviewed scientific journals.

### Trial status

The study opened 2023-03-15, and patient recruitment is ongoing. Patient recruitment is estimated to be completed in December 2027. Study completion, when the last included study subject have completed Part III, is estimated in December 2032. After study completion, results will be analysed and published.

## Discussion

pTTL therapy relies on an individualized approach, where T cell clones derived from the patient’s tumour-draining lymph nodes are selectively activated and expanded towards up to 36 personal tumour neoantigens. The personalized nature of this ACT has the potential to enhance the precision and efficacy of the immune response against tumour cells. Neoantigens, being unique to cancer cells and absent in normal tissues, present an ideal target for immune-based therapies [29]. By focusing on neoantigens, this therapy aims to reduce the risk of off-target toxicity and improve the efficacy and specificity of the anti-tumour response. Another benefit of targeting neoantigens is that pTTL can be applicable, in theory, in any cancer type where neoantigen-encoding mutations have occurred and where RLNs are accessible for surgical resection to serve as a source of T cells for pTTL production.

The use of RLNs instead of tumour-infiltrating T cells to generate pTTL is driven by the observation that these T cells are less influenced by the immunosuppressive tumour microenvironment (TME) than TILs [22,39]. Due to TME exposure, TILs usually express inhibitory molecules like PD-1 (programmed death-1) and CTLA-4 (Cytotoxic T-lymphocyte antigen 4) which are associated with reduced activity and which could also indicate limited functional capacity of the T cells within the tumour [24,40]. pTTL is a T cell product, displaying individually varying CD4/CD8 ratios and minor proportions of other cells, mainly NK cells. It contains a significant proportion of memory T cells with limited percentages of terminally differentiated T cells. (unpublished data)

The findings from this study will primarily provide insights into the safety profile of pTTL therapy while also investigating early indications of efficacy. Previous studies on adoptive cell therapies, including TIL therapy and CAR-T cell therapy, have demonstrated varying degrees of toxicity, often necessitating close monitoring and supportive care measures. For TIL therapy, a large part of toxicity can be attributed to the pre-conditioning and to post-infusion administration of interleukin 2 (IL-2) [18,41]. In the NEOGAP-CRC-01 trial, we will use cyclophosphamide and fludarabine at lower doses than for standard TIL therapy, and no IL-2 will be administered, reducing the risk of side effects. [42–44]

The option to obtain RLNs for pTTL production through study-specific surgery is an approach that is required to allow access to starting material in patients not eligible for standard-of-care surgery. The overall risk profile of pTTL therapy in this trial is therefore also characterized by study-specific procedures in Part I that patients would not be subjected to outside of the trial. Surgical risks include bleeding, infection, adhesions and bowel obstruction, and, when colon resection is performed, leakage from anastomosis or bowel incision sites. The risks are, however, considered justified by the unmet medical need of eligible patients lacking viable treatment options [45]. A long-term aim for pTTL therapy is to obtain RLNs and tumour tissue for sequencing during standard-of-care CRC surgery only, removing additional risks caused by study-specific surgery and incorporating pTTL procedures seamlessly into routine care.

This study’s primary aim is to show safety and feasibility for the application of pTTL as a novel ACT. It also represents an endeavour to determine the potential of pTTL while simultaneously addressing the limitations of current treatment options for advanced-stage CRC. Adoptive T cell therapy has so far had relatively limited success in CRC. For example, TIL therapy has been applied with modest results [46,47]. Encouraging data have, however, lately emerged. Objective responses have been described to therapies that target neoantigens in a similar manner as for the pTTL concept but use TILs as starting material, shown in clinical trials, including the phase 2 trial of TILs by Lowery et al [28], and two phase 1 trials of the cell product TBio-4101 (NCT05576077 and NCT05628883, Turnstone Biologics, discontinued) [48]. Support for the concept of T cell therapy selectively targeting neoantigens was seen in the phase 2 study when comparing unselected TILs to selected neoantigen-specific TILs in patients with gastrointestinal cancers. In that trial treatment response was seen only in patients treated with TIL products selected for neoantigen reactivity (in 7.7% of the patients) and the objective response rate was further enhanced when combining selected TILs with pembrolizumab (to 23.5% of the patients) [28]. TBio-4101 was investigated in multiple cancer indications, including CRC. Results for the CRC patients demonstrated a complete response (ongoing 48 weeks after treatment) in one of four treated patients, further indicating the potential of neoantigen-targeting T cells in this patient group [48].

Immunotherapy with checkpoint inhibition has been investigated with mixed results in CRC patients: Mismatch repair (MMR)-deficient tumours showed significant responses, while the effect in MMR-proficient tumours was limited [49–51]. This highlights that there are significant differences in immunological activity between tumour subgroups, indicating that sensitivity to T cell therapy could vary. However, it has also been noted that the relative degree of immune infiltration is correlated to prognosis across different CRC subtypes [6,7,52]. Thus, there is a rationale for adoptive T cell therapy in the entire CRC group, as improved infiltration by activated T cells could be hypothesised to be beneficial across subgroups, supporting the choice of CRC as initial target indication for pTTL.The results from this first-in-human trial will lay the groundwork for subsequent clinical studies, potentially leading to larger, randomized trials to further evaluate the efficacy and safety of pTTL therapy. Future studies could explore the combination of pTTL with other therapies. For example, checkpoint inhibitors could be used with pTTL to enhance anti-tumour responses and overcome immune resistance mechanisms. Additionally, pTTL may be investigated in other cancer types with surgically available RLNs, broadening the potential impact of this personalized therapy.

## Supporting information

S1 Checklist SPIRIT 2025 checklist. The checklist indicates where each recommended protocol item is addressed in the present study protocol.
(DOCX)

S2 File Clinical study protocol. The full current version of the clinical study protocol with masking of personal information regarding investigators and staff within the trial.
(PDF)
