# Supplementary material for: A first-in-human, phase I/IIa trial of the T cell immunotherapy personalised tumour trained lymphocytes in patients with advanced colorectal cancer: Study protocol
Source: PLoS One. 2026 Sep 18;21(9):e0351697. doi: 10.1371/journal.pone.0351697 (PMC13588363; doi:10.1371/journal.pone.0351697)
Supplement: Supplementary file 1 [file pone.0351697.s002.pdf]

**Clinical Trial Protocol**	
EU Trial No	2024-512296-13-00
Investigational medicinal product	pTTL
Trial code	NEOGAP-CRC-01
Protocol version and date	Final version 11.0, 30APR2025

A First-In-Human, Phase I/IIa Trial of the novel T cell Immunotherapy pTTL in Patients with Advanced Colorectal Cancer

**Phase**	I/IIa, first-in-human (FIH)
**Indication**	Patients with stage IV colorectal cancer (CRC)
**Test product**	Personalised Tumour Trained Lymphocytes (pTTL)
**Sponsor signatories**	[redacted], CEO<br>[redacted], Sponsor's Medical Representative<br><br>NEOGAP Therapeutics AB<br>Cancer Center Karolinska<br>Visionsgatan56<br>SE-171 64 Solna, Sweden
**Coordinating Investigator**	[redacted], MD, PhD<br><br>Phase 1 unit, Karolinska University Hospital<br>Solna, Stockholm, Sweden
**Clinical trial management**	CTC Clinical Trial Consultants AB<br><br>Dag Hammarskjölds väg 10B<br>SE-752 37 Uppsala, Sweden

CONFIDENTIALITY STATEMENT

All material, information (oral or written) and unpublished documentation including the present protocol and the eCRF, are exclusive property of the Sponsor, NEOGAP Therapeutics AB. These materials or information cannot be given or disclosed to any other unauthorised person without the prior formal written consent of the Sponsor.

Amendments made to the Clinical Trial Protocol are summarised below.

Type of change	Revised protocol version	Summary of changes
Initial Submission<br>EC approval: 20APR2022<br>CA approval: Not approved	Version 1.0 25MAR2022 (original version)	Not applicable
Answer to CA<br>EC approval: Not applicable<br>CA approval: Not approved	Version 2.0 06JUN2022	Revised based on CA objections/comments
Substantial Amendment No1 02NOV2022<br>EC approval: 30NOV2022<br>CA approval: Not applicable	Version 3.0, 28OCT2022	Changes in trial design and definition of site as well as requirements for sufficiently high quality of collected tumour biopsies.
Resubmission<br>EC approval: Not applicable<br>CA approval: Conditional approval 31JAN2023	Version 4.0 13JAN2023	Resubmission to CA
Substantial amendment No2 20FEB2023<br>EC approval: 08MAR2023<br>CA approval: Not applicable	Version 5.0, 10FEB2023	Addition of serology testing prior to inclusion in Part II.
		Prolongation of screening period for Part II. Previous: Day -21 to Day -10, New: Day -28 to Day -10.
		Clarification of pre-conditioning procedures
		Clarification of timing of vital signs assessments in association with pTTL infusion.
		Administrative changes and clarifications
Substantial amendment No3	Version 6.0, 29MAY2023	Change of exclusion criterion No 2 and 3 for Part I and Part II:<br>• No 2. Cardiac function assessment has been changed from being based on a clinical assessment of symptoms according to the NYHA classification to instead be based on left ventricle ejection fraction and the absence of significant valvular disease. These measurements are obtained by echocardiogram. This results in the addition of mandatory echocardiogram prior to inclusion in Part I for patients scheduled for trial specific surgery and prior to inclusion in Part II for all patients. Echocardiogram for patients scheduled for standard surgery is not mandatory prior to inclusion in Part I due to time limitations prior to standard surgery.<br>• No.3: Clarification that spirometry is not mandatory prior to inclusion in Part I for patients scheduled for standard surgery, even if the patient is considered to require assessment by

Type of change	Revised protocol version	Summary of changes
		spirometry before pTTL therapy. This was changes in order to facilitate inclusion of patients undergoing standard surgery were timelines can be very short. The spirometry will then be done as soon as considered relevant, and latest before inclusion in part 2
		Addition of informed consent procedure for pre-screening in Part I
		Clarifications of requirements of timing/stop of previous systemic cancer treatment, radiotherapy, major/minor surgery, participation in other clinical trial and administration of live attenuated vaccines (2 to 4 weeks prior to start of pre-conditioning).
		Correction of timing of pTTL administration after completion of product formulation from 2 hours after completion to 4 hours after completion.
		Change of Sponsor's chief medical officer (CMO) from [redacted] to [redacted]
		Administrative changes and clarifications
Substantial amendment No4	Version 7.0 19DEC2023	Change in pTTL drug substance formulation, introducing cryopreservation after production. The product will be thawed just before administration rather than being administered fresh.
		In consequence of this change, the planned treatment day of pTTL will always be Day 1 in relation to timing of pre-conditioning, which will take place between Day -5 and Day -3. In protocol version 6.0, pTTL could be administered either on Day -3 or Day -1 (pre-conditioning between Day -7 and Day -5) depending on the time needed for pTTL production. Supportive care will be given from Day -7 (previously from Day -9).
		Change of the accepted time between collection of starting material and administration of pTTL from 6 to 12 months
		Change in specification of allowed deviation range for planned doses. Previous deviation allowed: down to -5%. New deviation allowed: ±5%.
		At the screening visit (Visit 1, Part I): acceptance of use of safety laboratory test results (ordered for other purpose) obtained within 1 week of screening at the discretion of the Investigator.
		Administrative changes due to e.g. previous typos or inconsistencies.
Substantial amendment No5	Version 8.0 27MAR2024	Change of allowed surgical methods for trial-specific surgery: resection of a section of colon or rectum is included among the surgical methodologies for trial-specific surgeries.
		Adjustment of the number of regional lymph nodes (RLNs) retrieved by extirpation in association with the bowel segment resection to only be restricted by the judgement of the Investigator (no change of the up to 6 RLNs retrieved at trial-specific minimally invasive [laparoscopic] lymphadenectomy procedure).
		Addition of withdrawal criteria: patient's surgery/biopsy material does not meet the requirements for pTTL production.
		Change of criteria on radiological detection of RLNs before study specific surgery: not specified by number but by the judgement of radiologists, surgeons (study Investigator/Co-investigator) and oncologist (Coordinating PI).

Type of change	Revised protocol version	Summary of changes
		Addition of Investigator [redacted] and of the Medical Unit Pelvic Cancer at the Recruitment, Surgery & Treatment site (previously Recruitment & Treatment site).<br>Change of Principal Investigator at Recruitment & Surgery site 1 from [redacted] to [redacted].<br>Addition of Recruitment & Surgery site 2: Danderyd hospital AB, with [redacted] as Principal Investigator.<br>Administrative changes due to e.g. previous typos or inconsistencies.
CA request based on Substantial amendment No5	Version 9.0 20JUN2024	Addition of requirement for pre-surgery meeting in cases where bowel resection is considered. The internal safety review committee (iSRC), supplemented by an independent medical expert and expert surgeon(s), will assess if bowel resection is considered acceptable from an individual risk/benefit perspective. A recommendation for the procedure from the committee is required before the surgery may be performed.
Non-substantial modification	Version 9.1 03OCT2024	Change of the accepted time between collection of starting material and administration of pTTL from 12 months to approximately 19 months, by the addition of the storage time for the cryopreserved pTTL product.<br>Administrative changes and clarifications.
Substantial amendment No6 (SM-2 in CTIS)	Version 10.0 06MAR2025	The dose escalation strategy based on increasing dose levels and a modified 3+3 Simon's design is exchanged for a strategy allowing free dosing within the range encompassed by the pTTL product specification, $20x10^6 – 10^9$ cells. The iSRC will review accumulating safety data after each of the first 3 dosed patients and then quarterly instead of reviewing data after dosing of separate cohorts with a set number of patients. The stopping criteria have been updated to align with these changes. Reflected mainly in Section 8.4 "Selection of doses and rationale for dosing strategy"<br>Change of the accepted time between collection of starting material and administration of pTTL from 19 months to approximately 24 months, due to prolonged shelf life for cryopreserved RLN starting material and pTTL products equalling ≥ 2 years. Reflected in e.g. Section 8.1.1 "Part I: collection of tumour samples or next generation sequencing and starting material for pTTL manufacturing".<br>Clarification of procedures in case of tumour progression. Reflected in e.g. Section 8.1.3 "Part III: long-term follow-up".<br>Simplification and increased flexibility in the instructions for supportive care treatments for the pre-conditioning, Section 11.2.4 "Pre-conditioning and supportive care".<br>Updates to Section 6.4 "Clinical experience" and Section 6.5 "Trial rationale" based on recent data.<br>Minor update to Section 6.6.2.2 regarding risks for unwanted immunity – extended discussion on risks associated with debris and Section 6.6.2.5 "Toxicity and off-target reactions" regarding the potential impurity DNAse. No change in risk-benefit balance.<br>Clarification of withdrawal criteria in Section 9.1.1 "General withdrawal criteria".

Type of change	Revised protocol version	Summary of changes
		Clarifications of rescreening criteria in Section 9.8 “Screening failures”.<br>Adjustment of the number of RLNs that may be collected during trial specific surgery in Section 11.2.3 “Collection of material from regional lymph nodes for pTTL production (Part I)”.<br>Minor revision of Section 11.1.7 “Prior and concomitant medications” to simplify the instructions for registration in the eCRF.<br>Change of Sponsors Medical Representative from [redacted] to [redacted]<br>Administrative changes due to e.g. previous typos or inconsistencies.
Response to Substantial amendment No 7 (SM-2 in CTIS)	Version 11.0 30APR2025	Removal of risk assessment discussion on DNase in section 6.6.2.5 “Toxicity and of target-reactions”.<br>Section 14.1: Adjusted from Clinical Trials Directive to Clinical Trials Regulation (EU) 536/2014

1 TRIAL SYNOPSIS

<table>
<tr><td colspan="2">Trial title
A First-In-Human, Phase I/IIa Trial of the novel T cell Immunotherapy pTTL in Patients with Advanced Colorectal Cancer</td></tr>
<tr><td colspan="2">Trial advanced therapy investigational medicinal product
Personalised Tumour Trained Lymphocytes, pTTL</td></tr>
<tr><td>Trial code
NEOGAP-CRC-01</td><td>EudraCT No
2022-000394-96</td></tr>
<tr><td>Planned trial period
Part I and Part II: Q1 2023 to Q2/Q3 2026
Part III: until approximately Q4 2030</td><td>Phase of development
First-in-human (FIH)/Phase I/IIa</td></tr>
<tr><td colspan="2"><u>Recruitment, Surgery & Treatment Site</u>
Phase 1 unit, Center for Clinical Cancer Studies, Theme Cancer, Karolinska University Hospital Solna, Stockholm, Sweden
Coordinating Investigator/Principal Investigator: [redacted], MD, PhD
Medical Unit Cell therapy and Allogeneic stem cell transplantation (ME CAST), Theme Cancer, Karolinska University Hospital Huddinge, Stockholm, Sweden
Investigator: [redacted], MD, PhD, Associate Professor
Medical Unit Pelvic cancer - Colorectal cancer, Theme Cancer, Karolinska University Hospital Solna, Stockholm, Sweden
Investigator: [redacted], MD PhD, Associate Professor
Part I: patient screening of all referred patients, surgery and biopsy (collection of tumour material)
Part II: patient screening, administration of pre-conditioning therapy and the advanced therapy investigational medicinal product (ATIMP): personalised Tumour Trained Lymphocytes (pTTL), follow-up of patients after pTTL administration
Part III: follow-up
<u>Recruitment & Surgery Site 1</u>
Department of Surgery, Västmanlands hospital, Västerås, Sweden
Principal Investigator: [redacted], MD, PhD, Associate Professor
Part I: patient screening (local patients), surgery and biopsy (collection of tumour material)
<u>Recruitment & Surgery Site 2</u>
Department of Surgery and Urology, Danderyd's Hospital AB
Principal Investigator: [redacted], MD PhD, Professor
Part I: patient screening (local patients), surgery and biopsy (collection of tumour material)</td></tr>
<tr><td colspan="2">Trial design
This is an open-label, non-randomised FIH trial investigating the safety and tolerability of a novel ATIMP, pTTL, composed of autologous tumour-draining lymph node-derived T cells stimulated in vitro with personalised cancer neoantigens.
The neoantigens are selected through a process starting with next generation sequencing (NGS) of tumour material from the patient followed by ranking of detected neoantigenic mutations using the bioinformatic software system PIOR® Manufacturing, developed by the Sponsor. Selected neoantigen epitopes are expressed as recombinant proteins, NAG, and coupled to paramagnetic beads to form TC0301, the T cell-stimulating raw material used to promote neoantigen-specific T cell expansion in</td></tr>
</table>

vitro in pTTL production. The production process also includes stimulation with interleukin 2 (IL-2). pTTL is thus based on autologous cells stimulated with patient-specific neoantigens, and every pTTL product is unique and designated for use in one single individual.

pTTL will be administered to patients with stage IV colorectal cancer (CRC) as a single intravenous dose. The maximum dose to be administered is 10^9 viable cells.

Objectives

Primary objective

To establish that pTTL can be administered to patients with advanced CRC without unacceptable toxicity

Secondary objectives

- To evaluate treatment efficacy in terms of objective response.
- To evaluate treatment efficacy in terms of progression free survival and overall survival.

Exploratory objectives

- To explore biomarkers for:
  - pTTL persistence *in vivo*.
  - anatomical localisation of pTTL post infusion, with focus on tumour infiltration.
  - pTTL recognition of neoantigens *in vivo* and for pTTL capacity to achieve tumour cell eradication
  - pTTL characteristics
- To gain early insights in selection of neoantigens. Data on the association between the selected neoantigens, biomarker evaluation and clinical outcome will be collected.

Endpoints

Primary endpoint

The primary endpoint is safety with adverse events (AEs) assessed according to Common terminology criteria for AEs (CTCAE) v5.0. Special focus will be placed on immunological AEs and AEs known to be associated with T cell therapies, which are classified as AEs of special interest (AESI). AESI will include autoimmune reactions potentially resulting from off-target toxicity such as colitis, and immune-mediated reactions associated with immune cell activation, such as cytokine release syndrome (CRS).

Secondary endpoints

- Objective response (according to iRECIST).
- Characterisation of treatment response:
  - time to response
  - duration of response
- Time to tumour progression assessed using the iRECIST criteria (time from inclusion in Part II of the trial until disease progression).
- Kinetics of tumour progression/growth (compared to pre-treatment)
- Overall survival (time from inclusion in Part II to death of any cause).
- Progression-free survival (time from inclusion in Part II to tumour progression or death of any cause).
- Disease-specific survival (time from inclusion in Part II to death of disease).

<u>Exploratory endpoints</u>

- Evaluation of biomarkers for:
    - pTTL persistence. T cell receptor sequencing (TCRseq) will be used to characterise the pTTL product and to trace the identified pTTL clones in sequential peripheral blood samples post treatment.
    - pTTL tumour infiltration. If feasible, pre- and post-treatment biopsies will be taken for histological assessment of T cell infiltration and characterisation of tumour-infiltrating T cells.
    - pTTL neoantigen specificity and efficacy:
        - Functional T cell assays will be used to evaluate neoantigen-specific responses in the pTTL product and in peripheral blood T cells.
        - Markers of tumour burden will be evaluated.
    - pTTL characteristics: analysis of the pTTL product and of post therapy peripheral blood samples will be performed to evaluate the functional status, differentiation and lineage-identity of T cells and other immune cells.
- Storage of data relevant for the correlation of neoantigen selection to clinical outcome. The type of neoantigens targeted will be assessed in the context of clinical outcomes, signals of efficacy and biomarker evaluation.

Exploratory endpoints may be reported separately from the clinical trial report.

Number of patients planned

No formal inclusion limit is set for Part I of the trial in order to accommodate to the possibility that some of the included patients will be unable to continue to Part II due to e.g. disease progression.

Up to 16 patients will be included in Part II with the aim to achieve at least 12 evaluable patients treated with pTTL.

An evaluable patient is defined as a patient who has received treatment with pTTL (fulfilling the specification) and for whom at least 6 weeks follow-up data are available (unless death occurred).

Diagnosis and main eligibility criteria

The selected target group of the trial is adults with stage IV CRC. Patients may be considered eligible for inclusion and treatment if they have received treatments according to standard of care or if further standard of care treatment options are judged not to be in the patients' best interest (e.g. due to toxicity issues). Patients could also be eligible for inclusion in the trial during a scheduled pause in ongoing palliative therapy, at the discretion of the treating Investigator. Eligible patients must have a primary or metastatic lesion that is accessible to biopsy or surgery to obtain tumour material for NGS analysis. In addition, it is a requirement that the patient can undergo surgical excision of regional lymph nodes (RLNs) for use in pTTL production. Eligible patients should also have a good performance status (ECOG 0-1) and organ function, and no pre-existing conditionings deemed to increase their risk for severe side effects of pTTL therapy.

Materials for Part I of the trial can be collected, while non-curative therapeutic options remain, and cryopreserved for later use if the patient should become eligible for inclusion in Part II.

Purpose and rationale

Adoptive T cell therapies (ACT) have been shown to be successful in selected patients with advanced malignant disease. They have the potential for major and long-lasting efficacy as they rely on the immune system's capacity to first eradicate cancer cells and to then develop tumour-specific immunological memory, preventing cancer recurrence.

pTTL consists of autologous T cells activated and expanded *in vitro* using tumour-specific personalised neoantigens. The scientific rationale for pTTL relies on the fact that somatic mutations in tumour cells arising during malignant transformation result in altered proteins or peptides, so called

neoantigens. Each individual tumour presents a unique mutation profile. The neoantigens distinguish tumour cells from normal cells and make the tumour cells targetable to be eradicated by the immune system through a mechanism termed cancer immunosurveillance. Generally, each tumour harbours multiple mutations that form a unique pattern, which enables personalisation of each therapeutic product and also allows targeting of several neoantigens simultaneously. In the present trial, up to 36 neoantigens will be targeted in each pTTL product.

The choice of starting material, tumour-draining RLNs, is based on several advantages: The exposure to tumour antigens and tumour cells causes an enrichment of tumour-specific T cells in RLNs. These T cells also have the potential to be superior to tumour-infiltrating T cells due to less exposure to tumour-mediated immunosuppression.

The production process for pTTL includes *in vitro* expansion, which increases the neoantigen-reactive T cell population. The selective T cell expansion is achieved by stimulation with NAG-coupled beads bearing neoantigen epitopes, based on sequencing data from each patient's own tumour. This improves the chance of effective cancer cell eradication by both increasing the number of cells able to recognise and respond to the tumour, and by breaking immunological tolerance created *in vivo* by immunosuppressive mechanisms through activation in a non-suppressive environment.

pTTL will be combined with a pre-conditioning treatment with chemotherapy on 3 consecutive days prior to infusion.

In conclusion, the present trial combines harvest of enriched tumour-specific T cells from RLNs and neoantigen technology with the aim of producing an effective personalised immunotherapy applicable to any solid cancer with targetable neoantigens. The target indication for this FIH Phase I/IIa trial is advanced (stage IV) CRC.

Methodology

The trial is composed of 3 parts, referred to as Part I, Part II and Part III.

Part I of the trial entails patient identification, recruitment and screening of trial patients. It also comprises production of pTTL, which is performed in 3 steps:

1. Collection of tumour samples and normal samples for NGS and neoantigen selection. Production of stimulatory NAG-coupled beads raw material TC0301.
2. Collection of starting material for pTTL manufacturing from RLN.
3. pTTL manufacturing.

The time required from collection of tumour material and RLNs up until pTTL production and administration to the patient is approximately 8 to 12 weeks. The RLN starting material is cryopreserved after initial processing, enabling flexibility in the timing of pTTL administration. For this reason, potential trial patients will be consented separately for Part I and for Part II/III. A first consent is given for pre-screening activities in Part I, a second for tissue collection and pTTL production in Part I, and a third consent is given for pTTL treatment, including pre-conditioning chemotherapy and follow-up in Part II/III. The first consents may, in some cases, take place while the patient has other non-curative therapeutic options but might become eligible for inclusion in Part II of the trial within approximately 24 months.

Part I comprises 2 visits. Visit 1 (patient identification, recruitment and screening of trial patients) will be performed at one of the Recruitment & Surgery Sites or at the Recruitment, Surgery & Treatment Site. Potential trial patients will also be identified at the Referral Sites. Visit 2, surgery for collection of patient materials for pTTL manufacturing, will be performed at the Recruitment, Surgery & Treatment Site or at one of the Recruitment & Surgery Sites. Tumour biopsies can be obtained at the Recruitment & Surgery Sites and at the Recruitment, Surgery & Treatment Site.

Part II of the trial is performed at the Recruitment, Surgery & Treatment Site and entails pre-conditioning and supportive care therapy, treatment with pTTL, and 26 weeks safety follow-up. pTTL will be administered as a single intravenous dose. For the first three patients in the trial, at least

28 days safety and tolerability data must have been reviewed by the internal safety review committee (iSRC) prior to dosing of the next consecutive patient. From the fourth patient and throughout the trial, patients will be dosed one by one, at least 14 days apart. iSRC meetings will be held quarterly after the meeting following pTTL administration to patient 3.

Part II comprises 18 visits (Visit 3 to Visit 20) over 26 weeks. Visit 4 encompasses Day -7 to Day -1. Supportive care treatment will start on Day -7 or Day -6 and will continue according to schedule with discontinuation of antimicrobial prophylaxis when immune function is judged to be restored. Pre-conditioning will be administered for 3 days, starting on Day -5 and finishing on Day -3. Exceptions to this schedule are allowed and the planned pTTL administration day may be postponed (but will always be referred to as Day 1) as judged by the Investigator. The 3-day preconditioning should, however, be finalised at least 48 hours prior to pTTL administration, and not earlier than 120 hours prior to pTTL administration. The supportive care treatment may be given in an out-patient setting while the 3 days pre-conditioning may be performed in an in-patient or out-patient setting as judged by the Investigator and in line with the prevailing Covid-19 policy at the Recruitment, Surgery & Treatment Site. Patients will be admitted to ME CAST on Day 1 for pTTL treatment and will stay for observation for at least 7 days after pTTL administration. Follow-up assessments performed over the subsequent 26 weeks include safety assessments, biomarker sampling, medical imaging (and evaluation) and an optional tumour biopsy.

Part II will end when the last treated patient has been followed for 26 weeks or earlier in case of death or patient withdrawal.

Part III of the trial is a long-term follow-up initiated after completion of the initial 26 weeks safety follow-up period. Patients will participate in Part III for up to 4.5 years after end of Part II or until death, whichever comes first. Patients entering Part III will be followed-up in terms of safety, disease progression, survival and pTTL persistence (biomarker analysis).

The estimated recruitment time to complete inclusion of trial patients is 24 months. The total estimated time to complete Part I and Part II of the trial is 36 months, including active recruitment and pTTL treatment during approximately 30 months and 6 months follow-up of the last included patient.

Advanced therapy investigational medicinal product, dosage and mode of administration

The drug product, i.e. pTTL, is composed of *in vitro* expanded RLN cells, enriched for neoantigen specific T cells. It is formulated as a cell suspension, 1-10 x10^6 viable cells per mL in Ringer-Acetate and human serum albumin (25 mg/mL) mixed volume/volume with the cryopreservation medium Cryostor® CS10 in a transfusion bag intended for intravenous transfusion. The product is cryopreserved after production and thawed just before administration.

pTTL is unique for each patient as the neoantigen combination is unique for each individual tumour and due to the use of autologous node-derived T cells.

The cell dose obtained during the manufacturing process will vary depending on the size of the neoantigen-reactive clones present in the starting material and their proliferative capacity. The minimum dose of a pTTL unit is 20x10^6 viable cells and the maximum dose is 10^9 viable cells.

Non-investigational medicinal products (NIMPs)

Pre-conditioning therapy will be administered to all patients prior to treatment with pTTL. The pre-conditioning therapy includes 3 days intravenous infusion of cyclophosphamide 300 mg/m^2 BSA and fludarabine 30 mg/m^2 BSA. The pre-conditioning therapy infusion must be completed at least 48 hours before pTTL infusion to prevent negative chemotherapy effects on the infused pTTL product.

All patients will also receive supportive care therapy in association with pTTL administration (over Day -7/Day -6 to Day 8 in accordance with standard of care. The supportive care therapy includes

antiemetic therapy, antimicrobial prophylaxis, prophylactic treatment against tumour lysis syndrome, iv hydration, and optional preventive therapy against urothelial toxicity from cyclophosphamide.

Duration of treatment

Each patient will receive a single administration of pTTL.

pTTL should be administered in as short time as possible. The infusion rate, and thus the infusion time, is decided upon by the Investigator present at the pTTL administration, taking into account the volume of the product and any relevant underlying disease such as congestive heart failure or renal failure. The total infusion time depends on the volume of the pTTL product, and will vary, as volumes can range from 10 to 100 mL. The above, along with altered infusion rates or intermittent pauses due to reactions in response to pTTL infusion may affect the overall infusion time, however, the maximum estimated infusion time is 1 hour.

Duration of each patient's involvement in the trial

The minimum duration of participation in Part I (for patients proceeding directly to Part II) range between 9 and 15 weeks (the minimum time will vary according to patient-related and trial-related circumstances, and includes the time needed for collection of tumour material and RLNs, and for pTTL production). The longest allowed duration of participation in Part I is 24 months after collection of RLNs.

The total duration of each patient's involvement in Part II is estimated to approximately 29 weeks including a 14-day screening period, 7 days pre-administration supportive care therapy (of which 3 days includes pre-conditioning) and 26 weeks safety follow-up after pTTL administration.

The total duration of each patient's involvement in Part III will be 4.5 years or until death, whichever comes first.

Patients will be urged to contact the Recruitment, Surgery & Treatment Site in between visits, including long-term follow-up visits, in case of e.g. development of any new/recurrent cancer, development of infection or immunogenicity related reactions. If considered relevant by the Investigator, extra visits will be scheduled.

Safety assessments

AEs, weight, physical examinations, vital signs, electrocardiogram (ECG), blood sampling for analysis of safety laboratory parameters.

Preliminary efficacy assessments

Medical imaging and response per iRECIST, disease progression, survival.

Exploratory assessments

Blood and tumour sampling for biomarker analysis.

Statistical methods

No formal sample size calculation has been performed for this trial. The proposed sample size is considered sufficient for evaluation of the trial endpoints.

Descriptive statistics will be applied. All descriptive summaries and statistical analyses will be performed using SAS Version 9.4 or later (SAS Institute, Inc., Cary, NC).

Trial reporting

After completion of Part II, an International Council for Harmonisation of Technical Requirements for Pharmaceuticals for Human Use (ICH) E3 compliant clinical trial report will be prepared. Long-term follow-up data from Part III will be reported in an addendum to the clinical trial report. Data from all parts will also be reported in yearly development safety update reports (DSURs).

2 TABLE OF CONTENTS

1 TRIAL SYNOPSIS ... 6

2 TABLE OF CONTENTS ... 12

3 LIST OF ABBREVIATIONS AND DEFINITIONS OF TERMS ... 18
3.1 List of abbreviations ... 18
3.2 List of trial definitions ... 22

4 IMPORTANT MEDICAL PROCEDURES TO BE FOLLOWED BY THE INVESTIGATOR ... 23
4.1 Medical emergencies contacts ... 23

5 INVESTIGATORS AND TRIAL ADMINISTRATIVE STRUCTURE ... 24
5.1 Trial administrative structure ... 24
5.2 Trial conduct ... 25

6 INTRODUCTION ... 27
6.1 Background ... 27
6.1.1 Cancer Immunotherapy ... 27
6.1.2 Target patient group ... 27
6.2 Product characteristics and mechanism of action ... 28
6.3 Non-clinical development ... 30
6.4 Clinical experience ... 32
6.5 Trial rationale ... 34
6.6 Risk/benefit assessment ... 35
6.6.1 Benefits ... 35
6.6.2 Risks ... 36
6.6.3 Final risk/benefit assessment ... 47

7 TRIAL OBJECTIVES AND ENDPOINTS ... 48

8 TRIAL DESIGN ... 50
8.1 Overall trial design and schedule of events ... 50
8.1.1 Part I: collection of tumour samples for next generation sequencing and starting material for pTTL manufacturing ... 52
8.1.2 Part II: pre-conditioning, supportive care therapy, treatment and 26 weeks safety follow-up 55
8.1.3 Part III: long-term follow-up ... 61
8.2 Assessments at each visit ... 62
8.2.1 Visit 1: Screening Part I ... 62
8.2.2 Visit 2: Surgery (Part I) ... 63
8.2.3 Visit 3: Screening Part II ... 64
8.2.4 Visit 4: Pre-conditioning and supportive care therapy (Part II) ... 64
8.2.5 Visit 5: treatment with pTTL and initial treatment follow-up (Part II) ... 65
8.2.6 Visit 6 to Visit 20: 26 weeks safety follow-up (Part II) ... 66

8.2.7 Visit 21 and onwards: long-term follow-up (Part III) 66

8.3 Rationale for trial design 67

8.4 Selection of doses and rationale for dosing strategy 68

8.4.1 Selection of dose, and rationale for minimum and maximum dose 68

8.4.2 Stopping criteria and trial stopping rules 71

8.4.3 Internal safety review committee 72

9 TRIAL POPULATION 74

9.1 Recruitment 74

9.2 Screening and enrolment log 74

9.2.1 Pre-screening Part I 75

9.2.2 Screening and inclusion Part I 75

9.2.3 Screening and inclusion Part II 76

9.3 Number of patients 76

9.4 Eligibility criteria Part I 77

9.4.1 Inclusion criteria Part I 77

9.4.2 Exclusion criteria Part I 78

9.5 Eligibility criteria Part II 80

9.5.1 Inclusion criteria Part II 80

9.5.2 Exclusion criteria Part II 81

9.6 Prior and concomitant medication 82

9.7 Prior and concomitant procedures 83

9.8 Screening failures 83

9.9 Patient withdrawal 83

9.9.1 General withdrawal criteria 83

9.9.2 Procedures for discontinuation of a patient from the trial 84

9.9.3 Patient replacement 84

10 TREATMENTS 84

10.1 Identity of advanced therapy investigational medicinal products 84

10.2 Identity of non-investigational medicinal products 85

10.3 Manufacturing of the drug substance 85

10.4 Manufacturing of the drug product, packaging, labelling and release 86

10.5 Shipment of pTTL product and conditions for storage 87

10.6 Traceability 87

10.7 Preparation and accountability 87

10.8 Treatment administration and associated procedures 88

10.9 Continuation of treatment with advanced therapy investigational medicinal product 89

10.10 Treatment compliance 89

10.11 Return and destruction of advanced therapy investigational medicinal product 89

11 TRIAL ASSESSMENTS 89

11.1 Recording of data and baseline assessments 89

11.1.1 Informed consent 89

11.1.2 Eligibility criteria ... 89
11.1.3 Demographic information ... 89
11.1.4 Medical/surgical history ... 90
11.1.5 Serology ... 90
11.1.6 Weight and height ... 91
11.1.7 Prior and concomitant medication ... 91
11.1.8 Pregnancy test ... 92
11.1.9 Eastern Cooperative Oncology Group (ECOG) performance status ... 93
11.1.10 Covid-19 PCR test ... 93
11.1.11 Spirometry ... 93
11.1.12 Echocardiogram ... 93
11.2 Trial specific assessments ... 93
11.2.1 (Part I) Collection of tumour samples and normal samples for next generation sequencing 93
11.2.2 I) Collection of blood sample for next generation sequencing (normal control) (Part 94
11.2.3 Collection of material from regional lymph nodes for pTTL production (Part I) 95
11.2.4 Pre-conditioning and supportive care therapy ... 96
11.2.5 Biomarkers ... 100
11.2.6 Medical imaging and iRECIST ... 102
11.3 Safety assessments ... 104
11.3.1 Adverse events ... 104
11.3.2 Physical examination ... 112
11.3.3 Vital signs ... 112
11.3.4 Laboratory safety assessments ... 113
11.3.5 Electrocardiograms ... 114
11.4 Appropriateness of measurements ... 114
12 PROCEDURES FOR BIOLOGICAL SAMPLES ... 114
12.1 Sample collection ... 114
12.2 Volume of blood ... 115
12.3 Handling, storage and destruction of laboratory samples ... 115
12.4 Chain of custody of biological samples ... 116
12.5 Withdrawal of informed consent for donated biological samples ... 116
13 QUALITY MANAGEMENT, QUALITY ASSURANCE AND QUALITY CONTROL ... 116
13.1 Quality management: critical process, system and data identification ... 116
13.2 Quality assurance and quality control ... 117
14 ETHICAL AND REGULATORY REQUIREMENTS ... 117
14.1 Ethical conduct of the trial ... 117

14.2 Ethics and regulatory review ... 117
14.3 Patient information and consent ... 117
14.4 Patient alert card ... 118
14.5 Patient data protection ... 118
14.6 Changes to the approved clinical trial protocol ... 119
14.7 Audits and inspections ... 119
14.8 Insurance ... 119

15 TRIAL MANAGEMENT ... 120
15.1 Training of trial site personnel ... 120
15.2 Clinical monitoring ... 120
15.3 Source data documents ... 121
15.4 Trial agreements ... 121
15.5 Trial time table and end-of-trial ... 121
15.6 Termination of the trial ... 121
15.7 Reporting and publication ... 122
15.7.1 Clinical trial report ... 122
15.7.2 Annual safety report ... 122
15.7.3 Confidentiality and ownership of trial data ... 122
15.7.4 Publication ... 122
15.8 Archiving ... 123

16 DATA MANAGEMENT ... 123
16.1 The web-based eCRF ... 123
16.2 The entering of data into the eCRF ... 124
16.3 The query process ... 124
16.4 Audit trail ... 124
16.5 External data ... 124
16.6 Medical coding ... 124
16.7 Database lock ... 124

17 STATISTICAL METHODS AND DETERMINATION OF SAMPLE SIZE 125
17.1 General ... 125
17.2 Determination of sample size ... 125
17.3 Analysis data sets ... 125
17.3.1 Safety analysis set ... 125
17.4 Description of trial population ... 125
17.4.1 Demographics and baseline characteristics ... 125
17.4.2 Medical/surgical history and prior/concomitant medication ... 125
17.4.3 Treatment compliance ... 125
17.5 Analysis of primary endpoints ... 126
17.5.1 Adverse events ... 126
17.5.2 Physical examination ... 126
17.5.3 Vital signs ... 126
17.5.4 12-lead ECG ... 126
17.5.5 Safety laboratory analyses ... 126

17.6 Analysis of secondary endpoints ... 126
17.6.1 Definitions ... 126
17.6.2 Time-to-event variables ... 127
17.6.3 Tumour response ... 127
17.6.4 Correlation between tumour response and tumour markers ... 127
17.6.5 Exploratory analyses ... 127
18 REFERENCES ... 128
19 SIGNATURES ... 134
19.1 Principal Investigator statement ... 134
19.2 Signature page (approval of the clinical trial protocol) ... 135

List of Tables

Table 1 Medical emergencies contact ... 23
Table 2 Overview of pTTL cell composition and phenotypic characteristics ... 31
Table 3 Objectives and endpoints ... 48
Table 4 Schedule of events Part I ... 53
Table 5 Schedule of events Part II: screening, pre-conditioning, pTTL administration and initial treatment follow-up ... 57
Table 6 Detailed schedule of events Visit 4: supportive care therapy and pre-conditioning ... 59
Table 7 Schedule of events Part II: 6 months safety follow-up ... 60
Table 8 Schedule of events Part III: long term follow-up ... 62
Table 9 Planned safety review committee meetings ... 73
Table 10 Serology parameters ... 91
Table 11 Pre-conditioning therapy and supportive care overview ... 99
Table 12 Biomarker analysis schedule: pTTL (Part I and pTTL manufacturing) ... 100
Table 13 Biomarker analysis schedule: tumour ... 101
Table 14 Biomarker analysis schedule Part I and Part II: blood ... 101
Table 15 Biomarker analysis schedule Part III: blood ... 102
Table 16 Criteria for best overall response ... 104
Table 17 Safety laboratory parameters ... 113
Table 18 Estimated blood volumes Part I ... 115
Table 19 Estimated blood volumes Part II ... 115
Table 20 Estimated blood volumes Part III ... 115

List of Figures

Figure 1 Mechanism of activation and expansion of tumour specific T cells using TC0301 30

Figure 2 Overview of pTTL ACT 35

Figure 3 Overview of trial procedures (Part I, Part II and Part III) 51

Figure 4 pTTL pre-treatment chemotherapy and administration schedule 56

Figure 5 pTTL manufacturing overview 86

Appendices

Appendix 1 Management of Adverse Event Manual, Version 1.0 06JUN2022.

3 LIST OF ABBREVIATIONS AND DEFINITIONS OF TERMS

3.1 List of abbreviations

Abbreviation	Explanation
ACT	Adoptive cell therapy
ADL	Activities of daily living
AE	Adverse event
AESI	Adverse event of special interest
ALP	Alkaline phosphatase
ALT	Alanine aminotransferase
APC	Antigen-presenting cells
APTT	Activated partial thromboplastin time
AST	Aspartate aminotransferase
ATC	Anatomical therapeutic chemical
ATIMP	Advanced therapeutic investigational medicinal product
BMI	Body mass index
BSA	Body surface area
CA	Competent authority
CAR	Chimeric antigen receptor
CD	Cluster of differentiation
CIOMS	Council for International Organisations of Medical Sciences
CMS	Consensus molecular subtypes
CNS	Central nervous system
CR	Complete response
CRC	Colorectal cancer
CRES	CAR T-related encephalopathy syndrome
CRM	Clinical research manager
CRP	C-reactive protein
CRS	Cytokine release syndrome
CS10	Cryostor® Cryopreservation medium
CT	Computed tomography
CTP	Clinical trial protocol
CTC	Clinical Trial Consultants AB
CTCAE	Common terminology criteria for adverse events
ctDNA	Circulating tumour DNA
DLCO	Diffusing capacity of the lung for carbon monoxide
DLCOco	DLCO corrected for haemoglobin value
DLI	Donor lymphocyte infusion
DMSO	Dimethyl sulfoxide
CLL	chronic lymphatic leukaemia
CMV	Cytomegalovirus
DMP	Data management plan
DS	Drug substance

Abbreviation	Explanation
DSUR	Development safety update report
EBV	Epstein-Barr virus
ECG	Electrocardiogram
ECOG	Eastern Cooperative Oncology Group
eCRF	Electronic case report form
EDC	Electronic data capture
EEA	European Economic Area
EMA	European Medicines Agency
EPAR	European public assessment reports
EVF	Erythrocyte volume fraction
FAP	Familial adenomatous polyposis
FDA	U.S. Food and Drug Administration
FEV1	Forced expiratory volume in 1 second
FIH	First-in-human
GCP	Good clinical practice
GCS	Glasgow Coma Scale
GDPR	General data protection regulation
GLP	Good laboratory practice
GMP	Good manufacturing practice
Hb	Haemoglobin
HIV	Human immunodeficiency virus
HLA	Human leukocyte antigen
HLH	Hemophagocytic lymphohistiocytosis
HNPCC	Hereditary non-polyposis colorectal cancer
HSA	Human serum albumin
HTLV	Human T-lymphotropic virus type 1
IB	Investigator's brochure
ICANS	Immune effector cell-associated neurotoxicity syndrome
ICE	Impact, confidence and ease
ICF	Informed consent form
ICH	International Council for Harmonisation of Technical Requirements for Pharmaceuticals for Human Use
ICU	Intensive care unit
IEC	Independent ethics committee
iCR	Complete response
IL	Interleukin
IME	Important medical event
IMPD	Investigational medicinal product dossier
iPR	Partial response
iSD	Stable disease
ISF	Investigator site file
iSRC	Internal safety review committee
IUD	Intrauterine device

Abbreviation	Explanation
iCPD	Confirmed progression
iUPD	Unconfirmed progression
IUS	Intrauterine hormone-releasing system
LD	Longest diameter
LDH	Lactate dehydrogenase
LN	Lymph node
LVEF	Left ventricular ejection fraction
MAS	Macrophage activation syndrome
MCH	Mean corpuscular haemoglobin
MCV	Mean corpuscular volume
MedDRA	Medical dictionary for regulatory activities
MHC	Major histocompatibility complex
MoA	Mechanism of action
MPA	Medical products agency
MRI	Magnetic resonance imaging
MSI	Microsatellite instability
NCI	National cancer institute
NGS	Next generation sequencing
NIH	National Institute of Health
NIMP	Non-investigational medicinal product
NK	Natural killer
NYHA	New York Heart Association
PBID	Patient batch identity code
PD	Progressive disease
PII	Personally Identifiable Information
PK (INR)	Prothrombin complex international normalised ratio
PR	Partial response
PT	Preferred term
PV	Pharmacovigilance
QA	Quality assurance
QC	Quality control
RBM	Risk based monitoring
RLN	Regional lymph node
RSI	Reference safety information
SAR	Serious adverse reaction
SAE	Serious adverse event
SAP	Statistical analysis plan
SD	Stable disease
SDV	Source data verification
SmPC	Summary of product characteristics
SIV	Site initiation visit
SLN	Sentinel lymph node
SOC	System organ class

Abbreviation	Explanation
SOP	Standard operating procedures
SR	Sedimentation rate
SUSAR	Suspected unexpected serious adverse reaction
TCR	T cell receptor
TCRseq	T cell receptor sequencing
Tcm	Central memory T cells
Tem	Effector memory T cells
TIA	Transient ischemic attack
TIL	Tumour-infiltrating lymphocyte
TLS	Tumour lysis syndrome
TMF	Trial master file
TNF	Tumour necrosis factor
UBC	Urinary bladder cancer
VZV	Varicella-zoster virus
WES	Whole exome sequencing
WHO	World Health Organisation

3.2 List of trial definitions

Definition	Explanation
**Antigen**	A molecule, or molecular structure, which the immune system can identify and react against
**Drug product**	Formulated DS, cell suspension in Ringer-Acetate, human serum albumin (HSA; 25 mg/mL) mixed volume/volume with the cryopreservation medium Cryostor® CS10 for intravenous infusion. The final HSA concentration after mixture will be 12.5 mg/mL.
**Drug substance (DS)**	The cell suspension obtained upon cell culture harvest at the end of the pTTL manufacturing process, from which TC0301 has been removed
**EpiTCer beads**	Common designation of beads linked with any protein containing one or several antigenic epitopes
**NAG**	A recombinantly produced protein containing multiple neoantigen epitopes, a coupling tag, and a purification tag
**Neoantigen**	A protein or peptide containing somatic mutation(s) which translates into an altered primary sequence
**Neoantigen epitope**	A peptide (typically 8-25 amino acid residues) containing somatic mutation(s) that in complex with major histocompatibility complex (MHC) class I or II on antigen presenting cells display a putative site where the T cell may bind
**PIOR® Manufacturing**	The bioinformatic software system applied within the manufacturing process of EpiTCer® beads for design of neoantigen epitope-encoding fragments, based on processing of NGS data
**pTTL**	personalised Tumour Trained Lymphocytes, product name used for the investigational product. No recommended international non-proprietary name (INN) is yet available
**Regional lymph node (RLN)**	Lymph nodes (LN) in anatomical proximity to tumour tissue that are likely to receive lymphatic drainage from the tumour
**Starting material**	Surgically obtained RLNs are used as starting material for pTTL manufacture
**TC0301**	Sponsor's code for EpiTCer® beads with NAG: raw material in personalised Tumour Trained Lymphocytes (pTTL) manufacturing

4 IMPORTANT MEDICAL PROCEDURES TO BE FOLLOWED BY THE INVESTIGATOR

4.1 Medical emergencies contacts

The Principal Investigator at each site is responsible for ensuring that procedures and expertise are available to handle medical emergencies during the trial. A medical emergency usually constitutes a serious adverse event (SAE) and is to be reported as such. Detailed SAE reporting procedures are described in Section 11.3.1.14.

In the case of a medical emergency, the Investigator should primarily contact the Sponsor's Medical Representative. For contact details to the Sponsor's Medical Representative and the Medical Monitor, see Table 1.

Table 1 Medical emergencies contact

Name	Function in the trial	Telephone number and e-mail
[redacted], MD, PhD	Sponsor's Medical Representative	Telephone: [redacted]<br>E-mail: [redacted]@neogap.se
[redacted], MD	Medical Monitor	Telephone: [redacted]<br>E-mail: [redacted]@ctc-ab.se

5 INVESTIGATORS AND TRIAL ADMINISTRATIVE STRUCTURE

5.1 Trial administrative structure

**Sponsor**<br>NEOGAP Therapeutics AB<br>Cancer Center Karolinska<br>Visionsgatan 56<br>SE-171 64 Solna, Sweden	**Sponsor's Medical Representative**<br>[redacted]<br>Phone: [redacted]<br>E-mail: [redacted]@neogap.se<br>**Sponsor's Project Manager**<br>[redacted]<br>Phone: [redacted]<br>E-mail: [redacted]@neogap.se
**Trial management**<br>Clinical Trial Consultants AB (CTC)<br>Dag Hammarskjölds väg 10B<br>SE-752 37 Uppsala, Sweden	**Clinical Research Manager**<br>[redacted]<br>Phone: [redacted]<br>E-mail: [redacted]@ctc-ab.se<br>**Biostatistician**<br>[redacted]<br>Phone: [redacted]<br>E-mail: [redacted]@ctc-ab.se<br>**Medical writers**<br>[redacted]<br>Phone: [redacted]<br>E-mail: [redacted]@neogap.se<br>[redacted]<br>Phone: [redacted]<br>E-mail: [redacted]@neogap.se<br>[redacted]<br>Phone: [redacted]<br>E-mail: [redacted]@ctc-ab.se<br>**Medical monitor**<br>[redacted]<br>Phone: [redacted]<br>E-mail: [redacted]@ctc-ab.se
**Laboratory (Safety Lab)**	Local hospital laboratories
**Next generation sequencing (NGS):** whole exome sequencing (WES) and RNA sequencing based on tumour material	CeGaT Gmbh<br>[redacted]. 23h<br>DE-72076 Tübingen, Germany
**Laboratories for exploratory biomarker analyses**	To be decided (TBD)
**Advanced therapeutic investigational medicinal product (ATIMP) manufacturing, packaging, labelling and release**	Vecura (KCC, Karolinska Cell Therapy Center)<br>Karolinska University Hospital Huddinge, Novum<br>SE-141 86 Stockholm, Sweden
**Electronic data capture (EDC) system provider**	Viedoc Technologies AB<br>Stationsgatan 23<br>SE-753 40 Uppsala, Sweden

5.2 Trial conduct

Unit	Function in trial	Investigators
Karolinska University Hospital, Solna:<br>(i) Phase I unit, Center for Clinical Cancer Studies, Theme Cancer<br>(ii) Medical Unit Pelvic cancer - Colorectal cancer, Theme Cancer<br>Karolinska University Hospital, Huddinge:<br>Medical Unit Cell Therapy and Allogeneic Stem cell Transplantation (ME CAST)	**Recruitment, Surgery & Treatment Site:**<br>**<u>Part I:</u>**<br>Screening of local and referred patients<br>Surgery and biopsy:<br>• Collection of tumour material via either (i) standard surgery for palliative purposes (e.g. primary tumour surgery) or (ii) biopsy (if trial specific surgery)<br>• Collection of regional lymph nodes (RLNs) (pTTL starting material) in association with either (i) standard surgery or (ii) trial specific surgery.<br>**<u>Part II:</u>** screening, pre-conditioning, pTTL administration, follow-up of patients after pTTL administration<br>**<u>Part III:</u>** patient follow-up	**Coordinating Investigator/ Principal Investigator**<br>[redacted]<br>Phone: [redacted]<br>E-mail:<br>[redacted]@regionstockholm.se<br>**Investigators**<br>[redacted]<br>Phone: [redacted]<br>E-mail:<br>[redacted]@regionstockholm.se<br>[redacted]<br>Phone: [redacted]<br>E-mail: [redacted]@regionstockholm.se
Västmanlands hospital Västerås<br>Department of Surgery	**Recruitment & Surgery Site 1:**<br>**<u>Part I:</u>**<br>Screening of local patients<br>Surgery and biopsy:<br>• Collection of tumour material via either (i) standard surgery for palliative purposes (e.g. primary tumour surgery) or (ii) biopsy (if trial specific surgery)<br>• Collection of regional lymph nodes (RLNs) (pTTL starting material) in association with either (i) standard surgery or (ii) trial specific surgery.	**Principal Investigator**<br>[redacted]<br>Phone: [redacted]<br>E-mail:<br>[redacted]@regionvastmanland.se<br>**Investigators**<br>[redacted]

Danderyd's Hospital AB<br>Department of Surgery and Urology	**Recruitment & Surgery Site 2:**<br>**<u>Part I:</u>**<br>Screening of local patients<br>Surgery and biopsy:<br>• Collection of tumour material via either (i) standard surgery for palliative purposes (e.g. primary tumour surgery) or (ii) biopsy (if trial specific surgery)<br>• Collection of regional lymph nodes (RLNs) (pTTL starting material) in association with either (i) standard surgery or (ii) trial specific surgery.	**Principal Investigator**<br>[redacted]<br>Phone: [redacted]<br>E-mail: [redacted]@regionstockholm.se
Capio S:t Görans Hospital, Stockholm, Sweden<br>Section of Oncology, Department of surgery	**Referral Site:** patient identification	

Additional referral sites may be added.

Signatures are provided in Section 19.

6 INTRODUCTION

6.1 Background

Cancer is a major cause of mortality world-wide and is expected to become the most significant barrier to increased life expectancy in the 21st century (Bray et al. 2018). During 2018, 18.1 million new cancer cases were diagnosed, and 9.6 million deaths attributable to malignancy were recorded, illustrating the impact of cancer on human health (Bray et al. 2018). The emergence of new treatment options has greatly improved the prognosis of many cancer types, but, as the above statistics clearly indicate, additional therapeutic strategies are needed.

6.1.1 *Cancer Immunotherapy*

During the last decade, therapies that utilise the immune system as a tool to combat cancer have emerged. These therapies have as a common principle that they augment anti-tumour immunity in order to overcome the escape mechanisms that protect an established cancer from immune-mediated destruction. The immune system continuously monitors for, and eradicates, malignantly transformed cells through a mechanism termed cancer immunosurveillance (Finn 2018). Immune-mediated tumour cell destruction is triggered by aberrations arising during malignant transformation which distinguishes cancer cells from normal cells. These aberrations include mutations in the DNA of tumour cells giving rise to modified proteins, so-called neoantigens, that can be recognised as foreign by the immune system. However, cancer immunosurveillance is counteracted by the tumour's escape mechanisms. The capacity to avoid immune-mediated destruction is defined as one of the hallmarks of cancer (Hanahan and Weinberg 2011).

One therapeutic strategy which has reaped success and has further potential is adoptive cell therapy (ACT), where tumour-targeting immune cells are used to directly increase anti-tumour immunity. Notable examples include tumour-infiltrating lymphocyte (TIL) therapy (Besser et al. 2020; Chesney et al. 2022; Dudley et al. 2002; Goff et al. 2016; Haanen et al. 2022; Rosenberg et al. 2011) and treatment with chimeric antigen receptor (CAR) - and transgenic T cell receptor (TCR)-modified T cells (Kochenderfer et al. 2012, 2015; Lee et al. 2015; Lu et al. 2017; Morgan et al. 2006; Robbins et al. 2011). Among these, TIL therapy is the one closest resembling pTTL. Its rationale is based on enrichment of tumour-specific T cells present in the tumour environment, and the use of non-specific *in vitro* stimulation to induce activation, break tolerance, and promote proliferation. Another strategy, which is applied in the present trial, is to use antigen-specific *in vitro* stimulation with neoantigens (Schumacher and Schreiber 2015; Ward, Gubin, and Schreiber 2016) to activate and expand an enriched T cell population. A major benefit of targeting neoantigens is that they are highly specific for the individual targeted tumour and absent in normal tissues. This reduces the risk of off-target toxicity. However, neoantigen targeted therapies require a personalised approach. In the current trial neoantigen peptides will be produced based on sequencing data from the patient's own tumour and used for T cell stimulation. This approach could be applicable to many types of cancer, as the presence of mutations with the potential of giving rise to neoantigens is the only absolute prerequisite.

6.1.2 *Target patient group*

The pTTL therapy could in theory be used in any cancer where neoantigenic mutations have occurred and are expressed as protein. Accessible tumour-draining regional lymph nodes (RLNs) for T cell expansion is a second prerequisite. These criteria can be met in patients

with various cancer types (Schumacher and Schreiber 2015). For this initial first-in-human (FIH) trial, colorectal cancer (CRC) has been selected.

CRC is one of the most common cancer forms in the industrialised countries. Over 1.93 million new cases and close to 0.94 million deaths are estimated every year of the disease (Xi and Xu 2021). In Sweden, approximately 6500 cases are diagnosed yearly (Nationellt kvalitetsregister för tjock- och ändtarmscancer, 2018). Surgery is the primary treatment modality, in combination with chemotherapy, and, mainly in rectal cancer, radiotherapy, in more advanced and aggressive disease. Over 90% of patients with stage I-II tumours (which constitute 40-50%) achieve long-term survival, while patients with stage IV metastasized disease have poor prognosis, with a 5-year survival below 20% (Rawla, Sunkara, and Barsouk 2019; Nationellt kvalitetsregister för tjock- och ändtarmscancer, 2018). The focus of the current trial is patients with stage IV CRC.

There are approximately 900 cases of stage IV colon cancer and 350 cases of stage IV rectal cancer per year in Sweden (Nationellt kvalitetsregister för tjock- och ändtarmscancer, 2018). Newer treatment regimens with chemotherapy in combination with targeted therapies directed against angiogenesis and different steps of the EGF/KRAS/BRAF/MEK pathway, and checkpoint inhibitors, have markedly prolonged the survival of the entire group of stage IV CRC patients, but stage IV CRC is a considerable heterogenous disease group, and effective therapies are still lacking for subgroups of patients (Jung et al. 2021).

The prognosis of patients with stage IV CRC depends on several factors, including the number and localisation of distant metastasis, and whether radical surgery can be performed. The prognosis is also highly influenced by the molecular and immunological characteristics of the cancer, as is the efficacy of available standard therapies (Guinney et al. 2015; Sveen, Kopetz, and Lothe 2020). As an example, only consensus molecular subtype 1 (CMS1)-type tumours respond significantly to checkpoint inhibition. The latter highlights the significance of the differences in immunological activity and infiltration into the tumour by different classes of immune cells between subgroups, and indicates that sensitivity to cellular immunotherapy could vary. However, it has also been noted that the relative degree of immune infiltration is correlated to prognosis across different CRC subtypes (Tosolini et al. 2011, Galon et al. 2006, Fridman et al. 2017). Although the degree of success of pTTL therapy can be expected to be influenced by multiple factors, there is a rationale for adoptive T cell therapy in the entire group, as improved infiltration by activated T cells could be hypothesised to be beneficial across subgroups, and to not be limited to CMS1.

6.2 Product characteristics and mechanism of action

pTTL, personalised Tumour Trained Lymphocytes, consists of *in vitro* expanded autologous neoantigen-specific T cells intended for re-infusion to the patient. The T cells for pTTL are sourced from RLNs, which are naturally enriched for neoantigen-reactive T cells.

Source of T cells for pTTL

The protocol exploits natural enrichment of tumour-specific T cells, in a manner similar to the TIL concept, by using RLNs as the source of T cells for production. RLNs receive lymphatic drainage from the tumour tissue, leading to exposure to tumour antigens via the lymphatic drainage and through migration of antigen-presenting cells from the tumour to the draining lymph node (LN). The T cell population found in the LN is consequently exposed to tumour antigens, and a resulting enrichment of T cells specific for these antigens occurs here.
The first LN(s) to receive lymphatic drainage, the sentinel LN, (SLN, most often 1 to 3 lymph nodes) has the most enriched T cell population (Marits et al. 2006) as a consequence of its

localisation. SLN detection will be performed when technically feasible but will not be possible in all patients in this trial. RLNs will then be retrieved based on anatomical location and enlarged size. The RLN starting material is defined as lymph nodes in anatomical proximity to malignant tissue that receive, or are likely to receive, lymphatic drainage from the tumour.

pTTL manufacturing

The RLNs are surgically harvested and processed *in vitro*, forming the starting material for pTTL manufacturing. The starting material is cryopreserved until TC0301, a raw material for pTTL production, is available and the patient is ready to enter the treatment part, Part II, of the trial. pTTL production is started within Part I of the trial in parallel to inclusion of the patient in Part II.

TC0301 is produced using an array of selected personalised neoantigens. The selection of neoantigens for TC0301 production is based on a bioinformatic software system developed by the Sponsor (PIOR® Manufacturing) for design of neoantigen epitope-encoding DNA fragments, in turn used for production of neoantigen epitope-containing protein (NAG). A maximum of 36 mutations are selected (depending on the possibilities of each patient's mutational landscape) and divided in up to 6 individually designed NAGs which are then manufactured by recombinant production. The NAGs are covalently coupled to paramagnetic beads to form TC0301.

In vitro T cell stimulation with TC0301 is a process including phagocytosis, processing, and presentation on major histocompatibility complex (MHC) molecules by antigen presenting cells (APCs), see Figure 1. The micrometre size of TC0301 promotes phagocytosis, and the beads are readily engulfed by APCs present in the RLN material. The NAG carried on TC0301 are then processed and presented as shorter peptides on MHC molecules in a physiological manner. This enables presentation of multiple short peptides derived from the same NAG and makes the methodology independent of human leukocyte antigen (HLA) type.

The manufacturing process also includes stimulation with interleukin 2 (IL-2), well known to efficiently promote proliferation of T cells (Rosenberg 2014). A relatively low concentration of IL-2 was selected for the expansion protocol as this cytokine is known, besides promoting expansion of functional and activated T cells, to also stimulate differentiation towards a senescent phenotype, especially in high concentrations and with longer *in vitro* culture time (Petrozziello, Sturmheit, and Mondino 2015; Yang et al. 2011; Zhou et al. 2011), and lower concentrations and shorter protocols have been found to correlate with better outcome in CAR T cell therapy (Ramos et al. 2016).

The manufacturing process takes approximately 2 weeks (12 to 15 days). At the end of production, the product is cryopreserved. As outlined in Section 10.8, pTTL is thawed just before administration. The pTTL product contains patient-derived neoantigen-specific T cells that have been activated and expanded to promote successful tumour eradication.

Figure 1 Mechanism of activation and expansion of tumour specific T cells using TC0301

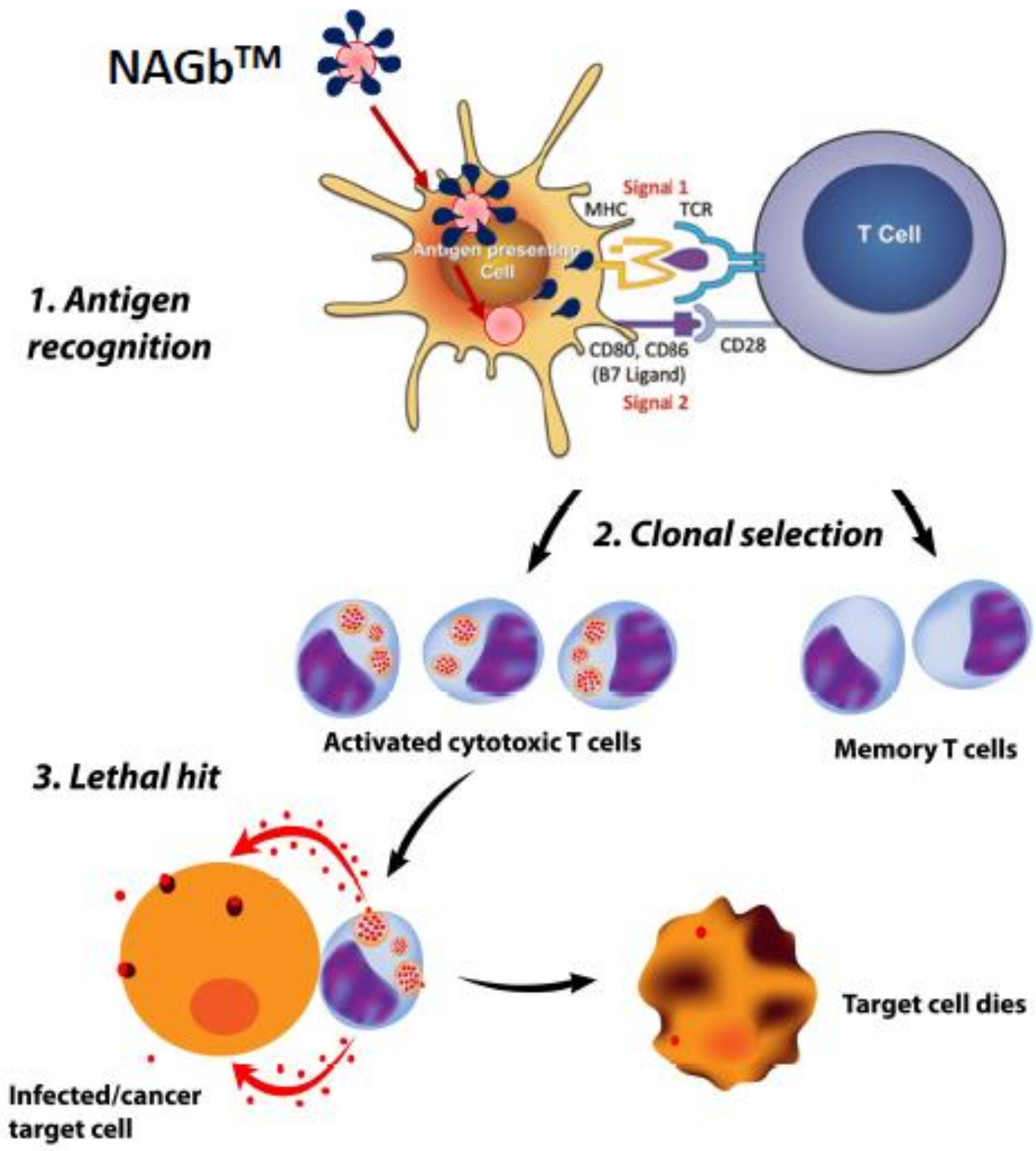

APC=antigen-presenting cells, MHC=major histocompatibility complex, TCR=T cell receptor

TC0301 are readily taken up by APCs. NAG are processed to peptides that are loaded onto MHC molecules, making use of the APC's machinery for antigen presentation. T cells carrying a TCR specific for a NAG-derived peptide in the context of MHC I or II will receive an antigen specific activating signal (signal 1) through TCR signalling and a co-stimulatory signal (signal 2) through costimulatory molecules expressed on the APC through cluster of differentiation 28 (CD28) signalling. This will lead to selective clonal expansion of NAG-specific T cells, giving rise to activated cytotoxic T cells capable of killing tumour cells directly and to memory T cells able to reactivate and kill tumour *in vivo*.

6.3 Non-clinical development

Antigen-specific T cells have long been known to induce specific destruction of cells expressing their appropriate target antigen, *i.e.,* they are capable of selective response limited to tissue expressing their cognate antigens without toxicity to surrounding tissue. Adoptive transfer of polyclonally activated T cells from tumour-draining lymph nodes induces cell destruction observed to be confined to tumour deposits (Wang et al. 2007). The idea of adoptive therapy with tumour-draining lymph node-derived T cells originated from such observations of selective anti-tumour reactivity. Each pTTL product contains a unique T cell population derived from the T cell repertoire present in the RLNs and selected further by stimulation with personalized TC0301. The biological activity of pTTL depends on the composition and characteristics of the T cells and is only measurable in the context of that individual's own tumour. It follows that every pTTL is unique and, consequently, non-clinical studies to investigate pharmacodynamics, pharmacokinetics and toxicity in animal models in a regular pharmacological sense are not feasible. However, the pharmacokinetics and biodistribution properties of the autologous cells of pTTL are not expected to differ from other intravenously injected genetically unmanipulated cells.

Non-clinical studies performed by the Sponsor to support the anticipated mode of action (MoA) of pTTL are described in the Investigator's Brochure (IB).

Summary of non-clinical data

pTTL has been extensively characterised *in vitro* in terms of cell composition and phenotypic markers for T cells with expected biological activity and markers associated with suboptimal biological activity. These studies show that pTTL is an oligoclonal T cell product, mainly consisting of central memory (cm) and effector memory (em) T cells (considered to be associated with expected biological activity *in vivo*) and enriched for certain T cell clones. Both CD4+ and CD8+ T cells make up the product, and the CD4/CD8 T cell ratio varies between individual products. Among other immune cells that to a minor extent are present in pTTL, NK cells are the dominating cell type. An overview of the cell composition of pTTL is shown in Table 2.

Table 2 Overview of pTTL cell composition and phenotypic characteristics

**Cell population**	**Proportion in % median (n[1])**	**Description**
T cells	**86** (n=11/15)	CD45+CD3+ of live cells
CD4+ T cells	**78** (n=15/15)	CD4+ of CD3+ live cells
CD8+ T cells	**18** (n=15/15)	CD8+ of CD3+ live cells
CD4+ cells w expected *in vivo* capacity (Tem+Tcm)	**72** (n=14/15)	CD45RA-CCR7+/-(CD95+) of live CD3+CD4+ cells
CD8+ cells w expected *in vivo* capacity (Tem+Tcm)	**84** (n=14/15)	CD45RA-CCR7+/-(CD95+) of live CD3+CD8+ cells
Treg cells	**1.8** (n=7)	Demethylated FOXP3 promoter quantified by PureQuant[2]
NK cells	**6.8** (n=15/15)	CD56+ of live cells
B cells	**3.1** (n=15/15)	CD19+(HLADR+) of live cells

Tem=effector memory T cells, Tcm=central memory T cells

1. n = number of analysed pTTL batches, analysed by pTTL release analysis FACS panel (n=11) or an R&D developed FACS panel (n=15). Note that analysis of CD45RA did not work for one pTTL batch and Tem and Tcm data are therefore based on n=14 batches.
2. PureQuant method used to analyse FOXP3 promoter demethylation to quantify regulatory T cells. Data shown for the n=7 pTTL products analysed.

The MoA of pTTL has not been directly assessed in a tumour model setting. However, the capacity of prophylactically administered TC0301 to induce an immune response able to supress tumour growth has been demonstrated in a murine MC38 colon carcinoma model (Corbett et al. 1975), where EpiTCer® beads with NAG that were selected with a development version of PIOR® clearly delayed MC38 tumour growth (result presented in IB). Additionally, the capability of EpiTCer® beads to selectively stimulate expansion of T cells specifically targeting neoantigens from a melanoma patient has been studied in a human *in vitro* model system where reactivity to autologous or allogeneic tumour cell lines could be assessed. It was shown that the T cells induced by the EpiTCer® beads display both tumour specificity, with little cross-reactivity to normal autologous cells, and selectivity to autologous tumour cells compared to allogeneic tumour cells (Kiessling et al. 2022).

The toxicity of the paramagnetic microbead component of TC0301 used during pTTL manufacture has been assessed in an acute toxicity single-dose study in rats. The microbeads are removed by a magnet before pTTL formulation in a process called debeading, but even the maximum amount of added TC0301 that could theoretically be present in pTTL, if no debeading was applied, was shown to provide a 12.5 x safety margin, calculated on a person weight of 70 kg, to the evaluated safe dose. From this study it can be concluded that TC0301 does not constitute a risk for toxicity of pTTL.

6.4 Clinical experience

Since the current trial is a FIH trial, no prior clinical trials with pTTL have been performed. At the data cutoff of this version of the CTP (January 31st, 2025) two patients have been treated in the FIH trial. No safety concerns have been identified. There have been no withdrawals due to adverse events (AEs) and all AEs, including adverse events of special interest (AESIs), assessed as related to pTTL have been of grade 1. One SAE, back pain grade 2, has been reported for patient 101 (SAE criterion: hospitalisation). The event was assessed as unlikely related to pTTL but as related to the underlying disease.

Considering that only initial safety data from two treated patients are available for pTTL, other studies on T cell ACTs relevant for evaluating the expected safety profile of pTTL are described below.

In general, safety profile assessments for ACTs similar to pTTL, i.e. T cell therapies which have not undergone genetic manipulation, have been favourable, with limited toxicity associated to the T cell product.

TIL therapy resembles pTTL in several aspects. It is, like pTTL, based on the sourcing of an enriched tumour-specific T cell population (tumour in the case of TILs and RLN for pTTL), includes no genetic modification, and generates a T cell product with multiple T cell clones and specificities. However, the use of non-specific *in vitro* stimulation to induce activation, break tolerance, and induce proliferation of TILs differs from the neoantigen-targeting approach of pTTL. Moreover, in addition to pre-conditioning chemotherapy, which is also applied for pTTL (and mostly based on the same drugs as for pTTL), post-infusion cytokine administration is only used in the TIL setting. TIL therapy is applied mainly in malignant melanoma and has been shown to induce objective response in approximately 40-50% of treated patients, with complete responses in 10-20% of patients (Dafni et al, 2019, Rohaan et al. 2022). The TIL product Lifileucel was under the name AMTAGVI™ approved by the US Food and Drug Administration (FDA) in February 2024 for treatment of patients with unresectable or metastatic melanoma (AMTAGVI FDA Summary Basis for Regulatory Action. 2024). The objective response rate in the pivotal phase 2 trial was 31%, with a CR rate of 5%, in a heavily treated patient group (Chesney et al, 2022). The use of post-infusion cytokine treatment complicates comparisons between the therapies, especially with regards to AEs, as cytokine therapy is associated with considerable toxicity (Andersen et al. 2016; Besser et al. 2020; Dudley et al. 2008; Goff et al. 2016; Rosenberg et al. 2011; Sarnaik et al. 2021). Reported AEs from the Phase 2 trial of Lifileucel in patients with advanced melanoma were shown to be consistent with expected reactions to the lymphodepletion pretreatment and post-treatment IL-2, as well as a consequence of the underlying disease (Chesney et al. 2022). The most common treatment-emerging AEs were thrombocytopenia, anemia, and febrile neutropenia and the AE incidence decreased rapidly over the first two weeks after Lifileucel infusion. In a Phase III trial in advanced melanoma, reported AEs in patients treated with TILs were mainly chemotherapy-related myelosuppression (Rohaan et al. 2022).

Also similar to pTTL is a therapy utilising tumour-draining LNs, or more precisely sentinel lymph nodes (SLNs), as a source of tumour-targeting T cells. This ACT, developed by Prof. O. Winqvist and co-workers, has previously been applied in CRC (Karlsson et al. 2010; Zhen et al. 2015) and urinary bladder cancer (UBC; Sherif et al. 2015). The therapy was shown to induce clinical responses, with five out of nine treated stage IV CRC patients responding (4 complete responses and one partial response) in the first trial (Karlsson et al. 2010). In the second trial, a significantly increased 24-month survival rate was noted for nine SLN T cell treated stage IV CRC patients compared to a control group of 14 patients: 55.6 vs. 17.5 % (p = 0.02), but overall survival was not significantly prolonged (Zhen et al. 2015). In the third trial, including patients with urinary bladder cancer, objective responses was seen in two patients (one CR and one PR) out of nine treated patients (Sherif et al. 2015). No significant toxicity was reported for this therapy.

Although the sourcing of T cells is similar, this SLN-based therapy employs stimulation with tumour lysate to direct the T cell expansion against tumour antigens, in contrast to the neoantigen-epitope containing TC0301 used for stimulation in pTTL. TC0301 contains only selected neoantigen epitopes, while a tumour lysate contains all proteins contained in the tumour, of which the majority are normal. T cells expanded with tumour lysate stimulation could thus target normal proteins, and the proportion of tumour-specific T cells is expected to be lower than when stimulation with tumour-specific neoantigens is employed. Thus, the tumour-targeting precision is expected to be higher, and the likelihood of off-target toxicity potentially lower with pTTL than the SLN-based therapy developed by Winqvist and colleagues. Moreover, unlike pTTL, this ACT is a stand-alone therapy where no pre-conditioning chemotherapy or post-infusion cytokine therapy is administered (Karlsson et al. 2010; Sherif et al. 2015; Zhen et al. 2015).

In addition to the ACTs described above, some ATIMPs with similarities to pTTL have reached clinical stage and data are available from early clinical phase trials. The cell products TIDAL-01 (Turnstone Biologics), cNeT (Achilles Therapeutics) and GEN-011 (Genocea Bioscience) are all neoantigen-targeting T cell products. They differ from pTTL regarding the starting material for ATIMP production: cNeT and TIDAL-01 products were based on neoantigen-specific stimulation of tumour tissue-derived T cells in analogy with TILs, including similar pre-conditioning therapy and post-infusion IL-2, while GEN-011 was focused on neoantigen-directed T cells derived from peripheral blood. cNeT was found to have an acceptable safety profile, similar to unselected TILs, in a preliminary evaluation of patients in two Phase I/II studies in malignant melanoma and non-small cell lung cancer. Of note however, three cases of cytokine release syndrome (CRS) and one case of immune effector cell-associated neurotoxicity syndrome (ICANS) were reported (Turajlic et al. 2021). This could indicate that neoantigen targeting might result in both higher efficacy and increased toxicity compared to TIL therapy. Genocea’s early clinical program had a slightly different setup including two arms: one with pre-conditioning chemotherapy prior to one cell infusion followed by IL-2 injections, and the other with administration of multiple smaller cell doses without prior chemotherapy and followed by post-infusion IL-2. Similar tolerability data were reported for the two dose cohorts and CRS, maximum grade 2, and one grade 2 ICANS were reported (Gillison et al., 2022). The TIL product TIDAL-01, composed of selected neoantigen-activated T cells, was tested in two Phase I trials (NCT05576077 and NCT05628883) targeting multiple cancer indications, including CRC. Early result from CRC patients in these trials revealed complete response (ongoing 48 weeks after treatment) in one of four treated patients with CRC (Turnstone Biologics Press release August 14, 2024), further supporting the concept of neoantigen-targeted cell therapy in CRC. Safety data were consistent with expected AE profiles associated with the lymphodepletion regimen and IL-2

(applied in both trials), and Pembrolizumab (applied in NCT05576077). The clinical trial programs for the three mentioned neoantigen targeted cell products have all been halted. Importantly, the discontinuation was not due to any safety concerns.

In summary, extensive clinical experience of TIL therapy has established that AEs are mostly linked to preconditioning therapy or IL-2 and are not considered to be reactions to the TILs. High grade CRS and ICANS are generally not seen with TIL therapy (Betof Warner et al. 2024). The TIL therapy Lifileucel (Amtagvi) is approved for treating malignant melanoma since 2024. The safety profile of neoantigen-targeted cell products, cNeT, TIDAL-01 and GEN-011, is similar to that of TIL therapy, i.e., reactions to treatment are mainly associated to the accompanying preconditioning or post infusion cytokine therapy.

6.5 Trial rationale

The present trial combines harvest of enriched tumour specific T cells from RLNs and application of patient-specific neoantigen technology. Neoantigen-driven *in vitro* T cell stimulation promotes activation and proliferation of T cells specific for a combination of neoantigens uniquely expressed in the autologous tumour. This strategy could prove more efficacious than non-personalised immunotherapy due to the possibility of expanding rare highly tumour-reactive T cell clones that otherwise wouldn't be capable of mounting a response, especially if a number of such clones with different target epitopes are expanded.

Targeting neoantigens has several benefits, including a broad applicability to many cancer types and the possibility to personalise each therapeutic product to an individual patient. The therapeutic strategy is cancer type-agnostic, however, the current FIH trial will only include patients with CRC. CRC is considered suitable for neoantigen targeted immunotherapy, as it is an immunogenic tumour, displaying an intermediate mutational load (Lee et al. 2018).

Proof-of-concept for neoantigens as efficient targets for cell therapy in CRC was provided in a case report on a metastatic CRC patient with a KRAS G12D mutant who was treated with a TIL product composed to 75% of clonotypes reactive to the KRAS mutant and who upon treatment displayed effective antitumour responses and cancer regression (Tran et al., 2016). Moreover, a complete response was reported for one CRC patient treated with a similar cell therapy, the TIDAL-01 neoantigen targeted TIL therapy described above (Turnstone Biologics Press Release August 14, 2024). In a Phase II trial evaluating an ACT with personalised neoantigen-reactive TCR-transduced T cells, objective clinical responses were reported for 3 out of 7 patients with metastatic, mismatch repair-proficient colorectal cancers, further supporting the concept of neoantigens as therapeutic targets in this disease (Parkhurst et al., 2024). As mentioned in Section 6.1.2 “Target patient group”, a higher degree of tumour immune cell infiltration, specifically by T cells, is associated with better prognosis. In consequence, improving T cell infiltration with tumour-targeting adoptive T cell immunotherapy like pTTL has the potential to improve patient outcomes.

The *in vitro* stimulation with the selected NAGs allows activation of T cells outside the suppressive tumour-associated environment, in absence of the tolerogenic mechanisms which tumour-specific T cells have experienced *in vivo*. The increase of cell numbers resulting from the intense proliferation induced by antigen stimulation is also of importance, higher cell dose has been associated with increased chance of success, both in other SLN-derived adoptive T cell therapy (Karlsson et al. 2010) and in TIL therapy (Besser et al. 2020; Goff et al. 2016).

In conclusion, the hypothesis behind pTTL therapy is that the re-introduction of neoantigen-specific RLN T cells selected, activated, and expanded according to the pTTL production

process, could induce significant tumour eradication, and provide effective personalised T cell immunotherapy.

The application of pTTL for ACT in cancer is outlined in Figure 2, showing an overview of the ACT approach. The rational for the trial design is described in Section 8.3.

Figure 2 Overview of pTTL ACT

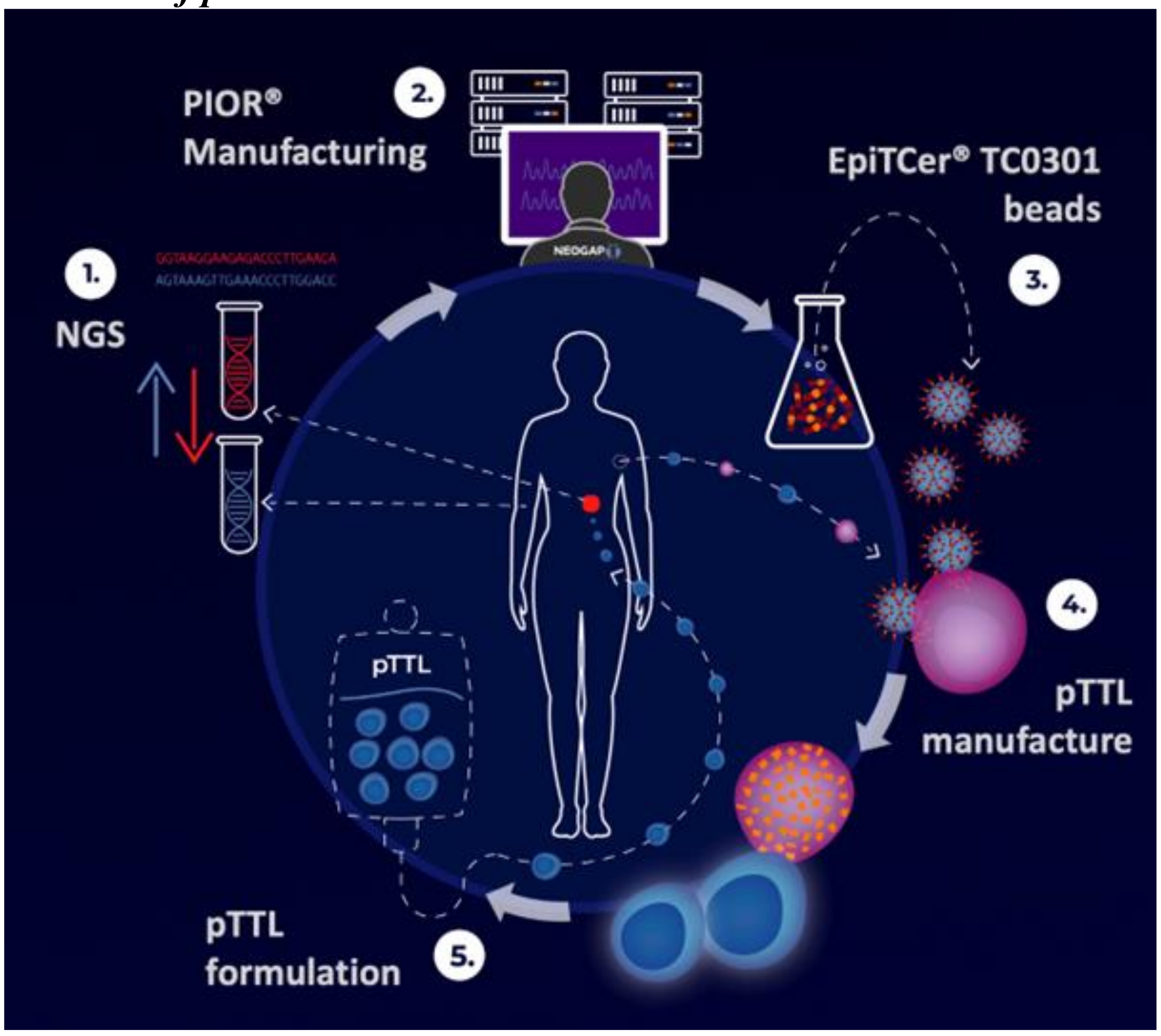

NGS=next generation sequencing

Tumour (red) and normal (*i.e.* blood) tissue (blue) is obtained from the patient and subjected to NGS (1). Tumour mutations are identified by NGS analysis and personalised NAG designed using the PIOR® Manufacturing bioinformatic software system (2). NAG (red spikes) are produced in bacteria by recombinant technology and coupled to paramagnetic beads (light blue spheres) to form the EpiTCer bead raw material TC0301 (3). pTTL is manufactured by *in vitro* stimulation of RLN cells obtained at surgery, leading to neoantigen selective expansion of autologous T cells (pink cells=APC, blue cells=T cells, orange dots=phagocytosed and processed TC0301) (4). The pTTL is formulated for autologous infusion to treat the cancer patient (5).

6.6 Risk/benefit assessment

In this FIH trial of a novel ATIMP, risks and benefits associated with the treatment are unknown. Prospective trial patients will be carefully informed about the lack of clinical experience with pTTL. In addition, patients will be provided with information about the efficacy and toxicity of other relevant T cell therapies. They will be made aware of potential treatment-associated, and trial procedure-associated risks and they will be informed that arguments about potential benefits are hypothetical at this stage.

6.6.1 *Benefits*

The subjects eligible for this FIH trial are patients with Stage IV CRC, with a poor prognosis and few or no therapeutic options available apart from participation in a clinical trial. Adoptive T cell therapies such as pTTL have the potential for major and long-lasting responses by harnessing the immune system's capacity to first mount an anti-tumoral immune response to eradicate cancer cells, and then to develop immunological memory, preventing cancer recurrence.

The safety profile for pTTL is expected to be similar to other T cell products. Precautions are taken to meet the requirements for the expected safety profile, but also to ensure preparedness for potential severe complications. By this, the safety of the subjects should be ensured as far as possible without the specific knowledge of risks associated to pTTL.

6.6.2 *Risks*

Potential risks associated with pTTL therapy can be divided into several different categories. A risk-based assessment has been performed that addresses the following types of risks:

1) tumour formation
2) unwanted immunogenicity
3) treatment failure
4) transmission of infectious organisms
5) toxicity and off-target reactions
6) risks associated with trial-related procedures, including side effects from pre-conditioning therapy

Perceived risks and associated mitigations are summarised below. A more detailed discussion on risk management is available in the IB (Section 6.9).

6.6.2.1 Risk for tumour formation

This risk could be divided into 2 separate entities:

1. Re-infusion of tumour cells present in the starting material.
2. Malignant transformation of normal cells during pTTL production.

Tumour cells can be present in the starting material, as the tumour-draining lymph nodes could contain metastases or isolated tumour cells. Thus, re-infusion of tumour cells with the pTTL product is theoretically possible. However, the conditions under which pTTL production takes place do not support tumour cell survival. A flow cytometry-based assay has been set up for tumour cell detection which is used for evaluation of the starting material to ensure awareness of tumour contamination in the starting material and vigilance when metastatic nodes are used. Tumour cells can also be recognised at microscopic inspection, and their presence and gradual decline during pTTL expansion is monitored. To ensure that re-infusion of cancer cells do not occur, absence of cancer cells is a product release criterion.

The risk of malignant transformation of normal cells during the pTTL production process is considered minimal. Cell growth is carefully monitored during the production process. The raw materials used in pTTL manufacture are not considered carcinogenic. Finally, the culture time in pTTL manufacture is relatively short and contains less intense and more physiological stimulation compared to many other T cell expansion protocols for clinical immunotherapy and should confer minimal risk for malignant transformation.

6.6.2.2 Unwanted immunogenicity

The starting material originates from the patient and the generated cell product is thus autologous. This removes the risk of allogeneic reactions.

The risk that the paramagnetic bead component of TC0301 is immunogenic to an extent which could cause toxicity is considered low. During the final stages of pTTL production, the

TC0301 beads are removed and only trace amounts could be present at infusion. Even if disregarding the removal of TC0301, a non-clinical acute toxicity study in rats has not indicated that inflammatory responses to the TC0301 beads are evoked *in vivo* at doses that are considerably higher than those applicable to the pTTL product.

Unwanted immunogenicity also includes allergic reactions directed to constituents of the product. The starting material is collected and transported in cell culture medium containing antibiotics. The antibiotics are gradually diluted during pTTL manufacturing, by replacement with medium without antibiotics. The formulated product may still contain trace amounts of antibiotics after washing during formulation. Allergic reactions to the minute amounts of antibiotic substances cannot be excluded, but the risk is small. In patients with known allergy, a risk assessment will be made, and if the risk is considered manageable, pre-medication will be given and increased vigilance regarding allergic reactions at infusion will be observed. The excipients in pTTL include the cryopreservation medium Cryostor® CS10 (hereafter referred to as CS10). Ingredients in this proprietary solution includes dimethyl sulfoxide (DMSO), a substance with known toxicity. Allergic reactions are, however, unlikely to the ingredients of CS10 (see also section 6.6.2.5 below).

Lastly, the cell debris generated during pTTL manufacture, that to some extent remain in the final product, could theoretically elicit autoimmune responses. Cell debris is formed during pTTL production through the death of cells present in the starting material: non-T cells (NK cells, B cells, APCs) and T cells not specific to neoantigens, and thus not activated by TC0301 stimulation. Some debris may also be caused by the cryopreservation and thaw of the pTTL product. Debris from damaged and dying cells can cause significant immune activation and inflammation through binding of receptors against intracellular components. This is a normal process in which several intrinsic systems, including phagocytosis by immune cells, the liver's reticuloendothelial system, DNases, the actin scavenger system, and the coagulation system, participate in the clearance of cell debris. In extreme situations such as major trauma or burns, the large amounts of cell debris generated can promote the extensive inflammation associated with these injuries. In situations with limited damage, however, inflammation is controlled. Exposure to cell debris does not normally cause autoimmunity. (Schuermans et al., 2024). However, defects in debris clearance have been linked to autoimmune diseases such as systemic lupus erythematosus (Muñoz et al., 2010), and theoretically, cell debris could cause presentation of normal self-antigens by immune cells, which could confer an increased risk of autoimmunity. Further implications of cell debris in the product are discussed in Section 6.6.2.5 "Toxicity and off-target reactions".

6.6.2.3 Treatment failure

Treatment failure can be divided into 2 separate entities:

1. Production failure, meaning the failure of producing and releasing an acceptable pTTL product
2. Therapeutic failure, when the pTTL unit is administered but fails to elicit the expected effect.

Production failure is a potential risk in any manufacturing process and is particularly relevant in the production of cell-based products. The personalised nature of pTTL contributes to the uncertainties of the process, as efficient expansion of tumour-specific T cells depends on the size and fitness of the tumour-specific pre-existing T cell clones in the starting material, the availability of targetable neoantigens and the correct selection of such targets.

Suboptimal selection of neoantigens for TC0301 may lead to poor expansion of T cells specific for clinically relevant neoantigens and may ultimately cause treatment failure, either by insufficient T cell expansion during pTTL production or through failure to evoke a clinical response. The PIOR® Manufacturing system for selecting mutations and designing neoantigens is optimised for finding the best neoantigen targets according to currently available knowledge. A range of up to 36 neoantigens are targeted in order to optimise the chance of covering the most relevant neoantigens.

Correct timing of pTTL harvest is crucial to promote manufacture of an optimal product and thus potentially also to prevent treatment failure. Due to individual differences in T cell expansion kinetics, the time point when it is optimal to prepare the cell product in terms of number of T cells and T cell phenotype (non-exhausted and with the lowest potential risk of activation-induced cell death), may vary. Most pTTL products have a proliferation kinetic where harvest after 15 days of manufacture is considered optimal, but as there are products with an earlier proliferation peak, an alternative time point for product preparation after 12 days of culture is included in the production protocol.

pTTL is cryopreserved and thawed just prior to administration. This procedure enables flexibility with regard to administration, and quality assessments can be performed before use of the product. There is, however, a small risk of adverse effects to the cell product associated with cryopreservation, storage at ultra-low temperature, and thaw. Pre-clinical development has shown that the product is not adversely affected by cryopreservation, but unforeseen occurrences such as freezer failure adds a small additional risk which would affect any cryopreserved product.

Patient- and disease-related factors might also influence the chance of obtaining treatment response. Cancer type, anatomical location of lesions, tumour-burden and pace of disease progression might all affect the chances of treatment response. However, as pTTL is a previously untried ATIMP, the relative impact of different patient and disease characteristics are difficult to predict. Therefore, all patients with stage IV CRC for whom standard of care therapies are not available or suitable (and who fulfil the entry criteria) will be eligible for participation in this first pTTL trial. Treatment with pTTL may be more beneficial to certain subgroups, but clinical data are needed to determine which patient groups would benefit most from treatment with pTTL.

If the patient is on a treatment that has a toxic effect on T cells, this may hamper the pTTL treatment. Instructions on what usage of immunosuppressive drugs is allowed in order to prevent drug-mediated inhibition of pTTL efficacy are found in the entry criteria (Sections 9.4 and 9.5) and in the trial assessment and procedures section. This applies both for starting material harvest at entry in trial Part I and at pTTL treatment and in response to AEs in Part II.

6.6.2.4 Transmission of infectious organisms

The starting material of pTTL is derived from RLNs close to the colon, which could confer a risk of bacterial contamination with gut flora. However, measures to avoid contamination are applied at node harvest. When RLNs are harvested at standard surgery, or in case of study specific bowel resection, the excised section of bowel will be clamped at both ends to prevent bowel content from leaving the lumen, and RLN are excised before the tumour, and thus the bowel lumen, is dissected. In trial specific surgery by laparoscopic or minimally invasive lymphadenectomy, bowel incision is generally not included in the surgical procedure and

RLNs are approached from the abdominal cavity. The harvested material undergoes bioburden testing and heavily contaminated material is disqualified as starting material.

pTTL release testing includes sterility, mycoplasma and endotoxin testing. Sampling for these tests is done at harvest, prior to cryopreservation. Interim results from microbial testing are available after 3 to 4 days and are used for preliminary release of the product. Final test results of sterility, which can take up to 14 days to deliver, are required for pTTL release and for starting the treatment, while pre-conditioning chemotherapy may be initiated based on preliminary release data. The pTTL manufacturing process is performed in aseptic conditions using sterile reagents. The risk of transmitting microbial organisms is thus considered minimal and is further minimised with available measures. The risk that other disease-transmitting agents from the starting material will cause disease upon pTTL transfusion is also minimal, as the starting material is obtained from autologous tissue.

6.6.2.5 Toxicity and off-target reactions

Treatment-associated toxicity includes adverse reactions (ARs) associated to the MoA of pTTL and toxicities caused by impurities in the product. Toxicity could occur both in direct association with administration of the therapy and when some time has elapsed.

Impurities

Impurities in the pTTL context include any substance or cell in the formulated product that was introduced or generated during the manufacturing process and not considered part of the active ingredient of the product: immunostimulatory molecules (cytokines), cell debris, antibiotics, TC0301 and other cells than T cells are all considered impurities. Allergic reactions to trace amounts of antibiotics in pTTL are discussed above (Section 6.6.2.2, “Unwanted immunogenicity”).

The cytokine IL-2, which is used in pTTL manufacture, can cause dramatic systemic reactions. However, the IL-2 formulation used here is a pharmaceutical grade drug for human use that is considered safe with proper safeguards in far larger amounts than what is applicable in this setting, especially as the IL-2 is removed with the culture medium during product formulation. The cells in pTTL can also produce cytokines while stored after formulation. This has been described for standard blood products and is known to induce acute transfusion reactions such as fever. Strategies to manage acute ARs caused by remaining IL-2 or other cytokines will be in place in analogy with the precautions used with blood product transfusion, and reactions are expected to be well manageable (Tinegate et al. 2012).

Cell debris is formed during pTTL manufacturing and processing. A large proportion of the remaining cells (such as B cells, non-responding T cells, APCs) die, and aggregates of debris are commonly seen during pTTL manufacturing. Cryopreservation and thaw might increase debris formation. Larger particulates and/or large amounts of debris may constitute a risk for embolism and other adverse events. Filtration and magnetic TC0301 bead removal performed during formulation removes any visible particulates, and larger debris remaining or formed during storage of the product is removed by an in-line filter in the transfusion aggregate, preventing entry into the circulation. Smaller, non-sticky particles may pass through the filter, however. The clinical risk of these smaller particles is less well-defined than with larger aggregates, but cell debris is known to have immunostimulatory properties, as discussed in more detail in Section 6.6.2.2 “Unwanted immunogenicity”. However, except under special circumstances, cell debris neither cause serious non-specific inflammation on a systemic level nor autoimmunity (Schuermans et al, 2024; Muñoz et al, 2010). Based on clinical

observations risks are considered to be small: described reactions have been manageable (Watz et al. 2015; Zambelli et al. 1998).

Trace amounts of antibiotics (streptomycin sulphate and gentamicin sulphate) may be present in pTTL. As discussed above (Unwanted immunogenicity) this is a risk factor for patients allergic to antibiotics. A mitigation plan has been put in place for allergic patients.

The raw material TC0301 is removed before product formulation. Only residual amounts of TC0301 beads may remain as impurities in pTTL. These are unlikely to cause toxicity, as the number of beads is kept well below the evaluated safe dose as determined in a good laboratory practice (GLP) acute toxicity study in rodents (see Section 6.3).

Non-T cell leukocytes normally present in the RLNs used as starting material for pTTL manufacturing can be present in the pTTL product, as seen during pre-clinical characterisation. These non-T cells, that are considered as impurities in pTTL, are of autologous origin. The most commonly detected non-T cell type in pTTL is natural killer (NK) cells. These selectively recognise malignantly transformed or infected cells and the risk of damage to normal tissue is judged to be minimal. NK cell-based therapies have been conducted with limited safety concerns (Lamb et al. 2021). A smaller proportion of B cells has also been observed in pTTL. The B-cells are not selectively manipulated during the culture process. Thus, the non-T cells present in pTTL are not regarded as a safety concern.

Adverse reactions associated with the proposed MoA of pTTL

ARs associated to the product’s predicted MoA are yet unknown as the product has never been used in humans. However, based on knowledge from previous studies of similar products, certain risks have been identified as potential risks in the pTTL context. They can be divided into acute infusion reactions and reactions associated with the functional response of infused T cells. In general, as these side effects could be associated with pTTL, strategies for their management have been put in place.

Potential adverse reactions associated with the functional response of pTTL cells

One potential side effect is CRS. This side effect is considered to be triggered by a massive synchronous T cell activation causing extensive cytokine production and subsequent activation of other immune cells. This causes further pro-inflammatory mediator production and initiation of inflammatory cascades leading to systemic toxicity (Sheth and Gauthier 2021; Yáñez, Sánchez-Escamilla, and Perales 2019). This toxicity has been primarily associated with CAR T cell therapy to date but has occurred also in transgenic TCR T cell therapy (Ishiara et al. 2022, Parkhurst et al. 2024) and with bispecific antibodies such as blinatumomab (Adkins 2019; Jain and Litzow 2020). CRS is generally not observed after TIL therapy (Betof Warner et al., 2024). It has however been reported in a few cases for neoantigen targeted TIL products like cNeT (Turajlic et al. 2021).

CAR T cell therapy and transgenic TCR T cell products differ from pTTL in some important respects. The T cells in these ATMPs all express the same targeting receptor and are thus directed against a single antigen target with the same (generally high) affinity. The tumour-reactive population in pTTL will be composed of several clones with different specificities and affinities, and although the number of cells in CAR T cell products and pTTL products are in the same range, the former will contain a large proportion of cells with potential to respond rapidly and simultaneously toward the same target, while such a large-scale response is unlikely with an oligoclonal pTTL product. This supposition is supported by the fact that CRS generally is not seen after conventional TIL therapy, and have been reported in only a few cases after neoantigen-stimulated TILs, as mentioned above. Despite the assessment of a

low risk, however, it cannot be excluded that pTTL could trigger CRS. The results from cNeT therapy (Achilles Therapeutics®) confirms that neoantigen-targeting genetically unmanipulated T cell products could be associated with CRS. Thus, pre-cautions, including availability of tocilizumab, will be taken to ensure correct management in the case that such an AE should occur. Further information about the management of adverse events can be found in the "Management of adverse event manual" appended to this protocol.

Other severe complications associated to CAR T cell therapy which could be relevant also in this setting include neurotoxicity, termed ICANS or CAR T-related encephalopathy syndrome (CRES; Jain and Litzow 2020; Sheth and Gauthier 2021). ICANS is generally not observed after TIL therapy (Betof Warner et al., 2024). An association between this type of side effect and CRS has been described, and the risk of this type of toxicity should, using the same arguments as above, be low but not negligible, and awareness and strategies for management needs to be in place.

Tumour lysis syndrome (TLS), an AE that can occur as a result of any type of rapidly working and effective therapy that causes large-scale destruction of cancer cells, could also be a risk in the pTTL context. The release of large amounts of cancer cell material into the bloodstream leads to a risk of severe organ toxicity, including damage to kidneys, the central nervous system (CNS) and the heart, and can even lead to sudden death (Jain and Litzow 2020). The risk for TLS is considered less for the oligo-clonal product pTTL than for example CAR T, but awareness that they may occur and measures for their management is necessary. The administration of allopurinol prophylactically will be applied in patients with high risk of TLS.

Another class of toxicity which could potentially be associated with pTTL is autoimmunity, or off-target toxicity. In TIL therapy, autoimmune reactions have been described (Andersen et al. 2016; Besser et al. 2020; Dudley et al. 2005, 2008; Goff et al. 2016). In TIL-treated patients with malignant melanoma, autoimmune reactions in the form of vitiligo and uveitis have been described. These disorders result from immune-mediated attack on melanocytes, the healthy counterpart of melanoma cells. In a meta-analysis of 280 treated patients pooled from 8 studies, vitiligo and uveitis occurred in 5.7% and 2.1%, respectively (Dafni et al. 2019). This type of autoimmune toxicity could hypothetically occur with pTTL as result from cross-recognition of normal counterparts to the targeted neoantigens in TC0301 NAG or of unrelated proteins that by chance contain a similar epitope. The tissue from which the treated tumour originated is a likely target for autoimmune toxicity, which in this trial would be the colon. In consequence, autoimmune colitis could be a potential side-effect of pTTL, but other tissues could also be affected. However, in trials of the SLN-based therapy developed by Prof O Winqvist and colleagues, no autoimmune toxicity or other AEs were observed in patients with CRC (Karlsson et al. 2010; Zhen et al. 2015) or UBC. One case of vitiligo was observed in a patient with melanoma in line with the observations in TIL therapy (unpublished material, personal communication Prof. O Winqvist). However, this therapy was administered as a monotherapy, without pre-conditioning, and the means of obtaining tumour specificity differ between the cell products (see Section 6.4 above).

The use of autologous mature T cells which have developed in the patient in the presence of tolerogenic mechanisms (thymic selection, peripheral tolerance and other mechanisms that reduces the risk of autoimmunity *in vivo*) and without the risk of allogeneic recognition should reduce the risk of autoimmune AEs. That no genetic manipulation of the TCR or the regulation of T cell activity is applied to pTTL T cells is expected to further reduce the risk, and possibly also the severity, of autoimmune reactions and hyperinflammatory responses. The target epitope and binding affinity of the TCR is the same as when the T cells were

harvested, and the cells will respond normally to anti-inflammatory signalling. This will not preclude autoimmunity, as unmanipulated immune cells can respond against self-antigens to cause autoimmune disease, but the risks seen in genetically manipulated T cell immunotherapy against a set target, such as unexpected cross-reactivity and expression of T cell epitopes in non-malignant tissues, are not expected here.

However, since this type of side-effect is unpredictable in terms of time of onset after therapy and could be severe, special vigilance for this type of event will be exercised during follow-up. All new clinical symptoms and abnormal laboratory findings that could indicate autoimmune organ toxicity will be considered carefully and further investigated as applicable, and early immunosuppressive therapy initiated when indicated. Some preventive measures are also taken to reduce the risk of pTTL off-target toxicity. Firstly, the selection of neoantigens by PIOR® Manufacturing takes into consideration that the neoantigen-causing mutations are not present in normal somatic cells to reduce the risk of pTTL recognition of normal tissue. Secondly, patient selection will take the risk of autoimmunity into account. Patients with underlying autoimmune conditions or other disorders affecting the immune system, such as immune defects, will be carefully evaluated to assess their potential risks before inclusion in the trial.

Acute infusion reactions

Acute infusion reaction is a known risk of therapy with cell-based products, including standard blood components such as erythrocyte concentrates. There are several causes for such reactions, including infusion of pro-inflammatory cytokines contained in the cell product and allergic reactions. In the TIL setting, reported acute infusion reactions have been of a mild to moderate severity and well manageable, and have included transient fever, chills, hypertonia, tachycardia, hypoxia, dyspnoea and nausea (Andersen et al. 2016; Besser et al. 2020; Dafni et al. 2019; Dudley et al. 2005, 2008; Ellebaek et al. 2012; Goff et al. 2016). The frequency varied between studies. The lymph node-based therapy developed by Prof. Winqvist and colleagues was associated with few and mild acute reactions (Karlsson et al. 2010; Sherif et al. 2015; Zhen et al. 2015 and unpublished material, personal communication Prof. O Winqvist).

DMSO, a substance in CS10, which is an excipient of pTTL used to enable cryopreservation, and which remains in the product after thaw, is known to cause toxicity. This substance is extensively used in cryopreservation of cells, including stem cell grafts, and is in many cases given to the patient with the product, as washing it away is associated with a risk of reducing the cell dose (Hornberger et al. 2019). DMSO toxicity is considered dose dependent, and reports have included mild, moderate and severe, even life-threatening, adverse effects. Common, mild to moderate side effects of DMSO infusion includes nausea, vomiting, and headache. More severe side effects include hypo- and hypertension, hypoxia and respiratory distress, cardiac arrest, encephalopathy, transient global amnesia, epileptic seizures, and shock (Cox et al. 2012, Mitrus et al. 2018). The amount of DMSO infused in this trial will be small compared to what is used with stem cell grafts, 1-5 ml pure DMSO (5%, calculated on product size 10 - 100 ml) compared to approximately 20 – 40 ml in stem cell grafts (5-10% in approximately 400 ml) (Cox et al. 2012, Mitrus et al. 2018). The low dose used, and the fact that the specific excipient CS10 is used in several marketed CAR T cell products (Yescarta™, Breyanzi™ and Tecartus™, see European public assessment reports [EPAR] product information) at, in two of the products, similar amounts to what is used with pTTL, would argue that the use is safe, even though awareness of potential side effects and preparedness to manage these are important.

Safety profiles of tumour-targeted T cell products

Safety data from T cell ACTs, that similar to pTTL selectively target neoantigens, are available from early phase clinical trials. cNeT (Achilles Therapeutics), which is a neoantigen-targeting TIL product, has been found to have an acceptable safety profile, comparable to standard TIL therapy, in patients treated in two phase I/II studies in malignant melanoma and non-small cell lung cancer (Achilles Therapeutics Press Release April 4 2024). Three cases of CRS grades 1-2 have however been reported, and two cases of neurological toxicity of which one was classified as ICANS/CRS and potentially associated with the cell product (Turajlic et al. 2021). Immatics® published results from 14 patients with their tumour-associated antigen-targeting product, and adverse events included side effects of lymphodepletion and IL-2 and low-grade CRS (Tsimberidou et al. 2020). No autoimmune reactions were reported for either of the therapies. Hypothetically, the risk of autoimmune off-target effects could be smaller when targeting specifically designed neoantigens derived from mutations in the individual tumour than when applying non-specific stimulation like anti-CD3 or using a tumour extract including a considerable amount of unaltered self-proteins for the production of the T cell product.

Conclusion

The observations from similar T cell ACTs indicate that pTTL can be expected to have a favourable safety profile with a relatively low risk of severe responses regarding all the types of toxicity discussed. However, the risk for side effects cannot be entirely predicted based on other ACTs and precautions and preparations to meet AEs will be in place for the trial.

6.6.2.6 Risks associated with trial-related procedures

Some risks associated with trial participation are linked to trial-related procedures, including trial specific surgery, collection of peripheral blood samples, and tumour biopsies.

RLN starting material for pTTL manufacture can only be obtained through extirpation of RLNs during abdominal surgery. Two alternative options are available for the target patient group in this trial: non-curative standard care surgery, when such is planned in an opportune fashion for trial purposes, or trial specific surgery. Either procedure includes general anaesthesia, which confers some specific risks besides the actual surgery (Bainbridge et al. 2012). All patients will undergo an anaesthesiologic evaluation to ensure that the patient is eligible for operative procedures under anaesthesia. The surgical procedure will be associated with the standard risks associated with intra-abdominal surgery, such as intra- and post-operative haemorrhage, infection, and thrombosis. When RLN collection is performed at surgery that is part of standard care, the risks are not increased by trial participation, while the risks are entirely trial-related when trial specific surgery for RLN retrieval is performed.

Trial specific surgery is performed exclusively for that purpose, and the procedure will be limited in scope to what is necessary to obtain satisfactory starting material. The surgical technique is selected to result in minimally invasive surgery based on the characteristics of the patient and disease, taking into account factors like previous surgery and the localisation of lymph nodes identified by pre-surgery radiology. The procedure is required to entail limited convalescence and risk of complications. The surgical methods for trial specific surgery include laparoscopic or minimally invasive lymphadenectomy, and laparoscopic or minimally invasive resection of the section of bowel where the primary tumour is localised. Conversion from a laparoscopic procedure to a laparotomy is considered in cases where the identification of lymph nodes is found to be difficult at lymphadenectomy, or where indicated due to medical considerations.

Minimally invasive colorectal resection is considered to be associated with a higher risk of complications than minimally invasive lymphadenectomy, and with a longer convalescence, but is judged to have the highest chance of collecting satisfactory starting material. In general, abdominal surgery comes with a risk of complications, including postoperative infection, bleeding, thromboembolism, and ileus. The relative risk for each complication between the surgical methods is difficult to predict, but bowel resection is expected to confer a higher risk of infection (Scarborough et al. 2017, Pak et al. 2020). Bowel resection will result in the establishment of a stoma in patients without a pre-existing stoma, as bowel anastomosis will generally be avoided, and may only be considered in exceptional cases at the discretion of the Investigator, to reduce risks. This also entails a few days longer convalescence and in-patient care. Arguments for bowel resection in selected cases include, besides an improved chance of collecting satisfactory starting material, that such a procedure might have psychological benefits for certain patients, and that such surgery might be necessary in some cases at a future time point. The choice of surgical method will be made taking these factors in consideration, together with the characteristics and specific circumstances of each patient. When bowel resection is considered, the internal safety review committee (iSRC), supplemented by an independent medical expert and expert surgeon(s), will assess if bowel resection is considered acceptable from an individual risk/benefit perspective, see Section 8.4.3. A recommendation for the procedure from the committee is required before the surgery may be performed. The removal of asymptomatic primary tumours in patients with colorectal cancer stage IV and non-resectable metastases has been shown to have neither a positive, nor a negative impact on outcome and overall total survival (Anwar et al. 2021, Kanemitsu et al. 2021). Anastomosis may only be considered in exceptional cases after careful individual evaluation of the risks for anastomotic leakage and potential delay or prevention of further oncological treatment caused by possible complications (Sciuto et al. 2018, Zarnescu et al. 2021).

All patients undergoing abdominal surgery routinely receive prophylactic antibiotic treatment. Patients considered to have substantial risks at surgery and anaesthesia in general should not be included in the trial, and neither should patients for which the procedure required to obtain RLNs is considered a high-risk surgery. This would apply to, for example, patients that have undergone surgery previously in the same anatomical area that would now need to be targeted. This means that patients that have previously undergone surgery to remove the primary tumour in the colon cannot be candidates for inclusion in the present trial due to the complicated nature of the required surgical procedure to obtain RLNs in these patients. The convalescence time for the projected trial specific surgery will occur in parallel with TC0301 production and will not delay pTTL administration unless severe complications should occur.

To ensure that trial specific surgery is limited to patients for whom there is a high likelihood of successfully obtaining acceptable RLN starting material, pre-operative radiological examination must show identifiable RLNs accessible to surgery and should guide the choice of surgical approach. This is expected to reduce the risk of unsuccessful surgery, and to guide the surgery to the correct area, limiting the operation, both the procedure extent and surgery time. If sufficient current radiological examination is lacking, or if the quality of the available examinations is insufficient to provide pertinent information, an additional, trial specific examination can be performed.

Radiological examination is part of standard evaluation of patients in the target patient group, but trial specific imaging could be necessary, as discussed above. Radiological examinations will also be performed more frequently during trial follow up than in standard care: patients in the trial will undergo radiological examination every 6th week during Part II compared to approximately every 8th week in standard care. The radiation of computer tomography, the

most common type of radiological imaging which will be used, will confer some additional radiation. For trial specific examinations performed to gain pre-surgery information, the associated risks of one extra examination are considered small compared to the unnecessary risks conferred by unsuccessful, and thus unnecessary, surgery. The increased radiation conferred by slightly increased examination frequency during Part II is considered to be balanced by the importance of the information gained. Earlier detection of both potential complications and, in particular, of progression could be enabled. In the case of progression, the patient can seek other possible treatment alternatives. This is of importance in all patients, but especially those that are treated during a scheduled pause in treatment, for which the paused treatment can then commence without unnecessary delay.

Other trial procedures include the collection of peripheral blood samples and tumour biopsies. The collection of tumour tissue can be performed as well as RLN collection at standard care tumour surgery in the patients that are planned to undergo such an operation. The trial-related risks associated with the resection of tumour tissue and lymph nodes for trial purposes for these patients are judged to be very small. Standard histopathological evaluation is not performed on the RLN material used for pTTL manufacture. However, the patients in this trial have metastatic cancer without curative treatment options, and the risk of impaired detection of lymph node metastases is thus not going to materially affect diagnosis or assessed prognosis. For the patients who undergo trial specific surgery for RLN collection, tumour sampling for NGS analysis cannot be performed during this procedure, as an approved NGS analysis identifying tumour mutations is required prior to the study specific surgery. Instead, tumour samples will be obtained by biopsy, mostly endoscopic, of the primary tumour, or percutaneous ultrasound/radiology-guided biopsy of an accessible metastasis, which will also be a trial specific procedure. If the biopsy does not fulfil the quality requirements for extracting material for NGS analysis, an additional biopsy may, at the discretion of the Investigator, be performed to obtain material for the analysis. Trial specific surgery for retrieval of RLNs will not be performed unless the NGS analysis render a sufficient number of tumour mutations for starting the production of TC0301. Endoscopy and biopsy are associated with risks, such as bleeding and post-procedure pain. There is a small risk of tumour spreading along the biopsy channel. However, procurement of biopsy, endoscopic and percutaneous, are standard procedure in clinical diagnosis, and the risks are well-known and have standardized mitigations and precautions. The procedures will be performed by hospital staff with experience of the procedure, and the risks are considered very small.

The collection of trial samples for explorative biomarker analyses can be associated with some risk, mainly in the case of biopsies. The risks are the same as described above with biopsy for NGS analysis. No biopsy will be collected in cases where the risks of complications are judged to be substantial. However, the information that can be obtained by study of biopsy material before and after pTTL therapy could be invaluable for biomarker evaluation of pTTL tumour infiltration, and biopsies are recommended when judged to be safe.

Blood samples will be collected as reference material for NGS analysis at surgery or in connection with biopsy. Trial specific blood samples will also be drawn during Part II and Part III of the trial. The risks associated with peripheral blood sample collection are minimal.

Three days pre-conditioning treatment will be given to the patients between Day -5 and Day -3. The side-effects of the combination of cytostatic drugs of the pre-conditioning treatment, cyclophosphamide at a dose of 300 mg/m^2 body surface area (BSA) and fludarabine 30 mg/m^2 BSA for three consecutive days, are well documented, manageable, and mitigations are available. Pre-conditioning chemotherapy should only be initiated when a

pTTL product has been produced and cryopreserved and preliminary release test results are available, to minimise the risk of pTTL production failure.

Patients will remain in in-patient care for at least one week after pTTL administration (until Day 8) and will be closely monitored by medical staff. The patients will also have scheduled follow-ups at the Recruitment, Surgery & Treatment Site during the 26 weeks that follows pTTL infusion. Visits to the Recruitment, Surgery & Treatment Site may be prolonged in case the Investigator finds it medically warranted for safety reasons.

An iSRC will monitor emerging safety data over the course of the trial, see Section 8.4.2 and Section 8.4.3 for stopping criteria and for further information about the iSRC.

Overdosing is not a significant concern with pTTL since the products are formulated at production in single doses, to be administered in their entirety. The dose is limited by pTTL specifications. The risk that a larger dose than allowed by specification would be formulated, e.g. if cell counting errors occur at harvest and formulation, is minimal. However, as much larger doses are used in other autologous T cell products than the highest allowed pTTL dose, the risks in such a scenario are considered small. pTTL will be administered by site personnel under medical surveillance and the patients are carefully monitored for adverse reactions. For further information regarding overdosing, refer to Section 11.3.1.20.

Each patient will be provided with a patient alert card with information about the patient’s participation in a clinical trial, see Section 14.4.

The Coordinating/Principal Investigator at the Recruitment, Surgery & Treatment Site will ascertain that adequate facilities and procedures are available to handle emergency situations, should they occur during Part II of the trial. The medical staff at the Recruitment, Surgery & Treatment Site have extensive experience from early Phase I and FIH studies and there are adequate procedures in place to handle unexpected and expected ARs in the trial patients. ME CAST, handling pTTL administration and in-patient care and monitoring, is located in the same building as the Intensive Care Unit (ICU) of the Karolinska University hospital, Huddinge, Stockholm. ME CAST performs standard of care stem cell transplantation and CAR-T cell therapy as their main procedure. Patients undergoing therapy here during participation in a clinical trial have the same access to ICU care, should such be necessary, as patients undergoing standard health care procedures.

6.6.2.7 Risk assessment in relation to Covid-19

Current recommendations from the authorities will be considered on a day-to-day basis and a continuous risk evaluation will be made to assess how the Covid-19 situation may affect the study conduct, quality/integrity of data and the safety of the trial subjects. The risks and mitigating actions are documented in a risk log as part of the Sponsor’s trial master file (TMF).

European Medicines Agency’s (EMAs) Guidance on the Management of Clinical Trials during the Covid-19 (SARS-CoV-2) pandemic as well as local guidelines from the Swedish Medical Products Agency (MPA) will be taken into consideration.

Testing of patients for SARS-CoV-2, in addition to the tests outlined in Section 8.1, can be performed at any stage of Part I and Part II at the discretion of the Investigator if it is deemed indicated for the safety reasons. No ATIMP will be administered to patients with a suspected or confirmed ongoing SARS-CoV-2 infection. Patients who cannot receive pTTL due to SARS-CoV-2 infection may receive the treatment at a later time point when the infection has resolved.

6.6.3 *Final risk/benefit assessment*

The patients eligible for this trial are patients with advanced malignancy with few or no therapeutic options available apart from participation in a clinical trial. All participants have a poor long-term prognosis with no therapeutic options with a chance of cure available. Based on similar T cell products, the safety profile of pTTL is anticipated to be positive and therefore justify the potential risks incurred by its use in this category of patients with advanced Stage IV CRC. Precautions are expected to meet the requirements for the compiled anticipated safety profile, but also to provide preparedness for severe complications.

Adoptive T cell therapies have the potential for major and long-lasting efficacy as they aim to harness the immune system's capacity to first mount an immune response which can eradicate a perceived threat, the cancer cells, and then to develop immunological memory, preventing cancer recurrence by recognition and eradication of new cancer cells that are formed.

As this is the first time that this ATIMP is being tested in humans, prospective trial patients will be made aware of the potential risks that have been identified based on previous similar T cell immunotherapies and information on observed toxicities will be provided. Information regarding the risks of trial specific procedures, including, when applicable, trial specific surgery, will also be provided to ensure an informed decision. They will also be informed that only theoretical arguments can be made about the therapy's advantages.

In summary, it is concluded that the potential benefits justify the potential risks incurred by pTTL treatment of advanced CRC.

More information about the risk-benefit assessment, justification of risks and risk management per category are provided in the current version of the IB.

7 TRIAL OBJECTIVES AND ENDPOINTS

The objectives, endpoints and associated assessments are summarised Table 3. Exploratory endpoints may be reported separately from the clinical trial report.

Table 3 Objectives and endpoints

**Objective**		**Endpoint**	**Assessment(s)**
Primary	To establish that pTTL can be administered to patients with advanced CRC without unacceptable toxicity	The primary endpoint is safety (with AEs assessed according to Common terminology criteria for AEs [CTCAE] v5.0). Special focus will be placed on immunological AEs and AEs known to be associated with T cell therapies, which are classified as AEs of special interest (AESI). AESI will include autoimmune reactions potentially resulting from off-target toxicity such as colitis, and immune-mediated reactions associated with immune cell activation, such as CRS.	AE reporting and questioning (Section 11.3.1), physical examinations, vital signs, safety laboratory parameters and electrocardiogram (ECG) (Sections 11.3.2 to 11.3.5)
Secondary	To evaluate treatment efficacy in terms of objective response.	Objective response (according to iRECIST).	Medical imaging and iRECIST (Section 11.2.6)
		Characterisation of treatment response:<br>• time to response<br>• duration of response	Medical imaging and iRECIST (Section 11.2.6)
		Time to tumour progression assessed using the iRECIST criteria (time from inclusion in Part II of the trial until disease progression).	Medical imaging and iRECIST (Section 11.2.6)
		Kinetics of tumour progression/growth (compared to pre-treatment)	Medical imaging and iRECIST (Section 11.2.6)
	To evaluate treatment efficacy in terms of progression free survival and overall survival.	Overall survival (time from inclusion in Part II to death of any cause).	
		Progression-free survival (time from inclusion in Part II to tumour progression or death of any cause).	Medical imaging and iRECIST (Section 11.2.6)
		Disease-specific survival (time from inclusion in Part II to death of disease).	
Exploratory	To explore biomarkers for pTTL persistence *in vivo*.	Evaluation of biomarkers for pTTL persistence. TCR sequencing (TCRseq) will be used to characterise the pTTL product and to trace the identified pTTL clones in sequential peripheral blood samples post treatment.	Biomarker sampling (Section 11.2.5)
	To explore biomarkers for anatomical localisation of pTTL post infusion, with focus on tumour infiltration.	Evaluation of biomarkers for pTTL tumour infiltration. If feasible, pre- and post-treatment biopsies will be taken for histological assessment of T cell infiltration and characterisation of tumour-infiltrating T cells. Characterisation is performed if possible based on the amount of	Biomarker sampling (Section 11.2.5)

Objective		Endpoint	Assessment(s)
		material obtained, and could include TCRseq analysis (to identify pTTL clones) and characterisation of T cells properties.	
	To explore biomarkers for pTTL recognition of neoantigens *in vivo* and for pTTL capacity to achieve tumour cell eradication.	Evaluation of biomarkers for pTTL neoantigen specificity and efficacy:<br>• Functional T cell assays will be used to evaluate neoantigen-specific responses in the pTTL product and in peripheral blood T cells.<br>• Markers of tumour burden will be evaluated. This may include analysis of circulating tumour DNA (ctDNA) and serum tumour markers in peripheral blood, and will constitute a complement to iRECIST evaluations	Biomarker sampling (Section 11.2.5)
	To explore biomarkers for pTTL characteristics.	Analysis of the pTTL product and of post therapy peripheral blood samples will be performed to evaluate the functional status, differentiation and lineage-identity of T cells and other immune cells.	Biomarker sampling (Section 11.2.5)
	To gain early insights in selection of neoantigens. Data on the association between the selected neoantigens, biomarker evaluation and clinical outcome will be collected.	Storage of data relevant for the correlation of neoantigen selection to clinical outcome. The type of neoantigens targeted will be assessed in the context of clinical outcomes, signals of efficacy and biomarker evaluation.	

8 TRIAL DESIGN

8.1 Overall trial design and schedule of events

This FIH trial is composed of 3 parts, referred to as Part I, Part II and Part III.

Part I of the trial entails patient identification, recruitment and screening of trial patients. It also comprises production of pTTL, which is performed in 3 steps:

- Collection of tumour samples and normal samples for NGS and neoantigen selection. Production of stimulatory NAG-coupled beads raw material TC0301.
- Collection of starting material for pTTL manufacturing from RLNs.
- pTTL manufacturing.

Further details on Part I are provided in Section 8.1.1.

Part II of the trial entails pre-conditioning and supportive care therapy, treatment with pTTL and subsequent 26 weeks safety follow-up. Further details on Part II are provided in Section 8.1.2.

Part III of the trial is a long-term follow-up initiated after completion of Part II. The long-term follow-up will continue for 4.5 years or until death, whichever comes first. Further details on Part III are provided in Section 8.1.3

An overview of the trial procedures is presented in Figure 3. The estimated recruitment time to complete inclusion of trial patients is 24 months. The total estimated time to complete Part I and Part II of the trial is 36 months, including active recruitment and pTTL treatment during approximately 30 months and 6 months follow-up of the last included patient.

Figure 3 Overview of trial procedures (Part I, Part II and Part III)

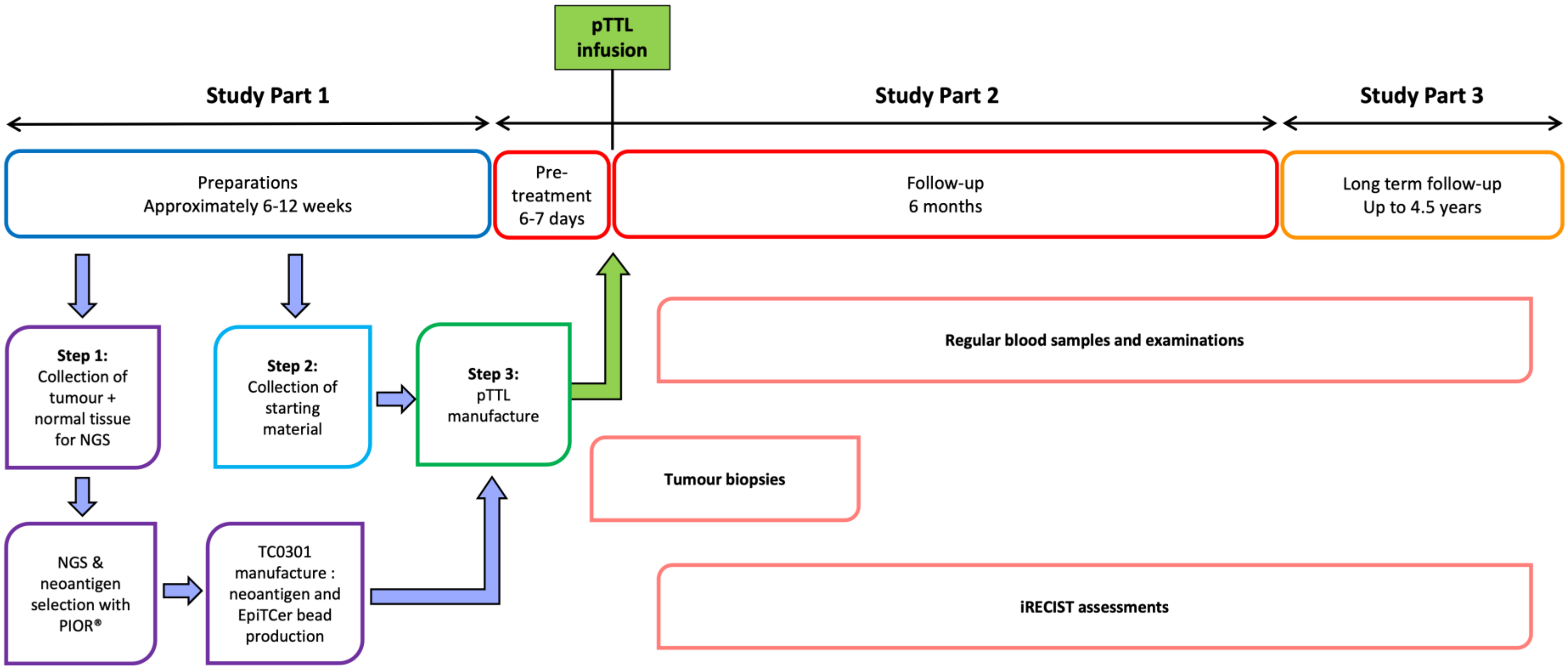

NGS= next generation sequencing, pTTL=personalised tumour trained lymphocytes, TC0301=neoantigen-coupled beads

The figure displays the timeline of trial procedures in Part I, II and III including pTTL production steps. The collection of tissue for step 1 and step 2 in Part I can be performed during the same surgery or at separate occasions according to what is most suitable for each included patient. Regular blood sampling and examinations are performed throughout the trial (Part I, II and III). Pre-treatment (pre-conditioning and supportive care treatment) is detailed in Figure 4.

8.1.1 *Part I: collection of tumour samples for next generation sequencing and starting material for pTTL manufacturing*

Part I comprises 2 visits. Visit 1, screening, is performed at one of the Recruitment & Surgery Sites (local patients) and/or or at the Recruitment, Surgery & Treatment Site (all patients referred from Referral Sites or identified from the general referral flow of patients at the Recruitment, Surgery & Treatment Site). Visit 2, surgery, involves collection of tumour samples and normal samples for NGS and neoantigen selection and collection of starting material for pTTL manufacturing from RLNs as outlined below.

The production of pTTL is performed in 3 steps:

1. Collection of tumour samples and normal samples for NGS and neoantigen selection.

   Suitable tumour material can be obtained either from biopsies or from surgically excised tumour material. In association with the biopsy/surgery, a blood sample is obtained to use as a normal tissue control. Surgeries and biopsies are performed at one of the Recruitment & Surgery Sites or at the Recruitment, Surgery and Treatment Site.

2. Collection of starting material for pTTL manufacturing from RLNs.

   Step 1 and 2 can be performed at separate occasions or during the same surgery, depending on what best suits the clinical workflow in a specific patient case. The starting material will be cryopreserved to facilitate trial logistics and increase flexibility in the inclusion process. The surgery to obtain starting material is performed at the Recruitment, Surgery and Treatment Site or at one of Recruitment & Surgery Sites.

3. pTTL manufacturing.

   The pTTL starting material is transported to the selected tissue establishment centre and good manufacturing practice (GMP)-compliant manufacturing facility, Vecura, Karolinska University Hospital Huddinge, Stockholm, Sweden, where it is processed and cryopreserved for storage until a suitable time for pTTL manufacture. pTTL manufacture is initiated when personalised TC0301 production has been finalised, and the patient is judged to be eligible for Part II. The pTTL manufacturing process is described in Section 10.3 and Section 10.4.

The time required from collection of tumour material and RLNs up until pTTL production and administration to patient is approximately 8 to 12 weeks. The cryopreservation of the RLN starting material enables flexibility in the choice of pTTL administration time (as the pTTL production, which requires approximately 2 weeks, can be started at any suitable time after the production of TC0301 has been completed). For this reason, potential trial patients will be asked to give separate consents for Part I, Part II and Part III of the trial. The first consent is given for pre-screening activities in Part I, the second for tissue collection and pTTL production in Part I, and a third consent is given for pTTL treatment and subsequent follow-up in Part II/III. The first consents may, in some cases, take place while the patient has other non-curative therapeutic options but might become eligible for inclusion in Part II of the trial within approximately 6 to 24 months. For details on the informed consent process, see Section 14.3.

No formal inclusion limit is set for Part I of the trial in order to accommodate to the possibility that some of the included patients will be unable to continue to Part II due to e.g. disease progression. The minimum duration of participation in Part I (for patients proceeding directly to Part II) range between 9 and 15 weeks (the minimum time will vary according to patient-related and trial-related circumstances, and includes the time needed for collection of

tumour material and RLNs, and for pTTL production). However, as patients may be included in Part I before they are eligible to enter Part II, participation in Part I might be as long as approximately 24 months after collection of RLNs (both the starting material for pTTL production and the pTTL products are cryopreserved with shelf life of ≥1 year). Other factors, including the limitation in the frequency a new patient can receive pTTL therapy that is set to enable safety evaluation, could also affect the time patients participate in Part I. The trial assessments to be performed in Part I are shown in Table 4 and are detailed in Section 8.2.1 (Visit 1) and Section 8.2.2 (Visit 2). Details of biomarker sampling are given in Section 11.2.5.

Table 4 Schedule of events Part I

**Visit**		**Visit 1**	**Visit 2**
**Assessment**	**See CTP Section:**	**Screening**	**Surgery**
Informed Consent pre-screening Part I	9.2.1, 14.3	X[1]	
Informed Consent Part I	9.2.2, 14.3	X	
Inclusion/exclusion criteria	9.4	X[2]	
Demographics	11.1.3	X	
Weight/height/BMI	11.1.6	X	X[3]
Medical history	11.1.4	X	X
Serology	11.1.5	X	
Physical examination	11.3.2	X	X
Vital signs	11.3.3	X	X
12-lead safety ECG	11.3.5	X	X
Safety laboratory profile	11.3.4	X[4]	X
Urine analysis	11.3.4	X	
ECOG performance status	11.1.9	X	
Pregnancy test	11.1.8	X	
Spirometry (if indicated)	11.1.11	X[5]	X[5]
Echocardiogram	11.1.12	X[6]	X[6]
Pre-operative medical imaging (MRI or CT)	11.2.6	X[7]	
Patients scheduled for trial specific surgery - Collection of blood & tumour samples for NGS: tumour material is collected through biopsy	11.2.1<br>11.2.2	X[8]	
Patients scheduled for standard surgery - Collection of blood & tumour sample for NGS: surgically excised tumour material is collected in association with standard surgery	11.2.1<br>11.2.2		X[9]
Collection of starting material for pTTL production from RLNs	11.2.3		X[10]
Biomarkers (blood)	11.2.5.3	X[11]	
Biomarkers (pTTL)	11.2.5.1		X[12]
Biomarkers (tumour and normal tissue)	11.2.5.2		X[13]
AEs	11.3.1	X[14]	X[14]
Prior medications	11.1.7	------X-----	

AE=adverse event, BMI=body mass index, CT=Computed tomography, CTP=clinical trial protocol, ECOG=Eastern Cooperative Oncology Group, MRI=magnetic resonance imaging, NGS=next generation sequencing, RLN=regional lymph node

1. Informed consent for pre-screening activities: sharing of imaging data and medical records necessary to enable discussions regarding the patient's potential suitability for trial participation.
2. Patients scheduled for trial specific surgery: spirometry (if indicated) and echocardiogram should be performed at Visit 1 to assess exclusion criteria No 2 and No 3, respectively. Patients scheduled for standard surgery: spirometry (if indicated) and echocardiogram may be performed after Visit 1 and are not mandatory for inclusion in Part I due to time limitations prior to standard surgery.
3. Weight only at Visit 2.
4. If results from safety laboratory profile analyses (taken during standard care or performed for other purposes) not older than 1 week are available, these analyses are valid and new sampling is not required at the discretion of the Investigator.
5. Patients scheduled for trial specific surgery: a spirometry assessment should (if indicated) be performed at Visit 1 for assessment of exclusion criterion No 3 (Part I). Patients scheduled for standard surgery: spirometry (if indicated) may be performed after Visit 1 as judged by the Investigator and is not mandatory for participation in Part I due to time limitations prior to standard surgery.
6. In patients scheduled for trial specific surgery: an echocardiogram assessment should be performed at Visit 1 for assessment of exclusion criterion No 2. If an echocardiogram with acceptable results has been performed within 3 months prior to screening (Visit 1), this may be used for assessment of exclusion criterion No 2 as judged by the Investigator. Patients scheduled for standard surgery: echocardiogram may be performed after Visit 1 as judged by the Investigator and is not mandatory for participation in Part I due to time limitations prior to standard surgery.
7. Medical imaging is performed as needed to confirm that there are identifiable RLNs accessible for minimally invasive trial specific surgery when previously performed standard care imaging provides insufficient basis for this assessment.
8. In case a trial specific surgery for collection of RLN material is required, the blood and tumour sample for NGS will be collected at biopsy procedure as the last screening assessment at Visit 1. For further details, refer to Section 11.2.1, Section 11.2.2 and Section 11.2.3.
9. In case of standard surgery for collection of RLN material, the blood and tumour sample for NGS will be collected at standard surgery. For further details, refer to Section 11.2.1, Section 11.2.2 and Section 11.2.3.
10. Performed in association with standard surgery or as trial specific surgery.
11. Biomarker sampling from blood may be performed on Visit 1 or, alternatively, on Visit 2.
12. Biomarker sampling from RLN starting material for pTTL production. Additional sampling will be performed in association with the pTTL manufacturing process as outlined in Section 11.2.5.1.
13. Tumour biomarker sampling obtained in association with standard surgery for tumour excision. Not applicable for patients undergoing trial specific surgery for collection of RLN material. For further details, see Section 11.2.5.2.
14. Collection of AEs will start in association with surgery or biopsy, whichever comes first.

8.1.2 *Part II: pre-conditioning, supportive care therapy, treatment and 26 weeks safety follow-up*

Part II of the trial entails pre-conditioning and supportive care therapy, treatment with pTTL, and subsequent follow-up as detailed in Table 5 (screening, pre-conditioning, pTTL administration and initial follow-up), Table 6 (detailed schedule of events for Visit 4, pre-conditioning) and Table 7 (26 weeks safety follow-up).

Screening for Part II, pre-conditioning, pTTL administration and patient follow-up will be performed at the Recruitment, Surgery & Treatment site.

Up to 16 patients will be included in Part II with the aim to achieve at least 12 evaluable patients. pTTL will be administered as a single intravenous dose within the dose range $20x10^6$ to 10^9 cells.

Patients will be dosed one by one. For the first three patients, at least 28 days safety and tolerability data for each patient must have been reviewed by the iSRC prior to dosing of the next consecutive patient. From patient 4 in the study, patients will be dosed at least 14 days apart. iSRC meetings are scheduled quarterly after the meeting evaluating patient 3. For further details on the iSRC evaluation and the planned iSRC meetings, see Section 8.4.3.

Part II comprises 18 visits (Visit 3 to Visit 20). Visit 3 is a screening visit for confirmation of eligibility. Visit 4 encompasses Day -7 to Day -1. Supportive care treatment will start on Day -7 or Day -6 and will continue according to schedule with discontinuation of antimicrobial prophylaxis when immune function is judged to be restored. Pre-conditioning will be administered for 3 days, starting on Day -5 and finishing on Day -3. Exceptions to this schedule are allowed and the planned pTTL administration day may be postponed (but will always be referred to as Day 1) as judged by the Investigator. The 3-day preconditioning should, however, be finalised at least 48 hours prior to pTTL administration, and not earlier than 120 hours prior to pTTL administration. The supportive care treatment may be given in an out-patient setting while the 3 days pre-conditioning may be performed in an in-patient or out-patient setting as judged by the Investigator and in line with the prevailing Covid-19 policy at the Recruitment, Surgery & Treatment site.

pTTL will be administered at ME CAST on Day 1, Visit 5. Further details on the administration procedure are provided in Section 10.8. The patients will be discharged from ME CAST after a 7-day post-treatment observation period (i.e. on Day 8), provided that no complications warrant further hospital care. During the in-patient stay, there will be immediate access to appropriate equipment, qualified staff, and an ICU in case of an emergency necessitating such care.

The 3-day pre-conditioning chemotherapy with cyclophosphamide 300 mg/m^2 BSA and fludarabine 30 mg/m^2 BSA should be completed at least 48 hours before pTTL infusion to prevent negative chemotherapy effects on the infused pTTL product. In addition, a maximum of 120 hours between completion of the chemotherapy session and start of pTTL infusion is allowed. The pre-conditioning and supportive care therapy to be administered are shown in Table 11 in Section 11.2.4. Timing of the 3-day chemotherapy in relation to the pTTL administration on Day 1 is shown in Figure 4.

Figure 4 pTTL pre-treatment chemotherapy and administration schedule

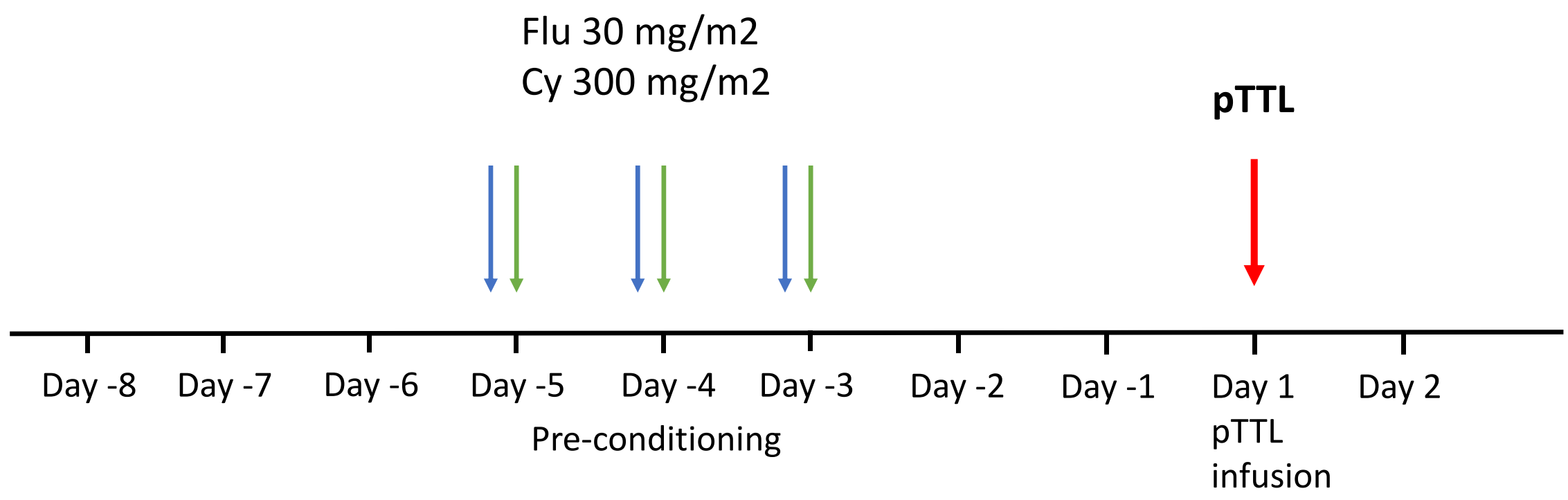

Flu: fludarabine, Cy: cyclophosphamide (blue and green arrows).

Schedule for pre-conditioning and pTTL administration. Preconditioning chemotherapy is administered on three consecutive days, starting on Day -5 and completed on Day -3, in relation to pTTL administration. Exceptions to this schedule are allowed, but the 3-day preconditioning must be finalised earliest 120 hours and latest 48 hours prior to pTTL administration. The patients will receive the pTTL therapy on Day 1 (red arrow).

A confirmation from the pTTL manufacturer that a pTTL product with preliminary release test results is available for thaw and use is required before start of pre-conditioning.

Continued follow-up including safety assessments, biomarker evaluations and medical imaging (including evaluation) will be performed regularly for 26 weeks after pTTL administration. Bi-weekly safety follow-up visits will take place during week 2, 3 and 4 (Visit 6 to Visit 11). During this time, patients living outside the Stockholm area will stay at a patient accommodation close to Karolinska University Hospital Huddinge to be able to receive specialist care in case of AEs. Follow up visits will become gradually less frequent during week 5 to week 26 (Visit 12 to Visit 20), and patients living outside Stockholm may return home.

Participants with tumour progression, confirmed according to iRESCIST criteria, will remain in the trial unless receiving another investigational therapy. Follow up will, however, focus primarily on safety and follow the schedule of Part III, with one main difference: AE collection will be extended from including only AESI to collection of all AEs during the first 6 months after pTTL administration.

The schedule of events for pre-conditioning and supportive care therapy, treatment with pTTL, and subsequent 1-week follow-up is shown in Table 5, details of the supportive care therapy and pre-conditioning visit, Visit 4, are shown in Table 6. The pre-conditioning and supportive care therapy to be administered are shown in Table 11 in Section 11.2.4. The follow-up period in Part II (week 2 to week 26) is outlined in Table 7. Details of biomarker sampling are given in Section 11.2.5.

Part II will end when the last treated patient has been followed for 26 weeks or earlier in case of death or withdrawal of the patient. The total duration of each patient’s involvement in Part II is estimated to approximately 29 weeks including a 14-day screening period, 7 days pre-administration supportive care therapy (of which 3 days includes pre-conditioning) and 26 weeks safety follow-up after pTTL administration.

Trial assessments are described in Section 11

Table 5 Schedule of events Part II: screening, pre-conditioning, pTTL administration and initial treatment follow-up

			**Screening**	**Pre-conditioning**	**pTTL administration and initial treatment follow-up In-patient stay**							
**Visit**			**Visit 3**	**Visit 4**	**Visit 5**							
**Assessment**		**CTP section**	Day-28 to Day-8	Day-7 to Day-1[1]	Day 1	Day 2	Day 3	Day 4	Day 5	Day 6	Day 7	Day 8
Informed Consent Part II and Part III		9.2.3, 14.3	X									
Inclusion/exclusion criteria		9.5	X									
Medical history		11.1.4	X	X								
Serology		11.1.5	X									
Weight		11.1.6	X	X	X	X	X	X	X	X	X	X
Physical examination		11.3.2	X	X[2]	X[3]	X	X	X	X	X	X	X
Vital signs		11.3.3	X	X[2]	X[4]	X[4]	X	X	X	X	X	X
12-lead safety ECG		11.3.5	X	X[2]	X	X					X	
Pregnancy test		11.1.8	X	X[2]								
Spirometry (if indicated)		11.1.11	X									
Echocardiogram		11.1.12	X[5]									
Safety laboratory profile large panel[6]		11.3.4	X		X[3]						X	
Safety laboratory profile small panel[6]		11.3.4		X[2]		X	X	X	X	X		X
Urine analysis		11.3.4	X		X						X	
Covid-19 PCR test		11.1.10		X[2]								
ECOG performance status		11.1.9	X									
Baseline medical imaging (MRI or CT)		11.2.6	X[7]									
Biomarkers[8]	Tumour (tissue biopsy from RECIST non-target lesions)[9]	11.2.5.2	X									
	Blood	11.2.5.3	X	X[2]							X	
Assignment of patient ID, formal inclusion in Part II		9.2.3		X[10]								

		Screening	Pre-conditioning	pTTL administration and initial treatment follow-up In-patient stay							
**Visit**		**Visit 3**	**Visit 4**	**Visit 5**							
**Assessment**	**CTP section**	Day-28 to Day-8	Day-7 to Day-1[1]	Day 1	Day 2	Day 3	Day 4	Day 5	Day 6	Day 7	Day 8
Pre-conditioning and supportive care therapy[11]	11.2.4		X[11]								
pTTL administration	10.8			X							
AEs	11.3.1	--------------------X--------------------									
Prior and concomitant medications	11.1.7	--------------------X--------------------									

AEs=adverse events CT=Computed tomography, CTP=clinical trial protocol, ECG=electrocardiogram, ECOG=Eastern Cooperative Oncology Group, MRI=magnetic resonance imaging

1. Patients may be discharged 1 hour after completed chemotherapy and may be homebased until Day -1 as judged by the Investigator and will in that case come in to the clinic for Covid-19 test (if applicable) and pre-dose safety laboratory sampling on Day -1 (corresponding to the pre-dose large safety panel on Day 1, see Table 5). If considered feasible by the Investigator, and if allowed by the prevailing Covid-19 policy at the Recruitment, Surgery & Treatment Site, patients may also leave the clinic after each day of chemotherapy administration and return the next day in time for the next scheduled chemotherapy administration.
2. Timing of assessments is detailed in Table 6.
3. Pre-dose physical examination and safety laboratory sampling (large panel) may be performed within 24 hours prior to dose.
4. For timing of vital signs assessments in association with pTTL administration (Day 1 and Day 2), see Section 10.8,
5. If an echocardiogram with acceptable results has been performed within 3 months prior to start of pre-conditioning (Visit 4), this may replace an examination performed during the screening (Visit 3) for assessment of exclusion criterion No 2 as judged by the Investigator.
6. Safety blood tests may be drawn more frequently to evaluate potential AEs or for other reasons, and less frequently towards the end of follow-up, depending upon clinical circumstances. For details on parameters in large and small panels, respectively, see 11.3.4. The small panel can always be exchanged for the larger if clinically indicated.
7. Medical imaging within 28 days prior to pTTL administration. Should be the same modality during the whole trial for each patient.
8. Timing and details of biomarker sampling and the analyses to be performed in association with each sampling occasion are provided in Section 11.2.5.2 and Section 11.2.5.3.
9. Tissue biopsies are strongly encouraged unless the risk to the patient is judged to be greater than the potential benefit. Fine needle biopsies are not allowed. Biopsy should be taken within 28 days prior to pTTL administration.
10. Assignment of patient ID will be done on the start day for pre-conditioning therapy (before start of pre-conditioning).
11. Timing and details of pre-conditioning and supportive care therapy are given in Table 6 and in Table 11 Section 11.2.4.

Table 6 Detailed schedule of events Visit 4: supportive care therapy and pre-conditioning

			Out-patient	Out-patient	In-patient or out-patient stay: pre-conditioning[1]			Out-patient	Out-patient
**Assessment**		**CTP section**	Day-7	Day -6	Day -5	Day -4	Day -3	Day -2	Day -1
Medical history		11.1.4			X[5]				
Weight		11.1.6			X	X	X		
Physical examination		11.3.2			X[5]	X	X		
Vital signs		11.3.3			X[5]	X	X		
12-lead safety ECG		11.3.5			X[5]	X	X		
Pregnancy Test		11.1.8							X[2]
Safety laboratory profile small panel[3]		11.3.4			X[5]	X	X		
Covid-19 PCR test		11.1.10		X[4]					X[4]
Biomarkers	Blood	11.2.5.3			X[5]				
Supportive care therapy[6]		11.2.4	X	X	X	X	X	X	X
Assignment of patient ID, formal inclusion in Part II		9.2.3			X[5]				
Pre-conditioning[6]		11.2.4			X	X	X		
AEs		11.3.1	-------------------------------X--------------------------------						
Prior and concomitant medications		11.1.7	-------------------------------X--------------------------------						

AEs=adverse events, CTP=clinical trial protocol, ECG=electrocardiogram

1. Patients will be admitted to the Recruitment, Surgery & Treatment Site for start of pre-conditioning on Day -5. Patients may be discharged 1 hour after completed chemotherapy on Day -3 and may be homebased between Day -3 and Day -1 as judged by the Investigator and will in that case come to the clinic for Covid-19 test (if applicable) and pre-dose safety laboratory sampling on Day -1 (corresponding to the pre-dose large safety panel on Day 1). If considered feasible by the Investigator, and if allowed by the prevailing Covid-19 policy at the Recruitment, Surgery & Treatment Site, patients may also leave the clinic after each day of chemotherapy administration and return the next day in time for the next scheduled chemotherapy administration.
2. Pregnancy test must be performed within 72 hours prior to pTTL administration.
3. For details on parameters in large and small panels, respectively, see Section 11.3.4. The small panel can always be exchanged for the larger if clinically indicated.
4. Covid-19 PCR test prior to admission(s) to the Recruitment, Surgery & Treatment Site, i.e. Day -7 or Day -6 and on Day -1 if required by the prevailing Covid-19 policy at the Recruitment, Surgery & Treatment Site. Additional Covid-19 PCR tests may be performed at the discretion of the Investigator.
5. Prior to start of pre-conditioning. Safety lab sampling and biomarker sampling may be performed on either Day -7 or Day -6 instead of Day -5.
6. Patients with risk for TLS will start supportive care therapy on Day -7. All other patients will start supportive care therapy on Day -6. Additional details of pre-conditioning and supportive care therapy are given in Table 11 Section 11.2.4.

Table 7 Schedule of events Part II: 6 months safety follow-up

Visit		V6	V7	V8	V9	V10	V11	V12	V13	V14	V15	V16	V17	V18	V19	V20
Assessment	CTP section:	W2	W2	W3	W3	W4	W4	W5	W6	W8	W10	W12	W14	W18	W24	W26
		Day 11 ±1 day	Day 14 ±1 day	Day 18 ±1 day	Day 21 ±1 day	Day 25 ±1 day	Day 28 ±1 day									
Weight	11.1.6	X		X		X										
Physical examination[1]	11.3.2	X		X		X		X	X	X	X	X	X	X	X	X
Vital signs	11.3.3	X		X		X		X	X	X	X	X	X	X	X	X
12-lead safety ECG	11.3.5	X		X		X										
Pregnancy Test	11.1.8						X			X		X	X	X	X	
Medical imaging (MRI or CT)	11.2.6								X			X		X	X	
Tumour progression evaluation	11.2.6								X			X		X	X	
Safety laboratory profile large panel	11.3.4		X		X		X		X	X						
Safety laboratory profile small panel	11.3.4	X		X		X		X			X	X	X	X	X	X
Urine analysis	11.3.4		X		X		X		X	X						
Bio-markers[2] – Tumour (tissue biopsy from RECIST non-target lesions)[3]	11.2.5.2						X[3]									
Bio-markers[2] – Blood	11.2.5.3		X				X		X			X		X	X	
AEs	11.3.1	--------------------X--------------------														
Concomitant medications	11.1.7	--------------------X--------------------														

CTP=clinical trial protocol, CT=Computed tomography, MRI=magnetic resonance imaging W=week

1. Physical examination may be replaced by telephone contact at the discretion of the responsible Investigator, but trial patients must have a physical visit at least once 2 weeks until Visit 14, and every 4 weeks between Visits 14 and 18, and finally, either Visit 19 or 20, depending on when the last radiological evaluation results are available.
2. Timing and details of biomarker sampling and the analyses to be performed in association with each sampling occasion are provided in Section 11.2.5.2 and Section 11.2.5.3.
3. Tissue biopsies are strongly encouraged unless the risk to the patient is judged to be greater than the potential benefit. Fine needle biopsies are not allowed. Biopsy approximately 4 weeks (Day 28 ±7 days) after pTTL administration.

8.1.3 *Part III: long-term follow-up*

Patients who have completed Part II of the trial will continue in a long-term follow-up, which, for each patient, will continue for 4.5 years or until death, whichever comes first. Additionally, patients with confirmed tumour progression will enter Part III of the trial after the visit where tumour progression has been communicated to the patient, unless they enter a clinical trial to receive another investigational therapy. The first visit (Visit 21) will occur 3 months after the last visit in Part II. The visits will then continue at the same intervals as for patients entering Part III after completing all of Part II. Visit times may be adjusted slightly within the allowed windows shown in Table 8 to make the total follow up after pTTL administration 5 years, adjusting for the patient leaving Part II earlier.

During the long term-follow-up, patients will visit the Recruitment, Surgery & Treatment Site at regular occasions (quarterly to once every 6 months over the first 2 years and then once yearly), see Table 8. In case medical imaging has not been performed as part of the patient's standard of care, MRI/CT may be scheduled at the discretion of the Investigator. Any AESI assessed as at least potentially related to pTTL will be recorded in the electronic case report form (eCRF). Information for treatment efficacy evaluation, including tumour progression and any additional anti-tumoral therapy the patient receives will be collected, as will survival data, including cause of death when applicable. Blood samples for biomarker analysis to evaluate pTTL persistence and to assess tumour burden will be obtained. The samples will be used for immunological analysis with the purpose to study the persistence of pTTL derived T cells in circulation in relation to tumour progression and survival time. Details of biomarker sampling is given in Section 11.2.5. Data collection will be continued until death, or for a maximum of 4.5 years after completion of Part II.

The following endpoints are relevant for the long-term follow-up part of the trial:

Primary endpoint:

- For patients who have not entered Part III early due to tumour progression, safety will have been assessed in Part II of the trial, but AE data collection will be continued as regards immunological AE: AESIs with suspected relationship to pTTL therapy. The AESI definition includes autoimmune reactions potentially resulting from off-target toxicity and immune-mediated reactions associated with immune cell activation.
- For patients with tumour progression entering Part III earlier than 6 months after pTTL administration, all AEs will be collected until 6 months after pTTL administration has passed. After this timepoint, AE data collection will continue as described above.

Secondary endpoints:

- Overall survival
- Tumour progression according to iRECIST (as performed in clinical follow-up of the patient), including time to progression
- Progression-free survival
- Duration of response

Exploratory endpoints:

- Long-term persistence of pTTL, and, if applicable, functional and phenotypical properties of persisting pTTL

- Tumour burden analysis

Table 8 Schedule of events Part III: long term follow-up

**Visit**			**V21**	**V22**	**V23**	**V24**	**V25-27**
**Assessment**		**CTP section:**	9 months[2] ±1 month	12 months[2] ±1 month	18 months[2] ±1 month	24 months[2] ±1 month	Yearly FU ±2 months
Tumour progression evaluation/survival		11.2.6	X	X	X	X	X
Biomarkers	Blood	11.2.5.3	X	X	X	X	X
AESI[1]		11.3.1.6	X	X	X	X	X
Anti-tumoral therapy		11.1.7	X	X	X	X	X

AESI=AEs of special interest, CTP=clinical trial protocol

1. All AEs are collected for patients entering Part III early due to tumour progression until 6 months after pTTL administration.
2. The time lapsed after pTTL will be different from what is stated here for patients entering Part III early due to tumour progression, but the intervals between visits are the same.

8.2 Assessments at each visit

8.2.1 *Visit 1: Screening Part I*

- Informed consent Part I (the informed consent form for Part I must be signed by the patient and investigator prior to performing any trial specific procedures). For details on the informed consent procedures, see Section 14.3.
- Registration of the patient in the trial eCRF, which will generate a unique trial ID, see Section 9.2.2.
- Inclusion/Exclusion Criteria, see Section 9.4.
- Pregnancy test, see Section 11.1.8.
- Demographics, see Section 11.1.3.
- Weight, height and BMI, see Section 11.1.6.
- Medical history, see Section 11.1.4.
- Blood samples for serology testing, see Section 11.1.5. The results must be in place before collection of starting material for pTTL production.
- Physical examination, see Section 11.3.2.
- Vital signs, see Section 11.3.3.
- ECG, see Section 11.3.2.
- Blood samples and urine sample for safety tests, see Section 11.3.4.
    - *Safety test results from analyses that have been performed for other purposes within 1 week before Visit 1 can be used at the discretion of the Investigator.*
- ECOG performance status, see Section 11.1.9.
- Pre-operative medical imaging (MRI or CT) if needed prior to trial specific surgery for RLN collection, see Section 11.2.6.
- Biomarker sampling (blood), see Section 11.2.5.3. The biomarker sampling of blood may instead be performed at Visit 2.

- Spirometry (if indicated), see Section 11.1.11.
    - *In patients scheduled for trial specific surgery, a spirometry assessment should (if indicated) be performed at Visit 1 for assessment of exclusion criterion No 3.*

      In patients scheduled for standard surgery, spirometry may be performed after Visit 1 as judged by the Investigator. Spirometry for patients scheduled for standard surgery is not mandatory for participation in Part I, regardless if considered indicated before pTTL therapy.
- Echocardiogram, see Section 11.1.12.
    - *In patients scheduled for trial specific surgery, an echocardiogram assessment should be performed at Visit 1 for assessment of exclusion criterion No 2. The echocardiogram at Visit 1 is not mandatory if an echocardiogram with acceptable results has been performed within 3 months prior to Visit 1 as judged by the Investigator.*

      In patients scheduled for standard surgery, echocardiogram may be performed after Visit 1 as judged by the Investigator. The echocardiogram for patients scheduled for standard surgery is not mandatory for participation in Part I.
- Prior and concomitant medications, see Section 11.1.7.

The assessments may be performed on different days for e.g. logistical reasons.

8.2.2 *Visit 2: Surgery (Part I)*

- Blood samples for serology testing, see Section 11.1.5.
- Weight, see Section 11.1.6.
- Physical examination, see Section 11.3.2.
- Vital signs, see Section 11.3.3.
- ECG, see Section 11.3.2.
- Echocardiogram, see Section 11.1.12.
    - *In patients scheduled for standard surgery, an echocardiogram assessment may be performed before or after surgery (Visit 2) as judged by the Investigator. The echocardiogram for patients scheduled for standard surgery is not mandatory for participation in Part I.*
- Blood samples for safety tests, see Section 11.3.4.
- Collection of tumour sample (biopsy or surgically excised tumour material) for NGS, see Section 11.2.1.
- Collection of blood sample for NGS (normal control), see Section 11.2.2.
- Collection of starting material for pTTL production from RLNs for pTTL production, see Section 11.2.3.
- Biomarker sampling (pTTL and tumour and normal tissue): see Section 11.2.5.1 and Section 11.2.5.2, respectively.
- If not performed at Visit 1: biomarker sampling (blood), see Section 11.2.5.3.
- Medical history (events between Visit 1 and Visit 2).
- AEs (from surgery or biopsy, whichever comes first).
- Prior medications, see Section 11.1.7.

In case trial specific surgery for collection of RLN material is required, the tumour sample will be collected via biopsy. The 2 procedures may be performed on different occasions. For further details, refer to Section 11.2.1 and Section 11.2.3.

If the patient does not proceed to Part II, the collected samples will be used for pre-clinical development providing the patient does not withdraw their consent.

8.2.3 *Visit 3: Screening Part II*

- Informed consent Part II and Part III (the informed consent form for Part II must be signed by the patient and investigator prior to performing any trial specific procedures in Part II), see Section 14.3.
- Inclusion/Exclusion Criteria, see Section 9.5.
- Medical History, see Section 11.1.4.
- Weight, see Section 11.1.6.
- Physical examination, see Section 11.3.2.
- Vital signs, see Section 11.3.3.
- ECG, see Section 11.3.2.
- Pregnancy test, see Section 11.1.8.
- Blood samples for serology testing, see Section 11.1.5.
- Blood samples (large panel) and urine sample for safety tests (results used for entry criteria evaluation), see Section 11.3.4.
- ECOG performance status, see Section 11.1.9.
- Schedule MRI or CT for week 6, see Section 11.2.6.
- Medical Imaging within 28 days before start of trial treatment. (MR of the brain in cases of CNS metastases must be performed not later than 4 weeks before start of treatment), see Section 11.2.6.
- Biomarker sampling (tumour biopsy from RECIST non-target lesions and blood): see Section 11.2.5.2 and Section 11.2.5.3, respectively.
- Spirometry (if indicated), see Section 11.1.11.
- Echocardiogram, see Section 11.1.12
    - *An echocardiogram assessment should be performed at Visit 3 for assessment of exclusion criterion No 2. The echocardiogram at Visit 3 is not mandatory if an echocardiogram with acceptable results has been performed within 3 months prior to the planned start of pre-conditioning (Visit 4) as judged by the Investigator.*
- Prior medications, see Section 11.1.7.

The assessments may be performed on different days for e.g. logistical reasons.

8.2.4 *Visit 4: Pre-conditioning and supportive care therapy (Part II)*

Supportive care therapy: up to 7 days between Day -7 and Day -1

Day -7:

Start of supportive care therapy (allopurinol) for patients with risk of TLS according to treatment schedule, see Section 11.2.4.

Day -6:

- Continued supportive care therapy for patients with risk of TLS and start of supportive care therapy for the remaining patients, see Section 11.2.4.

Day -7 or Day -6

Covid-19 PCR test if required by the prevailing Covid-19 policy at the Recruitment, Surgery & Treatment Site. Performed one or two days prior to start of chemotherapy, at the convenience of the Recruitment, Surgery & Treatment Site.

Day -5 to Day -1

- Day -5: assignment of patient ID (prior to start of pre-conditioning), see Section 9.2.3.
- Day -5 to Day -3: 3 days pre-conditioning (chemotherapy), see Section 11.2.4.
- Day -5 to Day -1: continued supportive care therapy, see Section 11.2.4.
- Weight, (each day of pre-conditioning [chemotherapy], i.e. Day -5, Day -4 and Day -3), see Section 11.1.6.
- Physical examination (each day of pre-conditioning [chemotherapy], i.e. Day -5, Day -4 and Day -3), see Section 11.3.2.
- Pregnancy test (once within 72 hours prior to treatment with pTTL), see Section 11.1.8.
- Vital signs (daily during the 3-day pre-conditioning [chemotherapy], i.e. Day -5, Day -4 and Day -3), see Section 11.3.3.
- Blood samples for safety tests. Small panel prior to start of pre-conditioning and on second and third day of pre-conditioning, see Section 11.3.4. Urine sample prior to start of pre-conditioning.
- ECG (daily during the 3-day pre-conditioning [chemotherapy], i.e. Day -5, Day -4 and Day -3), see Section 11.3.5.
- Biomarker sampling (blood) (prior to start of pre-conditioning [chemotherapy]): see Section 11.2.5.3.
- Prior medications, see Section 11.1.7.

Patients may be homebased between treatments and assessments on all days as judged by the Investigator and if allowed by the prevailing Covid-19 policy at the Recruitment, Surgery & Treatment Site.

While preconditioning is planned to occur between Day -5 and Day -3, exceptions are allowed, and the planned pTTL administration day (Day 1) may be postponed as judged by the Investigator. The 3-day preconditioning should, however, be finalised at least 48 hours prior to pTTL administration, and not earlier than 120 hours prior to pTTL administration.

8.2.5 *Visit 5: treatment with pTTL and initial treatment follow-up (Part II)*

Day 1, i.e. the day of pTTL administration, is expected to occur approximately 48-120 hours after the end of pre-conditioning chemotherapy. There must be at least 48 hours between completed chemotherapy and pTTL administration.

Treatment (Day 1)

- Intravenous infusion of pTTL, see Section 10.8.
- Weight, see Section 11.1.6.
- Physical examination (pre-dose, may be performed within 24 hours prior to dose), see Section 11.3.2.

- Vital signs (pre- and post-dose), see Section 11.3.3 and Section 10.8.
- Blood samples (large panel, pre-dose, may be performed within 24 hours prior to dose) and urine sample (pre-dose) for safety tests, see Section 11.3.4.
- ECG (pre-dose), see Section 11.3.5.
- AEs, see Section 11.3.1.
- Concomitant medications, see Section 11.1.7.

Post-treatment (Day 2)

- Weight, see Section 11.1.6.
- Physical examination, see Section 11.3.2.
- Vital signs, see Section 11.3.3 and Section 10.8.
- Blood samples (small panel) for safety tests, see Section 11.3.4.
- ECG, see Section 11.3.5.
- AEs, see Section 11.3.1.
- Concomitant medications, see Section 11.1.7.

Post-treatment (Day 3)

- Weight, see Section 11.1.6.
- Physical examination, see Section 11.3.2.
- Vital signs, see Section 11.3.3.
- Blood samples (small panel) for safety tests, see Section 11.3.4.
- AEs, see Section 11.3.1.
- Concomitant medications, see Section 11.1.7

Post-treatment (Day 4, 5, 6, 7, 8):

- Blood samples for safety tests, see Section 11.3.4. Day 4, Day 5, Day 6 and Day 8: small panel, Day 7: large panel. Urine sampling concomitantly with large panel blood sampling.
- AEs, see Section 11.3.1.
- Concomitant medications, see Section 11.1.7.
- Weight, see Section 11.1.6.
- Physical examination, see Section 11.3.2.
- Vital signs, see Section 11.3.3.

Day 7 only

- ECG, see Section 11.3.5.

8.2.6 *Visit 6 to Visit 20: 26 weeks safety follow-up (Part II)*

The assessments to be performed at Visit 6 to Visit 20 are specified in Table 7.

8.2.7 *Visit 21 and onwards: long-term follow-up (Part III)*

The assessments to be performed during the long-term follow-up are specified in Table 8.

8.3 Rationale for trial design

The EMA guideline on strategies to identify and mitigate risks for FIH and early clinical trials with IMPs (2017) has been considered, as has e.g. European Commission guideline on Good Clinical Practice (GCP) specific to Advanced Therapy Medicinal Products (2019).

The design of the trial is chosen with the aim to study safety, tolerability and preliminary efficacy of a single dose of pTTL within a dose range of minimum $20x10^6$ and maximum 10^9 viable cells in a limited number of patients with stage IV CRC. The administered dose of pTTL is determined by the production yield of each pTTL product, within the defined dose range. The design involves careful monitoring of the patient's well-being. The trial will provide important safety, tolerability and efficacy data to support the design of further studies in patients.

In regard to the trial population, patients with rapid tumour progression may not be able to wait the time required for NGS, antigen selection and pTTL manufacturing (8-12 weeks from collection of tumour material for sequencing) if included at a late stage. Therefore, inclusion of patients in Part I of the trial (for collection of samples for sequencing and of starting materials) while they still have other non-curative treatment options can be beneficial, as judged by the Coordinating Investigator; the Principal Investigator at the Site where the surgery is planned, the Investigator at ME CAST, and the Sponsor's medical representative. The production of pTTL and subsequent pTTL administration can then be performed at a later time point, giving a delay from decision to treat to administration of only 2-3 weeks.

The trial design includes a pre-conditioning treatment with chemotherapy (according to standard conditions for cellular therapies), which will be given on 3 consecutive days prior to pTTL infusion (Berdeja et al. 2021). Pre-conditioning lymphodepleting chemotherapy provides an optimal environment for the infused T cells, by elimination of regulatory T cells, increase of T cell homeostatic cytokines and elimination of resident T cells competing for trophic cytokines (Klebanoff et al. 2005). A crucial role of pre-conditioning chemotherapy has been demonstrated in adoptive T cell therapy, by promoting expansion, tumour infiltration and persistence of infused T cells (Bechman and Maher 2021; Chapuis et al. 2017; Pockaj et al. 1994). The doses used in the pre-conditioning therapy included in this protocol are relatively low, and the side effects, based on previous experiences are expected to be limited (Neelapu 2019; Ying et al. 2019; Zhang et al. 2017). No post-infusion cytokine treatment is included in the trial design, which is expected to significantly reduce toxicity compared to, for example, what is seen with TIL therapy (Andersen et al. 2016; Besser et al. 2020; Goff et al. 2016).

In general, the use of the pre-conditioning treatment with cyclophosphamide and fludarabine can lead to myelosuppression. Therefore, a high index of suspicion for occult bacteraemia should be maintained until marrow recovery. Myelosuppression could theoretically occur after initial recovery as a side effect of pTTL therapy, as is seen with CD19 CAR T cell therapy (Locke et al. 2022). After discharge, if a patient develops fever or other signs of infection, safety blood and urine analyses together with other appropriate tests should be performed, and an assessment should be made by an Investigator at the Recruitment, Surgery & Treatment Site to initiate antimicrobial therapy if indicated.

The majority of acute reactions are expected to occur within the timeframe of 3 days after pTTL administration. Observation for one week after pTTL administration have been judged appropriate to add a safety margin in where any acute reactions should be detected.

After the initial follow-up within Part II, patients will be followed long-term in Part III of the trial. The health of the patient, and tumour progression, including side-effects of increasing

tumour burden, will be monitored and managed according to standard procedures for patients with stage IV CRC, and palliative care will be applied according to institutional standard. Data to enable analysis of pTTL treatment efficacy and survival will be collected. AESI, or immunological events, suspected to be associated to pTTL therapy will also be collected and reported to the appropriate authorities.

The transition of follow up from Part II to Part III at 6 months after pTTL administration was decided based on the very low likelihood of additional AEs of pTTL therapy occurring at such a long time after a single administered dose. Also, as the patient's included in the trial have advanced disease, and tumour progression within 6 months is likely in patients with limited or no effect of pTTL. Patients with such effect of pTTL that tumour progression has been halted for 6 months or more is considered possible to monitor according to standard practise for metastasised CRC.

8.4 Selection of doses and rationale for dosing strategy

8.4.1 *Selection of dose, and rationale for minimum and maximum dose*

The cell yield obtained during the manufacturing process of pTTL will vary depending on the size of the neoantigen-reactive clones present in the starting material and their proliferative capacity. The dose range for pTTL was selected taking into consideration the expected production range and with attention to potential safety concerns. pTTL characteristics and experiences from other T cell ACTs formed the basis for the selection of appropriate minimum and maximum doses, see below. Dose selection and associated safety considerations are discussed also in the IB.

The minimum dose of a pTTL unit, 20 $x10^6$ viable cells, is chosen as the lowest yield considered to have an acceptable potential for therapeutic effect. The main concern is the ability of a small number of cells to be able to reach the tumour and exert effective tumour killing. In the setting of donor lymphocyte infusions, used to treat manifest or threatening relapse of haematological malignancy after allogeneic stem cell transplantation, doses comparable to the minimum pTTL dose, $0.5x10^6$ CD3+ T cells/kg body weight is used. This is an indication that doses this low can have clinically significant effect (Sureda et al, 2024. the EBMT handbook). The maximum dose, 10^9 viable cells, is set taking into consideration the expected range of successfully produced pTTL units.

The dosing strategy in the present trial, to administer the entire cell yield from pTTL production if the dose falls within the product specification, has been designed taking into account that cell dose is expected to be poorly predictive of toxicity, and also of the potency of a pTTL product. This argues against the use of a classical dose escalation. Other product-related and patient-specific factors are considered more likely to contribute to pTTLs potential to induce anti-tumour effects and to cause toxicity.

Potency and T cell-associated toxicity are expected to depend mainly on the characteristics and, to a lesser extent, the size of activated T cell clones in the product. For toxicity, the pTTL clones' potential for cross reactivity between target neoantigens and normal proteins is an important factor. Currently, there are no tools to accurately predict which clones have the potential to contribute to potency and/or toxicity and to determine important characteristics as e.g. effector status for these specific clones. The dosing strategy can thus not at present be adjusted to take these characteristics into account.

The composition of clones and their characteristics will be unique to each pTTL product and hence for each patient. Therefore, a dose-oriented safety evaluation of one pTTL product is

not considered predictive of the dose-related safety profile of another product. For an individual patient, it is possible that a higher dose of pTTL might result in a more rapid onset of T cell activity, and thus early inflammation-related toxicity, than a lower dose from the same product. However, a cell dose-based comparison of risk between two different products intended for two different individuals is unlikely to correctly predict T cell-related toxicity. Another aspect complicating cell dose comparisons is the fact that the cell number can be expected to change in the human body after pTTL infusion according to the properties of the T cells, e.g. T cell subtype, memory or effector status and whether the T cells are exhausted or senescent, and in response to signals encountered after infusion.

One aspect of toxicity which, in fact, may be associated with cell dose relates to impurities in the product. Impurities to consider in this context are cell debris from dead cells and non-T-cells. The variability with regard to debris between products is, however, relatively limited within the dose range of pTTL when compared to what is expected with, for example, the far larger TIL products, with cell numbers that are up to several orders of magnitude higher. The non-T cells include mainly autologous NK cells and B cells which are minimally changed by the manufacturing process and should pose a minimal risk at re-infusion. The risks associated with cell debris and non-T-cells within the product are therefore considered to be small.

Prediction of toxicity for pTTL is based on knowledge of the product's characteristics and on experiences from other T cell therapies. Comparability between T cell ATMPs when it comes to potential for off-target effect is complicated, but the available knowledge aids in providing some information on the risks.

Therapies similar to pTTL, *i.e.* autologous T cells with a native T cell receptor and limited *in vitro* manipulation given as a single dose, have been associated with limited risk and relatively large dose ranges are considered safe.

Data from cell therapies relevant for comparison indicate that pTTL is likely to have a favourable safety profile. These include:

- **TIL therapy**: The intended dose range for pTTL is considerably lower than what is used in TIL therapy. Here, standard doses have been 10-1000-fold larger than the ones estimated for pTTL (Andersen et al. 2016; Besser et al. 2020; Dafni et al. 2019; Goff et al. 2016). The recently approved TIL therapy Amtagvi (Lifileucel) has an accepted dose range of 7.5 x 10^9 to 72 x 10^9, which is wide and encompass doses 7.5-72 times higher than the highest pTTL dose. A median dose of 21x10^9 cells has been reported from a Phase 2 trial with Lifileucel (Chesney et al. 2022). Even though the products are not directly comparable they have many similarities (see e.g Section 6.1.1 and Section 6.4) and the expectation is that the safety profile of pTTL will be similar, or possibly more favourable, to what has been seen for TILs. According to the literature, most adverse reactions following TIL administration have been associated with pre-conditioning and IL-2 administration rather than with the drug itself. Toxicity associated directly with the cell product, mainly infusion reactions and immune-related reactions such as vitiligo and uveitis, have not been reported to correlate to cell dose (Betof Warner et al., 2024; Julve et al., 2024). Interestingly, immune-related toxicities have only been seen in patients with melanoma and has been associated with T cell reactivity towards melanoma antigens. Similar toxicities have not been described for patients with other solid tumours (Betof Warner et al., 2024).
- An **SLN-based therapy** produced using tumour homegenate-stimulation and given without pre-conditioning chemotherapy or post-infusion cytokine therapy. This T cell product is similar to pTTL, using the same type of starting material and a relatively

comparable production strategy (see Section 6.4). The therapy was associated with almost no side effects (Karlsson et al. 2010; Sherif et al. 2015; Zhen et al. 2015). The reported average doses and ranges were similar to pTTL's: $71x10^6$ cells (range $1x10^6$-$270x10^6$; Karlsson et al. 2010), $185x10^6$ cells (range 13 $x10^6$-704 $x10^6$; Sherif et al. 2015) and $153x10^6$ cells (range $21x10^6$-$639x10^6$; Zhen et al. 2015), supporting that the selected pTTL dose range is safe. Of note, in the SLN ACT trials, a single lymph node was used for manufacture of the IMP, while several RLNs will be used for pTTL, increasing the chance of larger starting materials and potentially also larger products. This has resulted in the selection of a somewhat higher maximum dose for pTTL than what was administered in the SLN trials referred to above.

- The neoantigen-targeting T cell therapy **cNeT** developed by Achilles Therapeutics has been studied in trials including similar pre-conditioning therapy to what is applied in the current trial, but with additional post-infusion IL-2. cNeT was found to have an acceptable safety profile for doses from 14 x 10^6 to >6.5 x 10^9, given to 32 patients (Turajlic et al. 2021, Interim clinical data report in the Achilles Therapeutics Press Release April 4 2024), and no correlation between the cNet dose and tolerability was observed (Achilles presentation at TIL summit Oct. 2024). Of note, three cases of grades 1-2 CRS and one case of ICANS were reported (Turajlic et al. 2021), indicating that neoantigen stimulation could increase the risk of toxicity related to T cell activation, possibly as a result of a higher degree of targeted T cells in the product as compared to TIL. However, the safety profile was considered comparable to the safety profile seen for unselected TILs, without any perceived increase in risk.
- For their T cell product **GEN-011**, Genocea has presented similar tolerability data for the two dose cohorts (one cohort with multiple lower doses and one with one higher dose) evaluated in the Phase I/II trial in patients with refractory solid tumours (Gillison et al., 2022). CRS, maximum grade 2, and one grade 2 ICANS was reported.
- Turnstone Biologics have presented data for four CRC patients treated with their **TIDAL-01** product in Phase I trials. The target dose was 10^9 cells and all patients received >10^9 cells. The safety profile was favourable, with adverse reactions considered as associated with pre-conditioning, post-infusion IL-2 and pembrolizumab, and not with the ATIMP (Turnstone Biologics Press Release August 14 2024).
- A personalised neoantigen-specific T cell therapy with **BNT221**, an autologous cell product derived from blood, has been evaluated for safety and tolerability in a dose-finding Phase I trial (NCT04625205). Two dose levels, 10^8-10^9 cells and $2x10^9$-10^{10} cells, were tested in three and six advanced stage melanoma patients respectively. No dose-limiting toxicity or related SAE was reported (Borgers et al. 2025).

No correlation between dose and toxicity was noted in either study. See also Section 6.4.

In contrast to these examples, dose-limiting toxicity has been described for CAR T cell therapies (Kochenderfer et al. 2010, 2012, 2013). An initial dose range of 1 x 10^9 – 1 x 10^{10} total CAR T cells was de-escalated in several steps due to toxicity. This product is, however, genetically modified to express a chimeric antigen receptor which binds strongly to its target and has incorporated co-stimulatory elements enhancing activation and is thus expected to have a higher level of toxicity than what is expected for pTTL, but the safe dose range is relatively close to the dose range allowed for pTTL. The maximum pTTL dose of 10^9 viable cells is also comparable to the upper limit for the CAR T cell product Kymriah, which is set to $600x10^6$ CAR+ cells. pTTL is a more heterogenous product than Kymriah, with multiple

specificities, and a somewhat larger dose is on this ground expected to be acceptable with regards to toxicity.

Tecelra, a product containing T cells modified by introduction of a T cell receptor targeting a MAGE-A4 epitope, which through its modification should have a safety profile more similar to CAR T cells, have a wide accepted dose range of 1-10x10^9, indicating that even genetically modified T cells could be safe at cell doses higher than the pTTL dose range. Tecelra has been approved by FDA for treatment of advanced synovial sarcoma (Tecelra FDA approval August 2 2024).

In summary, the above-described experiences from other T cell ACTs have led to the proposed dose strategy for pTTL. Dose-related toxicity is considered unlikely for pTTL based on experiences with the most similar T cell therapies, and also difficult to quantify due to each product's unique properties. The maximum pTTL dose is considered to be safe. This is based primarily on clinical precedence of the similar product AMTAGVI, that is approved for doses that are up to 72 times higher than the proposed maximum dose for pTTL (AMTAGVI FDA Summary Basis for Regulatory Action. 2024). Moreover, a number of neoantigen-specific therapies have been evaluated at similar or higher doses (1-10 x 10^9) with few reported adverse reactions.

As outlined in Section 8.4.2 and Section 8.4.3, strict dose stopping criteria will apply and regular monitoring of emerging data will be carried out by an iSRC throughout the study.

8.4.2 *Stopping criteria and trial stopping rules*

The Investigators and the iSRC (Section 8.4.3) will follow the recommendations and grading system of CTCAE v5.0, see Section 11.3.1.8 but also take into account the recommendations published by Sibille et al. 2010, which is an adaptation to FIH studies of the grading systems previously proposed by National Cancer Institute (NCI), World Health Organisation (WHO), National Institute of Health (NIH) and the FDA. The grade and the frequency of AEs will be considered.

A rolling review of emerging safety data will be performed throughout the trial considering any AEs, the number of patients in whom they occur, concurrency of more than one event within the same patient and any trends. Changes from baseline measurements will also be considered and not just absolute cut-off based on upper- or lower limits of normal.

Trends or safety signals may warrant the scheduling of *ad hoc* iSRC meetings after which the iSRC will make recommendations to the Sponsor on e.g. whether to continue or terminate the trial.

AEs and SAEs are expected to occur frequently in this trial based on the patient population and the nature of the advanced disease under study. Regular systematic review of SAEs and AESI will serve as the basis for pausing or prematurely stopping the trial. SAEs that are assessed as related to pTTL will be the primary criteria for halting or stopping the trial. Review of these SAEs and any decision to pause enrolment or terminate the trial, will be determined by the iSRC and the Sponsor, see Section 8.4.3.

An *ad hoc* iSRC meeting will be scheduled if:

- Three or more patients experience an AR (grade ≥3) not classified as a SAR
- One or more patients develop an AESI (≥grade 2) not classified as a SAR

- One or more patients develop an AE related to study-specific procedures or study treatments (other than pTTL) which are considered a safety concern in the opinion of the Investigator, or
- Any other safety concerns are identified, as judged by the investigator.
- Patient enrolment will be paused pending an iSRC meeting if one patient experiences a SAR.

The iSRC will evaluate safety findings and decide whether the finding warrants a halt of the trial and an amendment to the protocol. The iSRC evaluation will determine whether a definitive termination of the trial is indicated based on findings of compromised patient safety.

A SAR is defined as an SAE occurring in a patient treated with pTTL and for which causality has been assessed as at least possible by the Investigator or the Medical Monitor, see Section 11.3.1.4.

Additional withdrawal criteria are summarised in Section 9.9.

8.4.3 *Internal safety review committee*

The voting members of the iSRC will consist of the Coordinating/Principal Investigator, [redacted], MD, PhD, the Investigator, [redacted], MD, PhD, Associate Professor, at the Recruitment, Surgery & Treatment Site (or delegates) and the Sponsor's medical representative (or delegate). In addition, the clinical research manager (CRM) and additional Sponsor representatives and internal/external experts will be invited as appropriate.

As outlined in Table 9, in general, patients will be dosed one by one, at least 14 days apart. For the first three subjects, however, at least 28 days safety and tolerability data must have been reviewed by the iSRC before dosing of the next patient. If the safety and tolerability, as assessed individually for each of the first three patients, is considered acceptable by the iSRC, a written recommendation to proceed to the next patient will be provided to the Sponsor. The maximum dose of 10^9 viable cells cannot be exceeded.

After three patients have been treated, iSRC meetings will be scheduled approximately quarterly. If no new data of relevance are available, the iSRC meeting can be postponed to approximately 28 days after pTTL administration for the next patient to be treated in the trial.

If any safety concern arises, the iSRC may recommend that the trial is halted for further investigation of perceived risks, or even terminated if the perceived risk justifies such a measure (see Section 8.4.2)

The recommendation of the iSRC will be made in consensus between the iSRC members and documented as appropriate. In case there is disagreement between the 3 voting members, the most conservative approach will be taken. Further details regarding iSRC review and the data to be reviewed will be provided in a separate iSRC charter.

In addition to assessing emerging safety data and providing recommendations for conduct of the trial, the iSRC will also review and approve the surgery method at a pre-surgery meeting in all cases where bowel resection is considered, see Table 9. The evaluation will include an individual risk/benefit assessment to determine if resection surgery is considered acceptable to access starting material for pTTL. An independent medical expert will be included as a voting member at each pre-surgery meeting. Furthermore, at least 2 surgeons (including the surgeon who made the initial assessment of surgery method) must have evaluated the proposed surgery method, and at least 1 of them will be invited to participate in the meeting. A

recommendation for the procedure from the committee is required before the surgery is performed. The recommendation of the iSRC will be made in consensus between the iSRC members and documented as appropriate. In case there is disagreement between the 4 voting members, the most conservative approach will be taken.

In case the trial is temporary halted for safety reasons and if the Risk/Benefit ratio is changed, the Sponsor will submit a substantial amendment to the competent authority (CA) and independent ethics committee (IEC), and the trial will not be restarted until approval.

Table 9 Planned safety review committee meetings

iSRC meeting	Number of patients	Data to be reviewed	Meeting outcome
No 1	1	At least 28 days safety and tolerability data for the first patient.	If safety and tolerability of pTTL treatment is considered acceptable: proceed to patient 2
No 2	2	All available data for the first patient assessed as relevant by the iSRC.<br>At least 28 days safety and tolerability data for patient 2.	If safety and tolerability of pTTL treatment is considered acceptable: proceed patient 3.
No 3	3	All available data for patients 1 and 2 assessed as relevant by the iSRC.<br>At least 28 days safety and tolerability data for patient 3.	If safety and tolerability of pTTL treatment is considered acceptable: proceed to patient 4 and dosing of the remaining patients (at least 14 days apart)
No 4 and all other upcoming quarterly meetings	Any number of patients	28 days safety and tolerability data for all treated patients and all additional available data assessed as relevant by the iSRC.	If safety and tolerability of pTTL treatment is considered acceptable: proceed with the trial as planned
Ad hoc meeting	Any number of patients	All available data assessed as relevant by the iSRC.	As determined by assessment of concerns raised which triggered the meeting
Pre-surgery meeting	Patients who are considered for study specific surgery involving bowel resection	Results from medical examinations and safety samples at screening.<br>Data on disease stage and prior treatment.<br>Radiology data for assessment of RLN accessibility.<br>Surgeon's report on minimal invasive surgery method of choice with acceptable prospect of retrieving RLNs.	Recommendation on surgery method and whether resection surgery is acceptable, based on risk/benefit assessment.

9 TRIAL POPULATION

Prospective approval of protocol deviations to eligibility criteria, also known as protocol waivers or exemptions, is not permitted.

The selected target group of the trial is adults with metastatic adenocarcinoma of the colon or rectum (stage IV CRC) with no or limited (but not curative) therapeutic options.

Patients are eligible for inclusion if they have received treatments according to institutional standard of care, or if further standard of care treatment options are judged not to be in the patients' best interest (e.g. due to toxicity issues), or the patient have declined available standard of care treatment options. Patients can also be eligible for inclusion in Part II of the trial and to receive pTTL therapy in a scheduled break in an ongoing non-curative standard of care therapy at the discretion of their treating Investigator.

Eligible patients must have a primary or metastatic lesion that is accessible to biopsy or surgery to obtain tumour material for NGS analysis and must be willing and able to undergo the procedure. In addition, it is a requirement that the patient is able to undergo surgical excision of RLNs for use in pTTL production. This could be performed during palliative standard of care surgery of the primary tumour due to i. e. tumour obstruction, or bleeding from the tumour. Patients can be included in Part I of the trial at surgery even while standard, non-curative, therapy options remain, as the starting material is cryopreserved and available for pTTL production at a later stage. In patients not scheduled for standard surgery, starting material will have to be obtained through trial specific surgery. It is a prerequisite that the collection of RLNs can be performed with minimal clinical impact, and with good chances of obtaining starting material. To prevent performing unsuccessful surgery where RLNs could not be obtained, patients will undergo radiological screening, and only proceed to surgery if accessible lymph nodes can be identified. Also, only patients judged suitable for surgery based on patient characteristics such as previous abdominal surgery, anatomy, and other patient characteristics of importance, can be included.

Examples of patient groups of special interest include stage IV CRC patients with microsatellite instability (MSI) that have progression on checkpoint inhibitor therapy, due to the accessibility of these tumour to immunotherapy. However, all stage IV CRC patients fulfilling the requirements above are eligible for inclusion.

9.1 Recruitment

Stage IV CRC patients will be identified by the Referral sites, by the Recruitment & Surgery Sites and by the Recruitment, Surgery & Treatment Site, see Section 5.2, Section 9.2.1 and Section 9.2.2.

9.2 Screening and enrolment log

Investigators must keep a record of all screened patients even if they were not subsequently included in the trial. This information is necessary to verify that patients were selected without bias. The reason for screen failure should be stated for all patients screened but not included. The reason for withdrawal should be stated for all patients included but not completed.

Inclusion into Part I and Part II/III of the trial are separate procedures. The screening and inclusion process for Part I of the trial is performed at one of the Recruitment & Surgery Sites or at the Recruitment, Surgery & Treatment Site. The screening and inclusion in Part II are

performed at the Recruitment, Surgery & Treatment Site. The procedures are described in Section 9.2.2 and Section 9.2.3 below.

9.2.1 *Pre-screening Part I*

A potential trial participant will be asked to sign consent to share imaging data and medical records necessary to enable discussions regarding the patient's potential suitability for trial participation, see Section 9.2.2. All pre-screened patients will be documented in a pre-screening log, but no screening number will be assigned until the patient has been confirmed as suitable for screening, and the patient has signed consent to participate in the trial at Visit 1.

9.2.2 *Screening and inclusion Part I*

Eligible patients are identified by Referral Sites or by the Recruitment, Surgery & Treatment Site or the Recruitment & Surgery Sites. In association with screening of a potential trial patient, the following actions are initiated at any of the Recruitment Sites:

- The suitability of the intended trial patient is confirmed following a discussion between the Investigators at the Recruitment, Surgery & Treatment Site, the Principal Investigator at the Recruitment & Surgery Site where the surgery is planned and the Sponsor (a consensus decision may not be feasible for patients included in Part I in association with standard surgery for palliative purposes planned with short notice, see Section 11.2.1).
- A unique screening number will be allocated to each patient in connection to the informed consent process at the screening visit (Visit 1). The screening number is generated automatically in the eCRF and will follow the patient throughout the trial. The screening number will allow identification of patients irrespective of their possible eligibility for the trial.
- A notification is sent via the eCRF system to Sponsor representatives, to EpiTCer® bead production (ngs@neogap.se) and to selected representatives at CTC. The patient is registered in the PIOR® Manufacturing bioinformatic software system by the Sponsor and is given a unique patient batch identity code (PBID) in this system. The screening ID and PBID will be linked.
- Once the patient has been found eligible, a notification will be sent in accordance with the quality agreement (if possible via the eCRF system) to the pTTL production unit Vecura, Karolinska University Hospital Huddinge, Stockholm, Sweden as well as to other predefined recipients. The site that performed the screening procedures will inform Vecura about the scheduled date for the surgery when starting material for manufacturing will be obtained.

For details regarding informed consent procedures for Part I, see Section 14.3. For assessments to be performed at the screening visit (Visit 1) and at the surgery visit (Visit 2), see Section 8.2.1.

9.2.3 *Screening and inclusion Part II*

Patients will come for a second screening visit (Visit 3) prior to inclusion in Part II. The second screening visit will be performed at the Recruitment, Surgery & Treatment Site.

To proceed to Part II of the trial, the following two steps included in Part I must have been completed:

- Successful analysis of NGS data and production of TC0301, to allow for initiation of pTTL manufacturing.
- Collection, processing, and cryopreservation of RLN-derived starting material with a sufficient cell dose to initiate pTTL production.

When this has been achieved, preparations for Part II can be initiated. The clinical status of the trial patient is assessed by the responsible Investigator, and safety blood and urine samples are analysed at the screening visit to ascertain that it is feasible to administer pTTL in 2 to 3 weeks. The suitability of the intended trial patient is confirmed following a discussion between the Investigators at the Recruitment, Surgery & Treatment Site and the Sponsor. The cryopreserved pTTL product is subjected to release testing. Pre-conditioning may be initiated based on preliminary release test results. The pre-conditioning chemotherapy will start on Day -5 before scheduled infusion of pTTL on Day 1 (final release test results are required before pTTL infusion). The details of product specification are found in the IMPD.

Once pTTL has been preliminary released for patient administration, and prior to the start of the pre-conditioning on Day -5, the patient will be formally included in Part II and a 3-digit patient ID (101, 102 etc.) will be assigned.

For details regarding informed consent procedures for Part II, see Section 14.3. For assessments to be performed at the screening visit (Visit 3), see Section 8.1.2.

9.3 Number of patients

No formal inclusion limit is set for Part I of the trial in order to accommodate to the possibility that some of the included patients will be unable to continue to Part II due to e.g. disease progression.

Up to 16 patients will be included in Part II with the aim to achieve at least 12 evaluable patients treated with pTTL. An evaluable patient is defined as a patient who has received treatment with pTTL (fulfilling the specification) and for whom at least 6 weeks follow-up data are available (unless death occurred).

For replacements of patients who discontinues early from the trial, see Section 9.9.3.

9.4 Eligibility criteria Part I

9.4.1 *Inclusion criteria Part I*

For inclusion in Part I, patients must fulfil the following criteria:

1. Signed informed consent.
2. Adult (age ≥18 years).
3. Histological or cytological confirmation of CRC.
4. Verified metastatic disease (stage IV classification).
5. Measurable disease according to RECIST1.1.
6. Minimum life expectancy of 6 months.
7. ECOG performance status 0 to 1.
8. Adequate hematopoietic, hepatic and renal function defined as:
    a. Haemoglobin ≥ 95 g/L (blood transfusion not less than 21 days prior to screening),
    b. Absolute neutrophil count ≥ 1.0 x 10^9/L, and
    c. Platelets ≥100 x 10^9/L.

       In the case of recent bleeding, haemoglobin <95 but >85 g/L and blood transfusion more recent than 28 days are allowed, provided that criteria b, and c are met.
    d. Total bilirubin < 1.5 x ULN (does not apply to patients with Gilberts Syndrome)
    e. AST and ALT ≤ 1.5 x ULN (or ≤ 5 x ULN in the presence of liver metastases)
    f. Serum creatinine ≤ ULN (if serum creatinine is between 1 and 1.5 x ULN, patients may be eligible provided that the calculated GFR is at least 35 mL/min using Cockcroft-Gault method).
    g. Albumin ≥24 g/L.
9. Patients of childbearing potential or their partners of childbearing potential must be willing to take the appropriate precaution to avoid pregnancy or fathering a child for the duration of Part II and practice an approved, highly effective method of birth control during treatment and for 6 months after receiving pTTL. For patients of childbearing potential, appropriate precautions to avoid pregnancy is required also in Part I, as pregnancy would preclude proceeding to Part II of the trial.

   Approved methods of birth control include:

    - Combined (oestrogen and progesterone containing) hormonal birth control associated with inhibition of ovulation: oral, intravaginal, transdermal
    - Progesterone-only hormonal birth control associated with inhibition of ovulation: oral, injectable, implantable
    - Intrauterine device (IUD) or intrauterine hormone-releasing system (IUS)
    - Bilateral tubal occlusion
    - Vasectomised partner

- True sexual abstinence when this is in line with the preferred and usual lifestyle of the patient. Periodic abstinence (e.g., calendar ovulation, symptothermal, post-ovulation methods) is not acceptable.

10. Able to undergo surgery or biopsy to obtain tumour tissue for neoantigen evaluation and to retrieve RLNs as starting material for pTTL manufacturing.
11. The area from which the RLNs will be obtained shall not have been exposed to radiotherapy.

9.4.2 *Exclusion criteria Part I*

Patients must not enter Part I if any of the following exclusion criteria are fulfilled:

1. Less than 4 months since a clinically significant cardiovascular event such as myocardial infarction, unstable angina, angioplasty, bypass surgery, stroke or transient ischemic attack (TIA). Atrial fibrillation if treated and well controlled is not considered a bar to inclusion even if diagnosed less than 6 months ago.
2. *For patients scheduled for trial specific surgery:* Left ventricular ejection fraction (LVEF) <45% and/or significant heart valve dysfunction (stenosis or insufficiency) as determined by echocardiogram performed within 3 months of the screening visit (Visit 1).

   For patients scheduled for standard surgery: congestive heart failure New York Heart Association (NYHA) class III or IV. Echocardiogram for inclusion in Part I is not mandatory due to time limitations prior to standard surgery.
3. *For patients scheduled for trial specific surgery*: Significantly reduced lung function with clinical implications. If such is suspected, spirometry should be performed. Spirometry should also be considered in patients who have been hospitalised due to Covid-19 infection during the last 6 months, and in patients with any other lung affectation judged significant by the Investigators, in discussion with Sponsor's Medical Representative, such as treatment-related pneumonitis or severe lung infection. Spirometry results of less than 65% of the expected value regarding forced expiratory volume in 1 second (FEV1) and/or diffusion capacity (diffusing capacity of the lung for carbon monoxide, DLCO, corrected for haemoglobin value, DLCOco) is regarded as a criterium for exclusion.

   For patients scheduled for standard surgery: not applicable - spirometry for inclusion in Part I is not mandatory due to time limitations prior to standard surgery.
4. Any severe acute or chronic medical condition that places the patient at increased risk or interferes with the interpretation of trial results (as judged by the Investigators, in agreement with Sponsor's Medical Representative).
5. Immunodeficiency disorders which may pose a risk for patients treated with pTTL, and/or affect the outcome of the pTTL treatment, as judged by the Investigator at the Recruitment, Surgery & Treatment Site.

   Immunodeficiency disorders are here defined as including inborn and acquired disorders reducing immunity but excluding human immunodeficiency virus (HIV), which is discussed below. Examples include common variable immunodeficiency and status post transplantation of a solid organ or stem cells.

   Immunodeficiency caused by the cancer disorder to be treated within the trial or such cancer treatments as have already been administered is considered a separate entity.

This, if severe, might impact the production of pTTL and potentially also the treatment outcome, and needs to be carefully assessed as regards patient and pTTL production risks before inclusion.

6. Autoimmunity disorders which may pose a risk for patients treated with pTTL, and/or affect the outcome of the pTTL treatment, as judged by the Investigator at the Recruitment, Surgery & Treatment Site.
7. Leptomeningeal metastases (patient with previously treated brain metastases are eligible if there is no evidence of disease progression for a minimum of 8 weeks prior to inclusion – in these cases a CNS MRI is required within the screening period. These patients must not have symptoms from their brain metastases or treatment thereof and must not be taking steroid medications for treatment of CNS symptoms).
8. Systemic immunosuppressive concomitant medications, including chemotherapy for CRC and steroids for antiemetic prophylaxis, must be discontinued at a minimum 2 weeks prior to surgery. Steroid medications are allowed if they are used as substitution or are administrated topically or as inhalation steroids for asthma.
9. Previous Grade 3 or greater immune-related toxicity from checkpoint modulation or other immunotherapy (unless the toxicity has resolved and the patient rechallenged with the therapy without recurrence of toxicity, in which situation the patient can be considered).
10. Acute or chronic infection with hepatitis B or C or syphilis.
11. HIV infection.
12. Pregnancy or breast-feeding.
13. Investigator considers the patient unlikely to comply with trial procedures, restrictions and requirements.
14. For patients required to undergo trial specific surgery to obtain starting material:
    a. No identifiable lymph nodes on pre-surgery radiology accessible, or lymph nodes assessed as unsuitable for surgical excision.
    b. Previous surgical removal of the primary CRC tumour (would entail a high risk surgery)
    c. Unable to withstand the planned surgery (including ineligibility for general anaesthesia).

9.5 Eligibility criteria Part II

9.5.1 *Inclusion criteria Part II*

1. Signed informed consent.
2. Adult (age ≥18 years).
3. Histological or cytological confirmation of CRC.
4. Verified metastatic disease (stage IV classification).
5. Measurable disease according to RECIST1.1.
6. Have received all possible standard of care therapies, OR further standard of care therapies are currently not considered to be in the patient's best interest, OR toxicity from previous therapy limits the choice of suitable standard of care therapy OR scheduled pause in palliative standard of care therapy as judged by the Investigator.
7. Minimum life expectancy of 3 months from the time that the individual pTTL product is estimated to be available (as per Investigators clinical assessment).
8. ECOG performance status 0 to 1.
9. Adequate hematopoietic, hepatic and renal function defined as:
   a) Haemoglobin ≥ 95 g/L (blood transfusion not less than 21 days prior to screening),
   b) Absolute neutrophil count ≥ 1.0 x 10^9/L, and
   c) Platelets ≥100 x 10^9/L.

      In the case of recent bleeding, haemoglobin <95 but >85 g/L and blood transfusion more recent than 28 days are allowed, provided that criteria b, and c are met.
   d) Total bilirubin < 1.5 x ULN (does not apply to patients with Gilberts Syndrome)
   e) AST and ALT ≤ 1.5 x ULN (or ≤ 5 x ULN in the presence of liver metastases)
   f) Serum creatinine ≤ ULN (if serum creatinine is between 1 and 1.5 x ULN, patients may be eligible provided that the calculated GFR is at least 35 mL/min using Cockcroft-Gault method).
   g) Albumin ≥24 g/L.
10. Patients of childbearing potential or their partners of childbearing potential must be willing to take the appropriate precaution to avoid pregnancy or fathering a child for the duration of Part II and practice an approved, highly effective method of birth control during treatment and for 6 months after receiving pTTL.
    Approved methods of birth control include:
    a) Combined (oestrogen and progesterone containing) hormonal birth control associated with inhibition of ovulation: oral, intravaginal, transdermal
    b) Progesterone-only hormonal birth control associated with inhibition of ovulation: oral, injectable, implantable
    c) IUD or IUS
    d) Bilateral tubal occlusion
    e) Vasectomised partner

f) True sexual abstinence when this is in line with the preferred and usual lifestyle of the patient. Periodic abstinence (e.g., calendar ovulation, symptothermal, post-ovulation methods) is not acceptable.

9.5.2 *Exclusion criteria Part II*

1. Less than 6 months since a clinically significant cardiovascular event such as myocardial infarction, unstable angina, angioplasty, bypass surgery, stroke or TIA. Atrial fibrillation if treated and well controlled is not considered a bar to inclusion even if diagnosed less than 6 months ago.
2. LVEF <45% and/or significant heart valve dysfunction (stenosis or insufficiency) as determined by echocardiogram performed within 3 month before start of pre-conditioning therapy, or more recently if clinically indicated.
3. Significantly reduced lung function with clinical implications. If such is suspected, spirometry should be performed. Spirometry should also be considered in patients who have been hospitalised due to Covid-19 infection during the last 6 months, and in patients with any other lung affectation judged significant by the Investigators, in discussion with Sponsor's Medical Representative, such as treatment-related pneumonitis or severe lung infection. Spirometry results of less than 65% of the expected value regarding FEV1 and/or diffusion capacity (diffusing capacity of the lung for carbon monoxide, DLCO, corrected for haemoglobin value, DLCOco) is regarded as a criterium for exclusion.
4. Any severe acute or chronic medical condition that places the patient at increased risk or interferes with the interpretation of trial results (as judged by the Investigators, in agreement with Sponsor's Medical Representative).
5. Immunodeficiency disorders which may pose a risk for patients treated with pTTL, and/or affect the outcome of the pTTL treatment, as judged by the Investigator at the Recruitment, Surgery & Treatment Site.

   Immunodeficiency disorders are here defined as including inborn and acquired disorders reducing immunity but excluding HIV which is discussed below. Examples include common variable immunodeficiency and status post transplantation of a solid organ or stem cells.

   Immunodeficiency caused by the cancer disorder to be treated within the trial or such cancer treatments as have already been administered is considered a separate entity. This, if severe, might impact the production of pTTL and potentially also the treatment outcome, and needs to be carefully assessed as regards patient and pTTL production risks before inclusion.
6. Autoimmunity disorders which may pose a risk for patients treated with pTTL, and/or affect the outcome of the pTTL treatment, as judged by the Investigator at the Recruitment, Surgery & Treatment Site.
7. Leptomeningeal metastases (patient with previously treated brain metastases are eligible if there is no evidence of disease progression for a minimum of 8 weeks prior to inclusion – in these cases a CNS MRI is required within the screening period. These patients must not have symptoms from their brain metastases or treatment thereof and must not be taking steroid medications for treatment of CNS symptoms).
8. Systemic immunosuppressive concomitant medications must be discontinued at a minimum 2 weeks prior pTTL administration. Steroid medications are allowed if they

are used as substitution or are administrated topically or as inhalation steroids for asthma.

9. Previous Grade 3 or greater immune-related toxicity from checkpoint modulation or other immunotherapy (unless the toxicity has resolved and the patient rechallenged with the therapy without recurrence of toxicity, in which situation the patient can be considered).
10. Acute or chronic infection with hepatitis B or C or syphilis.
11. HIV infection.
12. Pregnancy or breast-feeding.
13. Investigator considers the patient unlikely to comply with trial procedures, restrictions and requirements.
14. Less than 4 weeks prior to start of pre-conditioning since stopping previous systemic cancer treatment.
15. Less than 2 weeks prior to start of pre-conditioning since stopping radiotherapy.
16. Less than 4 weeks prior to start of pre-conditioning after major surgery and less than 3 weeks after minor surgery.
17. Participation in any other clinical cancer therapy trial, and planned treatment or treatment with another investigational drug, within the previous 4 weeks prior to start of pre-conditioning.
18. Less than 4 weeks prior to start of pre-conditioning since administration of live attenuated vaccines.

9.6 Prior and concomitant medication

No investigational treatments may be administered within 4 weeks prior to administration of pTTL and while the trial patient is under follow-up and evaluation after pTTL administration. Trial patients who wish to participate in another trial in order to receive other investigational therapy or patients receiving standard of care cancer therapy after treatment with pTTL will be withdrawn from the current trial.

Live attenuated vaccines should not be administered within 4 weeks prior to the start of trial therapy, during therapy and follow-up. Live attenuated vaccines may be considered no earlier than 3 months after trial patients have received pTTL, and not prior to iRECIST evaluation scheduled at 12 weeks after administration.

Immunosuppressive or immunomodulatory medications, such as cytotoxic agents, thalidomide, steroids and tumour necrosis factor α (TNFα) inhibitors are not allowed within 2 weeks of starting therapy or during follow-up. This does not apply to topical or inhalation steroids, which are not prohibited.

The use of immunosuppressive agents is allowed for treating trial related immune-mediated toxicity, but only after careful consideration, see Section 11.3.1.17 for more detailed information.

Ongoing treatment of other underlying diseases such as heart disease, hypertonia, and hypothyreosis, and therapies aiming to alleviate cancer-related symptoms such as pain are generally allowed during the trial, but all ongoing treatments will be evaluated by the Investigator performing patient inclusion.

9.7 Prior and concomitant procedures

Radiotherapy to relieve symptoms is allowed while the patient is in the trial. pTTL will not be administered together with radiotherapy but may be administered 2 weeks prior to or 2 weeks after completion of radiotherapy. If radiotherapy of a target lesion is required, the lesion will no longer be considered measurable according to iRECIST criteria. The date of any radiotherapy and the area subjected to radiotherapy will be recorded in the eCRF.

9.8 Screening failures

Screening failures are defined as patients who consent to participate in the clinical trial but do not fulfil all eligibility criteria. A minimal set of screening failure information is required to ensure transparent reporting of screening failures. Minimal information includes documentation of signed and dated ICF and reason(s) for screening failure.

Screening failure may also be a result of pTTL manufacturing failure, in which case the patient cannot receive an approved pTTL product. If production fails during the early part of the process, either prior to surgery, due to failure in obtaining tumour material of sufficient quality for NGS analysis, or prior to start of pre-conditioning with chemotherapy, the trial patient is considered a screening failure.

Patients who do not meet the criteria for participation in Part I or Part II may be rescreened. Rescreening can be performed once if any of the following were reasons for screening failure (as judged by the Investigator):

- Any transient condition that precluded inclusion at first screening, but which has since healed or passed.
- Any transient condition causing a laboratory test or examination to yield results precluding inclusion at first screening, but which has since improved.
- Positive Covid-19 test and other significant infections as judged by the Investigator.

In case of re-screening for participation in Part I, the patient will be given a new screening number. The final screening number in Part I (original or new in case of re-screening) will be the same for the patient during the remaining part of the trial, also in case of re-screening for Part II.

9.9 Patient withdrawal

9.9.1 *General withdrawal criteria*

Patients are free to discontinue their participation in the trial at any time and for whatever reason without affecting their right to an appropriate follow-up investigation or their future care. If possible, the reason for withdrawal of consent should be documented.

Patients may also be withdrawn from the trial at any time at the discretion of the Investigator.

Reasons for withdrawal include:

- Patient's surgery/biopsy material does not meet the requirements for pTTL production
- pTTL production failure
- Eligibility criteria for Part II not fulfilled (screening failure Part II)
- Patient decision (withdrawal of consent)
- Severe non-compliance to trial protocol procedures, as judged by the Investigator and/or Sponsor

- The patient's own well (at the discretion of the Investigator or Sponsor for safety, behavioural, or administrative reasons)
- Death
- Treatment with any other investigational drug

Progression of the underlying CRC is not a cause for withdrawal unless the patient is treated with another investigational therapy. Thus, treatment with standard-of-care therapy at tumour progression after pTTL administration does not result in withdrawal. However, in the case of tumour progression, with or without treatment with standard therapy, further follow up is primarily focused on safety. Details of how follow up is organized after tumour progression have been diagnosed according to the iRESCIST criteria can be found in Section 8.1.3.

9.9.2 *Procedures for discontinuation of a patient from the trial*

In cases of premature withdrawal from the trial, follow-up of remaining issues associated with unresolved AEs will be performed.

For patients who withdraw their consent, no further trial specific procedures may be performed, and no additional data collected. The Sponsor may retain and continue to use any data collected before withdrawal of consent. If the patient agrees, an end-of-trial visit will be performed in association with patient withdrawal. At the end-of-trial visit, the patient will be asked about the reason(s) for discontinuation, a medical history review and a physical examination will be performed and safety blood (large panel of end-of-trial visit occurs within 3 months after pTTL treatment, small panel otherwise) and urine sampling will be performed as specified in Section 11.3.4. Any ongoing AEs will be followed as described in Section 11.3.1.18. Follow-up concerning survival will be maintained.

The primary reason for discontinuation/early withdrawal must be specified in the eCRF.

9.9.3 *Patient replacement*

No formal inclusion limit is set for Part I of the trial in order to accommodate to the possibility that some of the included patients will be unable to continue to Part II due to e.g. disease progression.

No replacements will be made in Part II. Up to 16 patients will be included in Part II to ensure 12 evaluable patients.

10 TREATMENTS

10.1 Identity of advanced therapy investigational medicinal products

The drug product, i.e. pTTL, is composed of *in vitro* expanded RLN cells, enriched for neoantigen specific T cells. It is formulated as a cell suspension, 1-10 x10^6 viable cells per mL, with the excipients Ringer-Acetate with human serum albumin (HSA; 25 mg/mL) and CS10 volume/volume in a transfusion bag intended for intravenous transfusion. The product is cryopreserved after production and thawed just before administration.

pTTL is different for each patient as the neoantigen combination is unique for each individual tumour and due to the use of autologous RLN-derived T cells. The cell culture conditions during pTTL manufacturing promote selective expansion of T cell clones specifically recognising neoantigen epitopes included in NAG on the EpiTCer beads. Thus, pTTL is a T cell product, mainly composed of CD45+CD3+ T cells and to minor extent of other

autologous cells such as B cells and NK cells. The product will include both CD4+ and CD8+ T cells, displaying individual varying CD4/CD8 ratios, see Section 6.3.

The cell dose obtained during the manufacturing process will vary depending on the size of the neoantigen-reactive clones present in the starting material and their proliferative capacity. The minimum dose of a pTTL unit is 20 $x10^6$ viable cells and the maximum dose is 10^9 viable cells.

10.2 Identity of non-investigational medicinal products

Pre-conditioning therapy will be administered to all patients prior to treatment with pTTL. The pre-conditioning therapy includes 3 days intravenous infusion of cyclophosphamide 300 mg/m^2 BSA and fludarabine 30 mg/m^2 BSA. The pre-conditioning therapy infusion should be completed at least 48 hours before pTTL infusion to prevent negative chemotherapy effects on the infused pTTL product.

All patients will also receive supportive care therapy in association with pTTL administration (over Day -7 to Day 8 in accordance with standard of care). Supportive care therapies are based on standard practises and include intravenous hydration, antiemetic therapy, antimicrobial prophylaxis, prophylaxis against tumour lysis syndrome (TLS) and uroprotective measures (if deemed appropriate) in conjunction with administration of chemotherapy. Details on doses, routes of administration and duration of treatment are given in Section 11.2.4.

10.3 Manufacturing of the drug substance

pTTL is manufactured according to GMP at a GMP-compliant tissue establishment centre and production facility (Vecura, Karolinska University Hospital, Huddinge).

The pTTL manufacturing process is a multi-step procedure which begins with collection of starting material from the patient in a surgery room. The starting material is transported in cell medium containing antibiotics by a designated courier to the production facility Vecura.

Production starts with processing of the starting material into a single-cell suspension. The cell suspension will be cryopreserved until the patient is ready for inclusion in the second, treatment, part of the trial. The cell expansion part of the pTTL production is possible to start when the personalised TC0301 has been prepared. Manufacture of TC0301 involves analysis of sequencing data obtained from tumour and normal tissue samples (see Section 11.2.1 and Section 11.2.2) by the PIOR® Manufacturing bioinformatic software developed by the Sponsor, which detects and ranks mutations likely to result in neoantigens. The neoantigenic peptide sections resulting from the selected mutations are combined to form a maximum of six specially designed neoantigen peptides, and recombinantly produced. They are then coupled to superparamagnetic beads to form TC0301.

An overview of the pTTL production procedure is shown in Figure 5. The single cell suspension of thawed RLN cells is seeded in culture flasks, stimulated using patient-specific TC0301 and cultured in medium supplemented with IL-2. Antibiotics are present in the cell culture medium at start of the culture and gradually phased out as the starting medium is replaced by medium without antibiotics. Cell cultures are harvested after 12 or 15 days of culture, according to pre-set criteria for the cell monitoring analyses. The raw material TC0301 is removed from the harvested cells by the application of a magnet and the resulting cell suspension forms the drug substance, pTTL-DS.

pTTL-DS is formulated into the drug product by adding a solution containing Ringer-Acetate and HSA. Then, the product is cryopreserved after addition of the cryopreservation solution

CS10. In parallel, a sample of the product is cryopreserved in a separate vial for release testing. Product release analyses are performed on this sample to assess the cell content and quality of the product. Microbial testing is part of the release testing but is performed on the formulated product before cryopreservation. Preliminary release is based on the cell content/quality analyses and interim results of the microbial testing, while product release requires the final microbial test results to be included.

Figure 5 pTTL manufacturing overview

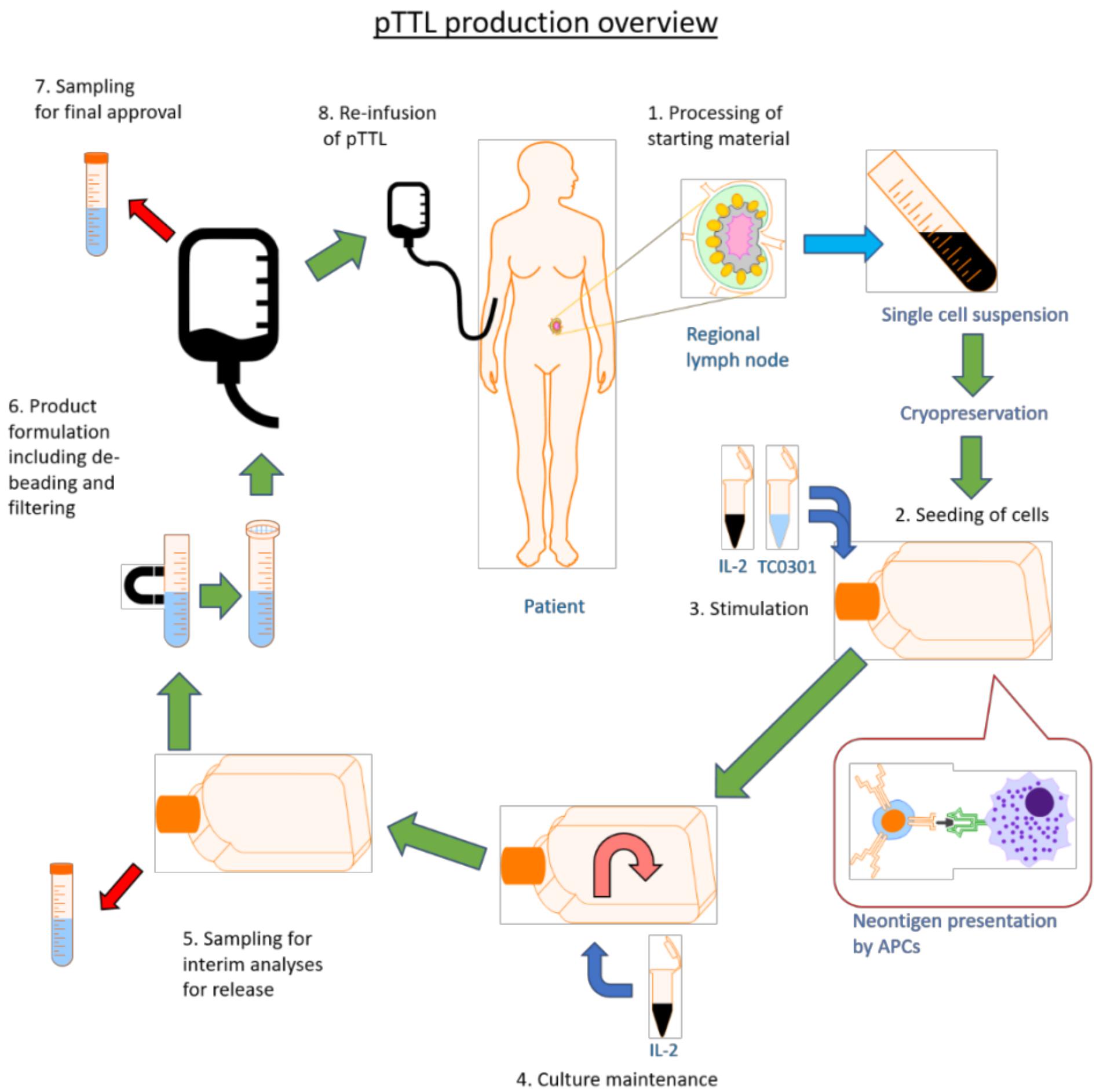

Starting material is RLNs that are processed into single cell suspension and cryopreserved (1). Thawed cells are seeded in culture flasks (2), and TC0301 EpiTCer beads and IL-2 are added to the culture (3). During cell culture selective cell expansion of neoantigen-specific T cell clones takes place (4). During the course of cell culturing sampling for interim analysis is performed (5). Cells are harvested and debeaded, forming the pTTL-DS (6). Cells are formulated, cryopreserved, and pTTL released (7). Thawed pTTL is administrated to the patient (8).

10.4 Manufacturing of the drug product, packaging, labelling and release

The drug product, pTTL, is provided cryopreserved, and contains a frozen suspension of 1-10 x10^6 viable cells/mL in Ringer-Acetate and HSA (25 mg/mL) mixed volume/volume with CS10 (contains DMSO).

Prior to formulation, possible aggregates are removed from the pTTL-DS cell suspension using a 100 µm cell strainer, the cells are washed by centrifugation and resuspended in Ringer-Acetate supplemented with 25 mg/ml HSA. An equal volume of CS10 is added, and

the product, pTTL, is then formulated in a bag intended for cryopreservation. The product is frozen at a controlled rate and preserved at ≤-150ºC.

Written instructions on handling of pTTL will be available to the personnel at the Recruitment, Surgery & Treatment Site. The pTTL product is transported to the Site and thawed there just prior to administration by intravenous infusion. No further reconstitution or dilution is required before administration. Administration is performed through an infusion line especially intended for transfusion of blood products containing an in-line filter.

Labels will comply with applicable GMP requirements, with Annex 13 of the European Union GMP regulations and local regulatory requirements.

There may be instances where pTTL will not meet the quality requirements as laid down in the specification of the IMPD. For example, the product may contain fewer cells than specified (less than $20x10^6$ viable cells) and therefore falls out of specification. In such exceptional cases, pTTL may still be administered to the patient following a positive benefit/risk assessment by the manufacturer and the research physician at the manufacturer site, the Coordinating Investigator and Sponsor's medical representative.

The manufacturer should provide the treating Investigator with its evaluation of the risks and notify the Investigator that the out of specification product is being supplied at his/her request. The confirmation of the treating Investigator to accept the product should be recorded by the manufacturer.

10.5 Shipment of pTTL product and conditions for storage

After formulation, the pTTL product is cryopreserved and stored at the GMP manufacturing unit, Vecura. Before scheduled administration, the product is transported frozen to the Recruitment, Surgery & Treatment Site and thawed by staff from ME Clinical immunology and Transfusion medicine, Karolinska University Hospital, Huddinge.

10.6 Traceability

Traceability of autologous pTTL starting material and product is critical in the autologous immunotherapeutic setting. The manufacturer must ultimately ensure that the patient receives only their own cell preparation, as the inherent safety of autologous cell products is that they represent 'self'. Records will be kept, which document each individual product to ensure traceability from sourcing, manufacturing, packaging, storing, transport, delivery to ME CAST, administration to the patients, reconciliation and destruction or final disposition.

A stringent system and staff training will be in place to ensure that pTTL is only administered in an autologous manner.

10.7 Preparation and accountability

pTTL handling will be done by trained personnel in accordance with standard procedures at ME CAST and ME Clinical immunology and Transfusion medicine.

The Investigator will maintain a Storage and Accountability Log as well as a Drug Dispensing Log detailing the dates and quantities of trial medication received and used for each patient and trial medication destroyed after each pTTL administration. Any discrepancies between prepared and returned pTTL product must be explained and documented. Products deliberately and/or accidentally destroyed by the site must be accounted for.

10.8 Treatment administration and associated procedures

pTTL is stored in, and infused from, a bag especially suited to the cryopreservation of blood cells. Just prior to infusion, pTTL is thawed and administered to the patient within 1 hour after thawing. The bag with the pTTL product shall be connected to a standard infusion kit for transfusion of blood products equipped with a filter. This is then connected to a venous catheter for infusion to the patient.

pTTL should be administered in as short time as possible. The volume to administer is determined by the viable cell count, as determined by the release test analysis, and the entire volume of the product should be infused. The infusion rate, and thus the infusion time, is decided upon by the Investigator present at the pTTL administration, taking into account the volume of the product and any relevant underlying disease such as congestive heart failure or renal failure. Diuretic medication can be considered based on medical considerations. The total infusion time depends on the volume of the pTTL product, and will vary, as volumes can range from 10 to 100 mL. However, the maximum estimated infusion time is 1 hour. Start and stop time of the infusion, product volume and dose will be recorded in the eCRF. Any issues during infusion will also be recorded in the eCRF.

The product bag is flushed with sterile Ringer-Acetate after infusion, and the bag is gently shaken to release remaining cells adhering to the plastic of the bag. The added volume is then infused at the same infusion rate as the product. If necessary due to risk of clotting in the filter, the transfusion kit can be changed.

Monitoring of vital signs, level of consciousness and the patient's general condition will be performed according to the schedule stated below before, during and after the infusion of pTTL.

The following will be collected and assessed in association with pTTL infusion:

- Blood pressure
- Pulse
- Blood oxygenation (pulse oximetry)
- Respiratory frequency
- Temperature
- Level of consciousness
- An assessment of the patient's general condition is also performed and noted (symptoms like pain, discomfort, nausea, shivering, confusion etc is recorded as AEs).

The measurements will be performed:

- Before start of infusion
- Every 30 minutes (+/- 10 minutes) during infusion of pTTL, during infusion of the Ringer-Acetate used to wash out pTTL cells remaining inside the bag after infusion, and for 2 hours after the wash-out Ringer-Acetate infusion has been terminated.

  In case the infusion is shorter than 30 minutes, the first measurement will be performed approximately 30 minutes (+/- 10 minutes) after start of the infusion.
- Hourly (+/- 15 minutes) between 2 hours and 4 hours after the wash-out Ringer-Acetate infusion.
- Six hours (+/- 30 minutes) after end of pTTL administration, or, when appropriate, after the wash-out Ringer-Acetate infusion, and every sixth hour (+/- 30 minutes) until 24 hours have elapsed.

Management of acute ARs is discussed in Section 11.3.1.17.

10.9 Continuation of treatment with advanced therapy investigational medicinal product

There will be no treatment with pTTL after end-of-trial.

10.10 Treatment compliance

pTTL will be administered at ME CAST under medical supervision to ensure compliance.

10.11 Return and destruction of advanced therapy investigational medicinal product

After pTTL administration, the used bag is preserved and refrigerated for 4 hours in order to allow further testing in case of an AR. After this time, the bag is discarded according to the standard procedures for disposal of biohazard classified waste.

Any unused product will be saved for immunological analyses in a Biobank for up to 10 years.

The Monitor will perform final ATIMP accountability reconciliation including a source document verification against the documentation of shipped, received, dispensed and disposed product. The pTTL label will be available in 2 copies, one will be saved for accountability purposes.

11 TRIAL ASSESSMENTS

The trial assessments are described in the sections below and the timing of these assessments are detailed in the schedule of events (Table 4 [Part I], Table 5 [Part II: pre-conditioning, pTTL product administration and treatment follow-up, Table 6 [Part II pre-conditioning and supportive care - details], Table 7 [Part II 26 weeks safety follow-up] and Table 8 [Part III long term follow-up]).

11.1 Recording of data and baseline assessments

The Principal Investigator at each site will provide the Sponsor with all data produced during the trial from the scheduled trial assessments. He/she ensures the accuracy, completeness, legibility, and timeliness of the data reported to Sponsor in the eCRF and in all required reports.

11.1.1 *Informed consent*

Signed informed consent must be obtained before any screening procedures are initiated. The informed consent procedure is further described in Section 14.3.

11.1.2 *Eligibility criteria*

Eligibility criteria should be checked during each screening visit and will be verified before pTTL administration. The criteria are specified in Section 9.4 (Part I) and Section 9.5 (Part II).

11.1.3 *Demographic information*

The following demographic data will be recorded: gender, age, ethnicity and race.

11.1.4 *Medical/surgical history*

Medical/surgical history will be obtained by patient interview and by access to patient medical records in order to verify that the eligibility criteria are met.

11.1.4.1 Disease specific parameters

The following parameters specific for the CRC will be recorded in the eCRF in association with screening in both Part I and Part II:

- Date of diagnosis
- TNM classification T-staging of tumour, N-staging of lymph node metastasis, M-staging of distant metastasis
- Localisation in bowel (right-sided: caecum, transversum, left-sided: distal transversum, sigmoideum, rectal)
- Localisation(s) of metastases (liver, lungs, peritoneal carcinosis, brain, skeleton, distant lymph nodes, other site)
- Histological type (classic adenocarcinoma, mucinous adenocarcinoma, other)
- Molecular parameters (if available)
    - Microsatellite stable/instable
    - BRAF mutation
    - KRAS mutation
    - NRAS mutation
    - PIK3CA mutation
- Whether the CRC is considered hereditary (Lynch syndrome [hereditary non-polyposis colorectal cancer, HNPCC], familial adenomatous polyposis [FAP], or other)
- Previous CRC treatments, see Section 11.1.7.1 for details.

11.1.5 *Serology*

Part I

Patients will be tested for HIV, human T-lymphotropic virus type 1 (HTLV), Hepatitis A, Hepatitis B, Hepatitis C, and Syphilis at the screening visit in Part I (Visit 1). The results must be in place before collection of starting material for pTTL production. Any positive result will exclude the patient from participating in the trial. Serology testing will be repeated at surgery (Visit 2) in line with standard procedures for GMP handling of human cells. The donor testing is performed in accordance with the EU directive 2006/17/EC.

Part II

Serology testing will again be repeated at the screening visit in Part II (Visit 3), with exception for Syphilis. Serology for cytomegalovirus (CMV), Epstein-Barr virus (EBV) and Varicella-Zoster virus (VZV) will be added in order to establish whether the patient is carrying any of these viruses. This information will be used to decide whether certain microbial prophylaxis (valaciclovir) is needed as part of supportive care, and to determine whether testing for reactivation (CMV and EBV) should be performed as part of the safety analyses in Part II, see Table 17.

Table 10 Serology parameters

Parameter	Part I		Part II
	Screening (Visit 1)	Surgery (Visit 2)	Screening (Visit 3)
Hepatitis A	X	X	X
Hepatitis B	X	X	X
Hepatitis C	X	X	X
HIV	X	X	X
HTLV	X	X	X
Syphilis	X	X	
EBV			X
CMV			X
VZV			X

11.1.6 *Weight and height*

Weight and height will be measured without shoes. Body mass index (BMI) will be calculated, with one decimal, from the height and weight recorded. Weight, height and BMI will be recorded in the eCRF at Visit 1. Thereafter, only weight will measured and recorded at the designated visits, see Table 4, Table 5, Table 6 and Table 7.

11.1.7 *Prior and concomitant medication*

Medications are classified as prior if the stop date was before the day of pTTL product administration and as concomitant if ongoing on the day of pTTL product administration, stopped after the pTTL product administration or started after the pTTL product administration.

11.1.7.1 Prior medications

All prior medications taken within 4 weeks before inclusion in Part I and within 4 weeks before inclusion in Part II will be recorded in the Prior/Concomitant Medications Log of the eCRF in association to the screening visit in each trial Part in order to verify that the eligibility criteria are met (see also Section 9.6) and for ensuring availability of pertinent information for assessments of potential interactions and late effects during the trial.

Special categories of prior therapy which should be registered also when administered outside of the 4 weeks period prior to inclusion in Parts I and II:

Prior CRC therapy

Previous CRC therapy should be registered in the eCRF without time-limit at inclusion in both Part I and Part II. Registration should, however, only include such medications that directly targets the cancer in each treatment line (for example fluorouracil and irinotecan in a 5-FU + irinotecan regimen). An exception to this rule is made for medications which directly target white blood cells and bone marrow function such as G-CSF (Zarzio, Neulasta, Neupogen etc) and corticosteroids (prednisolone, betamethasone, dexamethasone etc).

Each treatment line will be registered, with details on included anti-CRC medication and other relevant medications (G-CSF or corticosteroids), including given dose and whether the dose was modified according to the standard regimen. The number of treatments and the time during which the regimen was administered should also be registered, as well as the reason for discontinuing the regimen (if applicable).

Prior immunotherapy

Previous immunotherapy (defined as therapies that modifies immune system functions on a systemic level), as well as their side effects, should be registered in the eCRF without time limit. This includes immunotherapy against CRC according to above, but also immunotherapy used against other disorders.

The class of immunotherapy of most immediate importance includes treatments that stimulates immune function, such as checkpoint inhibitors. Side effects to this class of therapy includes immune-related disorders such as pneumonitis/pleuritis, colitis, hepatitis, arthritis/myositis, immune-related dysfunction of endocrine organs (thyroiditis, hypophysitis, type 1 diabetes, adrenal insufficiency or other), nephritis, autoimmune cytopenias (thrombocytopenia, haemolytic anaemia or other), and immune-related neurological side effects [each inflammatory neurological side effect to be specified] and other inflammatory disorders, and should be registered together with a severity grading. This is of special importance as previous moderate to severe side effects to immunotherapy could confer a risk of toxicity to pTTL and is an exclusion criterion.

Other immunotherapies which primarily causes immunosuppression, such as TNF-alpha inhibitors, corticosteroid treatment at a dose and duration which results in significant immunosuppression, low dose chemotherapy against autoimmune diseases, chemotherapy against other cancers, and other treatments with similar effects, should also be registered, as they may affect pTTL product quality and the effect of pTTL treatment. Potential side effects need not be registered unless they are autoimmunity-related.

Prophylactic treatment in association with surgery in the trial

Prior to surgery, prophylactic antibiotics are administered iv in accordance with standard of care. The dose and type of antibiotics is decided on an individual basis. In addition, also in accordance with standard of care, anticoagulants will be given post-surgery. Use of prophylactic antibiotics and post-surgery anticoagulants will be documented.

11.1.7.2 Concomitant medications

Use of concomitant medication from pTTL administration until end of Part II must be documented appropriately in the patient's eCRF. Relevant information (i.e. name of medication, dose, dose form, unit, route, frequency, start and stop dates, reason for use) should be recorded. All changes in medication should be noted in the eCRF.

Medications used on an as needed basis will be entered once in the eCRF (no stop date until medication is no longer used).

Use of infusion solutions such as saline solution must not be entered in the eCRF.

In Part III, only concomitant medications used for treatment of CRC progression should be entered into the eCRF.

11.1.8 ***Pregnancy test***

For each female patient, the investigator will document non-childbearing status (surgically sterile or post-menopausal for at least 1 year) or potential childbearing status. Patients with non-childbearing status do not require a pregnancy test. For pre-menopausal patients, a serum pregnancy test will be performed at the Part I and Part II screening visits, prior to treatment with pTTL (result must be available prior to the administration of pTTL) and at monthly intervals during the 26 weeks safety follow-up period, see Table 7.

11.1.9 *Eastern Cooperative Oncology Group (ECOG) performance status*

The disease status of the patients will be checked at each screening visit using the ECOG performance status scale. The scale includes 6 levels ranging from 0 (fully active, able to carry on all pre-disease performance without restriction) to 5 (dead). The ECOG result will be recorded in the eCRF.

11.1.10 *Covid-19 PCR test*

Covid-19 PCR test will be performed at visits indicated in Table 6 if required by the prevailing Covid-19 policy the Recruitment, Surgery & Treatment Site. In connection to surgery in Part I, Covid-19 PCR test is performed if required by, and according to, prevailing Covid-19 policy at the site where surgery is performed.

11.1.11 *Spirometry*

At the screening visits of Part I (patients scheduled for trial specific surgery) and Part II (all patients), spirometry will be performed (if indicated as outlined in exclusion criterion No 3 of each part) before inclusion in the trial. Measurements will be done in accordance with local hospital routines. FEV1 and diffusion capacity (DLCOco) will be recorded in the eCRF.

11.1.12 *Echocardiogram*

At the screening visits of Part I (required for patients scheduled for trial specific surgery) and Part II (required for all patients), an echocardiogram assessment will be performed before inclusion in the trial as per exclusion criterion No 2 of each part.

If an echocardiogram with acceptable results has been performed within 3 months prior to the screening visit in Part I (Visit 1) and/or prior to the start of pre-conditioning in Part II (Visit 4), respectively, these results can be used for assessment of exclusion criterion No 2 of each part at the discretion of the Investigator. A new examination is then not required. Measurements will be done in accordance with local hospital routines. Ejection fraction will be recorded in the eCRF.

11.2 Trial specific assessments

11.2.1 *Collection of tumour samples and normal samples for next generation sequencing (Part I)*

Suitable tumour material for whole exome sequencing (WES) and RNA sequencing (RNAseq) can be obtained either from biopsies or from surgically excised tumour material. In association with the biopsy/surgery, a blood sample is obtained for WES to use as a normal tissue control, see Section 11.2.2. Surgeries are performed at the Recruitment & Surgery Sites or at the Recruitment, Surgery & Treatment Site. Biopsies from tumour tissue may be performed at either one of the Recruitment & Surgery Sites or at the Recruitment, Surgery & Treatment Site.

The date of each procedure will be recorded in the eCRF as will the following data:

- surgical procedure/biopsy procedure performed (if tumour biopsy: surgically or by endoscopy)
    - If collection is performed during surgery, the type of surgery is recorded
    - If collection is performed by biopsy, biopsy type, percutaneous or by endoscopy, is recorded, and in the former case, if guided by ultrasound, CT etc.
- biopsy complications (bleeding, infection, pain, organ injury, unplanned surgical procedure etc) will be recorded in the AE Log of the eCRF.
- surgical complications, when tumour material have been collected at the same surgery as the RLN starting material, are reported with the reporting for the collection of material from RLNs for pTTL production, see Section 11.2.3.
- length of stay

Per operative complications (bleeding, organ injury, unplanned surgical procedure) will be recorded in the AE Log of the eCRF.

The following surgery/biopsy scenarios apply:

1. **Standard surgery for non-curative purposes**: **Bowel resection or deviating surgery (stoma) in metastatic patients with symptomatic primary tumour**

    The patient may be included in Part I and may be assessed for eligibility in Part II once the steps outlined in Section 9.2.3 are completed. RLNs and tumour material will then be collected during the same surgical procedure, see Section 11.2.3.

2. **Biopsy**

    Applicable for patients for whom no standard of care surgery is planned. Tumour samples will be achieved as biopsies in association with either colonoscopy, or by radiology- or palpation-guided biopsy of tumour lesions. The biopsy procedures and the trial specific surgical intervention for collection of RLN starting material may be performed on the same occasion or on different occasions, see Section 11.2.3. A second biopsy may be performed if the first biopsy did not render material that fulfil the quality requirements for extracting material for NGS analysis. This should be considered in a patient where a second biopsy is judged to have a good chance of yielding better material than the first. If the low quality of the first biopsy is judged to be due to patient-related factors, which make it less likely that a second biopsy would yield better material, or if other medical factors have arisen that make renewed biopsy unsuitable, the patient may be prevented from further participation in the trial due to inability to produce TC0301. Planned trial specific surgery should, in such a case, be cancelled.

11.2.2 *Collection of blood sample for next generation sequencing (normal control) (Part I)*

Concomitantly with the collection of tumour material (Section 11.2.1), a blood sample is obtained for WES to use as a normal tissue control. The peripheral blood sample is collected in a designated DNA/RNA collection tube and subsequently sent for sequencing. One sample tube is sent to the Sponsor and kept as a back-up sample until successful NGS analysis has been confirmed. The date of collection of each sample will be recorded in the eCRF.

11.2.3 *Collection of material from regional lymph nodes for pTTL production (Part I)*

The pTTL starting material is based on RLNs excised from the area anatomically close to, and known to receive lymphatic drainage from, the area where the selected tumour lesion (primary tumour or metastasis) is located. The RLN starting material collection can be performed in association with the standard surgery procedure outlined in Section 11.2.1 or in a trial specific surgery in case no standard of care surgery is planned (see below).

<u>*RLN collection in case of standard surgery for non-curative purposes*</u>

During standard care surgeries, sentinel node detection methods may be performed to localise tumour-draining lymph node(s), if judged technically feasible. This node or nodes are to be used preferentially in the lymph node pool used for pTTL manufacturing. An adequate number of RLNs may be collected, as judged by the Investigator. Detailed instructions for the retrieval of RLNs are given in the surgery manuals.

The date of each procedure will be recorded in the eCRF as will the following data:

- Type of surgical procedure performed.
- Operating time.
- Per operative blood loss.
- Number of harvested nodes and their location in respect to closet large blood vessel (close to left colic artery, inferior mesenteric artery, sigmoid artery and so on).
- Total number of identified nodes.
- If sentinel node detection methodology was applied, whether detection of tumour-draining lymph nodes was successful, and which of the harvested nodes were identified as tumour-draining.
- Per operative complications will be recorded in the AE Log of the eCRF.
- Postoperative complications according to Clavien Dindo (Grade 0, I, II, III, IV or V).
- Length of hospital stay.

<u>*RLN collection in case of trial specific surgery*</u>

A trial specific surgery will be performed in case that no standard of care surgery is planned. Medical imaging (MRI or CT) will be performed at screening, if no satisfactory imaging data are available from the patient's standard of care, to ensure that there are accessible RLNs prior to the surgery. At least one lymph node accessible by surgery must be identified. The decision on whether it is feasible to retrieve starting material, and, if so, the optimal surgical method, will be based on the status of the lymph nodes, e.g. if assessed as metastatic or if enlarged, the lymph nodes' accessibility by surgery, and consideration of radiological signs of peritoneal carcinomatosis, which may obstruct RLN retraction. The assessment is performed by radiologist(s), surgeon(s), and the coordinating PI. A stop-go decision from the Sponsor regarding successful NGS analysis allowing manufacturing of TC0301 is a prerequisite for executing the trial specific surgery. The surgical procedure will be minimally invasive according to existing conditions, including previous surgery and lymph node location, and in order to collect sufficient RLN starting material for pTTL manufacture. Surgical methods for trial specific surgery include minimally invasive (laparoscopic) lymphadenectomy, and minimally invasive resection of the bowel segment containing the primary tumour, with the option of conversion to a laparotomy if required. The aim of the procedure is extirpation of approximately 6-10 RLNs (at the judgement of the Investigator), in case of minimally invasive lymphadenectomy, or an adequate number of RLNs at the discretion of the

Investigator, in case of resection of bowel segment. The surgical method judged most suitable to obtain this goal will be performed. If the intended surgical method involves resection surgery, the iSRC will assess the appropriateness of the method before the surgery may be performed, see Section 8.4.3. For further details and risks associated with the trial specific surgery procedure, see Section 6.6.2.6. Detailed instructions for the collection of RLNs are found in the Surgery manuals.

The trial specific surgeries to obtain starting material are performed the Recruitment, Surgery and Treatment site or at one of the Recruitment & Surgery Sites.

The date of each procedure will be recorded in the eCRF as will the following data:

- Type of surgical procedure performed.
- Operating time.
- Per operative blood loss.
- Number of harvested nodes and their location in respect to closet large blood vessel (close to left colic artery, inferior mesenteric artery, sigmoid artery and so on).
- Complications to the performance of the procedure (no identified node, unplanned change of surgical procedure).
- Per operative complications (bleeding, organ injury, complications to anaesthesia and other acute complications) will be recorded in the AE Log of the eCRF.
- Postoperative complications according to Clavien Dindo (Grade 0, I, II, III, IV or V).
- Length of hospital stay.

11.2.4 *Pre-conditioning and supportive care therapy*

The chemotherapy protocol was originally adapted from a standard regime for chronic lymphatic leukaemia (CLL; Schiavone et al. 2003), and the version used in this trial is a pre-conditioning regimen used in several CAR T cell trials (Berdeja et al. 2021; Cappell et al. 2020). Supportive care therapies have been based on standard protocols and include antiemetic therapy, antimicrobial prophylaxis, prophylaxis against tumour lysis syndrome (TLS) and uroprotective measures in conjunction with administration of chemotherapy.

All treatments will be recorded in the concomitant medications Log of the eCRF.

Supportive care, Day -7

Patients are assessed for high risk of TLS. TLS is very rare in CRC, but the following general risk factors should be considered:

1. High tumour burden
2. High grade tumours with rapid cell turnover
3. Pre-existing renal impairment or renal involvement by tumour
4. Increased age
5. Concomitant use of drugs that increase uric acid levels, for example: aspirin, caffeine, thiazide diuretics, ethambutol, levodopa, methyldopa, nicotinic acid, pyrazinamide, phenothiazines and theophylline.

In cases considered higher risk for TLS than the general CRC population, prophylaxis (e.g., allopurinol, 300 mg orally, once daily) can be started on Day -7 and is suggested to go on

until Day 7 or according to individual assessment by the Investigator. Additional therapies can be considered as per local routine.

Supportive care, Day -6

If preventive therapy against urothelial toxicity from cyclophosphamide is judged to be indicated, treatment can be started on Day -6 or Day -5 depending on whether uromitexan is given alone or in combination with sodium bicarbonate. Sodium bicarbonate is usually started on the day before administration of cyclophosphamide while uromitexan is commonly initiated on the same day.

Pre-conditioning, Day -5 to Day -3

- 1.0 L of intravenous fluid therapy with a crystalloid infusion solution, for example Ringer-Acetate or 0.9% NaCl, given, if possible, at 500 mL/hour, with a suggested start at 10.00.
- Cyclophosphamide 300mg/m^2 IV over 60 minutes, starting approximately 2 hours after the start of the crystalloid infusion. If the crystalloid infusion cannot be administered in 2 hours, the infusions can be parallel.
- Fludarabine 30mg/m^2 IV over 30 minutes, starting after Cyclophosphamide have been administered.
- 1.0 L of intravenous fluid therapy with a crystalloid solution, for example Ringer-Acetate or 0.9% NaCl, given, if possible, at 500 mL/hour, starting after Fludarabine has been administered, or when the previous crystalloid infusion is finished, depending on which occurs last.

Hydration may be adjusted as clinically indicated, for example, adjustment to a slower infusion rate can be considered in patients with reduced heart or kidney function, or other relevant conditions. Patients should be instructed to drink plenty of liquids during and for 24 hours following the chemotherapy (approximately 2 L/24 hours).

Supportive care, from Day -5

Prophylaxis against TLS, is either ongoing since Day -7 or initiated on Day -5, and discontinued according to clinical routine.

If preventive therapy against urothelial toxicity from cyclophosphamide has been judged to be indicated, this continues according to clinical routine.

- Antiemetic therapy, for example with a combination of a serotonin 5-HT3 receptor antagonists such as ondansetron and a steroid such as Betapred, in combination with antiemetic medications from additional drug classes as needed, is given according to clinical routine. However, steroids cannot be given after Day -3.
- Pneumocystis prophylaxis with, as the first choice, an oral trimethoprim and sulfamethoxazole combination drug, is administered on Mondays, Wednesdays and Fridays, starting day and dose to be selected according to institutional guidelines. A pause between pTTL administration and end of post chemotherapy leukopenia can, for example, be utilised. Other agents, such as Pentamidine, can be used if judged preferable due to, for example, allergy or reduced kidney function. Treatment is continued until the risk of pneumocystis pneumonia is considered very low.
- Antifungal prophylaxis, with for example, fluconazole, can be indicated during neutropenia and is administered as per routine practise when ANC $< 0.5 \times 10^9$/L until ANC is $>0.5 \times 10^9$/L, or when otherwise clinically indicated.

- Valaciclovir is initiated orally on Day -5 at a dose selected by the Investigator and continued until the risk of virus reactivation is judged to have resolved, in accordance with clinical routine.
- Antibacterial prophylaxis with, for example, Ciprofloxacin, is indicated during neutropenia and is administered as per routine practise when ANC < 0.5 x 10^9/L until ANC is >0.5 x 10^9/L, or when otherwise clinically indicated.

.

Table 11 Pre-conditioning therapy and supportive care overview

	Visit 4							Visit 5							
	Out-patient care		In-patient or out-patient care					In-patient care							
**Therapy**	Day-7	Day-6	Day-5	Day-4	Day-3	Day-2	Day-1	Day 1	Day 2	Day 3	Day 4	Day 5	Day 6	Day 7	Day 8
Cyclophosphamide			X	X	X										
Fludarabine			X	X	X										
Iv hydration			X	X	X										
Prevention of urothelial toxicity		(X)[1]	(X)[1]	(X)[1]	(X)[1]	(X)[1]									
Antiemetic prophylaxis			X	X	X	(X)[1]	(X)[1]								
Prophylaxis against TLS	(X)[1]	(X)[1]	X	X	X	X	X	(X)[1]	(X)[1]	(X)[1]	(X)[1]	(X)[1]	(X)[1]	(X)[1]	
Pneumocystis pneumonia prophylaxis			X			X		(X)[1]			(X)[1]		(X)[1]		(X)[1]
Antifungal prophylaxis								(X)[1]	(X)[1]	(X)[1]	(X)[1]	(X)[1]	(X)[1]	(X)[1]	(X)[1]
Valaciclovir			X	X	X	X	X	(X)[1]	(X)[1]	(X)[1]	(X)[1]	(X)[1]	(X)[1]	(X)[1]	X[1]
Antibacterial prophylaxis (if neu <0,5)								(X)[1]	(X)[1]	(X)[1]	(X)[1]	(X)[1]	(X)[1]	(X)[1]	(X)[1]

Supportive care treatment starts on Day -7 or Day -6 and continues until Day -1. Patients may be homebased between end of chemotherapy on Day -3 and Day -1 as judged by the Investigator and will in that case come to the Recruitment, Surgery & Treatment Site for Covid-19 testing (if applicable) and pre-dose safety laboratory sampling on Day -1 (corresponding to the pre-dose large safety panel on Day 1, see Table 5). If considered feasible by the Investigator, and if allowed by the prevailing Covid-19 policy at the Recruitment, Surgery & Treatment Site, patients may also leave the clinic after end of each chemotherapy session.

1. Therapy given or omitted as per Investigators choice.

11.2.5 *Biomarkers*

Exploratory biomarker analysis will be performed using 3 sources of material: pTTL, tumour and blood, respectively, as outlined in the sections below. Details of sample handling and type of biomarker analysis to be performed will be provided in separate manuals. The date of collection of each sample will be recorded in the eCRF. The result of serum tumour marker analysis during Parts II-III will also be registered in the eCRF.

11.2.5.1 Biomarkers: pTTL

The pTTL biomarker analysis schedule and planned analyses are given in Table 12.

Table 12 Biomarker analysis schedule: pTTL (Part I and pTTL manufacturing)

**Timing**	**Part I, Visit 2**	**pTTL manufacturing**	
**Analysis**	**Starting Material**	**In process**	**pTTL product**
TCRseq	X		X
Phenotype (e.g. FACS, RNA sequencing, Treg analysis)	X	X	X
Functional assays	X		X
Protein/cytokine analysis	X	X	X

11.2.5.2 Biomarkers: tumour

Part I: Exploratory collection of tumour and normal samples for functional assays may be performed in association with standard surgeries and trial specific bowel resections. The procedure will not be applicable for patients undergoing trial specific laparoscopic or minimally invasive lymphadenectomy. Details on sample handling will be described in a separate manual.

Part II: Tissue biopsies from RECIST non-target lesions are strongly encouraged unless the risk to the patient is judged to be greater than the potential benefit. Fine needle biopsies are not allowed. If possible, several core biopsies should be taken, and the same target lesion biopsied both before and after pTTL administration. The biopsy should be performed with the method deemed suitable based on the size and anatomical location of the lesion targeted. Based on this, biopsy needle/implement type and size are selected and a decision regarding whether it is suitable to use radiology-based guidance is made.

The tumour biomarker analysis schedule and planned analyses are given in Table 13. The immunohistochemistry analysis is the main analysis during Part II, the TCRseq and T cell phenotype analysis will be performed according to availability of tumour material.

Table 13 Biomarker analysis schedule: tumour

**Part**	**Part I**	**Part II**	
**Visit**	**Visit 2**	**Visit 3**	**Visit 11**
**Analysis**	**Surgery**	**Screening**	**Week 4<br>Day 28 ±7 days**
Functional assays	X		
IHC for T cell infiltration		X	X
TCRseq		X	X
Phenotypic analysis		X	X

11.2.5.3 Biomarkers: blood

The blood biomarker analysis schedule and planned analyses are given in Table 14 (Part I and Part II) and in Table 15 (Part III).

Table 14 Biomarker analysis schedule Part I and Part II: blood

**Part**	**Part I**	**Part II**								
**Visit**	**V1**	**V3**	**V4**	**V5**	**V7**	**V11**	**V13**	**V16**	**V18**	**V19**
	Screen.	Screen.	W-1	W1	W2	W4	W6	W12	W18	W24
Analysis		Day -28 to Day -8	Day -7[1]	Day 7	Day 14 ±1 day	Day 28 ±1 day				
TCRseq		X		X	X	X	X	(X)[2]	(X)[2]	(X)[2]
Phenotype, limited	X[3]			X			X			X
Phenotype, large		X			X	X				
Functional analysis	X[3]				X	X				
Plasma/serum protein analysis		X		X	X	X	X	X	X	X
Tumour burden analysis: ctDNA			X				X	X	(X)[4]	(X)[4]
Tumour burden analysis: serum tumour markers[5]			X				X	X	X	X

1. Sampling before start of pre-conditioning on either of Day-7, Day-6 or Day-5.
2. Excluded if no pTTL clone detected in previous samples.
3. May be performed at Visit 1 or Visit 2.
4. Excluded if not considered to add any information in excess of what is gained by serum tumour markers and RECIST.
5. Result of analysis must be documented in eCRF.

Table 15 Biomarker analysis schedule Part III: blood

**Visit**		**V21**	**V22**	**V23**	**V24**	**V25**	**V26**	**V27**
**Analysis**		**9 months**	**12 months**	**18 months**	**24 months**	**3 years**	**4 years**	**5 years**
TCR sequencing		(X)[1]	(X)[1]	(X)[1]	(X)[1]	(X)[1]	(X)[1]	(X)[1]
Phenotype, large		X	X	X	X	X	X	X
Functional analysis		X	X	X	X	X	X	X
Plasma/serum protein analysis		X	X	X	X	X	X	X
Tumour burden analysis	ctDNA	(X)[1]	(X)[1]	(X)[1]	(X)[1]	(X)[1]	(X)[1]	(X)[1]
	serum tumour markers[2]	X	X	X	X	X	X	X

1. If applicable based on previous results.
2. Result of analysis must be documented in eCRF.

11.2.6 *Medical imaging and iRECIST*

Patients will undergo a baseline CT of the thorax/abdomen/pelvis at a maximum of 28 days prior to treatment start. MRI examinations may replace CT when judged to be appropriate. A baseline MRI of the brain must have been performed within 4 weeks of starting treatment in cases of previously treated CNS metastases. Additional medical imaging such as scintigraphy, PET-CT etc. can also be performed as part of radiological evaluation as deemed appropriate.

Tumour lesions are categorised as measurable and/or non-measurable lesions using RECIST 1.1 criteria (Eisenhauer et al., 2009). Measurable disease includes lesions that can be measured in at least one dimension and which have not been previously irradiated. The longest diameter (LD) must be at least 10 mm as measured with a CT scan. Measurable lesions that consist of pathologically enlarged lymph nodes must be at least 15 mm at the short axis to be considered measurable.

Non-measurable disease includes lesions such as bone metastases, ascites, pleural/pericardial effusion and all other lesions that have not reached adequate size as referred to above.

Measurable lesions consist of up to a maximum of 2 lesions per organ and 5 lesions in total. They are identified and recorded at baseline medical imaging. A sum of the LD for all target lesions is calculated and reported as the baseline sum LD. The baseline sum LD will be used as the reference by which to characterise the objective tumour response.

Non-target lesions are recorded at baseline and in follow-up as present or absent.

Tumour response and progression assessments will be conducted using the iRECIST criteria (Seymour et al., 2017) as the primary measurement, while RECIST 1.1 will be used primarily for evaluating disease burden at baseline. The main criterion for tumour response endpoints will be an assessment according to iRECIST. If imaging indicates progressive disease (PD) in a clinically stable participant, a follow-up confirmation scan in accordance with iRECIST guidelines must be carried out within a timeframe of no less than 4 weeks and no more than 8 weeks following the initial assessment.

For target-lesions:

Complete response (CR): The disappearance of all target lesions (lymph nodes must be < 10 mm).

Partial response (PR): At least a 30 % decrease in the sum of the LD of target lesions, taking as reference the baseline sum LD (including lymph node short-axis if applicable).

Stable disease (SD): Neither sufficient shrinkage to qualify for PR or progressive disease

Progressive disease (PD): At least a 20 % increase in the sum of the LD of target lesions, taking as reference the smallest sum LD recorded since the treatment started or the appearance of one or more new lesions.

For non-target lesions:

CR: The disappearance of all non-target lesions.

SD: The persistence of one or more non-target lesion(s).

PD: The appearance of one or more new lesions and/or unequivocal progression of existing non-target lesions.

Immune therapies may result in an inflammatory process in target and non-target lesions. This makes interpretation of iRECIST measurements more difficult and has resulted in the term *pseudoprogression.* Pseudoprogression refers to the possibility that a target lesion may appear larger compared to baseline but this may be due to inflammation and not real disease progression. The same can be true of new lesions. New measurable lesions are added to the sum of target lesions. With iRECIST, it is necessary to repeat the medical imaging at least 4 weeks later to confirm if disease progression is real or not.

The following will be recorded in the eCRF:

Baseline:

- Number of target lesions (maximum 5 per patient and maximum 2 per organ)
- Number of non-target lesions (unlimited)
- Localisation of lesions (target and non-target lesions)
- LD per target lesion
- Sum of LDs (target lesions)

Post-treatment visits:

- Number of target lesions.
- Number of non-target lesions (new and previous).
- Localisation of lesions (target and non-target lesions).
- LD per target lesion.
- Sum of LDs (target lesions).

In addition, at each post-treatment imaging visit, the overall response will be summarised as shown in Table 16.

Table 16 Criteria for best overall response

Target Lesions	Non-Target Lesions	New Lesions	Overall Response
CR	CR	No	iCR
CR	SD	No	iPR
PR	Non-PD	No	iPR
SD	Non-PD	No	iSD
PD	Any	Yes or No	iUPD/iCPD
Any	PD	Yes or No	iUPD/iCPD
Any	Any	Yes	iUPD/iCPD

11.3 Safety assessments

11.3.1 *Adverse events*

The Principal Investigator at each site is responsible for ensuring that all medical staff involved in the trial is familiar with the content of this section and the content of the applicable standard operating procedures (SOPs) regarding emergencies and FIH studies.

11.3.1.1 Definition of adverse event

An AE is defined as any untoward medical occurrence in a patient administered a medicinal product or exposed to a trial specific surgery/biopsy/treatment and which does not necessarily have a causal relationship with this treatment. An AE can therefore be any unfavourable and unintended sign (including an abnormal laboratory finding), symptom, or disease temporally associated with the use of a medicinal product, whether or not considered related to the medicinal product.

Complications during trial surgery(ies)/biopsies will be reported as AEs or SAEs, see also Section 11.3.1.9 for information about assessment of causality.

11.3.1.2 Definition of serious adverse event

An SAE is any AE which:

- results in death
- is life-threatening (this refers to a reaction in which the patient was at risk of death at the time of the reaction; it does not refer to a reaction that hypothetically might had led to death if the reaction was more severe)
- requires in-patient hospitalisation or prolongation of existing hospitalisation
- results in persistent or significant disability/incapacity
- is a congenital anomaly/birth defect
- is an important medical event (IME) (this refers to a reaction that may not be immediately life-threatening or result in death or hospitalisation, but may jeopardise the patient or may require intervention to prevent any of the other outcomes defined above)

Examples of IMEs are intensive treatment in an emergency room for allergic bronchospasm or blood dyscrasias, convulsions that do not result in hospitalisation, development of drug dependency, and drug abuse.

Planned hospitalisations or surgical interventions for a condition that existed before the patient signed the ICF and that did not change in intensity are not SAEs.

Complications during trial surgery(ies)/biopsies will be reported as AEs or SAEs, see also Section 11.3.1.9 for information about assessment of causality.

AE intensity (severity) is graded using CTCAE v5.0, which defines Grade 4 as “life-threatening consequences; urgent intervention indicated”, see Section 11.3.1.8. However, while CTCAE Grade 4 indicates a high level of severity, it does not automatically mean that the event must be classified as serious. The assessment of seriousness follows regulatory definitions, which are distinct from CTCAE severity grading. An AE is considered serious if it fulfils at least one of the SAE criteria defined above.

If there is any doubt as to whether an AE meets the definition of an SAE, a conservative viewpoint must be taken, and the AE must be reported as an SAE.

11.3.1.3 Definition of adverse reaction

The term AR is to be used whenever either the Investigator or Sponsor or designee assessed the AE as at least possibly related to the IMP.

11.3.1.4 Definition of serious adverse reaction

The term SAR is to be used whenever either the Investigator or Sponsor or designee assessed the SAE as at least possibly related to the IMP.

11.3.1.5 Definition of suspected unexpected serious adverse reaction

A suspected unexpected SAR (SUSAR) is any SAE that has a suspected causal relationship with the IMP (assessed as related by the Sponsor or Investigator), but the nature of which is not consistent with the applicable product information in the IB, and is therefore assessed as unexpected.

11.3.1.6 Definition of adverse events of special interest

AEs of special interest (AESI) in this trial will be immune-related toxicities. Reporting procedures for AESI are outlined in Section 11.3.1.16.

The AESI definition includes autoimmune reactions that could be a result of off-target toxicity mediated by pTTL T cells, such as colitis, thyroiditis, pneumonitis and other inflammatory disorders. CRS, ICANS and other immune-mediated, but not strictly classified as autoimmune, reactions are also included in the AESI definition. Such AEs have been described in other T cell immunotherapies (as discussed in other sections of the protocol, including Section 6.6.2.5) and are closely linked to activation and function of adoptively transferred T cells. The AESI definition is based on the need to collect and analyse AEs with a potential direct relationship with the functionality of the infused pTTL, especially the T cells, which is central in evaluating the primary endpoint, the safety of pTTL administration. Also of note is that this type of AE occurs in consequence of activation and functionality of the infused immune cells. This means that AESI might, speculatively, be

associated with treatment effect. In such a case, evaluation of AESI will need to be made in the context of potential benefits.

The treatment of immune-related toxicity with immunosuppressive agents, including steroids and cytokine inhibitors such as anti-TNFa and anti-IL-6 agents are allowed when judged necessary, but should, when possible, be performed after discussion with the Investigators at the Recruitment, Surgery & Treatment Site and Sponsor's Medical Representative, and with their agreement to the projected therapy. Further details regarding the trial policy on such therapies are provided separately.

11.3.1.7 Time period and frequency for collecting adverse events

AEs will be collected from start of the first surgery or biopsy, whichever comes first, at Visit 1/2 in Part I until the last visit in Part II. For the time period between the surgery/biopsy in Part I and the start of Part II, the duration of which may vary in length between patients, the following events will be recorded as AEs:

- Any event assessed by the Investigator as related to any of the trial procedures.
- Any other event assessed by the Investigator as relevant for the interpretation of trial results.

Any AE with start date on the day of pTTL administration must be recorded with start time.

At the last visit in Part II, information on new AEs or SAEs, if any, and stop dates for ongoing events must be recorded as applicable. Since immunotherapies may give rise to AEs long after treatment, AESI will be recorded also during the long-term follow-up part of the trial (Part III), see Section 8.1.3.

Baseline conditions that worsen in severity or frequency during the trial should be recorded and reported as AEs or as medical history (the latter if it occurs prior to administration of surgery/biopsy)

11.3.1.8 Assessment of intensity

The grading of the intensity of AEs will follow the CTCAE v5.0. Grade refers to the severity of the AE. The CTCAE displays grades 1 through 5 with unique clinical descriptions of severity for each AE based on this general guideline. The intensity of an AE can change over time.

The evaluation of AE intensity is separate from the assessment of whether an AE should be classified as an SAE. A severe (grade 3 or grade 4) AE should not necessarily be classified as an SAE. The SAE definition is found in Section 11.3.1.2.

The Investigator must assess the intensity of an AE using the following definitions, and record it on the AE Log in the eCRF:

**Grade 1**	Mild; asymptomatic or mild symptoms; clinical or diagnostic observations only; intervention not indicated.
**Grade 2**	Moderate; minimal, local or non-invasive intervention indicated; limiting age-appropriate instrumental activities of daily living (ADL)*.
**Grade 3**	Severe or medically significant but not immediately life-threatening; hospitalisation or prolongation of hospitalisation indicated; disabling; limiting self- care ADL**.

**Grade 4**	Life-threatening consequences; urgent intervention indicated.
**Grade 5**	Death related to AE.

**Instrumental ADL refer to preparing meals, shopping for groceries or clothes, using the telephone, managing money, etc.*

***Self- care ADL refer to bathing, dressing and undressing, feeding self, using the toilet, taking medications, and not bedridden.*

11.3.1.9 Assessment of causal relationship

The Investigator must assess the causal relationship between an AE and pTTL and record it in the AE Log of the eCRF using the definitions below:

**Probable**	The event has a strong temporal relationship to the IMP or recurs on re-challenge, and another aetiology is unlikely or significantly less likely.
**Possible**	The event has a suggestive temporal relationship to the IMP, and an alternative aetiology is equally or less likely.
**Unlikely**	The event has no temporal relationship to the IMP or is due to underlying or concurrent illness or effect of another drug (i.e., there is no causal relationship between the IMP and the event).

An AE is considered causally related to the use of pTTL when the causality assessment is probable or possible.
In addition to causality assessments in relation to pTTL, causality will also be assessed in relation to:

- Trial procedures (e.g. surgery, biopsy, medical imaging, pTTL administration process)
- Pre-conditioning and supportive care therapy
- Underlying disease

11.3.1.10 Assessment of outcome

The Investigator must assess the outcome of an AE using the definitions below and record it on the AE Log of the eCRF:

**Recovered/resolved**	The patient has recovered completely, and no symptoms remain.
**Recovering/resolving**	The patient's condition is improving, but symptoms still remain.
**Recovered/resolved with sequelae**	The patient has recovered, but some symptoms remain (for example, the patient had a stroke and is functioning normally but has some motor impairment).
**Not recovered/not resolved**	The patient's condition has not improved and the symptoms are unchanged (for example, an atrial fibrillation has become chronic).
**Fatal**	
**Unknown**	

11.3.1.11 Reporting of action taken with trial treatment

The Investigator must report the action taken with trial treatment using the definitions below and record it on the AE Log of the eCRF:

**Dose increased**	*Not applicable*
**Dose not changed**	
**Dose rate reduced**	
**Dose reduced**	*Not applicable*
**Drug interrupted**	
**Drug withdrawn**	*Not applicable*
**Not applicable**	
**Unknown**	

11.3.1.12 Collecting adverse events

AEs identified using any of the following methods will be recorded:

- AEs spontaneously reported by the patient
- AEs observed by the Investigator or medical personnel
- AEs elicited based on non-leading questions from the Investigator or medical personnel

To elicit AEs, questioning at each trial visit should begin with simple non-leading questions. For example:

- How have you felt since your last visit?
- Have you had any health problems since you were here last?

If a physician not involved with the trial sees a patient in relation to an AE, the physician should make every effort to contact the treating Investigator in a timely manner in order to obtain all information necessary to appropriate reporting of the event.

Information regarding AEs can also be derived from medical examinations such as the scheduled safety laboratory tests and radiology or a combination of sources.

11.3.1.13 Recording of adverse events

AEs must be recorded in the AE Log of the eCRF. The Investigator must provide information on the AE, preferably with a diagnosis or at least with signs and symptoms; start and stop dates, start and stop time; intensity; causal relationship to IMP; action taken, and outcome. If the intensity of an AE changes over time, this must be recorded as well.

If the AE is serious, this must be indicated in the eCRF.

If the AE is an AESI, this must be indicated in the eCRF.

An AE may be both of special interest and serious.

AEs, including out-of-range clinically significant clinical safety laboratory values, must be recorded individually, except when considered manifestations of the same medical condition or disease state; in such cases, they must be recorded under a single diagnosis.

11.3.1.14 Reporting of serious adverse events

SAE reporting must be performed by the Investigator within 24 hours of awareness via the eCRF. All available information regarding the SAE should be entered in the AE Log of the eCRF (i.e. term, intensity, causality, outcome, SAE criteria, action taken, narrative including rational for causality assessment). By saving the event as “serious” in the eCRF, and once the Investigator has signed-off of the event, an e-mail alert is automatically sent to predefined recipients to highlight that an SAE has been registered. The same information is automatically sent to sae@ctc-ab.se (with a copy to the Sponsor).

The SAE report is reviewed by a designated person at CTC’s Pharmacovigilance (PV) department to ensure that the report is valid and correct. For fatal or life-threatening SAEs where important or relevant information is missing, immediate follow-up is undertaken and queries to the site are raised. Investigators or other site personnel should inform CTC’s PV department of any follow-up information (including rational for changes, e.g. changes in causality assessment and intensity, that should be described in the SAE narrative) on a previously reported SAE immediately but no later than within 24 hours of awareness.

If the SAE report in the eCRF is updated and signed by the Investigator, a new e-mail alert will be sent.

If any additional documentation is required (e.g. autopsy report), CTC PV will request this information from the trial site.

In case the eCRF cannot be accessed, the SAE should be reported by manual completion of the paper SAE Form, provided in the Investigator Site File (ISF). The completed, signed and dated paper SAE Form should, within 24 hours, be scanned and delivered via encrypted e-mail or secure file transfer to CTC’s SAE inbox at sae@ctc-ab.se.

The trial site should notify the site Monitor via phone or e-mail about the submission of the SAE report. As soon as the site personnel have access to the eCRF, the SAE should be reported electronically as well.

The Sponsor or delegate will assume responsibility for reporting SAEs to the CA and IEC in accordance with local regulations.

11.3.1.15 Reporting of SUSARs to EudraVigilance, local CA and IEC

The term SAR is used whenever either the Investigator or Medical Monitor deems an SAE as possibly or probably related to pTTL. If a SAR is assessed as unexpected based on the Reference Safety Information (RSI) in the IB, it is a SUSAR and the EudraVigilance reporter will report the SUSAR to the CA, via the EudraVigilance database, and to the IEC in accordance with local regulations and CTC SOPs within the following timelines:

- 7 calendar days if fatal or life-threatening
- 15 calendar days if non-fatal and non-life-threatening

The clock for expedited initial reporting (Day 0) starts as soon as the Sponsor has received the information containing the minimum reporting criteria. The date must be documented on an acknowledgement receipt.

The Medical Monitor is responsible for medical review of the SAE narrative in the Council for International Organisations of Medical Sciences (CIOMS) form (or equivalent) prior to expedited reporting.

The Sponsor is responsible for informing the Investigators concerned of relevant information about SAEs and SUSARs that could adversely affect the safety of patients.

The Sponsor or delegate is responsible for, once a year throughout the clinical trial (or on request), submitting a safety report to the CA and the IEC taking into account all new available safety information received during the reporting period.

11.3.1.16 Reporting of adverse events of special interest

If the AESI is assessed as serious, reporting procedures for SAEs will apply, see Section 11.3.1.14.

Reporting of AESI should be performed by the Investigator, preferably within 24 hours of awareness, in the eCRF. All available information regarding the AESI should be entered (i.e. term, intensity, causality, outcome, action taken, narrative including rational for causality assessment). By saving the event “AESI” in the eCRF, an e-mail alert is automatically sent to predefined recipients to highlight that an AESI has been registered. If the AESI report in the eCRF is downgraded, a new e-mail alert will be sent.

11.3.1.17 Management of adverse events

A brief summary of reasonably expected AEs are given below. Details on each potential reaction, including detailed protocols for their management, are provided in a separate AE management manual (Appendix 1, Management of Adverse Event Manual, Version 1.0 06JUN2022).

Acute infusion-related ARs, including fever, skin reactions, allergic reactions and hypo/hypertension, are treated according to clinical practice at ME CAST. However, the use of steroids, which is a standard component of such treatment, will be avoided if at all medically feasible. The severity of the reaction will be evaluated and the need for steroid assessed in each case individually.

The management of immune-related AEs, in this trial defined as AESI, might necessitate immunosuppressive drugs. Previous experience with checkpoint blockade-induced pneumonitis and hepatitis have shown good effect of steroids, while for colitis, TNF-alpha blockade and a4b7 integrin blockade have been used with success as well as steroids which could indicate that steroids potentially could be avoided in certain cases of immune-mediated toxicity. In the majority of autoimmune side-effects, steroids are considered part of the standard of care, and appropriateness of use, suitable administration and dose, and advantages and disadvantages will be evaluated in each observed case of autoimmunity, especially in moderate to severe cases. The final decision is made by the Coordinating Investigator and Sponsor’s medical representative with advice from different medical specialist consultants as needed.

Autoimmune toxicity, such as colitis, thyroiditis, hepatitis, pneumonitis and other inflammatory reactions, will be managed according to standard protocols developed for management of such AEs in association with check-point inhibitor and also according to standard treatment guidelines for the disorder outside of the immunotherapy setting, after an evaluation have been performed regarding the appropriateness of steroid administration according to the discussion above.

Management of CRS and ICANS will be performed according to the guidelines for management and diagnosis of these AE setup for CAR T cell therapy. The same applies also to macrophage activation syndrome/hemophagocytic lymphohistiocytosis (MAS/HLH).

Details will be specified in the AE management manual (Appendix 1). The appropriate measures, including tocilizumab in the case of moderate to severe CRS, will be held available throughout the trial.

TLS will be managed according to standard of care. So also, will any other AE that is not judged as an AESI or deemed to be immune-related. The patients in this trial will all have advanced stage CRC, and cancer-related complications is expected, and will be managed according to institutional standard practises. This also applies to expected chemotherapy side effects, including nausea, affected renal and liver function, electrolyte imbalances, urinary tract toxicity and skin reactions.

11.3.1.18 Follow-up of adverse events

AEs must be followed up until resolution or until assessed as stable, whichever comes first. At the last visit in Part II, information on new AEs, if any, and stop dates for previously reported AEs must be recorded (if known). AEs assessed as stable by the Investigator at the end-of- trial visit of Part II will not have to be followed up until resolution.

It is the responsibility of the Investigator to follow-up on all SAEs and AESI until the patient has recovered, stabilised, or recovered with sequelae, and to report to the Sponsor all relevant new information using the same procedures and timelines as those for the initial report. Relevant information includes discharge summaries, autopsy reports, and medical consultation.

11.3.1.19 Procedures in case of pregnancy

In case of pregnancy or suspicion of possible pregnancy of any patients in Part II (after pTTL administration), the patient may continue in the trial.

Pregnancy itself is not regarded as an AE unless there is a suspicion that the IMP may have interfered with the effectiveness of the contraceptive medication. However, the outcome of all pregnancies (spontaneous miscarriage, elective termination, normal birth or congenital abnormality) must be followed up and documented even after the patient chose to withdraw from the trial. The outcome of all pregnancies of female trial participants or a female partner to a male patient, must also be followed up and documented, but the male patient does not need to be discontinued from the trial.

All events of congenital abnormalities/birth defects are SAEs. Spontaneous miscarriages should also be reported and handled as AEs. All outcomes of pregnancy must be reported to the Sponsor and the Principal Investigator on the pregnancy outcomes report form.

11.3.1.20 Treatment of overdose

An overdose is a dose in excess of the dose limit specified in this CTP. Over-dosing is not likely to occur in this trial since all IMP will be formulated as single doses. Overdoses could thus only occur as a result of formulation of a larger dose than allowed by the product specification. Overdoses, if they should occur after a formulation error, must be documented in the eCRF. An overdose with associated AE will be recorded as the AE diagnosis/symptoms in the AE Log of the eCRF and in the protocol deviation Log. An overdose without associated symptoms will only be reported in the patient's medical records and documented in the protocol deviation Log.

No antidote is available, as this is not a possibility with autologous cells.

11.3.2 *Physical examination*

A complete physical examination will include assessments of the head, eyes, throat, skin, neurological, lungs, cardiovascular, abdomen, palpable lymph nodes and extremities. Any abnormalities will be specified and documented as clinically significant or not clinically significant. Abnormal post-dose findings assessed by the Investigator as clinically significant will be reported as AEs.

The patient's general condition will be assessed (overall assessment) and documented in the eCRF. Symptoms like pain, discomfort, nausea, shivering, confusion etc will be reported as AEs.

An assessment of CRS is performed the first 3 months of follow up after pTTL administration: during in-patient care from Day 1 (Visit 5, daily assessments) and during out-patient follow up (Visit 6 to Visit 16). CRS grading is documented in the AE Log of the eCRF.

An ICANS evaluation will be performed as part of each physical examination just before and during the first 3 months of follow up after pTTL administration. A baseline assessment is performed on Day 1 before pTTL administration (Visit 5), and follow-up evaluations are performed from Day 1 (after the administration), during the remaining in-patient care included in Visit 5 (daily assessments) and during out-patient follow up (Visit 6 to Visit 16).

The evaluation takes into account neurological examination findings (level of consciousness, seizures if applicable, motor function findings, and cerebral oedema and/or increased intracranial pressure, if applicable) in combination with the impact, confidence and ease (ICE) score, see below. The ICANS grade is documented in the eCRF. The level of consciousness will be assessed according to the Glasgow Coma Scale (GCS, score 3 to 15) and documented in the eCRF.

The ICE score (0-10 points) is documented in the eCRF as follows:

- Orientation to year, month, city, hospital or place, 4 points
- Correct naming of three objects, 3 points
- Follow simple commands, 1 point
- Writing: ability to write a standard sentence, assessment of alteration in handwriting, 1 point
- Counting backwards from 100 by ten at a time (90, 80, 70…), 1 point

11.3.3 *Vital signs*

Monitoring of vital signs will be performed before, during and after the infusion of the pTTL product, see Section 10.8 and at visits outlined in Table 4 (Part I) and in Table 5 to Table 7 (Part II).

The following will be assessed and recorded in the eCRF:

- Systolic and diastolic blood pressure
- Pulse
- Blood oxygen saturation (pulse oximetry)
- Respiratory frequency (up until and including until week 4, Visit 11)
- Temperature (up until and including week 4, Visit 11)

11.3.4 *Laboratory safety assessments*

Blood samples for analysis of clinical chemistry, haematology and coagulation parameters will be collected through venepuncture, an indwelling venous catheter or arterial needle (if already present) and analysed at a local laboratory by routine analytical methods. Instructions regarding sample collection, preparation and shipping will be provided in a separate Laboratory Manual.

The safety laboratory parameters are defined in Table 17. In addition to the safety lab packages presented in Table 17, standard safety laboratory sampling will also be performed in association with surgery (Visit 2, Part I, approximately 13.2 mL blood)

Urine analysis and pregnancy tests will be performed in accordance with local hospital routines. Serology tests are described in Section 11.1.5. The assessments will be performed at visits specified in Table 4 (Part I) and in Table 5 to Table 7 (Part II).

Safety laboratory values will be specified and documented as normal, abnormal not clinically significant, or abnormal clinically significant in the eCRF. Abnormal post-treatment values assessed by the Investigator as clinically significant will be reported as AEs. If an abnormal value is associated with corresponding clinical signs or symptoms, the sign/symptom must be reported as the AE.

Table 17 Safety laboratory parameters

**Category**	**Parameter**	**Part I**	**Part II**	
		**Screening[1] and Visit 2**	**Large panel**	**Small panel**
**Clinical chemistry**	Alanine aminotransferase (ALT)	X	X	X
	Alkaline phosphatase (ALP)	X	X	
	Aspartate aminotransferase (AST)	X	X	X
	Bilirubin (total and conjugated)	X	X	X
	C-reactive protein (CRP)	X	X	X
	Albumin	X	X	X
	Creatinine	X	X	X
	Electrolytes (Na, K, Ca, Mg)	X	X	X
	Pancreas amylase		X	
	Lactate dehydrogenase (LDH)	X	X	X
	Urea		X	
	Sedimentation rate (SR)		X	
	Thyroid stimulating hormone (TSH)	X	X	
	Ferritin	X	X	X
	IL-6		X	
	Glucose	X	X	
	CMV-PCR (if applicable)		X	
	EBV PCR (if applicable)		X	

Category	Parameter	Part I	Part II	
		Screening[1] and Visit 2	Large panel	Small panel
**Haematology**	Haemoglobin, erythrocytes, platelets, leukocytes, leukocyte subpopulations (neutrophils, lymphocytes, monocytes, basophils and eosinophils), mean corpuscular haemoglobin (MCH), mean corpuscular volume (MCV), erythrocyte volume fraction EVF	X	X	X
**Coagulation**	Activated Partial Thromboplastin Time (APTT)	X	X	
	Prothrombin Complex International Normalised Ratio (PK[INR])	X	X	
**Urine analysis**	Erythrocytes	X	X	
	Leucocytes	X	X	
	Protein	X	X	
	Nitrite	X	X	

1. In case analysis results <1 week old are available, new safety samples are not required at Screening at the discretion of the Investigator.

11.3.5 *Electrocardiograms*

Single 12-lead ECG will be recorded in supine position after 10 minutes of rest using an ECG machine. HR and PR, QRS, QT and QTcB intervals will be recorded. Safety ECGs will be reviewed and interpreted on-site by the Investigator. Any abnormalities will be specified and documented as clinically significant or non-clinically significant. Abnormal post-dose findings assessed by the Investigator as clinically significant will be reported as AEs.

11.4 Appropriateness of measurements

The stopping rules for dose escalation used (see Section 8.4.2) follows the recommendations and grading system of CTCAE v5.0 but also take into account the recommendations published by Sibille et al. 2010, which is an adaptation to FIH studies of the grading systems previously proposed by NCI, WHO, NIH and FDA.

The surgery or biopsy for collection of tumour samples and the trial specific surgery for collection of RLNs, are necessary for the production of pTTL.

Other methods are commonly used in standard medical care and in Phase I cancer studies.

12 PROCEDURES FOR BIOLOGICAL SAMPLES

12.1 Sample collection

Safety laboratory samples are collected according to standard procedures as outlined in the Laboratory Manual. Tumour biopsies and RLN collection will be performed as described in Section 11.2.1 and Section 11.2.3, respectively.

12.2 Volume of blood

The anticipated volume of blood collected from each patient during Part I and Part II is approximately 865 mL over approximately 9 months. Estimated blood volumes to be collected are presented in Table 18 (Part I) Table 19 (Part II).

The anticipated volume of blood collected from each patient during Part III is approximately 450 mL over 4.5 years, see Table 20.

Table 18 Estimated blood volumes Part I

**Category**	**Estimated number of sampling occasions**	**Estimated volume per occasion**	**Estimated total volume**
Clinical chemistry, haematology, coagulation	2	16.2 mL	32.4 mL
Serology	2	3.5 mL	7 mL
Biomarkers	1	24 mL	24 mL
Standard safety lab sampling in association with surgery	1	13.2 mL	13.2 mL
**Total:**			**77 mL**

Table 19 Estimated blood volumes Part II

**Category**	**Estimated number of sampling occasions**	**Estimated volume per occasion**	**Estimated total volume**
Clinical chemistry, haematology, coagulation (including CMV-PCR and EBV-PCR) large panel	8	27.7 mL	221.6 mL
Clinical chemistry, haematology, coagulation small panel	19	10.5	199.5 mL
Serology	1	3.5 mL	3.5 mL
Biomarkers	9	20.5 – 51.5 mL	362.5 mL
**Total:**			**787 mL**

Table 20 Estimated blood volumes Part III

**Category**	**Estimated number of sampling occasions**	**Estimated volume per occasion**	**Estimated total volume**
Biomarkers	7	64 mL	448 mL
**Total:**			**448 mL**

12.3 Handling, storage and destruction of laboratory samples

Blood, urine and tissues samples will be collected and stored according to specifications in the trial specific Laboratory Manual. During the trial, samples will be registered in the eBiobank of Region Stockholm (IVO 722) and will once the trial is completed be transferred to the Biobank of Region Stockholm (IVO 914) in accordance with appropriate laws and

regulations. All samples will be coded and can only be identified via the code key in the possession of the investigator.

The patient retains the right to request the sample material be destroyed by contacting the investigator. Following the request from the patient, the investigator is to provide the sponsor with the patient ID number so that any remaining blood and tissues samples can be located and destroyed.

Any remains from the safety laboratory samples will be disposed of after analyses.

The samples for analyses of exploratory parameters will be stored at <-70°C until analysed. The samples will be stored in the Biobank of Region Stockholm (or other vendor as detailed in a material transfer agreement) for up to 10 years after end of Part III or after patient death, whichever comes first.

12.4 Chain of custody of biological samples

A full chain of custody is maintained for all samples throughout their lifecycle.

The trial sites keep full traceability of collected biological samples from the patients while in storage at the research clinic until shipment and keeps documentation of receipt of arrival.

The sample receiver keeps full traceability of the samples while in their storage and during use until used or disposed of.

The Sponsor keeps oversight of the entire life cycle through internal procedures, monitoring of trial sites and auditing of external laboratory providers.

12.5 Withdrawal of informed consent for donated biological samples

If a patient withdraws consent to the use of biological samples donated, the samples will be disposed of/destroyed, if not already analysed and documented.

The Principal Investigator at each site will ensure that:

- patient withdrawal of informed consent is notified immediately to Sponsor.
- biological samples from the patient, if stored at the research clinic, are immediately identified, disposed of/destroyed and the action is documented.

The Sponsor has to ensure that the laboratory(ies) holding the samples is/are informed about the withdrawn consent immediately and that samples are disposed of/destroyed and that the action is documented.

13 QUALITY MANAGEMENT, QUALITY ASSURANCE AND QUALITY CONTROL

13.1 Quality management: critical process, system and data identification

During CTP development, the Sponsor will identify those processes, systems (facilities, computerised systems) and data that are critical to ensure human subject protection and the reliability of trial results according to applicable SOPs and International Council for Harmonisation of Technical Requirements for Pharmaceuticals for Human Use (ICH) E6 R2.

Identified risks, including risks associated with any unforeseen event, e.g., a pandemic, will be categorised separately from the CTP.

Sponsor oversight responsibilities, such as monitoring, AE reporting, safety monitoring, changes in investigators and key trial team staff and quality assurance (QA) activities may need to be reassessed in relation unforeseen events, as a pandemic, and temporary, alternative proportionate mechanisms of oversight may be required.

13.2 Quality assurance and quality control

The Sponsor is responsible for implementing and maintaining QA and quality control (QC) systems with written SOPs with regards to management of identified risks, CTP compliance, GCP compliance and applicable regulatory requirements.

The Sponsor is responsible for securing agreements with involved subcontractors and to perform regular subcontractor oversight to ensure CTP compliance, GCP compliance and compliance with applicable regulatory requirements.

The Sponsor is responsible for implementing a risk-based validated electronic data capture system and maintain SOPs for the whole life cycle of the system.

QC should be applied to each stage of data handling to ensure that all data are reliable and have been processed correctly.

The Sponsor has delegated the responsibilities outlined above to CTC whilst maintaining overall trial oversight.

14 ETHICAL AND REGULATORY REQUIREMENTS

14.1 Ethical conduct of the trial

The trial will be performed in accordance with the CTP, the ethical principles that have their origin in the Declaration of Helsinki and are consistent with ICH/GCP E6 (R2), Clinical Trials Regulation (EU) 536/2014, and applicable local regulatory requirements.

14.2 Ethics and regulatory review

The Coordinating Investigator is responsible for submission of the CTP, the patient information and ICF, any other written information to be provided to the patients and any advertisements used for recruitment of patients to applicable IEC for approval.

The Sponsor has delegated to CTC the responsibility to submit trial documents to the applicable CA according to local regulatory requirements.

Approval must be obtained in writing from both IEC and CA before the first patient can be recruited.

The Sponsor will provide the CA, IEC and Principal Investigators with safety updates/reports according to local requirements. Progress reports and notifications of SUSARs will be provided to the IEC according to local regulations and guidelines.

14.3 Patient information and consent

It is the responsibility of the Investigator or an authorised associate to give each potential trial patient adequate verbal and written information before any trial specific assessments are performed. The information will include the objectives and the procedures of the trial as well as any risks or inconvenience involved. It will be emphasised that participation in the trial is

voluntary and that the patient may withdraw from participation at any time and for any reason, without any prejudice. All patients will be given the opportunity to ask questions about the trial and will be given sufficient time to consider participation before signing the ICF.

Prospective trial patients will be asked to give 3 separate consents, one for pre-screening in Part I (Section 9.2.1) and one for each part of the trial: tissue collection and pTTL production in Part I (Section 9.2.2), and pTTL treatment and follow-up in Part II and Part III (Section 9.2.3).

Before performing any trial-related procedures the ICF(s) must be signed and personally dated by the patient and by the Investigator. A copy of the patient information including the signed ICF will be provided to the patient. Documentation of the discussion and the date of informed consent must be recorded in the source documentation and in the eCRF. The patient information sheet and each signed ICF should be filed by the Investigator for possible future audits and/or inspections.

Substantial changes to the final approved versions of the patient information and ICFs must not be performed without approval from the Sponsor and the applicable IEC.

The ICF for participation in Part I must be signed by the patient and Investigator prior to performing any trial specific procedures. The patient receives oral and written information about the trial in its entirety, but with focus on Part I, by the responsible Investigator at any of the Recruitment Sites. It will be clearly explained also that inclusion in Part II of the trial is dependent on successful production of pTTL and that entry criteria will be re-evaluated at inclusion in Part II.

The first 2 consents (pre-screening and screening, Part I) may, in some cases, take place while the patient has other non-curative therapeutic options but might become eligible for inclusion in Part II of the trial within approximately 24 months.

The ICF for Part II/Part III must be signed by the patient and Investigator prior to performing any Part II procedures.

14.4 Patient alert card

The patient will be provided with a Patient information card including the following information:

- That he/she is participating in a clinical trial
- Patient trial ID
- That he/she is treated with pTTL including information about pTTL
- The name and phone number of the Coordinating Investigator
- Name and address of the Sponsor

14.5 Patient data protection

The ICF includes information that data will be recorded, collected and processed and may be transferred to European Economic Area (EEA) or non-EEA countries. In accordance with the European Union general data protection regulation (GDPR), Regulation (EU) 2016/679, the data will not identify any persons taking part in the trial.

The potential trial patient must be informed that by signing the ICF he/she approves that authorised representatives from the Sponsor and CTC and the concerned IEC have direct

access to their medical records for verification of clinical trial procedures. For further details on the patient information and ICF process, refer to Section 14.3.

The patient has the right to request access to his/her personal data and the right to request rectification of any data that is not correct and/or complete in accordance with Regulation (EU) 2016/679 and the request will be raised to the Principal Investigator.

The Investigator must file a Patient Identification List which includes sufficient information to link records, i.e. the eCRF and clinical records. This list should be preserved for possible future inspections/audits but must not be made available to the Sponsor except for monitoring or auditing purposes.

Personal data that are collected in the trial such as health information and ethnicity are considered as sensitive personal data. This data will be pseudonymised, i.e. personally identifiable information (PII) will be removed and replaced by a unique patient ID and will be processed by the Sponsor and other involved parties during the trial. After the trial end, only anonymised data, i.e. aggregated data sets, can be used.

For this trial, the Sponsor NEOGAP Therapeutics AB is the data controller of all data processed during the trial (e.g. TMF, trial reports) and CTC AB is the data processor. Any subcontractors used in the trial, are also data processors.

For data that are processed at the clinic(s) (e.g. medical records and ISF), each site is the data controller. The ISF is kept in a locked cabinet and only authorised staff will have access to it.

14.6 Changes to the approved clinical trial protocol

Any proposed change to the approved final CTP (including appendices) will be documented in a written and numbered clinical protocol amendment. All substantial amendments to the protocol must be approved by the appropriate IEC and/or CA before implementation according to applicable regulations.

14.7 Audits and inspections

Authorised representatives of Sponsor, a CA, or an IEC may perform audits or inspections at the research clinic, including source data verification (SDV). The purpose of an audit or inspection is to systematically and independently examine all trial-related activities and documents, to determine whether these activities were conducted, and data were recorded, analysed, and accurately reported according to the protocol, ICH-GCP guidelines and any applicable regulatory requirements. The Investigator will contact the Sponsor immediately if contacted by a CA about an inspection at trial site.

14.8 Insurance

Patients will be covered under Sponsor’s liability insurance policy through the Swedish Pharmaceutical Insurance (Läkemedelsförsäkringen). The certificate of insurance and an information leaflet containing essential information about the insurance coverage can be provided upon request. The participating patients are also protected in accordance with national regulations, as applicable.

15 TRIAL MANAGEMENT

15.1 Training of trial site personnel

Before enrolment of the first trial patient, CTC will perform a site initiation visit (SIV) at each site. The requirements of the CTP and related documents will be reviewed and discussed and the investigational staff will be trained in any trial specific procedures and system(s) utilised.

It is the responsibility of the Principal Investigator at each site to ensure that all personnel involved in the trial are fully informed of all relevant aspects of the trial and have a detailed knowledge of and training in the procedures that are to be executed by them. Any new information of relevance to the performance of this trial must be forwarded to the staff involved in a timely manner.

The Investigator will keep a list of all personnel involved in the trial together with their function and trial related duties delegated. A Curriculum Vitae will be available for all staff to whom trial specific duties are delegated.

15.2 Clinical monitoring

The Sponsor is responsible for securing agreement from all involved parties to ensure direct access to all trial related sites, source data/documents, and reports for the purpose of monitoring and auditing by the Sponsor, and inspection by domestic and foreign regulatory authorities.

As defined in the risk-based monitoring (RBM) plan, approved by the Sponsor and provided separately, the responsible Monitor will periodically visit the trial site at times agreed upon by the Investigator and the Monitor. Adaptations related to the on-site monitoring plan, when it is impossible or inappropriate to follow due to e.g., a pandemic, may be required such as supplementation with (additional/increased) centralised monitoring and central review of data if considered possible and meaningful. Results of adjusted monitoring/review measures should be reported to the Sponsor in monitoring reports and in the clinical trial report as applicable. At the time of each monitoring visit, the role of the Monitor is (but not limited to) to:

- provide information and support to the investigational team.
- confirm that facilities and resources remain acceptable.
- confirm that the investigational team is adhering to the CTP, applicable SOPs, guidelines, manuals and regulatory requirements.
- verify that data are being accurately and timely recorded in the eCRFs and that IMP accountability checks are being performed.
- verify that data in the eCRF are consistent with the clinical records (SDV) in accordance with the RBM plan.
- verify that the correct informed consent procedure has been adhered to for participating patients.
- ensure that withdrawal of informed consent to the use of the patient’s biological samples will be reported and biological samples are identified and disposed of/destructed accordingly, and that this action is documented and reported to the patient.
- verify that AEs are recorded and reported in a timely manner and according to the CTP.
- raise and escalate any serious quality issues, serious GCP breach and any data privacy breach to the Sponsor.

Centralised monitoring will also be performed continuously by trial team members at CTC in accordance with the RBM plan.

When the trial has been completed and all queries have been resolved and the database has been locked, the Monitor will perform a close-out visit.

15.3 Source data documents

A separate Origin of Source Data List will be generated for each site before start of enrolment, specifying the location of the source of derived information appearing in the eCRF. This document must be signed by the Principal Investigator at each site and the Monitor to confirm agreement before start of recruitment.

Source documents are all documents used by the Investigator or hospital that relate to the patient's medical history, that verifies the existence of the patient, the inclusion and exclusion criteria, and all records covering the patient's participation in the trial. They include laboratory notes, memoranda, material dispensing records, patient files, etc. The eCRF may constitute source data if clearly defined in the Origin of Source Data List.

The Investigator should guarantee access to source documents to the Monitor, CAs and the IECs, if required.

15.4 Trial agreements

The Principal Investigator at each site must comply with all the terms, conditions, and obligations of the Clinical Trial Agreement for this trial.

Agreements between the Sponsor and CTC must be in place before any trial-related procedures can take place, or patients be enrolled.

15.5 Trial time table and end-of-trial

Part I of the trial is expected to start in Q1 2023 and Part II is estimated to be completed by Q2/Q3 2026. Part III will commence for 4.5 years or until death of all patients until approximately Q4 2030.

A patient is considered to have completed Part II of the trial if he/she has completed all visits in Part II. A patient is considered to have completed the trial if he/she has completed the long-term follow-up or if he/she has participated in the trial until death.

The end of the trial will be defined as the date of the last patient's last visit in the trial.

15.6 Termination of the trial

The Investigator(s) or the Sponsor may terminate this trial prematurely for any reasonable cause. The IEC and CA should be informed promptly. Conditions that may warrant trial termination include, but are not limited to:

- The discovery of an unexpected, significant, or unacceptable risk to the patients enrolled in the trial or potential trial patients; or
- A decision by the Sponsor to suspend or discontinue development of pTTL.

If the CA obtains information that raises doubts about the safety or scientific validity of the clinical trial, the CA can suspend or prohibit the trial. Before the CA reaches its decision, it shall, except where there is imminent risk, ask the Sponsor and/or the Investigator for their opinion, to be delivered within one week (Directive 2001/20/EC, Article 12, Section 1).

If the trial is prematurely terminated or suspended for any reason, the Investigator should promptly inform the trial patients and should assure appropriate follow-up for the patients.

15.7 Reporting and publication

15.7.1 *Clinical trial report*

The trial results will be reported in the EudraCT database per applicable regulations within 12 months after completion of the trial. This fulfils the summarising report requirements specified in LVFS 2011:1 9, Chapter 9.

After completion of Part I and Part II, an ICH E3 compliant clinical trial report will be prepared by the Sponsor or delegate. The report will be reviewed and approved by, as a minimum, the Coordinating Investigator, the Statistician and the Sponsor. Long-term follow-up data will be reported in an addendum to the clinical trial report. Data from the long-term follow-up will also be reported in yearly development safety update reports (DSURs), see below.

Trial results will also be reported at ClinicalTrials.gov.

Exploratory endpoints may be reported separately from the clinical trial report.

15.7.2 *Annual safety report*

If the trial duration exceeds one year, the Sponsor must submit a DSUR to the CA and to the IEC. The report shall summarise all pertinent safety information collected during the reporting period and contain an update of the risk-benefit evaluation if there has been any change since the approval of the clinical trial.

15.7.3 *Confidentiality and ownership of trial data*

Any confidential information relating to pTTL or the trial, including any data and results from the trial, will be the exclusive property of the Sponsor. The Investigator and any other persons involved in the trial are responsible for protecting the confidentiality of this proprietary information belonging to the Sponsor.

15.7.4 *Publication*

The trial results will be communicated at scientific meetings and in peer-reviewed international journals.

When evaluating co-authorship, the Vancouver convention on authorship (http://www.icmje.org/icmje-recommendations.pdf) will be followed. A publication in a peer-reviewed journal will not be made until the main final clinical trial report describing Part I and Part II data has been signed off by the Sponsor. The clinical trial report will be the basis for the publication in a recognised international scientific journal. Participating Investigators at the Recruitment & Surgery Sites and the Recruitment, Surgery & Treatment Site will be offered co-authorship. The trial participation is considered to be equally meritorious for the Coordinating/Principal Investigator, [redacted], MD, PhD and the Investigator, [redacted], MD, PhD, Associate Professor, at the Recruitment, Surgery & Treatment Site, which will be reflected in the publication. Referral sites will either be offered co-authorship or will be mentioned in acknowledgements, in accordance with the Vancouver guidelines on authorship mentioned above.

Prior to submitting any results of this trial for publication or presentation, the author(s) must allow the Sponsor 30 days in which to review and comment upon the publication manuscript.

In accordance with generally recognised principles of scientific collaboration, co-authorship will be discussed and mutually agreed upon between the Sponsor and the collaborating investigator before submission of a manuscript to a publisher.

15.8 Archiving

The Principal Investigator at each site is responsible for maintaining essential documents, (as defined in ICH E6 GCP, Section 8) for 30 years after expiry date of the product. This includes any original source documents related to the trial, the Patient Identification List (providing the sole link between named patient source records and anonymous eCRF data), the original signed ICFs and detailed records of disposition of IMP.

It is the responsibility of the Sponsor to inform the Investigator/institution as to when these documents no longer need to be retained.

The Sponsor will archive the TMF in accordance with ICH E6 GCP, Section 8 and applicable regulatory requirements.

The data from the eCRFs will be sent to the Sponsor and a copy will be sent to the clinic and filed in the Investigator Trial File for archiving for 30 years after the expiry date of the product. The completed eCRFs are the sole property of the Sponsor and should not be made available in any form to third parties, except for authorised representatives of appropriate Health/Regulatory Authorities, without written permission from the Sponsor.

16 DATA MANAGEMENT

The data management routines include procedures for handling of the eCRF, database set-up and management, data entry and verification, data validation, QC of the database, and documentation of the performed activities including information of discrepancies in the process. The database, data entry screens, and program will be designed in accordance with the CTP.

Data validation/data cleaning procedures are designed to assure validity and accuracy of clinical data. These procedures consist of computerised online edit checks identifying e.g. data values that are outside the allowed range and SAS-programmed batch checks on data exports. All trial -specific and standard data validation programming will be tested in a separate testing environment prior to use on production data.

Detailed information on data management will be described in a trial -specific Data Management Plan (DMP).

16.1 The web-based eCRF

Clinical data will be entered into a 21 CFR Part 11-compliant eCRF (Viedoc™) provided by Viedoc Technologies, Uppsala, Sweden. The eCRF includes password protection and internal quality checks, such as automatic range checks, to identify data that appear inconsistent, incomplete, or inaccurate. Clinical data will be entered directly from the source documents or at bedside (if the eCRF data constitutes source data). Source data are to be defined at the sites before inclusion of the first patient (Section 15.3).

Authorised site personnel designated by the Investigator will complete data collection. Appropriate training and security measures will be completed with the Investigator and all

authorised trial site personnel prior to the trial being initiated and any data being entered into the system for any trial patient.

16.2 The entering of data into the eCRF

All entries, corrections, and alterations are to be made by the Investigator or designee. Neither the Monitor nor any other trial team member besides site staff can enter data in the eCRF. All data should be entered in English. The eCRFs should be completed as soon as possible during or after the patient's visit (maximum 3 days). The Investigator must verify that all data entries in the eCRFs are accurate and correct. If some assessments are not done, or if certain information is not available, not applicable or unknown, the Investigator or assigned clinical staff should record such information in the eCRF. The Investigator will be required to electronically sign off the clinical data. This will be performed by means of the Investigator's unique User ID and password; date and time stamps will be added automatically at time of electronic signature.

16.3 The query process

The Monitor will review the eCRFs and evaluate them for completeness and consistency. Data in the eCRF will be compared with the respective source documents to ensure that there are no discrepancies for critical data as described in the RBM plan. All entries, corrections, and alterations are to be made by the Investigator or designee.

If corrections are needed, queries will be raised within the eCRF, either as a result of built-in edit checks or manually raised by the monitor. An appropriate member of the site staff will answer the queries in the eCRF either by correcting the data or by entering a response to the query. The monitor will either approve the answer/correction or re-issue the query.

16.4 Audit trail

All entries in the eCRF will be fully recorded in a protected audit trail. Once clinical data have been saved, corrections to the data fields will be audit trailed, meaning that the reason for change, the name of the person who made the change, together with time and date will be logged.

16.5 External data

External data consists of electronic data that are not recorded in the eCRF. Key variables are defined in order to uniquely identify each sample record. File and data formats are agreed with the external data provider.

16.6 Medical coding

Medical coding will be performed by trained personnel at CTC. AEs and medical/surgical history verbatim terms will be coded using the Medical Dictionary of Regulatory Activities (MedDRA; latest version available at the time of eCRF design). Prior and concomitant medications will be coded according to the WHO Anatomic Therapeutic Chemical (ATC) classification system. All coding will be approved by Sponsor prior to database lock.

16.7 Database lock

When all data have been entered and discrepancies solved, clean file will be declared, the database for Part I and Part II will be locked and the data will be analysed. Part III data will be

collected in a separate database.

17 STATISTICAL METHODS AND DETERMINATION OF SAMPLE SIZE

The principal features of the statistical analysis to be performed are described in this section. A more technical and detailed elaboration of the principal features will be presented in a separate Statistical Analysis Plan (SAP), which will be signed and approved prior to database lock. Analyses will be performed by CTC.

17.1 General

Continuous data will be presented in terms of evaluable and missing observations, arithmetic mean, standard deviation, median, minimum and maximum value. Categorical data will be presented as counts and percentages. When applicable, summary data will be presented by treatment, and by assessment time. Individual patient data will be listed by patient number, treatment, and, where applicable, by assessment time.

All descriptive summaries and statistical analyses will be performed using SAS Version 9.4 or later (SAS Institute, Inc., Cary, NC).

Baseline will be defined as the visit with last data collection point prior to the first administration of pTTL.

No imputation of missing data will be performed.

17.2 Determination of sample size

No formal sample size calculation has been performed for this trial. The proposed sample size is considered sufficient for evaluation of the trial endpoints.

17.3 Analysis data sets

17.3.1 *Safety analysis set*

The Safety Analysis Set will comprise all patients who received pTTL. Additional analysis sets (e.g. an analysis set excluding patients who received out of specification treatment) may be defined in the SAP.

17.4 Description of trial population

17.4.1 *Demographics and baseline characteristics*

Descriptive statistics for demographics, weight and height will be presented.

17.4.2 *Medical/surgical history and prior/concomitant medication*

Medical/surgical history will be presented by system organ class (SOC) and preferred term (PT). Prior/concomitant medications will be presented by Anatomical Therapeutic Chemical (ATC) level 3 and 5.

All data will be listed by patient.

17.4.3 *Treatment compliance*

The patients treated and their individual dose will be listed.

17.5 Analysis of primary endpoints

17.5.1 *Adverse events*

An overview of all AEs, including SAEs, intensity, relationship to pTTL, and deaths will be presented. Incidence of AEs and SAEs will be summarised by SOC and PT. AESI and related AEs will be summarised separately if considered relevant.

All AE data will be listed patient and include the verbatim term entered by the Investigator.

17.5.2 *Physical examination*

Clinically significant and non-clinically significant abnormal findings will be specified and summarised. Changes over time will be presented using shift tables, if considered appropriate.

All data will be listed by patient.

17.5.3 *Vital signs*

Vital signs (systolic/diastolic blood pressure, pulse, saturation, respiratory rate and body temperature) will be summarised. Data will be presented with absolute change from baseline.

All data will be listed by patient.

17.5.4 *12-lead ECG*

All ECGs will be categorised as ”normal”, ”abnormal, not clinically significant”, or ”abnormal, clinically significant” (as judged by the Investigator) and summarised using frequency tables. Changes over time will be presented using shift tables, if considered appropriate.

All data will be listed by patient.

17.5.5 *Safety laboratory analyses*

Safety laboratory data will be summarised with absolute change from baseline at each visit. Abnormal, clinically significant values will be summarised separately, if considered appropriate.

All data will be listed by patient.

17.6 Analysis of secondary endpoints

17.6.1 *Definitions*

Response rate is defined as the proportion of patients who have achieved CR or PR, i.e. at least PR, in the target lesions measured using the iRECIST criteria.

Time to progression (TTP) is defined as the time from baseline until objective tumour progression. Time to progression will be calculated from the date of last post-therapy follow-up visit for patients that have not progressed. TTP does not include deaths.

Overall survival is defined as time from baseline (inclusion in Part II) until death from any cause. Survival time will be censored on the date of last post-therapy follow-up visit for patients who survived.

Progression-free survival (PFS) is defined as the time from baseline until objective tumour progression or death.

Disease specific survival (DSS) is defined as time from baseline until death from colon cancer. Survival time will be censored on the date of last post-therapy follow-up visit for patients who survived. Patients who died from other causes will be censored at time of death.

17.6.2 *Time-to-event variables*

All "time-to-event" variables will descriptively summarised and displayed in e.g. Kaplan-Meier plots.

17.6.3 *Tumour response*

CR and PR at 6 months in the target lesion will be based on the iRECIST criteria.

17.6.4 *Correlation between tumour response and tumour markers*

Correlation between tumour response and tumour markers will be calculated.

17.6.5 *Exploratory analyses*

Exploratory endpoints may be reported separately from the clinical trial report. Details on their analysis will be described in the SAP or elsewhere.

18 REFERENCES

Achilles Therapeutics Press Release April 4 2024. https://ir.achillestx.com/news-releases/news-release-details/achilles-therapeutics-provides-interim-phase-iiia-update-clonal

Achilles Therapeutics presentation at TIL Summit, Boston, October 2024. https://ir.achillestx.com/static-files/4e49f16d-2fee-4a9c-94d3-8b1a7c873da9

Adkins, RN, MSN, ANP-C, Sherry. 2019. "CAR T-Cell Therapy: Adverse Events and Management." *Journal of the Advanced Practitioner in Oncology* 10(4).

AMTAGVI FDA Summary Basis for Regulatory Action 2024. https://www.fda.gov/media/176893/download?attachment (March 18, 2024).

AMTAGVI Prescribing Information 2024. https://www.fda.gov/media/176417/download?attachment (March 18, 2024)

Andersen, Rikke et al. 2016. "Long-Lasting Complete Responses in Patients with Metastatic Melanoma after Adoptive Cell Therapy with Tumor-Infiltrating Lymphocytes and an Attenuated IL2 Regimen." *Clinical cancer research : an official journal of the American Association for Cancer Research* 22(15): 3734–45.

Anwar, S., Peter, M.B., Dent, J. and Scott, N.A 2012. "Palliative excisional surgery for primary colorectal cancer in patients with incurable metastatic disease. Is there a survival benefit? A systematic review." *Colorectal Disease* 14: 920-930.

Bainbridge et al. 2012. "Perioperative and anaesthetic-related mortality in developed and developing countries: a systematic review and meta-analysis." *The Lancet* 380(9847):1075-81.

Bechman, Natasha, and John Maher. 2021. "Lymphodepletion Strategies to Potentiate Adoptive T-Cell Immunotherapy – What Are We Doing; Where Are We Going?" *Expert Opinion on Biological Therapy* 21(5): 627–37.

Berdeja, Jesus G et al. 2021. "Ciltacabtagene Autoleucel, a B-Cell Maturation Antigen-Directed Chimeric Antigen Receptor T-Cell Therapy in Patients with Relapsed or Refractory Multiple Myeloma (CARTITUDE-1): A Phase 1b/2 Open-Label Study." *The Lancet* 398(10297): 314–24.

Besser, Michal J et al. 2020. "Comprehensive Single Institute Experience with Melanoma TIL: Long Term Clinical Results, Toxicity Profile, and Prognostic Factors of Response." *Molecular carcinogenesis* 59(7): 736–44.

Betof Warner, Allison; Hamid, Omid; Komanduri, Krishna; Amaria, Rodabe; Butler, Marcus O;Haanen, John; Nikiforow, Sarah; Puzanov, Igor; Sarnaik, Amod; Bishop, Michael R.; Schoenfeld, Adam J. 2024. "Expert consensus guidelines on management and best practices for tumor-infiltrating lymphocyte cell therapy". 2024. *J Immunother Cancer. 12:e008735*

Borgers, Jessica S. W., Divya Lenkala, Victoria Kohler, Emily K. Jackson, Matthijs D. Linssen, Sebastian Hymson, Brian McCarthy, et al. 2025. "Personalized, Autologous Neoantigen-Specific T Cell Therapy in Metastatic Melanoma: A Phase 1 Trial." *Nature Medicine*.

Bray, Freddie et al. 2018. "Global Cancer Statistics 2018: GLOBOCAN Estimates of Incidence and Mortality Worldwide for 36 Cancers in 185 Countries." *CA: a cancer journal for clinicians* 68(6): 394–424.

Cappell, Kathryn M. et al. 2020. "Long-Term Follow-Up of Anti-CD19 Chimeric Antigen Receptor T-Cell Therapy." *Journal of Clinical Oncology* 38(32): 3805–15.

Chapuis, Aude G. et al. 2017. "Tracking the Fate and Origin of Clinically Relevant Adoptively Transferred CD8+ T Cells in Vivo." *Science Immunology* 2(8).

Chesney J, Lewis KD, Kluger H, Hamid O, Whitman E, Thomas S, Wermke M, Cusnir M, Domingo-Musibay E, Phan GQ, Kirkwood JM, Hassel JC, Orloff M, Larkin J, Weber J, Furness AJS,

Khushalani NI, Medina T, Egger ME, Graf Finckenstein F, Jagasia M, Hari P, Sulur G, Shi W, Wu X, Sarnaik A. 2022. "Efficacy and safety of lifileucel, a one-time autologous tumor-infiltrating lymphocyte (TIL) cell therapy, in patients with advanced melanoma after progression on immune checkpoint inhibitors and targeted therapies: pooled analysis of consecutive cohorts of the C-144-01 study." *J Immunother Cancer* 10(12):e005755.

Cox, M.A., Kastrup, J. & Hrubiško, M. 2012. "Historical perspectives and the future of adverse reactions associated with haemopoietic stem cells cryopreserved with dimethyl sulfoxide." *Cell Tissue Bank* 13, 203–215.

Corbett, T H et al. 1975. "Tumor induction relationships in development of transplantable cancers of the colon in mice for chemotherapy assays, with a note on carcinogen structure". *Cancer Research* 35(9): 2434-9.

Dafni, U et al. 2019. "Efficacy of Adoptive Therapy with Tumor-Infiltrating Lymphocytes and Recombinant Interleukin-2 in Advanced Cutaneous Melanoma: A Systematic Review and Meta-Analysis." *Annals of Oncology* 30(12): 1902–13.

Dudley, Mark E et al. 2002. "A Phase I Study of Nonmyeloablative Chemotherapy and Adoptive Transfer of Autologous Tumor Antigen-Specific T Lymphocytes in Patients with Metastatic Melanoma." *Journal of Immunotherapy* 25(3): 243–51.

Dudley, Mark E et al. 2005. "Adoptive Cell Transfer Therapy Following Non-Myeloablative but Lymphodepleting Chemotherapy for the Treatment of Patients with Refractory Metastatic Melanoma." *Journal of clinical oncology : official journal of the American Society of Clinical Oncology* 23(10): 2346–57.

Dudley, Mark E et al. 2008. "Adoptive Cell Therapy for Patients with Metastatic Melanoma: Evaluation of Intensive Myeloablative Chemoradiation Preparative Regimens." *Journal of clinical oncology : official journal of the American Society of Clinical Oncology* 26(32): 5233–39.

Eisenhauer EA et al. 2009. "New response evaluation criteria in solid tumours: revised RECIST guideline (version 1.1)". *Eur J Cancer.* 45:228–47.

Ellebaek, Eva et al. 2012. "Adoptive Cell Therapy with Autologous Tumor Infiltrating Lymphocytes and Low-Dose Interleukin-2 in Metastatic Melanoma Patients." *Journal of Translational Medicine* 10(1): 169.

"European Commission. Clinical Trials – Directive 2001/20/EC. April 4, 2001. Ec.Europa.eu."

"European Commission. Directive 2006/17/Ec. February 8, 2006."

"European Commission. Regulation (EU) 2016/679 of the European Parliament and of the Council of 27 April 2016 on the protection of natural persons with regard to the processing of personal data and on the free movement of such data, and repealing Directive 95/46/EC (General Data Protection Regulation). 2016."

"European Commission. Guidelines on Good Clinical Practice specific to Advanced Therapy Medicinal Products. 2019)"

"European Medicines Agency. Guideline on strategies to identify and mitigate risks for first-in-human and early clinical trials with investigational medicinal products. EMEA/CHMP/SWP/28367/07 Rev. 1 2017"

"FDA Guidance for Industry. Toxicity Grading Scale for Healthy Adult and Adolescent Volunteers Enrolled in Preventive Vaccine Clinical Trials." 2007.

Finn, Olivera J. 2018. "A Believer's Overview of Cancer Immunosurveillance and Immunotherapy." *Journal of immunology (Baltimore, Md. : 1950)* 200(2): 385–91.

Fridman et al. 2017. "The immune contexture in cancer prognosis and treatment." *Nature Reviews Clinical Oncology* (14) 717–734.

Galon, J. et al. 2006. “Type, density, and location of immune cells within human colorectal tumors predict clinical outcome.” *Science* 313, 1960–1964.

Gillison,Maura et al. 2022. “Abstract CT153: TiTAN: a phase 1 study of GEN-011, a neoantigen-targeted peripheral blood-derived T cell therapy with broad neoantigen targeting”. *Cancer Res* (82) (12_Supplement): CT153.

Goff, Stephanie L. et al. 2016. “Randomized, Prospective Evaluation Comparing Intensity of Lymphodepletion before Adoptive Transfer of Tumor-Infiltrating Lymphocytes for Patients with Metastatic Melanoma.” *Journal of Clinical Oncology* 34(20): 2389–97.

Guinney, Justin et al. 2015. “The Consensus Molecular Subtypes of Colorectal Cancer.” *Nature Medicine* 21(11): 1350–56.

Haanen, J.B.A.G. et al. 2022. ”LBA3 - Treatment with tumor-infiltrating lymphocytes (TIL) versus ipilimumab for advanced melanoma: Results from a multicenter, randomized phase III trial”. *Annals of Oncology* 33, Suppl. 7, S1406”.

Hanahan, Douglas, and Robert A. Weinberg. 2011. “Hallmarks of Cancer: The Next Generation.” *Cell* 144(5): 646–74.

Hornberger, K; Yu, G; McKenna, D; Hubel, A. 2019. “Cryopreservation of Hematopoietic Stem Cells: Emerging Assays, Cryoprotectant Agents, and Technology to Improve Outcomes.” *Transfus Med Hemother* 46 (3): 188–196.

Ishiara, M et al. 2022. “NY-ESO-1-specific redirected T cells with endogenous TR knockdown mediate tumor response and cytokine release syndrome.” *J Immunother Cancer* 10(6):e003811.

Jain, Tania, and Mark R. Litzow. 2020. “Management of Toxicities Associated with Novel Immunotherapy Agents in Acute Lymphoblastic Leukemia.” *Therapeutic Advances in Hematology* 11: 204062071989989.

Julve, M; Wong, Y.N.S; Lim, K.H.J.; Furness, A.J.S. 2024. “Solid tumour cellular therapy — principles of toxicity management”. *Immuno-Oncology and Technology* 100737.

Jung, Bärbel et al. 2021. “Nationellt Vårdprogram Tjock- Och Ändtarmscancer.” https://kunskapsbanken.cancercentrum.se/globalassets/cancerdiagnoser/tjock--och-andtarm-anal/vardprogram/nationellt-vardprogram-tjock-andtarmscancer.pdf (December 10, 2021).

Kanemitsu Y, Shitara K, Mizusawa J 2021.”Primary Tumor Resection Plus Chemotherapy Versus Chemotherapy Alone for Colorectal Cancer Patients With Asymptomatic, Synchronous Unresectable Metastases (JCOG1007; iPACS): A Randomized Clinical Trial” *J Clin Oncol.* 2021 Apr 1;39(10):1098-1107.

Karlsson, Mona et al. 2010. “Pilot Study of Sentinel-Node-Based Adoptive Immunotherapy in Advanced Colorectal Cancer.” *Annals of Surgical Oncology* 17(7): 1747–57.

Kiessling, Adela et al. 2022. “Generation of Tumor-Specific Cytotoxic T Cells from Blood via in Vitro Expansion Using Autologous Dendritic Cells Pulsed with Neoantigen-Coupled-Microbeads.” *Frontiers in Oncology, section Cancer Molecular Targets and Therapeutics* 12:866763.

Klebanoff, C et al. 2005. “Sinks, Suppressors and Antigen Presenters: How Lymphodepletion Enhances T Cell-Mediated Tumor Immunotherapy.” *Trends in Immunology* 26(2): 111–17.

Kochenderfer, James N et al. 2010. “Eradication of B-lineage cells and regression of lymphoma in a patient treated with autologous T cells genetically engineered to recognize CD_{19}”. *Blood* 116(20): 4099-102.

Kochenderfer, James N et al. 2012. “B-Cell Depletion and Remissions of Malignancy along with Cytokine-Associated Toxicity in a Clinical Trial of Anti-CD19 Chimeric-Antigen-Receptor-Transduced T Cells.” *Blood* 119(12): 2709–20.

Kochenderfer, James N. et al. 2013. "Treating B-cell cancer with T cells expressing anti-CD19 chimeric antigen receptors". *Nat Rev Clin Oncol* 10(5): 267-76.

Kochenderfer, James N. et al. 2015. "Chemotherapy-Refractory Diffuse Large B-Cell Lymphoma and Indolent B-Cell Malignancies Can Be Effectively Treated With Autologous T Cells Expressing an Anti-CD19 Chimeric Antigen Receptor." *Journal of Clinical Oncology* 33(6): 540–49.

Lamb, Margaret G., Hemalatha G. Rangarajan, Brian P. Tullius, and Dean A. Lee. 2021. "Natural Killer Cell Therapy for Hematologic Malignancies: Successes, Challenges, and the Future." *Stem Cell Research & Therapy* 12(1): 211.

Lee, Daniel W et al. 2015. "T Cells Expressing CD19 Chimeric Antigen Receptors for Acute Lymphoblastic Leukaemia in Children and Young Adults: A Phase 1 Dose-Escalation Trial." *Lancet (London, England)* 385(9967): 517–28.

Lee C-H, Yelensky R, Jooss K, and Chan TA. 2018. "Update on Tumor Neoantigens and Their Utility: Why It Is Good to Be Different." *Trends in Immunology* 39(7): 536–48.

Locke, Frederick L. et al. 2021. "Axicabtagene Ciloleucel as Second-Line Therapy for Large B-Cell Lymphoma." *New England Journal of Medicine* 386(7):640.

Lu, Yong-Chen et al. 2017. "Treatment of Patients With Metastatic Cancer Using a Major Histocompatibility Complex Class II–Restricted T-Cell Receptor Targeting the Cancer Germline Antigen MAGE-A3." *Journal of Clinical Oncology* 35(29): 3322–29.

Marits, P. et al. 2006. "Sentinel Node Lymphocytes: Tumour Reactive Lymphocytes Identified Intraoperatively for the Use in Immunotherapy of Colon Cancer." *British Journal of Cancer* 94(10): 1478–84.

Mitrus, I., Smagur, A., Fidyk, W. et al. 2018. "Reduction of DMSO concentration in cryopreservation mixture from 10% to 7.5% and 5% has no impact on engraftment after autologous peripheral blood stem cell transplantation: results of a prospective, randomized study." *Bone Marrow Transplant* 53, 274–280.

Morgan, Richard A. et al. 2006. "Cancer Regression in Patients After Transfer of Genetically Engineered Lymphocytes." *Science* 314(5796): 126–29.

Muñoz, L., Lauber, K., Schiller, M. et al. 2010. "The role of defective clearance of apoptotic cells in systemic autoimmunity. *Nat Rev Rheumatol* 6; 280–289.

"National Cancer Institute Cancer Therapy Evaluation Program. Common Terminology Criteria for Adverse Events, CTCAE v5.0." 2017. https://ctep.cancer.gov/protocoldevelopment/electronic_applications/ctc.htm (January 24, 2022).

Neelapu, Sattva S. 2019. "Managing the Toxicities of CAR T-cell Therapy." *Hematological Oncology* 37(S1): 48–52.

Pak, H; Haji Maghsoudib, L; Soltaniana, A; Gholami, F. 2020. "Surgical complications in colorectal cancer patients." *Ann of Med & Surg.* 55:13-18.

Parkhurst, Maria et al. 2024. "Adoptive transfer of personalized neoantigen-reactive TCR-transduced T cells in metastatic colorectal cancer: phase 2 trial interim results." *Nature Medicine* 30, 2586–2595.

Petrozziello, Elisabetta, Tabea Sturmheit, and Anna Mondino. 2015. "Exploiting Cytokines in Adoptive T-Cell Therapy of Cancer." *Immunotherapy* 7(5): 573–84.

Pockaj, Barbara A. et al. 1994. "Localization of 111Indium-Labeled Tumor Infiltrating Lymphocytes to Tumor in Patients Receiving Adoptive Immunotherapy. Augmentation with Cyclophosphamide and Correlation with Response." *Cancer* 73(6): 1731–37.

Ramos, Carlos A et al. 2016. "Clinical Responses with T Lymphocytes Targeting Malignancy-Associated κ Light Chains." *The Journal of clinical investigation* 126(7): 2588–96.

Rawla, Prashanth, Tagore Sunkara, and Adam Barsouk. 2019. “Epidemiology of Colorectal Cancer: Incidence, Mortality, Survival, and Risk Factors.” *Gastroenterology Review* 14(2): 89–103.

Robbins, Paul F et al. 2011. “Tumor Regression in Patients with Metastatic Synovial Cell Sarcoma and Melanoma Using Genetically Engineered Lymphocytes Reactive with NY-ESO-1.” *Journal of clinical oncology : official journal of the American Society of Clinical Oncology* 29(7): 917–24.

Rohaan, Maartje W., Borch Troels H., van den Berg Joost H., Met Özcan, Kessels Rob, Geukes Foppen Marnix H., Stoltenborg Granhøj Joachim, et al. 2022. “Tumor-Infiltrating Lymphocyte Therapy or Ipilimumab in Advanced Melanoma.” *New England Journal of Medicine* 387(23): 2113–25.

Rosenberg, Steven A et al. 2011. “Durable Complete Responses in Heavily Pretreated Patients with Metastatic Melanoma Using T-Cell Transfer Immunotherapy.” *Clinical cancer research : an official journal of the American Association for Cancer Research* 17(13): 4550–57.

Rosenberg, Steven A. 2014. “IL-2: The First Effective Immunotherapy for Human Cancer.” *The Journal of Immunology* 192(12): 5451–58.

Sarnaik, Amod A et al. 2021. “Lifileucel, a Tumor-Infiltrating Lymphocyte Therapy, in Metastatic Melanoma.” *Journal of clinical oncology : official journal of the American Society of Clinical Oncology* 39(24): 2656–66.

Scarborough, JE; Schumacher, J; Kent, KC; Heise, CP, Greenberg, CC. 2017. “Associations of Specific Postoperative Complications With Outcomes After Elective Colon Resection. A Procedure-Targeted Approach Toward Surgical Quality Improvement.” *JAMA Surg.* 152(2):e164681.

Schiavone, Ettore Mariano et al. 2003. “Fludarabine plus Cyclophosphamide for the Treatment of Advanced Chronic Lymphocytic Leukemia.” *European Journal of Haematology* 71(1): 23–28.

Schuermans, S., Kestens, C. & Marques, P.E. 2024. “Systemic mechanisms of necrotic cell debris clearance.” *Cell Death Dis* 15, 557.

Schumacher, Ton N, and Robert D Schreiber. 2015. “Neoantigens in Cancer Immunotherapy.” *Science* 348(6230): 69 LP – 74.

Sciuto A, Merola G, De Palma GD, Sodo M, Pirozzi F, Bracale UM, Bracale U. 2018. “Predictive factors for anastomotic leakage after laparoscopic colorectal surgery.” *World J Gastroenterol.* 7;24(21):2247-2260.

Seymour L et al. 2017. “RECIST working group. iRECIST: guidelines for response criteria for use in trials testing immunotherapeutics.” *Lancet Oncol*.18(3):e143-e152. Erratum in: *Lancet Oncol.* 2019 20(5):e242. d

Sherif, Amir et al. 2015. “Pilot Study of Adoptive Immunotherapy with Sentinel Node-Derived T Cells in Muscle-Invasive Urinary Bladder Cancer.” *Scandinavian Journal of Urology* 49(6): 453–62.

Sheth, Vipul S, and Jordan Gauthier. 2021. “Taming the Beast: CRS and ICANS after CAR T-Cell Therapy for ALL.” *Bone Marrow Transplantation* 56(3): 552–66.

Sibille, Michel, Alain Patat, Henri Caplain, and Yves Donazzolo. 2010. “A Safety Grading Scale to Support Dose Escalation and Define Stopping Rules for Healthy Subject First-Entry-into-Man Studies.” *British Journal of Clinical Pharmacology* 70(5): 736–48.

Sureda Anna et al, 2024.The EBMT Handbook, 2024 Edition. p. 534. https://doi.org/10.1007/978-3-031-44080-9.

Sveen, Anita, Scott Kopetz, and Ragnhild A. Lothe. 2020. “Biomarker-Guided Therapy for Colorectal Cancer: Strength in Complexity.” *Nature Reviews Clinical Oncology* 17(1): 11–32.

Tecelra FDA approval, August 2nd 2024. https://www.fda.gov/media/180981/download?attachment

"The World Medical Association. Declaration of Helsinki – Ethical Principles for Medical Research Involving Human Subjects." 2018. https://www.wma.net/policies-post/wma-declaration-of-helsinki-ethical-principles-for-medical-research-involving-human-subjects (January 24, 2022).

Tinegate et al. 2012. Guideline on the investigation and management of acute transfusion reactions Prepared by the BCSH Blood TransfusionTask Force, *Br J Haemat* 159,143–153.

Tosolini, Marie, et al. 2011 "Clinical impact of different classes of infiltrating T cytotoxic and helper cells (Th1, th2, treg, th17) in patients with colorectal cancer." *Cancer research* 71.4: 1263-1271.

Tran, Eric et al., 2016. "T-Cell Transfer Therapy Targeting Mutant KRAS in Cancer." *N Engl J Med*. 375(23):2255-2262.

Tsimberidou, Apostolia et al. 2020. "Results of the First-in-Human Clinical Trial with Personalized Multi-Target Adoptive Cell Therapy (ACTolog IMA101). SITC Poster 293." *Journal for ImmunoTherapy of Cancer* 8(Suppl 3): A320–A320.

Turajlic, Samra et al. 2021. "Sensitive Quantification and Tracking of the Active Components of a Clonal Neoantigen T Cell (CNeT) Therapy: From Manufacture to Peripheral Circulation. SITC Poster 543." *Journal for ImmunoTherapy of Cancer* 9(Suppl 2): A572–A572.

Turnstone Biologics Press Release August 14 2024. https://ir.turnstonebio.com/news-releases/news-release-details/turnstone-biologics-corp-reports-positive-initial-data-phase-1

Wang LX, Shu S, Disis ML, Plautz GE. 2007. "Adoptive transfer of tumor-primed, in vitro-activated, CD4+ T effector cells (TEs) combined with CD8+ TEs provides intratumoral TE proliferation and synergistic antitumor response." *Blood* 109:4865-76.

Ward, Jeffrey P, Matthew M Gubin, and Robert D Schreiber. 2016. "The Role of Neoantigens in Naturally Occurring and Therapeutically Induced Immune Responses to Cancer." *Advances in immunology* 130: 25–74.

Watz, Emma et al. 2015. "Quality of the Hematopoietic Stem Cell Graft Affects the Clinical Outcome of Allogeneic Stem Cell Transplantation." *Transfusion* 55(10): 2339–50.

Xi, Yue, and Pengfei Xu. 2021. "Global Colorectal Cancer Burden in 2020 and Projections to 2040." *Translational Oncology* 14(10): 101174.

Yáñez, Lucrecia, Miriam Sánchez-Escamilla, and Miguel-Angel Perales. 2019. "CAR T Cell Toxicity: Current Management and Future Directions." *HemaSphere* 3(2): e186.

Yang, Shicheng et al. 2011. "In Vitro Generated Anti-Tumor T Lymphocytes Exhibit Distinct Subsets Mimicking in Vivo Antigen-Experienced Cells." *Cancer Immunology, Immunotherapy* 60(5): 739–49.

Ying, Zhitao et al. 2019. "A Safe and Potent Anti-CD19 CAR T Cell Therapy." *Nature Medicine* 25(6): 947–53.

Zambelli, A et al. 1998. "Clinical Toxicity of Cryopreserved Circulating Progenitor Cells Infusion." *Anticancer research* 18(6B): 4705–8.

Zarnescu, E.C.; Zarnescu, N.O.; Costea, R. 2021. "Updates of Risk Factors for Anastomotic Leakage after Colorectal Surgery." *Diagnostics* 11, 2382.

Zhang, Chengcheng et al. 2017. "Phase I Escalating-Dose Trial of CAR-T Therapy Targeting CEA^+ Metastatic Colorectal Cancers." *Molecular Therapy* 25(5): 1248–58.

Zhen, Yun Huan et al. 2015. "Phase I/II Study of Adjuvant Immunotherapy with Sentinel Lymph Node T Lymphocytes in Patients with Colorectal Cancer." *Cancer Immunology, Immunotherapy* 64(9): 1083–93.

Zhou, Juhua, Prakash Nagarkatti, Yin Zhong, and Mitzi Nagarkatti. 2011. "Characterization of T-Cell Memory Phenotype after in Vitro Expansion of Tumor-Infiltrating Lymphocytes from Melanoma Patients." *Anticancer research* 31(12): 4099–4109.

19 SIGNATURES

19.1 Principal Investigator statement

I have read and understood this CTP and agree to conduct the trial accordingly and to comply with the Investigator obligations stated in this CTP, GCP and applicable regulatory requirements.

Principal Investigator

____

[redacted], MD, PhD, Associate Professor
Västmanlands hospital Västerås, Department of Surgery
Recruitment and Surgery Site 1.

Principal Investigator

____

[redacted], MD, PhD, Professor
Danderyd's Hospital AB, Department of Surgery and Urology
Recruitment and Surgery Site 2.

19.2 Signature page (approval of the clinical trial protocol)

Sponsor signatories

____

[redacted], CEO Neogap Therapeutics AB

____

[redacted], Medical Representative, Neogap Therapeutics AB

Coordinating Investigator/Principal Investigator

____

[redacted], MD, PhD
Karolinska University Hospital, Solna – Recruitment, Surgery & Treatment Site
