# Supplementary material for: A first-in-human, phase I/IIa trial of the T cell immunotherapy personalised tumour trained lymphocytes in patients with advanced colorectal cancer: Study protocol
Source: PLoS One. 2026 Sep 18;21(9):e0351697. doi: 10.1371/journal.pone.0351697 (PMC13588363; doi:10.1371/journal.pone.0351697)
Supplement: Supplementary file 3 [file pone.0351697.s004.docx]

**Response to editors and reviewers**

**Editors´ comments**

- **Comment 1:**

Please ensure that your manuscript meets PLOS ONE's style requirements, including those for file naming.

Response: The manuscript has now been updated in accordance with the PLOS ONE style guidelines.

- **Comment 2:**
  You indicated that you had ethical approval for your study. In your Methods section, please ensure you have also stated whether you obtained consent from parents or guardians of the minors included in the study or whether the research ethics committee or IRB specifically waived the need for their consent.

  Response: Under Eligibility the manuscript states: “The target group of the study is adults (≥18 years of age) with stage IV CRC”. To further clarify the eligibility criteria, we have revised this statement to: *“The target group of the study is adults (≥18 years of age) with stage IV CRC who are able to provide written informed consent.”* A sentence has been added to the Ethical considerations section for clarification, stating: *“Subjects ≥18 years are eligble for participation in the study and their informed concent is obtained prior to trial inclusion.”*
- **Comment 3:**
  We note that you have selected “Clinical Trial” as your article type. PLOS ONE requires that all clinical trials are registered in an appropriate registry (the WHO list of approved registries. Please state the name of the registry and the registration number in the submission data and on the **title page** of your manuscript.

Response: The trial is registered in ClinicalTrials.gov with the ID NCT05908643. The registration number is now moved to the **title page**.

**a)** Please provide the complete date range for participant recruitment and follow-up in the methods section of your manuscript.

Response: The complete date range, including the date of study opening and the projected date of completion, is now clearly stated in the Methods section under the heading “Trial status”.

**b)** Trial registration:

Response: The trial is authorized under the Clinical Trial Regulation (Regulation EU No 536/2014), EU CT #2024-512296-13-00. It is registered on clinicaltrials.gov, ID NCT05908643.

- **Comment 4:**
  Your ethics statement should only appear in the Methods section of your manuscript. If your ethics statement is written in any section besides the Methods, please delete it from any other section.

  Response: the ethics statement is only mentioned in the Methods section.
- **Comment 5:**
  Please provide a complete Data Availability Statement in the submission form, ensuring you include all necessary access information or a reason for why you are unable to make your data freely accessible. If your research concerns only data provided within your submission, please write "All data are in the manuscript and/or supporting information files" as your Data Availability Statement.

  Response: All data are in the manuscript and/or supporting information files. A statement to that effect has been added on page 3 under Data availability and on page 21, under the heading Data management.
- **Comment 6:**
  Role of Funders.

  Response: As previously stated, the study is sponsored by Neogap Therapeutics AB (Neogap), and Neogap is in consequence responsible for the study design, protocol development, and preparation of the manuscript. The European Innovation Council provided co-funding but had no role in study design, data collection and analysis, decision to publish, or preparation of the manuscript. As requested, this amended Role of Funder statement has also been included in the manuscript.
- **Comment 7:**
  The competing interests’ section.

  Response: We have updated the Competing Interests statement by including the requested statement confirming that the declared competing interests do not alter our adherence to PLOS ONE policies on sharing data and materials. The updated Competing Interests statement has also been included in the cover letter.
- **Comment 8:**
  Please amend either the title on the online submission form (via Edit Submission) or the title in the manuscript so that they are identical.

  Response: The title has been corrected and is now identical in both the online submission form and the manuscript.
- **Comment 9:**
  Please amend your authorship list in your manuscript file to include author Ahmed Tarfy.

  Response: The Author Ahmed Tarfy is now correctly included in the manuscript.
- **Comment 10:**
  Please amend the manuscript submission data (via Edit Submission) to include author Ahmed Salim Tarfy.

  Response: The author's name has been corrected to Ahmed Tarfy and is now used consistently throughout the manuscript and the submission system.
- **Comment 11:**
  Please include a caption for figure 1.

  Response: A caption has now been added to Figure 1 identifying it as the SPIRIT 2025 checklist and describing its purpose.
- **Comment 12:**
  Please include captions for your Supporting Information files at the end of your manuscript, and update any in-text citations to match accordingly.

  Response: We have added a new section entitled *Supporting Information* after the References section, where captions for all supporting information are provided.

**Reviewers' comments:**

**Reviewer #1. We thank the reviewer for all valuable comments, which will improve the clarity of the description of statistical analysis. Please find our point-by-point responses below:**

- **Comment 1:**
  There is no specification of statistical tests (e.g., methods for time-to-event analyses, comparisons, or handling of exploratory endpoints). 

  Response: As this is a first-in-human, single-arm phase I/IIa study primarily designed to evaluate safety and feasibility, no formal hypothesis testing is planned. We have revised the Statistical Analysis section to clarify that safety data will be summarized descriptively, time-to-event endpoints will be presented using Kaplan–Meier methodology, and exploratory endpoints will be reported descriptively.
- **Comment 2:**
  The protocol does not describe methods for handling missing data, which is important in longitudinal and follow-up analyses. 

  Response: The Statistical Analysis section has been revised to clarify that missing data will not be imputed, analyses will be based on available data, and the number of evaluable participants will be reported for each endpoint.
- **Comment 3:**
  There is no discussion of multiplicity or control of type I error, particularly given multiple secondary and exploratory endpoints.

  Response: As this first-in-human phase I/IIa study is not designed for confirmatory hypothesis testing, no formal control of the type I error rate or adjustment for multiplicity is planned. We have clarified this in the Statistical Analysis section and specified that secondary and exploratory endpoints will be analysed descriptively.
- **Comment 4:**
  The protocol lacks detail on analysis populations (e.g., safety, intent-to-treat, per-protocol).

  Response: The Statistical Analysis section has been revised to specify that the Safety Population will include all participants who receive a pTTL infusion, while secondary and exploratory endpoints will be analysed using participants with evaluable data for the respective endpoint.
- **Comment 5:**
  While data capture software is specified, the statistical software for analysis is not identified.

  Response: All descriptive summaries and statistical analyses will be performed using SAS Version 9.4 or later (SAS Institute, Inc., Cary, NC). The statistical software is now available in the manuscript.
- **Comment 6:**
  There is limited detail on planned data presentation (e.g., confidence intervals, or estimation approaches).

Response: A separate section with the subheading “Data presentation” has been added at the end of the statistical analysis section to clarify this question.

**Comment 7:**
Overall, the statistical section would benefit from a more detailed and prespecified analysis framework to enhance transparency and reproducibility.

Response: The authors thank the reviewer for turning our attention to the need for further information in the statistical analysis section.
 In response to the comments, we have revised and expanded the Statistical Analysis section to provide a more detailed and prespecified analysis framework, including the planned analyses, analysis populations, handling of missing data, data presentation, and statistical software. We believe these revisions improve the transparency and reproducibility of the protocol.

**Reviewer #2. We thank the reviewer for all valuable comments. Please find our point-by-point responses below:**

- **Comment 1:**
  This work was supported by Neogap Therapeutics AB for the development of pTTL. Suggestions from related experts should be carefully considered by the editors of Plos One for the possible competing interests.

  Response: The funding source and all potential competing interests have been fully disclosed in the manuscript and the cover letter in accordance with the PLOS ONE requirements.
- **Comment 2:**
  In the background, Reference 10-21 were cited as a whole to support TIL therapy, which was inappropriate.

  Response: The references were intended to support the entire paragraph rather than only the statements related to TIL therapy. To improve clarity, we have revised the paragraph by assigning references to the specific statements they support. References relating to TIL therapy are now cited separately from those describing other forms of adoptive T-cell therapy.
- **Comment 3:**
  Was pTTL successful for the treatment of metastatic melanoma or gastrointestinal tumours cited in the Introduction? What was the traditional or the most successful treatment for colorectal cancer? Why did the protocol choose Stage IV CRC? How to classify the period of CRC? The information about CRC was not enough.

  Response: The Introduction already describes the current standard treatment and prognosis of colorectal cancer across different disease stages, including stage IV disease. To further strengthen the clinical background, we have added a paragraph describing the current standard-of-care (SOC) treatment for stage IV colorectal cancer and the limited treatment options available for patients with unresectable disease. As this is a first-in-human study, pTTL has not previously been evaluated in any cancer type. Patients with stage IV CRC were selected because this population has exhausted standard treatment options and represents a group with a high unmet medical need. From a regulatory and ethical perspective, only patients lacking effective treatment option can be treated in a first-in-human trial.
- **Comment 4:**
  Were 16 patients enough? Were there control patients? Was there some comparison plan with other treatments?

Response: The study is a first-in-human, single-arm phase I/IIa trial primarily designed to assess the safety, tolerability, and feasibility of pTTL treatment. The sample size of up to 16 treated participants, with a target of at least 12 evaluable participants, was considered appropriate for the descriptive evaluation of these objectives. The study was not designed or powered to establish comparative efficacy. Accordingly, no control group is included and no formal comparison with other treatments is planned in the first-in-human trial. Secondary efficacy and explorative outcomes will be described within the treated population and interpreted cautiously.

- **Comment 5:**
  Was the time of different phases during the trial enough? For example, the reason why it chose 26 weeks of post-infusion was not clear. And was the 5-year following-up enough?

Response: We have clarified the rationale for the selected follow-up periods in the Methods section. The 26-week follow-up period was selected because the vast majority of treatment-related adverse events are expected to occur within the first six months after pTTL infusion, allowing assessment of the primary safety endpoint and clinical outcomes. The subsequent follow-up of up to 5 years is included in accordance with requirements from the Swedish Medical Products Agency and enables the assessment of long-term safety and survival outcomes.

- **Comment 6:**
  Was eligibility a necessary factor for the patient choosing? For example, it was difficult to understand the trial process and experiment details even like me.

  Response: As this is a the first time pTTL is applied on humans, it is critical that the eligibility criteria ensure that the patients can tolerate pTTL treatment, including preconditioning lymphodepletion. This requires good organ function and an overall sufficiently good health status. The patient eligibility criteria and trial design are designed with this in mind, and includes requirements for adequate laboratory test results, a cardiac ejection fraction at minimum 45%, and all patients with any symtoms undergo spirometry to ensure adequate lung function. No specific criteria for weight is included, but a requirement for eastern collaborative oncology group (ECOG) performance status of 0-1, means that only asymptomatic and mildly asymptomatic patients who can perform activities of daily life without effort can be included. This is described in the Eligibilty section of the manuscript. We believe these requirements are sufficient to prevent patients with unacceptable risk for complications from being included.
- **Comment 7:**
  Other successful and unsuccessful protocols should be compared. How to evaluate the feasibility or practicability of this protocol？At least, how to guarantee evaluable 12 patients out of 16 within 5 years?

Response: The Discussion intends to place the present study in the context of previous adoptive T-cell therapy studies, by including references to conventional TIL therapy evaluated in the C-144-01 and M14TIL trials, as well as neoantigen-targeted TCR-engineered T-cell therapy reported by Parkhurst et al. The feasibility of the present protocol will be evaluated descriptively, including successful completion of tissue collection, pTTL manufacturing, treatment administration, and follow-up.

The study aims to fulfil the primary objective to show safety and tolerability of treatment with pTTL, which is anticipated to be feasible by including 12 evaluable participants in Part II. To account for potential attrition before treatment, no fixed upper limit is applied to enrolment in Part I, and recruitment is continuously assessed and adjusted to support achievement of the target number of evaluable treated participants.

**Reviewer #3. We thank the reviewer for all important comments. Please find our point-by-point responses below.**

- **Comment 1:**

Naming Convention & Title Overstatement: The title claims this is a "novel T cell immunotherapy." While the selection platform (PIOR and activation technology EpiTCer) are proprietary, the concept of using tumor-draining regional lymph nodes (RLNs) to expand tumor-reactive lymphocytes shares fundamental lineages with traditional Tumor-Infiltrating Lymphocyte (TIL) therapies. It is mathematically and historically incorrect to market the broad umbrella of the therapy as completely unprecedented.
Therefore, authors should refine the title or strictly clarify in the introduction what makes "Personalized Tumor Trained Lymphocytes" distinct from standard TIL or Marrow-Infiltrating Lymphocyte (MIL) protocols. Consider changing "novel T cell immunotherapy" to "novel neoantigen-selected T cell manufacturing approach (pTTL).

Response: We agree that the term *novel* in the title could be interpreted as implying that the overall concept of tumour-reactive T-cell therapy is unprecedented. To avoid this interpretation, we have revised the title by removing the term *novel*.

- **Comment 2:**
  Lack of HLA Restriction & Neoantigen Characterization: The abstract notes that optimal neoantigen epitopes are identified via bioinformatics and loaded onto paramagnetic particles (TC0301). However, the manuscript fails to mention the targeted Human Leukocyte Antigen (HLA) framework. It is unclear whether this platform is limited to HLA Class I (CD8+ restrictive), HLA Class II (CD4+ restrictive), or a pan-HLA presentation.
  Therefore, authors must define the mechanistic scope of the PIOR algorithm. Does it select peptides with predicted binding affinity for both MHC-I and MHC-II? Providing the baseline parameters of the target mutations/peptide lengths is critical for peer assessment of the trial's scientific validity.

  Response: The mechanistic scope of the PIOR® and EpiTCer® platforms is described in the *Personalized tumour-trained lymphocytes, pTTL* section of the manuscript. Up to 36 neoantigenic epitopes are selected based on the individual patient's mutational landscape and expressed as recombinant proteins including 21-amino acid sequences including the neoantigenic epitopes and subsequently coupled to EpiTCer® beads. Following uptake by antigen-presenting cells, these proteins are processed through the natural antigen-processing machinery and presented as shorter peptides on both MHC-I and MHC-II molecules in an HLA-independent manner, enabling expansion of both CD4+ and CD8+ T cells. Some further details have been added to the manuscript to clarify the concept. We believe that this provides the level of methodological detail appropriate for a clinical trial protocol. Detailed technical specifications of the proprietary PIOR® bioinformatics platform, including its ranking algorithm and peptide selection parameters, are beyond the scope of the present manuscript.
- **Comment 3:**
  Patient Selection Safeguards: Cytokine Release Syndrome (CRS) & Lymphodepletion: The protocol utilizes a fludarabine/cyclophosphamide lymphodepletion preconditioning regimen. While the authors state that pTTL has "comparably mild side effects," cellular infusions following lymphodepletion carry an inherent clinical risk of CRS, immune effector cell-associated neurotoxicity syndrome (ICANS), or severe cytopenias. Recruiting unselected Stage IV CRC patients who may be heavily pretreated, cachectic, or immunologically depleted, without rigorous baseline physiological filters poses a severe safety vulnerability.
  Therefore, the inclusion criteria may include a strict physiological threshold to ensure patients can tolerate potential cytokine storms and preconditioning stress. The authors should incorporate specific enrolment criteria based on: Body Mass Index (BMI) and/or Cachexia Status. To screen out severe cancer cachexia, which compromises cardiac and respiratory reserves during systemic immune activation. 

  Response: Patient safety has been a primary consideration in the study design. The study does not recruit unselected patients with stage IV CRC; eligibility is restricted through comprehensive inclusion and exclusion criteria, including requirements for adequate performance status, organ function, and haematological parameters to ensure that participants are able to tolerate lymphodepleting chemotherapy and pTTL treatment. Inclusion is also restricted to patients with Eastern cooperative oncology group performance status 0-1, which is defined as asymptomatic or only mildly symptomatic but fully able to perform activities of daily life. We therefore believe that the existing eligibility criteria provide appropriate safeguards.
- **Comment 4:**
  Definition of Cell Yield and Dose Limits: The protocol states that "between the limits of 20x106 - 1x109 cells, the entire pTTL yield will be administered." A fifty-fold variance between the minimum and maximum cell dose variables need to be consideration into this Phase I safety trial.
  Authors should elaborate or clarify how they intend to correlate toxicity endpoints if Patient A receives 20x106 cells and Patient B receives 1x109 cells.

  Response: The manuscript has been revised to explain that free dosing within the predefined dose range has been approved by the Swedish Medical Products Agency, taking into account the expected safety profile of pTTL, the unique characteristics of this autologous, patient-specific product, and current regulatory guidance for Advanced Therapy Medicinal Products (ATMPs). The manufacturing yield and pTTL characteristics varies significantly between the batches due to biological and technical factors, including the characteristics and fitness of the T cells in the lymph node starting material and the patient-specific neoantigens used to produce each pTTL batch. This means that each pTTL product is unique, and that direct comparisons between products is considered of limited value. Administration of the entire feasible product yield is thus considered appropriate. The primary objective of this first-in-human trial is to characterize the safety profile across the feasible dose range rather than to determine a maximum tolerated dose, consistent with current EMA and FDA guidance for early-phase cellular therapy trials.
