# Supplementary material for: A first-in-human, phase I/IIa trial of the T cell immunotherapy personalised tumour trained lymphocytes in patients with advanced colorectal cancer: Study protocol
Source: PLoS One. 2026 Sep 18;21(9):e0351697. doi: 10.1371/journal.pone.0351697 (PMC13588363; doi:10.1371/journal.pone.0351697)
Supplement: Peer review history

# Peer review: Decision Letter 0

**DOI:** 10.1371/journal.pone.0351697.r001

**Authors:** Michael Burger

5 Jul 2026

Dear Dr. Berglund,

Thank you for submitting your manuscript to PLOS ONE. After careful consideration, we feel that it has merit but does not fully meet PLOS ONE’s publication criteria as it currently stands. Therefore, we invite you to submit a revised version of the manuscript that addresses the points raised during the review process.

Please adjust the manuscript according to the suggestions brought forward by the reviewers, and discuss the reasons where this might not be feasible.

Please submit your revised manuscript by Aug 19 2026 11:59PM. If you will need more time than this to complete your revisions, please reply to this message or contact the journal office at plosone@plos.org. When you're ready to submit your revision, log on to https://www.editorialmanager.com/pone/ and select the 'Submissions Needing Revision' folder to locate your manuscript file.

- A letter that responds to each point raised by the academic editor and reviewer(s). You should upload this letter as a separate file labeled 'Response to Reviewers'.
- A marked-up copy of your manuscript that highlights changes made to the original version. You should upload this as a separate file labeled 'Revised Manuscript with Track Changes'.
- An unmarked version of your revised paper without tracked changes. You should upload this as a separate file labeled 'Manuscript'.

If you would like to make changes to your financial disclosure, please include your updated statement in your cover letter. Guidelines for resubmitting your figure files are available below the reviewer comments at the end of this letter.

If applicable, we recommend that you deposit your laboratory protocols in protocols.io to enhance the reproducibility of your results. Protocols.io assigns your protocol its own identifier (DOI) so that it can be cited independently in the future. For instructions see: https://journals.plos.org/plosone/s/submission-guidelines#loc-laboratory-protocols. Additionally, PLOS ONE offers an option for publishing peer-reviewed Lab Protocol articles, which describe protocols hosted on protocols.io. Read more information on sharing protocols at https://plos.org/protocols?utm_medium=editorial-email&utm_source=authorletters&utm_campaign=protocols.

As the corresponding author, your ORCID iD is verified in the submission system and will appear in the published article. PLOS supports the use of ORCID, and we encourage all coauthors to register for an ORCID iD and use it as well. Please encourage your coauthors to verify their ORCID iD within the submission system before final acceptance, as unverified ORCID iDs will not appear in the published article. *Only*  the individual author can complete the verification step; PLOS staff *cannot*  verify ORCID iDs on behalf of authors.

We look forward to receiving your revised manuscript.

Kind regards,

Michael C Burger, M.D.

Academic Editor

PLOS One

Journal Requirements:

When submitting your revision, we need you to address these additional requirements.

1. Please ensure that your manuscript meets PLOS ONE's style requirements, including those for file naming. The PLOS ONE style templates can be found at

https://journals.plos.org/plosone/s/file?id=wjVg/PLOSOne_formatting_sample_main_body.pdf and https://journals.plos.org/plosone/s/file?id=ba62/PLOSOne_formatting_sample_title_authors_affiliations.pdf

2. You indicated that you had ethical approval for your study. In your Methods section, please ensure you have also stated whether you obtained consent from parents or guardians of the minors included in the study or whether the research ethics committee or IRB specifically waived the need for their consent.

3. We note that you have selected “Clinical Trial” as your article type. PLOS ONE requires that all clinical trials are registered in an appropriate registry (the WHO list of approved registries is at https://www.who.int/clinical-trials-registry-platform/network/primary-registries" https://www.who.int/clinical-trials-registry-platform/network/primary-registries and more information on trial registration is at http://www.icmje.org/about-icmje/faqs/clinical-trials-registration/). Please state the name of the registry and the registration number (e.g. ISRCTN or

ClinicalTrials.gov) in the submission data and on the title page of your manuscript. a) Please provide the complete date range for participant recruitment and follow-up in the methods section of your manuscript. b) If you have not yet registered your trial in an appropriate registry, we now require you to do so and will need confirmation of the trial registry number before we can pass your paper to the next stage of review. Please include in the Methods section of your paper your reasons for not registering this study before enrolment of participants started. Please confirm that all related trials are registered by stating: “The authors confirm that all ongoing and related trials for this drug/intervention are registered”. Please see http://journals.plos.org/plosone/s/submission-guidelines#loc-clinical-trials for

our policies on clinical trials.

4. Your ethics statement should only appear in the Methods section of your manuscript. If your ethics statement is written in any section besides the Methods, please delete it from any other section.

5. Please provide a complete Data Availability Statement in the submission form, ensuring you include all necessary access information or a reason for why you are unable to make your data freely accessible. If your research concerns only data provided within your submission, please write "All data are in the manuscript and/or supporting information files" as your Data Availability Statement.

6. Thank you for stating the following financial disclosure:

This trial is sponsored by Neogap Therapeutics AB and is co-funded by the European Union, European Innovation Council project 190185439-NEOpTTL. An invitation letter and documentation of a bank transfer of part of the funding is submitted with the manuscript.

Please state what role the funders took in the study. If the funders had no role, please state: "The funders had no role in study design, data collection and analysis, decision to publish, or preparation of the manuscript."

If this statement is not correct you must amend it as needed.

Please include this amended Role of Funder statement in your cover letter; we will change the online submission form on your behalf.

7. Thank you for stating the following in the Competing Interests section:

AST, MN, and AC receive research funding from Neogap Therapeutics AB for the development of pTTL. SB, AS, GG, and HG are employed by Neogap Therapeutics AB.

Please confirm that this does not alter your adherence to all PLOS ONE policies on sharing data and materials, by including the following statement: "This does not alter our adherence to PLOS ONE policies on sharing data and materials.” (as detailed online in our guide for authors http://journals.plos.org/plosone/s/competing-interests). If there are restrictions on sharing of data and/or materials, please state these. Please note that we cannot proceed with consideration of your article until this information has been declared.

Please include your updated Competing Interests statement in your cover letter; we will change the online submission form on your behalf.

8. Please amend either the title on the online submission form (via Edit Submission) or the title in the manuscript so that they are identical.

9. Please amend your authorship list in your manuscript file to include author Ahmed Tarfy.

10. Please amend the manuscript submission data (via Edit Submission) to include author Ahmed Salim Tarfy.

11. Please include a caption for figure 1.

12. Please include captions for your Supporting Information files at the end of your manuscript, and update any in-text citations to match accordingly. Please see our Supporting Information guidelines for more information: http://journals.plos.org/plosone/s/supporting-information.

13. If the reviewer comments include a recommendation to cite specific previously published works, please review and evaluate these publications to determine whether they are relevant and should be cited. There is no requirement to cite these works unless the editor has indicated otherwise.

[Note: HTML markup is below. Please do not edit.]

Reviewers' comments:

Reviewer's Responses to Questions

**Comments to the Author**

1. Does the manuscript provide a valid rationale for the proposed study, with clearly identified and justified research questions?

Reviewer #1: Yes

Reviewer #2: Yes

Reviewer #3: Yes

**********

2. Is the protocol technically sound and planned in a manner that will lead to a meaningful outcome and allow testing the stated hypotheses?

Reviewer #1: Yes

Reviewer #2: Yes

Reviewer #3: Yes

**********

3. Is the methodology feasible and described in sufficient detail to allow the work to be replicable?

Reviewer #1: Yes

Reviewer #2: Yes

Reviewer #3: Yes

**********

4. Have the authors described where all data underlying the findings will be made available when the study is complete?

The PLOS Data policy

Reviewer #1: No

Reviewer #2: Yes

Reviewer #3: Yes

**********

5. Is the manuscript presented in an intelligible fashion and written in standard English?

Reviewer #1: Yes

Reviewer #2: Yes

Reviewer #3: Yes

**********

Please use the space provided to explain your answers to the questions above and, if applicable, provide comments about issues authors must address before this protocol can be accepted for publication. You may also include additional comments for the author, including concerns about research or publication ethics.

You may also provide optional suggestions and comments to authors that they might find helpful in planning their study.

Reviewer #1: This study protocol outlines a first-in-human phase I/IIa trial of a personalized T‑cell therapy (pTTL) for patients with advanced colorectal cancer and limited treatment options. The therapy uses T cells from tumor-draining lymph nodes that are expanded and trained to target patient-specific neoantigens, then infused after chemotherapy. Up to 16 patients will be accrued across manufacturing, treatment, and follow-up phases. The primary goal is to assess safety, with secondary aims evaluating preliminary efficacy, survival outcomes, and immune responses.

Minor recommended revisions:

1- There is no specification of statistical tests (e.g., methods for time-to-event analyses, comparisons, or handling of exploratory endpoints).

2- The protocol does not describe methods for handling missing data, which is important in longitudinal and follow-up analyses.

3-There is no discussion of multiplicity or control of type I error, particularly given multiple secondary and exploratory endpoints.

4- The protocol lacks detail on analysis populations (e.g., safety, intent-to-treat, per-protocol).

5- While data capture software is specified, the statistical software for analysis is not identified.

6- There is limited detail on planned data presentation (e.g., confidence intervals, or estimation approaches).

7- Overall, the statistical section would benefit from a more detailed and prespecified analysis framework to enhance transparency and reproducibility.

Reviewer #2: The manuscript of “A first-in-human, phase I/IIa trial of the novel T cell immunotherapy personalized tumour trained lymphocytes in patients with advanced colorectal cancer: study protocol” with No. of PONE-D-26-15858 was a study protocol for a trial design for the pTTL therapy in human. It was very important and the first protocol in human for CRC. However, there existed several problems. Thus, it should be accepted after major revision by Plos One.

(1) This work was supported by Neogap Therapeutics AB for the development of pTTL. Suggestions from related experts should be carefully considered by the editors of Plos One for the possible competing interests.

(2) In the background, Reference 10-21 were cited as a whole to support TIL therapy, which was inappropriate.

(3) Was pTTL successful for the treatment of metastatic melanoma or gastrointestinal tumours cited in the Introduction? What was the traditional or the most successful treatment for colorectal cancer? Why did the protocol choose Stage IV CRC? How to classify the period of CRC? The information about CRC was not enough.

(4) Were 16 patients enough? Were there control patients? Was there some comparison plan with other treatments?

(5) Was the time of different phases during the trial enough? For example, the reason why it chose 26 weeks of post-infusion was not clear. And was the 5-year following-up enough?

(6) Was eligibility a necessary factor for the patient choosing? For example, it was difficult to understand the trial process and experiment details even like me.

(7) Other successful and unsuccessful protocols should be compared. How to evaluate the feasibility or practicability of this protocol？At least, how to guarantee evaluable 12 patients out of 16 within 5 years?

Overall, editors should carefully consider the suggestions from all reviewers and carefully make the decision.

Reviewer #3: REVIEW REPORT:

Manuscript Title: A first-in-human, phase I/IIa trial of the novel T cell immunotherapy personalized tumor trained lymphocytes in patients with advanced colorectal cancer: study protocol.

Trial Name: NEOGAP-CRC-01 (EU CT #2024-512296-13-00)

Reviewer Summary & General Impression: The authors present an early-stage study protocol for an ongoing Phase I/IIa clinical trial. This study is investigating Personalized Tumor-Trained Lymphocytes (pTTL) in Stage IV Colorectal Cancer (CRC). The regulatory baseline, Good Clinical Practice (GCP) alignment under EU Regulation No 536/2014, and Institutional Review Board (IRB) safeguards are excellent, thorough, and highly legitimate. However, because this study represents a novel autologous, non-genetically modified T-cell approach utilizing specialized platform technologies (PIOR and EpiTCer), the protocol lacks essential structural definitions. Particularly, the cell mechanism and patient selection parameters. Given that the protocol involves lymphodepleting preconditioning (cyclophosphamide/fludarabine), the safety thresholds particularly related to systemic toxicities like Cytokine Release Syndrome (CRS) which require immediate tightening before publication.

Major Comments & Actionable Revisions:

1. Naming Convention & Title Overstatement: The title claims this is a "novel T cell immunotherapy." While the selection platform (PIOR and activation technology EpiTCer) are proprietary, the concept of using tumor-draining regional lymph nodes (RLNs) to expand tumor-reactive lymphocytes shares fundamental lineages with traditional Tumor-Infiltrating Lymphocyte (TIL) therapies. It is mathematically and historically incorrect to market the broad umbrella of the therapy as completely unprecedented.

Therefore, authors should refine the title or strictly clarify in the introduction what makes "Personalized Tumor Trained Lymphocytes" distinct from standard TIL or Marrow-Infiltrating Lymphocyte (MIL) protocols. Consider changing "novel T cell immunotherapy" to "novel neoantigen-selected T cell manufacturing approach (pTTL).

2. Lack of HLA Restriction & Neoantigen Characterization: The abstract notes that optimal neoantigen epitopes are identified via bioinformatics and loaded onto paramagnetic particles (TC0301). However, the manuscript fails to mention the targeted Human Leukocyte Antigen (HLA) framework. It is unclear whether this platform is limited to HLA Class I (CD8+ restrictive), HLA Class II (CD4+ restrictive), or a pan-HLA presentation.

Therefore, authors must define the mechanistic scope of the PIOR algorithm. Does it select peptides with predicted binding affinity for both MHC-I and MHC-II? Providing the baseline parameters of the target mutations/peptide lengths is critical for peer assessment of the trial's scientific validity.

3. Patient Selection Safeguards: Cytokine Release Syndrome (CRS) & Lymphodepletion: The protocol utilizes a fludarabine/cyclophosphamide lymphodepletion preconditioning regimen. While the authors state that pTTL has "comparably mild side effects," cellular infusions following lymphodepletion carry an inherent clinical risk of CRS, immune effector cell-associated neurotoxicity syndrome (ICANS), or severe cytopenias. Recruiting unselected Stage IV CRC patients who may be heavily pretreated, cachectic, or immunologically depleted, without rigorous baseline physiological filters poses a severe safety vulnerability.

Therefore, the inclusion criteria may include a strict physiological threshold to ensure patients can tolerate potential cytokine storms and preconditioning stress. The authors should incorporate specific enrollment criteria based on: Body Mass Index (BMI) and/or Cachexia Status. To screen out severe cancer cachexia, which compromises cardiac and respiratory reserves during systemic immune activation.

4. Definition of Cell Yield and Dose Limits: The protocol states that "between the limits of 20x106 - 1x109 cells, the entire pTTL yield will be administered." A fifty-fold variance between the minimum and maximum cell dose variables need to be consideration into this Phase I safety trial.

Authors should elaborate or clarify how they intend to correlate toxicity endpoints if Patient A receives 20x106 cells and Patient B receives 1x109 cells.

Minor Comments & Ethical Verification:

The ethical frameworks outlined under the Declaration of Helsinki and the explicit coverage under regular patient insurance for any trial-related harm are verified as exemplary for an active EU clinical trial.

Efficacy Confounders: The authors rightly acknowledge in the discussion that interpreting efficacy will be highly challenging due to the heterogeneity of advanced colorectal cancer. This transparency is above the level.

**********

what does this mean?). If published, this will include your full peer review and any attached files.

If you choose “no”, your identity will remain anonymous but your review may still be made public.

**Do you want your identity to be public for this peer review?**  For information about this choice, including consent withdrawal, please see our Privacy Policy

Reviewer #1: No

Reviewer #2: **Yes:** Nannan Pang

Reviewer #3: No

**********

[NOTE: If reviewer comments were submitted as an attachment file, they will be attached to this email and accessible via the submission site. Please log into your account, locate the manuscript record, and check for the action link "View Attachments". If this link does not appear, there are no attachment files.]

To ensure your figures meet our technical requirements, please review our figure guidelines: https://journals.plos.org/plosone/s/figures

You may also use PLOS’s free figure tool, NAAS, to help you prepare publication quality figures: https://journals.plos.org/plosone/s/figures#loc-tools-for-figure-preparation.

NAAS will assess whether your figures meet our technical requirements by comparing each figure against our figure specifications.

# Author comment: Author response to Decision Letter 1

**DOI:** 10.1371/journal.pone.0351697.r002

12 Aug 2026

Editors´ comments

- Comment 1:

Please ensure that your manuscript meets PLOS ONE's style requirements, including those for file naming.

Response: The manuscript has now been updated in accordance with the PLOS ONE style guidelines.

- Comment 2:

You indicated that you had ethical approval for your study. In your Methods section, please ensure you have also stated whether you obtained consent from parents or guardians of the minors included in the study or whether the research ethics committee or IRB specifically waived the need for their consent.

Response: Under Eligibility the manuscript states: “The target group of the study is adults (≥18 years of age) with stage IV CRC”. To further clarify the eligibility criteria, we have revised this statement to: “The target group of the study is adults (≥18 years of age) with stage IV CRC who are able to provide written informed consent.” A sentence has been added to the Ethical considerations section for clarification, stating: “Subjects ≥18 years are eligble for participation in the study and their informed concent is obtained prior to trial inclusion.”

- Comment 3:

We note that you have selected “Clinical Trial” as your article type. PLOS ONE requires that all clinical trials are registered in an appropriate registry (the WHO list of approved registries. Please state the name of the registry and the registration number in the submission data and on the title page of your manuscript.

Response: The trial is registered in ClinicalTrials.gov with the ID NCT05908643. The registration number is now moved to the title page.

a) Please provide the complete date range for participant recruitment and follow-up in the methods section of your manuscript.

Response: The complete date range, including the date of study opening and the projected date of completion, is now clearly stated in the Methods section under the heading “Trial status”.

b) Trial registration:

Response: The trial is authorized under the Clinical Trial Regulation (Regulation EU No 536/2014), EU CT #2024-512296-13-00. It is registered on clinicaltrials.gov, ID NCT05908643.

- Comment 4:

Your ethics statement should only appear in the Methods section of your manuscript. If your ethics statement is written in any section besides the Methods, please delete it from any other section.

Response: the ethics statement is only mentioned in the Methods section.

- Comment 5:

Please provide a complete Data Availability Statement in the submission form, ensuring you include all necessary access information or a reason for why you are unable to make your data freely accessible. If your research concerns only data provided within your submission, please write "All data are in the manuscript and/or supporting information files" as your Data Availability Statement.

Response: All data are in the manuscript and/or supporting information files. A statement to that effect has been added on page 3 under Data availability and on page 21, under the heading Data management.

- Comment 6:

Role of Funders.

Response: As previously stated, the study is sponsored by Neogap Therapeutics AB (Neogap), and Neogap is in consequence responsible for the study design, protocol development, and preparation of the manuscript. The European Innovation Council provided co-funding but had no role in study design, data collection and analysis, decision to publish, or preparation of the manuscript. As requested, this amended Role of Funder statement has also been included in the manuscript.

- Comment 7:

The competing interests’ section.

Response: We have updated the Competing Interests statement by including the requested statement confirming that the declared competing interests do not alter our adherence to PLOS ONE policies on sharing data and materials. The updated Competing Interests statement has also been included in the cover letter.

- Comment 8:

Please amend either the title on the online submission form (via Edit Submission) or the title in the manuscript so that they are identical.

Response: The title has been corrected and is now identical in both the online submission form and the manuscript.

- Comment 9:

Please amend your authorship list in your manuscript file to include author Ahmed Tarfy.

Response: The Author Ahmed Tarfy is now correctly included in the manuscript.

- Comment 10:

Please amend the manuscript submission data (via Edit Submission) to include author Ahmed Salim Tarfy.

Response: The author's name has been corrected to Ahmed Tarfy and is now used consistently throughout the manuscript and the submission system.

- Comment 11:

Please include a caption for figure 1.

Response: A caption has now been added to Figure 1 identifying it as the SPIRIT 2025 checklist and describing its purpose.

- Comment 12:

Please include captions for your Supporting Information files at the end of your manuscript, and update any in-text citations to match accordingly.

Response: We have added a new section entitled Supporting Information after the References section, where captions for all supporting information are provided.

Reviewers' comments:

Reviewer #1. We thank the reviewer for all valuable comments, which will improve the clarity of the description of statistical analysis. Please find our point-by-point responses below:

- Comment 1:

There is no specification of statistical tests (e.g., methods for time-to-event analyses, comparisons, or handling of exploratory endpoints).

Response: As this is a first-in-human, single-arm phase I/IIa study primarily designed to evaluate safety and feasibility, no formal hypothesis testing is planned. We have revised the Statistical Analysis section to clarify that safety data will be summarized descriptively, time-to-event endpoints will be presented using Kaplan–Meier methodology, and exploratory endpoints will be reported descriptively.

- Comment 2:

The protocol does not describe methods for handling missing data, which is important in longitudinal and follow-up analyses.

Response: The Statistical Analysis section has been revised to clarify that missing data will not be imputed, analyses will be based on available data, and the number of evaluable participants will be reported for each endpoint.

- Comment 3:

There is no discussion of multiplicity or control of type I error, particularly given multiple secondary and exploratory endpoints.

Response: As this first-in-human phase I/IIa study is not designed for confirmatory hypothesis testing, no formal control of the type I error rate or adjustment for multiplicity is planned. We have clarified this in the Statistical Analysis section and specified that secondary and exploratory endpoints will be analysed descriptively.

- Comment 4:

The protocol lacks detail on analysis populations (e.g., safety, intent-to-treat, per-protocol).

Response: The Statistical Analysis section has been revised to specify that the Safety Population will include all participants who receive a pTTL infusion, while secondary and exploratory endpoints will be analysed using participants with evaluable data for the respective endpoint.

- Comment 5:

While data capture software is specified, the statistical software for analysis is not identified.

Response: All descriptive summaries and statistical analyses will be performed using SAS Version 9.4 or later (SAS Institute, Inc., Cary, NC). The statistical software is now available in the manuscript.

- Comment 6:

There is limited detail on planned data presentation (e.g., confidence intervals, or estimation approaches).

Response: A separate section with the subheading “Data presentation” has been added at the end of the statistical analysis section to clarify this question.

Comment 7:

Overall, the statistical section would benefit from a more detailed and prespecified analysis framework to enhance transparency and reproducibility.

Response: The authors thank the reviewer for turning our attention to the need for further information in the statistical analysis section.

In response to the comments, we have revised and expanded the Statistical Analysis section to provide a more detailed and prespecified analysis framework, including the planned analyses, analysis populations, handling of missing data, data presentation, and statistical software. We believe these revisions improve the transparency and reproducibility of the protocol.

Reviewer #2. We thank the reviewer for all valuable comments. Please find our point-by-point responses below:

- Comment 1:

This work was supported by Neogap Therapeutics AB for the development of pTTL. Suggestions from related experts should be carefully considered by the editors of Plos One for the possible competing interests.

Response: The funding source and all potential competing interests have been fully disclosed in the manuscript and the cover letter in accordance with the PLOS ONE requirements.

- Comment 2:

In the background, Reference 10-21 were cited as a whole to support TIL therapy, which was inappropriate.

Response: The references were intended to support the entire paragraph rather than only the statements related to TIL therapy. To improve clarity, we have revised the paragraph by assigning references to the specific statements they support. References relating to TIL therapy are now cited separately from those describing other forms of adoptive T-cell therapy.

- Comment 3:

Was pTTL successful for the treatment of metastatic melanoma or gastrointestinal tumours cited in the Introduction? What was the traditional or the most successful treatment for colorectal cancer? Why did the protocol choose Stage IV CRC? How to classify the period of CRC? The information about CRC was not enough.

Response: The Introduction already describes the current standard treatment and prognosis of colorectal cancer across different disease stages, including stage IV disease. To further strengthen the clinical background, we have added a paragraph describing the current standard-of-care (SOC) treatment for stage IV colorectal cancer and the limited treatment options available for patients with unresectable disease. As this is a first-in-human study, pTTL has not previously been evaluated in any cancer type. Patients with stage IV CRC were selected because this population has exhausted standard treatment options and represents a group with a high unmet medical need. From a regulatory and ethical perspective, only patients lacking effective treatment option can be treated in a first-in-human trial.

- Comment 4:

Were 16 patients enough? Were there control patients? Was there some comparison plan with other treatments?

Response: The study is a first-in-human, single-arm phase I/IIa trial primarily designed to assess the safety, tolerability, and feasibility of pTTL treatment. The sample size of up to 16 treated participants, with a target of at least 12 evaluable participants, was considered appropriate for the descriptive evaluation of these objectives. The study was not designed or powered to establish comparative efficacy. Accordingly, no control group is included and no formal comparison with other treatments is planned in the first-in-human trial. Secondary efficacy and explorative outcomes will be described within the treated population and interpreted cautiously.

- Comment 5:

Was the time of different phases during the trial enough? For example, the reason why it chose 26 weeks of post-infusion was not clear. And was the 5-year following-up enough?

Response: We have clarified the rationale for the selected follow-up periods in the Methods section. The 26-week follow-up period was selected because the vast majority of treatment-related adverse events are expected to occur within the first six months after pTTL infusion, allowing assessment of the primary safety endpoint and clinical outcomes. The subsequent follow-up of up to 5 years is included in accordance with requirements from the Swedish Medical Products Agency and enables the assessment of long-term safety and survival outcomes.

- Comment 6:

Was eligibility a necessary factor for the patient choosing? For example, it was difficult to understand the trial process and experiment details even like me.

Response: As this is a the first time pTTL is applied on humans, it is critical that the eligibility criteria ensure that the patients can tolerate pTTL treatment, including preconditioning lymphodepletion. This requires good organ function and an overall sufficiently good health status. The patient eligibility criteria and trial design are designed with this in mind, and includes requirements for adequate laboratory test results, a cardiac ejection fraction at minimum 45%, and all patients with any symtoms undergo spirometry to ensure adequate lung function. No specific criteria for weight is included, but a requirement for eastern collaborative oncology group (ECOG) performance status of 0-1, means that only asymptomatic and mildly asymptomatic patients who can perform activities of daily life without effort can be included. This is described in the Eligibilty section of the manuscript. We believe these requirements are sufficient to prevent patients with unacceptable risk for complications from being included.

- Comment 7:

Other successful and unsuccessful protocols should be compared. How to evaluate the feasibility or practicability of this protocol？At least, how to guarantee evaluable 12 patients out of 16 within 5 years?

Response: The Discussion intends to place the present study in the context of previous adoptive T-cell therapy studies, by including references to conventional TIL therapy evaluated in the C-144-01 and M14TIL trials, as well as neoantigen-targeted TCR-engineered T-cell therapy reported by Parkhurst et al. The feasibility of the present protocol will be evaluated descriptively, including successful completion of tissue collection, pTTL manufacturing, treatment administration, and follow-up.

The study aims to fulfil the primary objective to show safety and tolerability of treatment with pTTL, which is anticipated to be feasible by including 12 evaluable participants in Part II. To account for potential attrition before treatment, no fixed upper limit is applied to enrolment in Part I, and recruitment is continuously assessed and adjusted to support achievement of the target number of evaluable treated participants.

Reviewer #3. We thank the reviewer for all important comments. Please find our point-by-point responses below.

- Comment 1:

Naming Convention & Title Overstatement: The title claims this is a "novel T cell immunotherapy." While the selection platform (PIOR and activation technology EpiTCer) are proprietary, the concept of using tumor-draining regional lymph nodes (RLNs) to expand tumor-reactive lymphocytes shares fundamental lineages with traditional Tumor-Infiltrating Lymphocyte (TIL) therapies. It is mathematically and historically incorrect to market the broad umbrella of the therapy as completely unprecedented.

Therefore, authors should refine the title or strictly clarify in the introduction what makes "Personalized Tumor Trained Lymphocytes" distinct from standard TIL or Marrow-Infiltrating Lymphocyte (MIL) protocols. Consider changing "novel T cell immunotherapy" to "novel neoantigen-selected T cell manufacturing approach (pTTL).

Response: We agree that the term novel in the title could be interpreted as implying that the overall concept of tumour-reactive T-cell therapy is unprecedented. To avoid this interpretation, we have revised the title by removing the term novel.

- Comment 2:

Lack of HLA Restriction & Neoantigen Characterization: The abstract notes that optimal neoantigen epitopes are identified via bioinformatics and loaded onto paramagnetic particles (TC0301). However, the manuscript fails to mention the targeted Human Leukocyte Antigen (HLA) framework. It is unclear whether this platform is limited to HLA Class I (CD8+ restrictive), HLA Class II (CD4+ restrictive), or a pan-HLA presentation.

Therefore, authors must define the mechanistic scope of the PIOR algorithm. Does it select peptides with predicted binding affinity for both MHC-I and MHC-II? Providing the baseline parame

Attachment Submitted filename: Response to editors and reviewers.docx

# Peer review: Decision Letter 1

**DOI:** 10.1371/journal.pone.0351697.r003

**Authors:** Michael Burger

20 Aug 2026

A first-in-human, phase I/IIa trial of the T cell immunotherapy personalised tumour trained lymphocytes in patients with advanced colorectal cancer: study protocol

PONE-D-26-15858R1

Dear Dr. Berglund,

We’re pleased to inform you that your manuscript has been judged scientifically suitable for publication and will be formally accepted for publication once it meets all outstanding technical requirements.

Within one week, you’ll receive an e-mail detailing the required amendments. When these have been addressed, you’ll receive a formal acceptance letter and your manuscript will be scheduled for publication.

An invoice will be generated when your article is formally accepted. Please note, if your institution has a publishing partnership with PLOS and your article meets the relevant criteria, all or part of your publication costs will be covered. Please make sure your user information is up-to-date by logging into Editorial Manager at Editorial Manager® and clicking the ‘Update My Information' link at the top of the page. For questions related to billing, please contact billing support.

If your institution or institutions have a press office, please notify them about your upcoming paper to help maximize its impact. If they’ll be preparing press materials, please inform our press team as soon as possible -- no later than 48 hours after receiving the formal acceptance. Your manuscript will remain under strict press embargo until 2 pm Eastern Time on the date of publication. For more information, please contact onepress@plos.org.

Kind regards,

Michael C Burger, M.D.

Academic Editor

PLOS One

Additional Editor Comments (optional):

Reviewers' comments:

Reviewer's Responses to Questions

**Comments to the Author**

1. Does the manuscript provide a valid rationale for the proposed study, with clearly identified and justified research questions?

Reviewer #1: Yes

**********

2. Is the protocol technically sound and planned in a manner that will lead to a meaningful outcome and allow testing the stated hypotheses?

Reviewer #1: Yes

**********

3. Is the methodology feasible and described in sufficient detail to allow the work to be replicable?

Reviewer #1: Yes

**********

4. Have the authors described where all data underlying the findings will be made available when the study is complete?

The PLOS Data policy

Reviewer #1: Yes

**********

5. Is the manuscript presented in an intelligible fashion and written in standard English?

Reviewer #1: Yes

**********

Please use the space provided to explain your answers to the questions above and, if applicable, provide comments about issues authors must address before this protocol can be accepted for publication. You may also include additional comments for the author, including concerns about research or publication ethics.

You may also provide optional suggestions and comments to authors that they might find helpful in planning their study.

Reviewer #1: Suggested Minor Revisions:

1- Provide confidence intervals for key safety and efficacy estimates whenever feasible, as the current statistical analysis section focuses primarily on descriptive summaries and does not explicitly state whether confidence intervals will be reported.

2- Clarify how censoring will be handled for overall survival, progression-free survival, disease-specific survival, and time-to-progression analyses displayed using Kaplan-Meier methods.

3- Define what constitutes an “evaluable” participant for each secondary and exploratory endpoint, since analyses are planned using participants with evaluable data.

4- Describe how feasibility outcomes will be summarized quantitatively, given that feasibility is stated as a key study objective.

**********

what does this mean?). If published, this will include your full peer review and any attached files.

If you choose “no”, your identity will remain anonymous but your review may still be made public.

**Do you want your identity to be public for this peer review?**  For information about this choice, including consent withdrawal, please see our Privacy Policy

Reviewer #1: No

**********

# Editor report: Acceptance letter

**DOI:** 10.1371/journal.pone.0351697.r004

**Authors:** Michael Burger

PONE-D-26-15858R1

PLOS One

Dear Dr. Berglund,

I'm pleased to inform you that your manuscript has been deemed suitable for publication in PLOS One. Congratulations! Your manuscript is now being handed over to our production team.

At this stage, our production department will prepare your paper for publication. This includes ensuring the following:

* All references, tables, and figures are properly cited

* All relevant supporting information is included in the manuscript submission,

* There are no issues that prevent the paper from being properly typeset

You will receive further instructions from the production team, including instructions on how to review your proof when it is ready. Please keep in mind that we are working through a large volume of accepted articles, so please give us a few days to review your paper and let you know the next and final steps.

Lastly, if your institution or institutions have a press office, please let them know about your upcoming paper now to help maximize its impact. If they'll be preparing press materials, please inform our press team within the next 48 hours. Your manuscript will remain under strict press embargo until 2 pm Eastern Time on the date of publication. For more information, please contact onepress@plos.org.

You will receive an invoice from PLOS for your publication fee after your manuscript has reached the completed accept phase. If you receive an email requesting payment before acceptance or for any other service, this may be a phishing scheme. Learn how to identify phishing emails and protect your accounts at https://explore.plos.org/phishing.

If we can help with anything else, please email us at customercare@plos.org.

Thank you for submitting your work to PLOS One and supporting open access.

Kind regards,

PLOS One Editorial Office Staff

on behalf of

Dr. Michael C Burger

Academic Editor

PLOS One
